# Rhodium-catalysed tetradehydro-Diels–Alder reactions of enediynes *via* a rhodium-stabilized cyclic allene†

**DOI:** 10.1039/d0sc04390g

**Published:** 2020-09-04

**Authors:** Srinivas Thadkapally, Kaveh Farshadfar, Melanie A. Drew, Christopher Richardson, Alireza Ariafard, Stephen G. Pyne, Christopher J. T. Hyland

**Affiliations:** School of Chemistry and Molecular Bioscience, University of Wollongong Wollongong NSW 2522 Australia chris_hyland@uow.edu.au; School of Physical Sciences, University of Tasmania Hobart TAS 7018 Australia; Department of Chemistry, Islamic Azad University, Central Tehran Branch Poonak Tehran 1469669191 Iran

## Abstract

Efficient methods for the synthesis of fused-aromatic rings is a critical endeavour in the creation of new pharmaceuticals and materials. A direct method for preparing these systems is the tetradehydro-Diels–Alder reaction, however this is limited by the need for harsh reaction conditions. A potential, but underdeveloped, route to these systems is *via* transition metal-catalysed cycloaromatisation of ene-diynes. Herein, tethered unconjugated enediynes have been shown to undergo a facile room-temperature Rh^I^-catalysed intramolecular tetradehydro-Diels–Alder reaction to produce highly substituted isobenzofurans, isoindolines and an indane. Furthermore, experimental and computational studies suggest a novel mechanism involving an unprecedented and complex Rh^I^/Rh^III^/Rh^I^/Rh^III^ redox cycle involving the formation of an unusual strained 7-membered rhodacyclic allene intermediate and a Rh^III^-stabilized 6-membered ring allene complex.

## Abstract 2

Room temperature Rh-catalysed tetradehydro-Diels–Alder reaction *via* an unusual Rh-stabilised allene.

## Graphical abstract

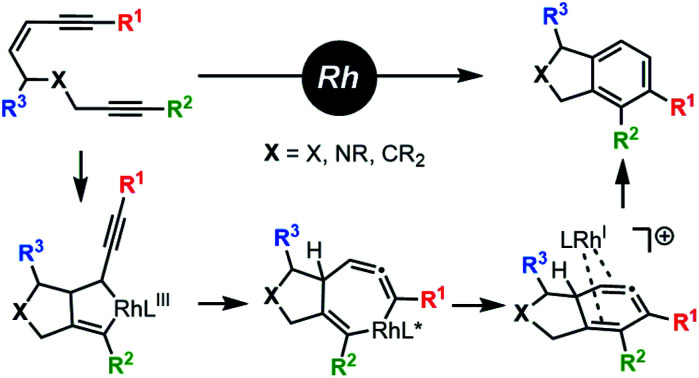

## Introduction

**Scheme 1:**
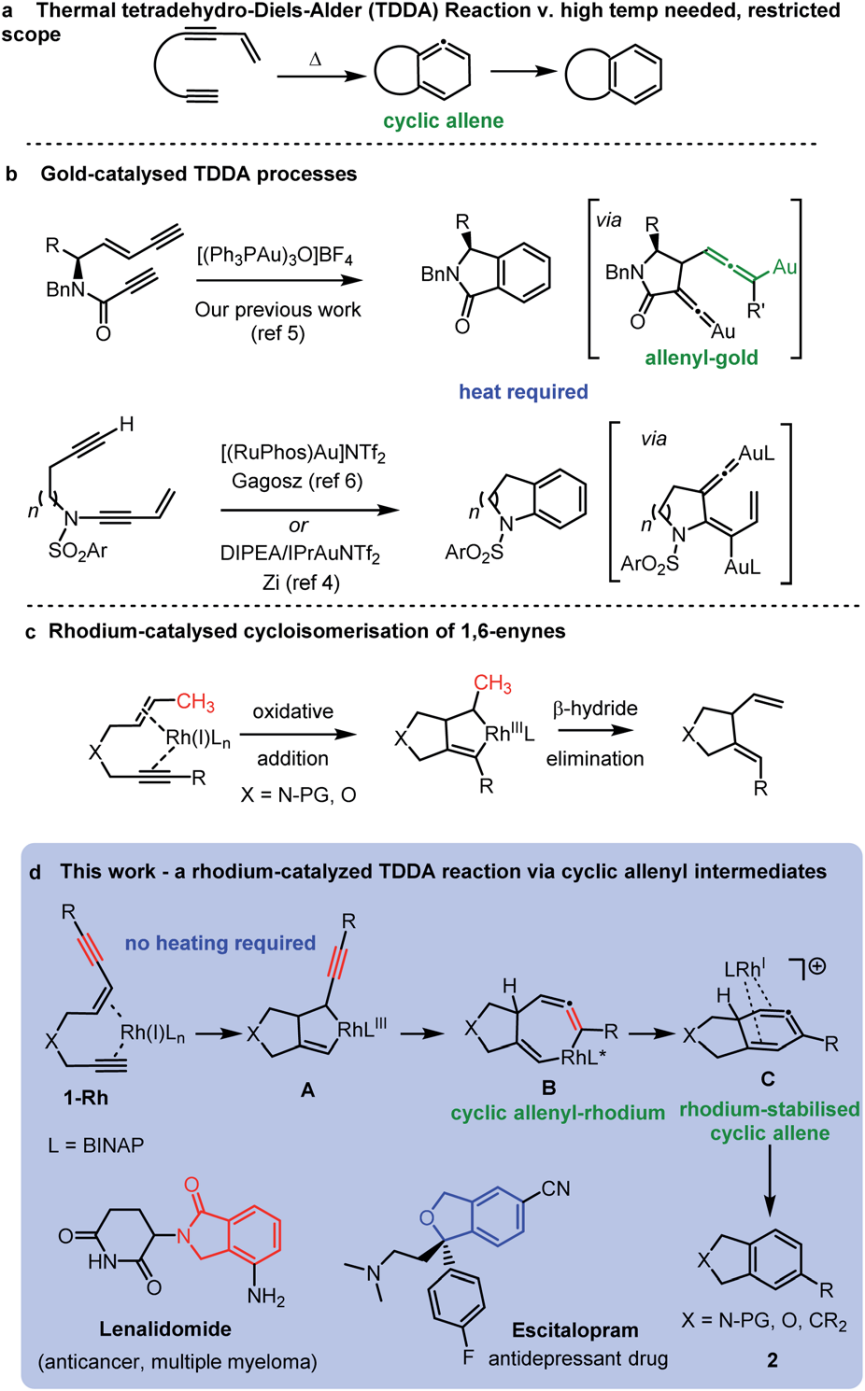
Tetradehydro-Diels–Alder reactions of enediynes: (a) thermal; (b) gold-catalysed; and (c) rhodium catalysed processes.

New methods for the construction of functionalized aromatic rings is an important area of research due to their wide occurrence in bioactive molecules and materials. An advanced strategy for preparing functionalized aromatic rings is cycloaromatisation of polyunsaturated acyclic building blocks, including, enediynes, enyne-allenes and dienynes.^1^ The tetradehydro-Diels–Alder (TDDA) reaction of polyunsaturated enediyne systems is of particular note as it allows for the direct formation of a benzenoid ring in a single step, *via* an intriguing high energy cyclic allene intermediate, which typically aromatises *via* a hydride shift (Scheme 1a).^2,3^ The thermal mode of this reaction has been investigated for many years, but its synthetic applications are severely limited by the requirement of the process for very forcing reaction conditions and particular substitution patterns on the starting materials.^3^ The transition metal-catalysed variant of the reaction has demonstrated potential for unveiling tetradehydro-Diels–Alder reactions of enediynes that proceed under milder conditions for a greater range of substrates. For example, several recent reports have harnessed dual gold-catalysts in cycloaromatisations *via* TDDA reactions of enediynes.^3–7^ In these cases the enediyne is not fully conjugated but has the alkyne and enyne units linked *via* a heteroatom tether leading to a diverse range of fused heterocyclic benzenoid structures of biological significance (Scheme 1b).

In the case of our previous work,^5^ the key reaction intermediate incorporated a vinylidene gold^8^ intermediate and a rare allenyl gold species (Scheme 1c).

Curiously, there have been no investigations of rhodium-catalysed cycloaromatisation reactions of tethered non-conjugated enediynes – and just a single report of a cycloaromatisation reaction of a conjugated enediyne.^9^ We hypothesized that a rhodium-catalysed cycloaromatisation reaction could be realized by harnessing the well-known oxidative cyclization process observed in Rh-catalysed 1,6-enyne reactions (Scheme 1c).^10–16^ The triple bond of an enediyne opens up the possibility of forming a cyclic vinyl-rhodium species **A***via* oxidative cyclization of ene-diyne **1-Rh**. Isomerization of the oxidative cyclization product **A** should form **B** – providing a direct route to the intriguing allenyl-rhodium intermediate **B** and an alternative route to a cyclic allene *cf.* the thermal TDDA reaction (Scheme 1d). A reductive elimination to the same cyclic allene proposed in the thermal TDDA reaction could then be envisaged en-route to the final fused-aromatic products **2***via* a potential final hydride shift. Herein, we report the successful implementation of this proposed pathway and provide experimental and computational evidence for a rhodium-catalysed TDDA reaction that proceeds *via* an unprecedented cyclic allenyl-rhodium species **B** and a rhodium-stabilised 6-membered cyclic allene **C** involving a complex Rh^I^/Rh^III^/Rh^I^/Rh^III^ redox cycle. The resulting isobenzofuran,^17^ isoindoline,^18,19^ isoindolinone^20^ and indane^21^ products are important heterocycles that are found in an array of important bioactive and medicinally important compounds.

## Results and discussion

**Scheme 2:**
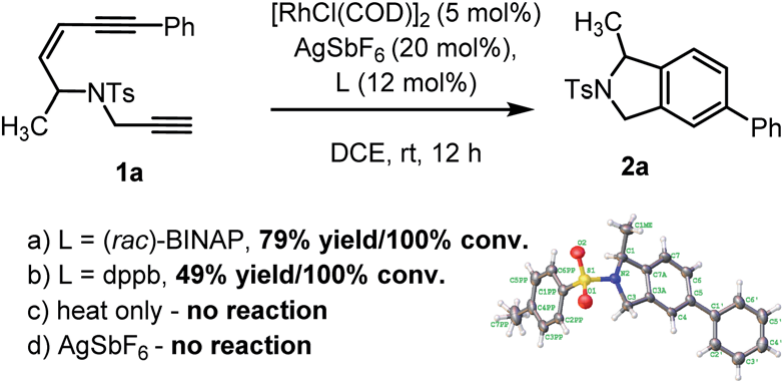
Initial cycloaromatisation reaction discovery using: (a) L = (*rac*)-BINAP; (b) L = dppb; (c) heat only; or (d) AgSbF_6_ alone.

To examine our hypothesis the NTs-tethered (*Z*)-enediyne **1a** was synthesized (see ESI†) as a model substrate and treated with [RhCl(COD)]_2_/AgSbF_6_ and (*rac*)-BINAP at rt in DCE – a general system that has previously proved successful in catalyzing the cycloisomerization of 1,6-enynes.^16^ Gratifyingly, this proved a highly efficient catalyst for the cycloaromatisation of **1a** to give isoindoline **2a** in high yield (Scheme 2a – structure determined unambiguously by X-ray crystallography).^22^ Replacing the ligand BINAP with dppb, which has a similar bite angle, had a deleterious effect on the yield (Scheme 2b). Starting material was recovered when **1a** was heated at reflux in DCE in the absence of catalyst, indicating that there is no competing non-catalysed TDDA reaction occurring (Scheme 2c).

**Chart 1:**
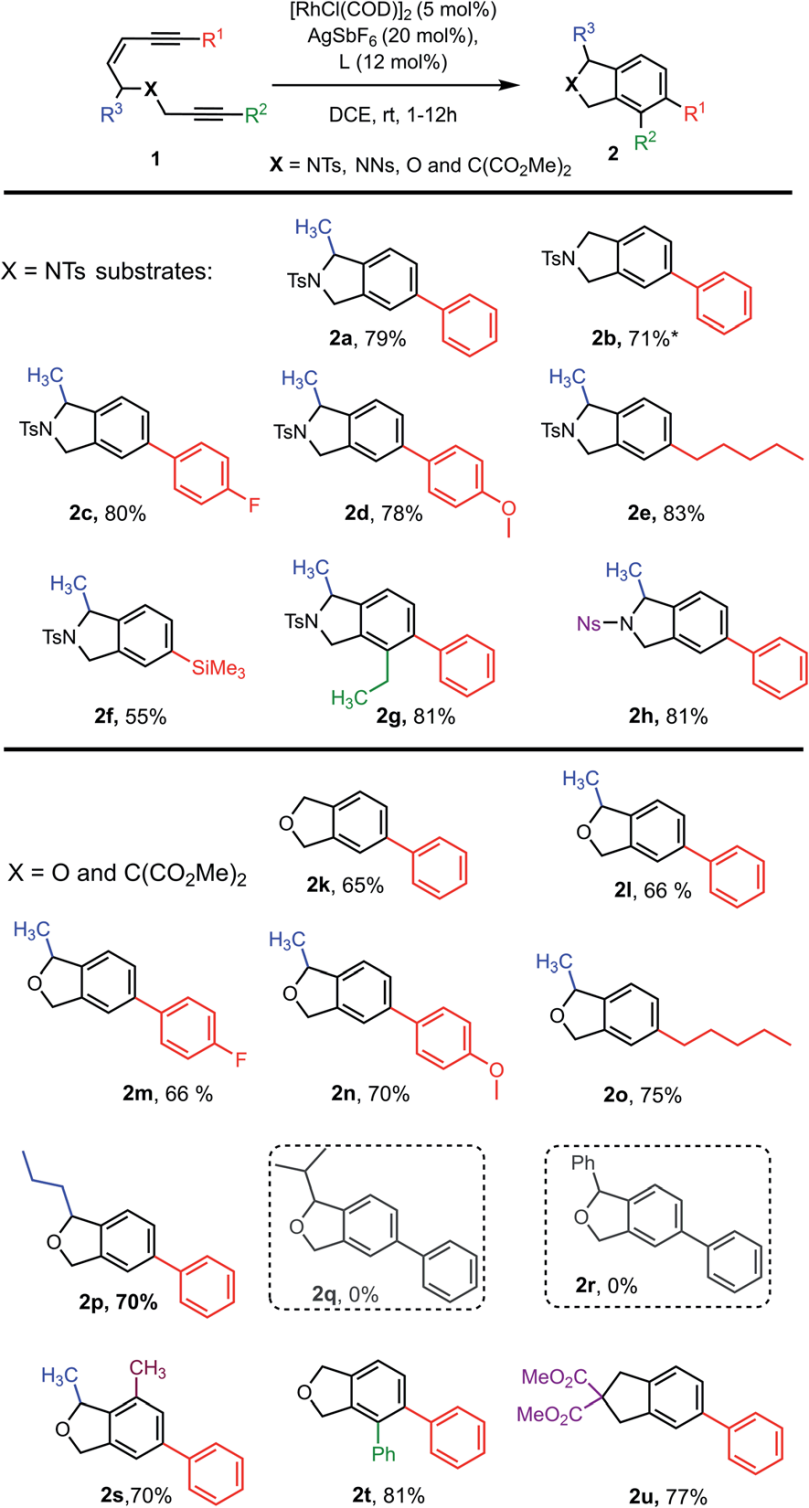
Substrate scope for Rh-catalysed tetradehydro-Diels–Alder reactions of enediynes **1**. ^a^The starting material for **2d** and **2f** were a mixture of *E*/*Z* isomers. *2.5 mol% of the Rh-catalyst gave the product in 10% yield (17% conversion) or 19% yield (38% conversion) at 80 °C.

The presence of AgSbF_6_ was essential as the reaction with [RhCl(COD)]_2_ alone resulting in no conversion. Replacing AgSbF_6_ with AgBF_4_ also proved detrimental giving only a 60% yield of isoindoline **2a** (80% conversion). Furthermore, lowering the Rh-catalyst loading to 2.5 mol% resulted in a significantly reduced conversion (75%) and yield (44%) over 12 h. This is even more striking for substrate **1b** to give **2b** (see Chart 1), where 2.5 mol% of the Rh-catalyst gave the product in 10% yield (17% conversion) or 19% yield (38% conversion) at 80 °C. Finally, changing the solvent to DCM reduced the yield to 62%.

With an effective catalyst system identified for the Rh-catalysed TDDA reaction, the substrate scope was established with a range of N- and O-tethered enediynes **1**, as shown in Chart 1. For both the N- and O-tethered enediynes, aryl (products **2a–d**, **2g–h**, **2k–n**, **2p** and **2s–2u**), alkyl (products **2e** and **2o**) and silyl (product **2f**) groups at R^1^ could be accommodated. Both R^1^ and R^2^ could be simultaneously substituted to provide more densely functionalised isoindolines and isobenzofurans **2g** and **2t**, respectively. The R^3^ substituent could be a hydrogen (products **2b**, **2k** and **2t**) or accommodate an *n*-propyl group in place of the methyl group (product **2p**). However, branched substituents or a phenyl ring (to give predicted products **2q** and **2r**) were not tolerated, presumably for steric reasons – in these cases only decomposition of starting material was observed. The dimethyl substituted O-tethered enediyne **1s** was also well tolerated under the cycloaromatisation reaction conditions to give trisubstituted isobenzofuran **2s** in good yield. Isoindoline **2h** formed smoothly from nosyl-protected enediyne **1h**. Other protecting groups on N were not investigated due to the established instability of isoindolines without an electron-withdrawing N group.^23–27^ Finally, a carbon-tethered enediyne **1u** was prepared and found to undergo smooth cycloaromatisation to trisubstituted indane **2u**.

**Scheme 3:**
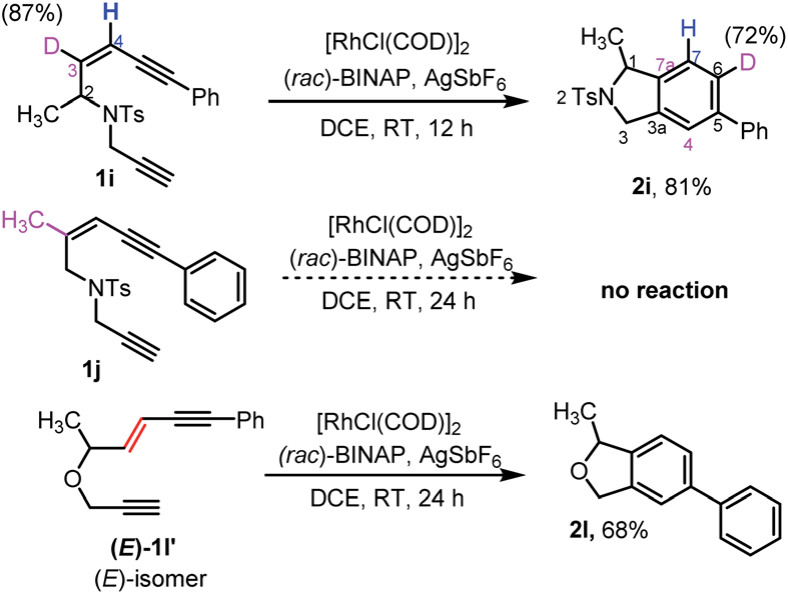
Experiments carried out to probe the reaction mechanism.

**Fig. 1:**
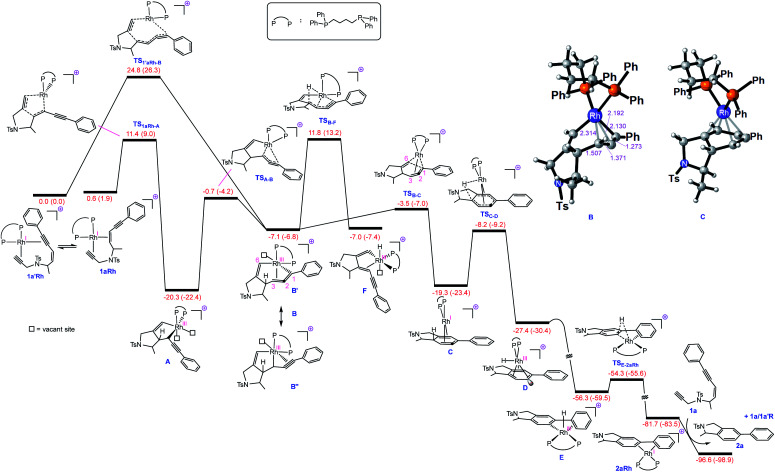
Free energy profile for Rh-catalysed tetradehydro-Diels–Alder reaction of a (*Z*)-enediyne. Free energies (potential energies) are given in kcal mol^−1^.

Our attention then turned to experimentally and computationally investigating the reaction mechanism (Scheme 3 and Fig. 1). The C3-position in the ene-diyne starting material **1** (X = O or NTs) is key to understanding the reaction mechanism as it becomes one of the carbons (C7a) fusing the two rings of the product **2***via* migration of the attached hydrogen. For the thermal TDDA reaction this typically occurs *via* one or more hydride shifts. To understand this process for the current system, a C3-deuterated analogue **1i** and a C3-methylated analogue **1j** were prepared (Scheme 3). To determine the fate of the C3-hydrogen, deuterated substrate **1i** was subjected to the reaction conditions, leading to predominant deuterium incorporation at the C6-position of the isoindoline benzenoid ring of **2i**. The partial loss of deuterium at the C6-position of the product pointed away from a hydride shift in intermediate **B** or **C** to yield the aromatic product and hinted at the potential formation of a rhodium-hydride species. A substrate (**1j**) with a methyl group on the enyne double bond stopped the reaction completely – again suggesting a key role for this position in the reaction. The sensitivity of the cycloaromatisation reaction towards the double bond geometry was also probed by utilizing (*E*)-enediyne **1l′**, which led to the formation of the isobenzofuran **2l** in comparable yield to its corresponding (*Z*)-enediyne starting material **1l**.

With this set of key experimental results in place, we turned attention to DFT calculations for further mechanistic insight, considering our proposed cyclic allenyl-Rh intermediate **B** (Fig. 1d).^28^ In contrast to simple 1,6-enynes, the initial rhodium-catalysed oxidative cyclization has two coordination possibilities – coordination between the enyne double bond and the triple bond of the propargyl sulfonamide (**1aRh**), or between the two triple bonds of the enediyne (**1a′Rh**). While oxidative cyclization *via***1a′Rh** would directly afford the proposed allenyl-Rh^III^ intermediate **B**, the barrier for this process was significantly higher than that for the oxidative cyclization *via***1aRh** to provide Rh^III^ metallacyclopentene **A**. Following this exergonic process, an endergonic ring-expansion transformation into the key Rh^III^ intermediate **B** occurs. This unusual intermediate can be represented by two resonance contributors: an allenyl-Rh^III^ intermediate **B′** and a propargyl Rh^III^**B′′** and its hybrid structure (top corner of Fig. 1) is indicative of a bent allenyl rhodium species. The ensuing allenyl-Rh intermediate is a branching point for two different reaction channels: (a) C–H elimination *via* transition structure **TSB-F** affording **F** and (b) C–C reductive elimination *via* transition structure **TSB-C** yielding strained Rh^I^-stabilised cyclic allene **C**.

Surprisingly, the C–C reductive elimination process is computed to be significantly more favourable than the C–H elimination. This is because the allenyl Rh resonance contributor **B′** makes a significant contribution to the overall structure **B** and this means that there is significant σ-bond character between the Rh and C1, thus promoting reductive elimination. The intermediate resulting from the reductive elimination can be considered as a stabilized version of the cyclic allene invoked in the thermal TDDA reactions of enediynes – thereby playing a key role in making the rhodium catalysed variant so facile (Scheme 1a). The catalytic cycle continues with the allene-bound Rh^I^ intermediate **C** undergoing oxidative addition to the C–H bond to form Rh^III^-hydride **D**, with the rhodium being bound to one face of the aromatic ring, having the formal anion at C6. This result explains the failure of substrate **1j** in the reaction due to the low propensity of C–C bonds to undergo oxidative addition compared with C–H bonds.^29^ This is also an interesting mechanistic point of difference to the thermal TDDA reaction, wherein formation of the final aromatic ring typically occurs *via* hydride-migration. In the current case, coordination of Rh to the π-system of intermediate **C** directs reactivity away from such a hydride migration and towards a C–H insertion.

**Fig. 2:**
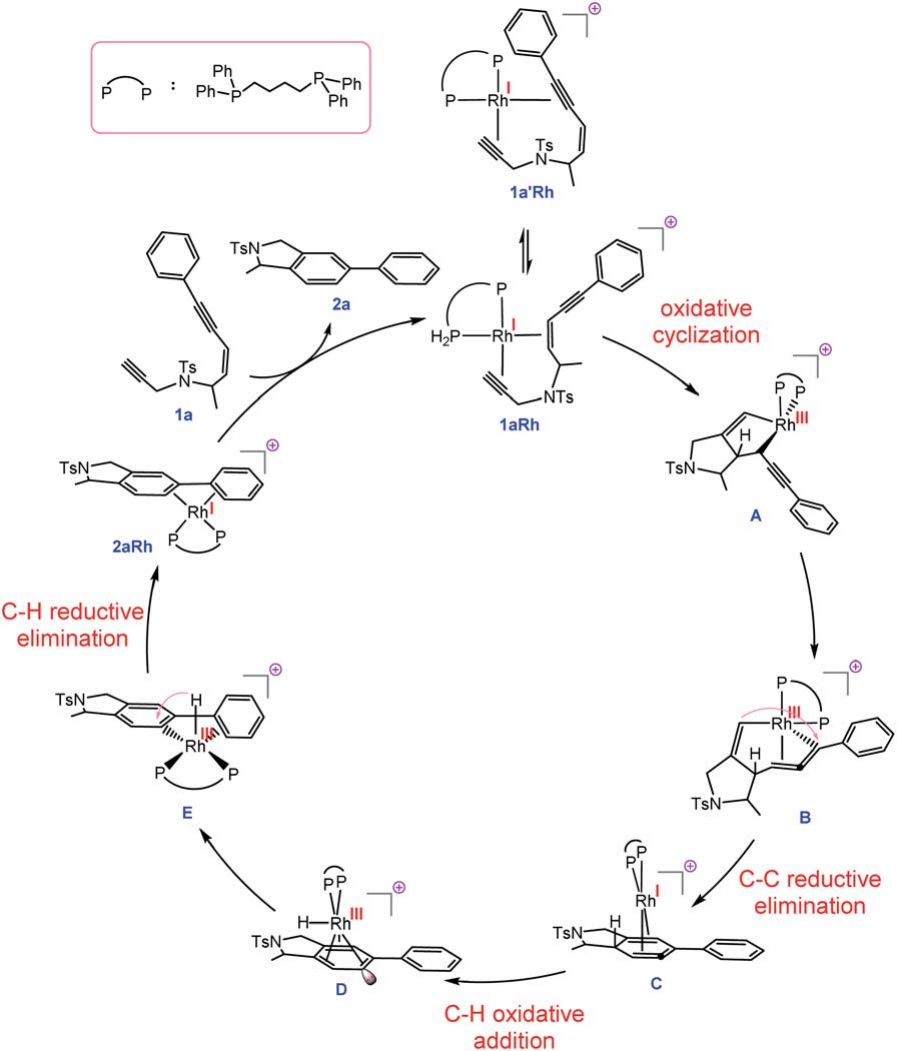
Proposed mechanism for the transformation **1a** → **2a** catalysed by a Rh complex involving Rh^I^/Rh^III^/Rh^I^/Rh^III^ cycle and allenic-intermediate **C**.

The hydride complex **D** undergoes rearrangement to **E**, which is primed for a highly favourable H–C6 reductive elimination. Once this has taken place, the organic product **2a** is formed and the Rh^I^ catalyst re-enters the catalytic cycle. This mechanism explains the incorporation of deuterium at C6, with some scrambling likely occurring between rhodium hydrides **D**/**E** and solvent/adventitious water. These calculations and the experimental studies enable the catalytic cycle in Fig. 2 to be proposed. Attempts to trap cyclic allene (derived from ene-diyne **1a** or **1j**) intermediate **C***via* Diels–Alder reactions with furan were not successful, which is due to the favorable oxidative addition towards intermediate **D** – which is a fast intramolecular process – as well as the coordination of Rh to the resulting cyclic allene.

**Scheme 4:**
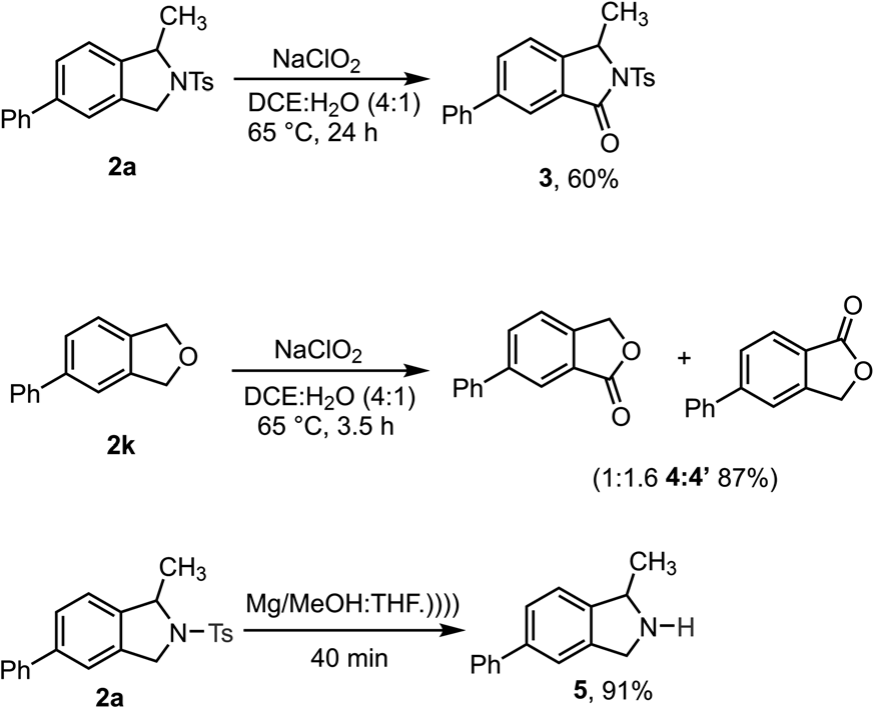
Synthetic modifications of isoindoline and isobenzofuran scaffolds.

Synthetic transformations were performed on the isoindoline and isobenzofuran products to demonstrate their utility (Scheme 4). Initially, **2a** and **2k** were converted to biologically important isoindolinone scaffolds **3** and isobenzofuranone **4** : **4′** respectively, using sodium chlorite as a mild and green oxidizing reagent.^30^ Gratifyingly, the N-Ts group could be readily removed from **2a** upon sonication in the presence of Mg/MeOH to produce 1-methyl-5-phenylisoindoline **5** in excellent yield – it should be noted, however, that this product is unstable and cannot be purified by chromatography.^31^

## Conclusions

In conclusion, a mild and versatile rhodium-catalysed tetradehydro-Diels–Alder reaction of enediynes has been developed allowing the synthesis of substituted, biologically important isoindolines, isobenzofurans and indanes. DFT calculations show that the reaction proceeds through an unprecedented Rh^I^/Rh^III^/Rh^I^/Rh^III^ catalytic cascade (Fig. 2) that includes two unusual organometallic allene-intermediates. As such, the reaction represents a conceptually new way to harness *in situ* generated metal-allenyl species through catalytic cycloisomerization reactions at room temperature. It also allows for formation of a low energy rhodium-stabilized cyclic allene intermediate and contributes to this catalytic process being more favourable and wider in scope than the thermal TDDA reaction. This intermediate is of particular interest given that cyclic allenes are emerging as powerful building blocks in modern synthesis and the new conceptual approach described here should lead to further developments in this field.^32–34^ Further work on generating and harnessing allenic-rhodium and rhodium-stabilized allene species in catalytic processes is currently underway.

## Conflicts of interest

There are no conflicts to declare.

## Supplementary Material

SC-011-D0SC04390G-s001

SC-011-D0SC04390G-s002
