# Supplementary material for: Rhodium-catalysed tetradehydro-Diels–Alder reactions of enediynes via a rhodium-stabilized cyclic allene
Source: Chem Sci. 2020 Sep 4;11(40):10945–50. doi: 10.1039/d0sc04390g (PMC8162385; doi:10.1039/d0sc04390g)
Supplement: SC-011-D0SC04390G-s001 [file SC-011-D0SC04390G-s001.pdf]

Electronic Supplementary Material (ESI) for Chemical Science.
This journal is © The Royal Society of Chemistry 2020

Table of contents

1. General characterization methods 2

2. General synthesis of 1,3-(*Z*)-enyn-ols-(**S5a-l**) and 1,3-(*E*)-enyn-ol-(**S5k**) 3

3. General scheme for the synthesis of tethered-enediynes (**1a-u**) 16

Method-A: Synthesis of N-tethered enediynes (**1a-j**) 16

Method-B: N-tethered enediyne from Mitsunobu coupling reaction (**1g**) 24

Method-C: General procedure for the synthesis of O-tethered enediynes (**1k-s** and **1t**) 27

Method-D: Synthesis of C-tethered enediyne (**1u**) 35

4. General procedure for the rhodium(I)-catalyzed cycloaromatization of enediynes 37

5. Deuterium isotope labelling study 49

6. Controlled mechanistic study 51

7. Stereocontrolled reaction 52

8. Product modification reactions 53

8.1. Oxidation of isoindoline (**2b**) to isoindolinone **3** 53

8.2. Oxidation of isobenzofuran (**2k**) to isobenzofuranones **4** and **4'** 54

9. N-Ts deprotection of 1-methyl-5-phenyl-2-tosylisoindoline (**2b)** 54

10. References 55

11. NMR spectra 57

12. Computational details 112

1. General Characterization Methods

The following general procedures were used for the synthesis of substrates unless otherwise noted. All reagents and solvents were commercially available. Anhydrous solvents were purchased (DCE, DCM, THF, MeCN, acetone) and stored over nitrogen over 4Å molecular sieves. All reactions were conducted under an inert atmosphere of nitrogen in oven dried glassware. Reactions were monitored by thin layer chromatography (TLC). TLC analysis was performed on aluminum on aluminum-backed silica gel sheets (F254 nm), TLC plates were visualized using solution of ($KMnO_4$ or phosphomolybdic acid in ethanol). Synthesized substrates were purified by flash column chromatography using Silica Gel (40-75 nm) as a stationary phase. ^{1}H NMR spectra were recorded at 400 MHz or 500 MHz in $CDCl_3$ and chemical shifts are reported to relative to tetramethylsilane TMS (δ = 0.00). ^{13}C NMR (proton decoupled) spectra were measured in $CDCl_3$ and chemical shifts are reported relative to the solvent resonance (δ = 77.16). ^{1}H NMR data are listed as chemical shift, multiplicity (m), coupling constant (Hz) and integration. The abbreviations used for multiplicity are : singlet (s), doublet (d), triplet (t), triplet of triplets (tt) quartet (q), doublet of doublet (dd), doublet of doublet of doublets (ddd), broad doublet (db), doublet of triplets (dt), doublet of doublets of triplets (ddt) broad (br), Melting points were measured using a Buchi Melting Point M-560 apparatus. Infrared spectra (IR) recorded on a Bruker Vertex 70 FTIR Spectrometer as neat samples. High-resolution mass spectra (HRMS) were generated on a Waters Xevo G1 QTOF mass spectrometer with electrospray ionization. Cambridge Crystallographic Data Centre (CCDC) deposition number for the compound 1999577.

2. General synthesis of 1,3-(*Z*)-enyn-ols-(S5a-l).

The substituted stereodefined 1,3-(*E*-*Z*)-enyn-ol starting materials (**S5a-k**) were prepared according to the scheme below, following literature methods.[1-3]

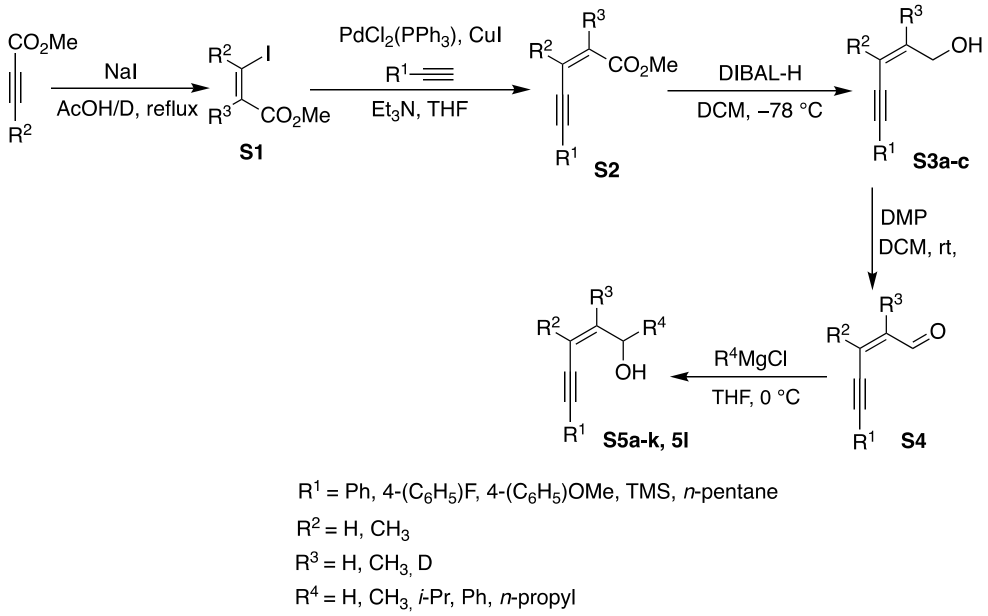

Synthesis of methyl (*Z*)-3-iodoacrylate-2-*d* (S1)

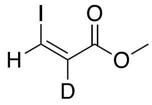

To a solution of sodium iodide (1.33 g, 8.91 mmol, 1.5 equiv) in acetic acid-*d* (99%, 3.49 mL, 1.7 M) at room temperature was added methyl propiolate (0.50 g, 5.94 mmol, 1 equiv) and the solution was then heated at reflux for 16 h. The reaction was cooled to room temperature and diluted with water and ethyl acetate. The organic layer was separated, and the aqueous layer extracted with EtOAc (3×10 mL). The combined extracts were washed with 3 M aqueous potassium hydroxide solution, brine, dried (Na_2SO_4) and concentrated in vacuo to yield the **S1** as a pale-yellow oil (1.16 g, 91%). **^{1}H NMR** (500 MHz, $CDCl_3$) δ 7.48 (d, J = 9.0 Hz, 0.09H) 7.48 (brs, 0.91H, =C*H*), 6.92 (d, J = 9.0 Hz, 0.09 H, =C*H*(D)), 3.79 (s, 3H, OCH_3). **^{13}C NMR** (126 MHz, $CDCl_3$) δ 165.0, 129.2 (t, J_{CD} = 25.3), 95.1, 51.6. **IR** (ν_{max}/cm^{-1}) 1729 (w) 1597 (w),

1346 (m), 1205 (w), 1163 (s), 761 (m), 665 (s). **HRMS** (ESI-OTF) *m/z* calcd for $C_4H_5DO_2I$ [M + H]$^+$ 213.9491, found 213.9490.

Methyl (*Z*)-5-phenylpent-2-en-4-ynoate-2-*d* (S2a)

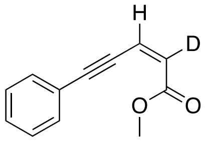

A solution of methyl (*Z*)-3-iodoacrylate-2-*d* (**S1**) (996 mg, 4.67 mmol, 1 equiv) in THF (9.3 mL) was sparged over 30 min with nitrogen then cooled to 0 °C, $PdCl_2(PPh_3)_2$ (32 mg, 0.046 mmol, 1 mol%), CuI (8.9 mg, 0.046 mmol, 1 mol%), triethylamine (1.3 mL, 9.35 mmol 2 equiv) and phenylacetylene (0.51 mL, 4.67 mmol, 1 equiv) were added the mixture was warmed to room temperature and stirred for 16 h. The reaction mixture was filter of through a pad of silica gel and washed with EtOAc. The crude product was purified by flash column chromatography on silica gel using EtOAc/*n*-hexane (0.5:9.5) to give **S2a** (862 mg, 96%) as a colorless oil; R_f (EtOAc/*n*-hexane 1:9) = 0.78. **^{1}H NMR** (500 MHz, $CDCl_3$) δ 7.55 – 7.52 (m, 1H, ArC*H*), 7.43-7.40 (m, 2H, 2ArC*H*) 7.37 – 7.33 (m, 2H, 2ArC*H*), 6.37 (d, *J* = 11.4 Hz, 0.09 H, =C*H*), 6.37 (brs, 0.94H, =C*H*), 6.15 (d, *J* = 11.4 Hz, 0.09H, =C*H*(D)), 3.80 (s, 3H, OCH_3). **^{13}C NMR** (126 MHz, $CDCl_3$) δ 165.2, 132.1, 129.2, 128.4, 127. 4 (t, J_{CD} = 25.7 Hz), 123.1, 122.6, 101.4, 86.9, 51.5. **IR** (ν_{max}/cm^{-1}) 3021(w), 2949 (w), 2199 (m), 1708 (s), 1602 (s), 1489 (s), 1434 (s), 1353 (s), 1241 (s), 1203 (s), 1061 (s), 987 (s), 789 (s), 690 (s), 528 (s). **HRMS** (ESI-OTF) *m/z* calcd for $C_{12}H_9DO_2Na$ [M + Na]$^+$ 210.0657, found 210.0656.

Methyl (*Z*)-5-(4-fluorophenyl)pent-2-en-4-ynoate (S2b)

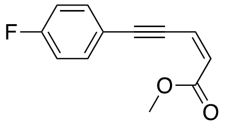

The title compound prepared using the general synthesis procedure for **S2a,** methyl (*Z*)-3-iodoacrylate (882 mg, 4.16 mmol), $PdCl_2(PPh_3)_2$ (29 mg, 0.041 mmol, 1 mol%), CuI (7.9 mg,

0.041 mmol, 1 mol%), triethylamine (1.16 mL, 8.32 mmol 2 equiv) and 1-ethynyl-4-fluorobenzene (0.47 mL, 4.16 mmol, 1 equiv). The crude product was purification by flash column chromatography on silica gel using EtOAc/*n*-hexane (0.5:9.5) eluted as a pale-yellow oil (600 mg, 71%). **^{1}H NMR** (400 MHz, $CDCl_3$) δ 7.53 (dd, *J* = 8.9, 5.4 Hz, 2H), 7.04 (t, *J* = 8.8 Hz, 2H), 6.35 (d, *J* = 11.4 Hz, 1H), 6.16 (d, *J* = 11.4 Hz, 1H), 3.80 (s, 3H). **^{13}C NMR** (101 MHz, $CDCl_3$) δ 165.23, 163.1 (d, J_{CF} = 251.2 Hz), 134.2 (d, J_{CF} = 8.7 Hz), 127.8, 123.1, 118.7 (d, J_{CF} = 3.6 Hz), 115.8 (d, J_{CF} = 22.3 Hz), 100.3, 86.1, 51.5. NMR data matches literature values.[4]

(*Z*)-2-Methyl-5-phenylpent-2-en-4-yn-1-ol (S2c)

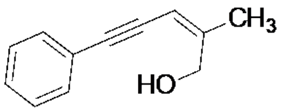

The title compound was obtained as a colourless liquid (211 mg, 87%) after flash column chromatography (EtOAc/*n*-hexane 1.5:8.5) using synthesis procedure for **S2a** using (*Z*)-3-iodo-2-methylprop-2-en-1-ol (280 mg, 1.41 mmol, 1 equiv), dry THF (2.82 mL), $PdCl_2(PPh_3)_2$ (10 mg, 0.014 mmol, 1 mol%), CuI (2.7 mg, 0.014 mmol, 1 mol%), trimethylamine (0.39 mL, 2.28 mmol 2 equiv) and phenylacetylene (0.15 mL, 1.41 mmol, 1 equiv). R_f(EtOAc/*n*-hexane 2:8) = 0.41. **^{1}H NMR (**400 MHz, $CDCl_3$) δ 7.46 – 7.37 (m, 2H, 2ArC*H*), 7.33–7.29 (m, 3H, 3ArC*H*), 5.61 (s, 1H, =C*H*), 4.44 (s, 2H, C*H*$_2$(OH)), 1.94 (d, *J* = 1.6 Hz, 3H, CH_3), 1.81 (bs, 1H, OH). **^{13}C NMR** (101 MHz, $CDCl_3$) δ 150.2, 131.3, 128.3, 128.1, 126.6, 123.4, 93.1, 80.8, 64.1, 20.3. **IR** (ν_{max}/cm^{-1}) 3340 (w), 2970 (m), 1466 (m), 1048 (m), 1160 (m), 1128 (s), 950 (s), 816 (m). **HRMS** (ESI-OTF) *m/z* calcd for $C_{12}H_{13}O$ [M + H]$^+$ 173.0961 found 173.0960.

(*Z*)-5-Phenylpent-2-en-4-yn-2-*d*-1-ol (S3a)

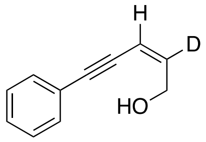

To a solution of methyl **S2a** (600 mg, 3.20 mmol, 1 equiv) in dry CH_2Cl_2 (16 mL, 0.2 M) at −78 °C was added dropwise diisobutylaluminium hydride (1.0 M) in hexane (6.41 mL, 6.41 mmol, 2 equiv). The mixture was stirred at the same temperature for 4 h. 1 M aqueous HCl (3 mL) was added and the mixture was warm to room temperature. The reaction mixture was extracted into CH_2Cl_2 (3 × 4 mL) the combined extracts were dried (Na_2SO_4) and concentrated under vacuo. The crude product was purified by flash column chromatography on silica gel using EtOAc/*n*-hexane (2:8) to give **S3a** (493 mg, 97%) as a colorless liquid; R_f (EtOAc/*n*-hexane 2:8) = 0.38. **^{1}H NMR** (500 MHz, $CDCl_3$) δ 7.46 – 7.40 (m, 2H, 2C*H*Ar), 7.34 – 7.30 (m, 3H, 3C*H*Ar), 5.81 (brs, 1H, =C*H*), 4.49 (d, *J* = 1.6 Hz, 2H, C*H*$_2$(OH)), 1.67 (brs, 1H, O*H*). **^{13}C NMR** (126 MHz, $CDCl_3$) δ 141.8 (t, J_{CD} = 24.4 Hz),131.4, 128.5, 128.3, 122.9, 110.6, 95.2, 84.9, 61,0. **IR** (ν_{max}/cm^{-1}) 3308 (w), 2924 (m), 2205 (m), 1951 (m), 1601 (s), 1488 (s), 1069 (s) 753 (s), 688 (s), 526 (s). **HRMS** (ESI-OTF) *m/z* calcd for $C_{11}H_{10}DO$ [M + H]$^+$ 160.0873, found 160.0878.

(*Z*)-5-(4-Fluorophenyl)pent-2-en-4-yn-1-ol (S3b)

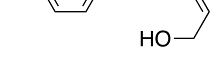

The title compound was obtained as a colorless liquid (242 mg, 94%) after flash column chromatography (ethylacetate/hexane 1.5:8.5) using the synthesis procedure of **S3a** and methyl (*Z*)-5-(4-fluorophenyl)pent-2-en-4-ynoate (300 mg, 1.47 mmol, 1 equiv), diisobutylaluminium hydride (1.0 M) in hexane (2.94 mL, 2 equiv). R_f(EtOAc/n-hexane 2:8) = 2.8. **^{1}HNMR** (400 MHz, $CDCl_3$) δ 7.49 – 7.32 (m, 2H), 7.01 (t, *J* = 8.7 Hz, 2H), 6.14 (dt, *J* = 10.9, 6.4 Hz, 1H), 5.78 (d, *J* = 10.9 Hz, 1H), 4.48 (dd, *J* = 6.4, 1.5 Hz, 2H), 2.00 (br s, 1H). **^{13}C NMR** (101 MHz,

$CDCl_3$) δ 162.6 (d, J_{CF} = 249.9 Hz), 141.4, 133.3 (d, J_{CF} = 8.4 Hz), 119.1 (d, J_{CF} = 3.6 Hz), 115.7 (d, J_{CF} = 22.1 Hz), 110.4, 94.1, 84.7 (d, J_{CF} = 1.5 Hz), 61.0. **IR** (ν_{max}/cm^{-1}) 2967 (w), 2839 (w), 1067 (m), 1520 (s), 1373 (s), 1298 (s), 1114 (s), 1032 (s), 852 (s), 539 (s). **HRMS** (ESI-OTF) *m/z* calcd for $C_{11}H_{10}OF$ $[M + H]^+$ 177.0710 found 177.0709.

(*Z*)-5-Phenylpent-2-en-4-yn-1-ol (S3c).

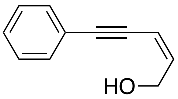

The title compound was prepared using general synthesis procedure for **S3a** using (*Z*)-5-phenylpent-2-en-4-ynoate (100 mg, 0.54 mmol), DIBAL-H (1.09 mL, 1.09 mmol), eluted as pale-yellow liquid (85.5 mg, 98%). **^{1}H NMR** (500 MHz, $CDCl_3$) *δ* 7.49-7.40 (m, 2H), 7.32 (m, 3H), 6.14 (dt, *J* = 10.9, 6.5 Hz, 1H), 5.81 (dt, *J* = 10.9, 1.5 Hz, 1H), 4.50 (dd, *J* = 6.4, 1.5 Hz, 2H), 2.30 (brs, 1H). **^{13}C NMR** (126 MHz, $CDCl_3$) δ 141.2, 131.4, 128.4, 128.3, 122.9, 110.6, 95.2, 84.9, 61.1. NMR data matches literature values.[5]

(*Z*)-5-Phenylpent-2-en-4-ynal-2-*d* (S4a)

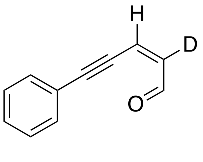

To a stirred solution of **S3a** (300 mg, 1.88 mmol, 1 equiv) in dry DCM (9 mL) was added MnO_2 (2.45 g, 28.2 mmol, 15 equiv). After 4.5 h the mixture was filtered through a pad of silica gel and crude product was purified by flash column chromatography (ether:pentane 1:9) to give the title product as colorless oil (201 mg, 68%), R*f* (EtOAc:*n*-hexane 1:9) = 0.76. **^{1}H NMR** (500 MHz, $CDCl_3$) δ 10.28 (s, 1H, C*H*O), 7.51 – 7.49 (m, 2H, 2ArC*H*), 7.41 – 7.35 (m, 3H, 3ArC*H*), 6.87 – 6.85 (m, 1H, =C*H*). **^{13}C NMR** (126 MHz, $CDCl_3$) δ 191.9, 136.9 (t, J_{CD} = 25.4 Hz) 131.9, 129.8, 128.6, 128.6, 121.7, 101.4, 84.0. **IR** (ν_{max}/cm^{-1}) 3058 (w), 2917 (w),

2192 (w), 1670 (s), 1489 (m), 1078 (m), 916 (m), 754 (s), 687 (s). **HRMS** (ESI-OTF) *m/z* calcd for $C_{11}H_8DO$ [M +H]$^+$ 158.0716 found 158.0722.

(*Z*)-5-(4-Fluorophenyl)pent-2-en-4-ynal (S4b)

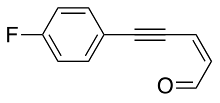

The title compound was obtained as a colorless liquid (260 mg, 99%) after flash column chromatography (ether:pentane 1:9) using the synthesis procedure for **S4a** and (*Z*)-5-(4-fluorophenyl)pent-2-en-4-yn-1-ol (265 mg, 1.5 mmol, 1 equiv), MnO_2 (1.963 g, 22.58 mmol, 15 equiv) and dry CH_2Cl_2 (7.5 mL), R_f (EtOAc:*n*-hexane 1:9) = 0.78. **^{1}H NMR** (400 MHz, $CDCl_3$) δ 10.26 (d, *J* = 8.2 Hz, 1H, C*H*O), 7.63 – 7.38 (m, 2H, 2ArC*H*), 7.15 – 6.94 (m, 2H, 2ArC*H*), 6.85 (d, *J* = 10.8 Hz, 1H, =C*H*), 6.33 (dd, *J* = 10.8, 8.2 Hz, 1H, =C*H*). **^{13}C NMR** (101 MHz, $CDCl_3$) δ 191.7, 163.3 (d, *J* = 252.4 Hz), 137.3, 134.0 (d, J_{CF} = 8.7 Hz), 128.5, 117.8 (d, J_{CF} = 3.6 Hz), 116.0 (d, J_{CF} = 22.3 Hz), 100.2, 83.8 (d, J_{CF} = 1.7 Hz). **IR** (ν_{max}/cm^{-1}) 3358 (w), 2926 (w), 1893 (m), 1661 (s), 1598 (s), 1504 (s), 1295 (s), 1221 (s), 1122 (s), 834 (s), 733 (s), 598 (s). **HRMS (**ESI-OTF) *m/z* calcd for $C_{11}H_8FO$ [M + H]$^+$ 175.0553 found 175.0553.

(*Z*)-5-(4-Methoxyphenyl)pent-2-en-4-ynal (S4c)

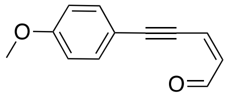

The title compound was prepared as a colorless liquid (211 mg, 71%), using the general synthesis procedure for **S4a** using (*Z*)-5-(4-methoxyphenyl)pent-2-en-4-yn-1-ol (300 mg, 1.59 mmol), MnO_2 (2.08 g, 23.9 mmol)**. ^{1}H NMR** (400 MHz, $CDCl_3$) δ 10.26 (d, *J* = 8.3 Hz, 1H), 7.45 (d, *J* = 8.7 Hz, 2H), 6.89 (d, *J* = 8.7 Hz, 2H), 6.85 (d, *J* = 10.8 Hz, 1H), 6.28 (dd, *J* = 10.8, 2.0 Hz, 1H), 3.84 (s, 3H). **^{13}C NMR** (101 MHz, $CDCl_3$) δ 192.0, 160.8, 136.3, 133.7, 129.2, 114.3, 113.7, 102.1, 83.4, 55.4. NMR data matches the literature value.[6]

(*Z*)-5-(Trimethylsilyl)pent-2-en-4-ynal (S4d)

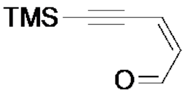

This compound was prepared as a colourless liquid (207 mg, 60%), using the general synthesis procedure for **S4a** using (*Z*)-5-(trimethylsilyl)pent-2-en-4-ynal (350 mg, 2.27 mmol), MnO_2 (2.96 g, 34.09 mmol). **^{1}H NMR** (400 MHz, $CDCl_3$) δ 10.16 (d, *J* = 8.3 Hz, 1H), 6.63 (d, *J* = 10.9 Hz, 1H), 6.29 (dd, *J* = 11.0, 8.2 Hz, 1H), 0.24 (s, 9H). NMR data matches literature values.[7]

(*Z*)-5-Phenylpent-2-en-4-ynal (S4e)

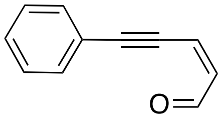

This compound was prepared using the general synthesis procedure for **S4a** using **S3c** (0.80 g, 5.0 mmol), MnO_2 (6.51 g, 75 mmol)**.** **^{1}H NMR** (500 MHz, $CDCl_3$) δ 10.28 (d, *J* = 8.2 Hz, 1H), 7.56 – 7.43 (m, 2H), 7.41 – 7.25 (m, 3H), 6.87 (dd, *J* = 10.8, 1.2 Hz, 1H), 6.33 (ddt, *J* = 10.8, 8.2, 0.6 Hz, 1H). **^{13}C NMR** (126 MHz, $CDCl_3$) δ 193.0, 139.1, 137.3, 132.5, 132.0, 129.8, 128.6, 121.8, 104.2, 86.0. NMR data matches the literature values.[8]

(*Z*)-6-Phenylhex-3-en-5-yn-3-*d*-2-ol (S5a)

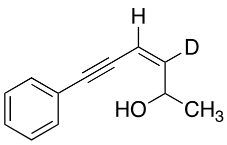

In an oven dried Schlenk flask **S4a** (180 mg, 1.14 mmol, 1 equiv) was dissolved in dry THF (2.3 mL) and the solution was cooled to −5 °C. A solution of CH_3MgCl (3.0 M) in THF (1.14 mL, 3.43 mmol, 3 equiv) was added dropwise over 2 min. After 5 min, the mixture was warmed

to room temperature and quenched with 1 M aqueous HCl (1 mL). The reaction mixture was extracted with EtOAc (3 × 1.5 mL) and the combined extracts dried (Na_2SO_4) and concentrated under vacuo the resulting oil was purified by flash column chromatography (EtOAc:*n*-hexane 2:8) to give **S5a** as colorless oil (186 mg, 93%), R_f (EtOAc/*n*-hexane 2:8) = 0.25. **^{1}H NMR** (500 MHz, $CDCl_3$) δ 7.46 – 7.40 (m, 2H, 2ArC*H*), 7.35 – 7.28 (m, 3H, 3ArC*H*), 5.99 (dd, *J* = 10.9, 8.1 Hz, 0.08H, =C*H*), 5.53 (dd, *J* = 10.9, 8.1 Hz, 0.08H, =C*H*) 5.73 (brs, 1H, =C*H*), 4.95 – 4.91 (q, *J* = 6.8 Hz, 1H, C*H*(CH_3)), 2.01 (brs, 1H, O*H*), 1.35 (d, *J* = 6.4 Hz, 3H, CH_3(CH)). **^{13}C NMR** (126 MHz, $CDCl_3$) δ 145.9 (t, J_{CD} = 24.3 Hz), 131.4, 128.4, 128.3, 123.0, 109.0, 94.9, 85.0, 66.3, 22.5. **IR** (ν_{max}/cm^{-1}) 3324 (w), 2970 (m), 1603 (s), 1571 (s), 1488 (s), 1367 (s), 1115 (s), 1057 (s), 916 (s), 886 (s), 686 (s), 524 (s). **HRMS** (ESI-OTF) *m/z* calcd for $C_{12}H_{12}DO$ $[M + H]^+$ 174.1029, found 174.1034.

(*Z*)-6-(4-Fluorophenyl)hex-3-en-5-yn-2-ol (S5b)

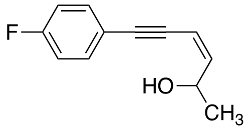

The title compound was obtained as colorless liquid (166 mg, 59%) after flash column chromatography (EtOAc:*n*-hexane 2:8) following the synthesis procedure for **S5a** using **S4b** (260 mg, 1.49 mmol, 1 equiv) dry THF (2.9 mL) and CH_3MgCl (3.0 M) in THF (1.49 mL, 1.48 mmol, 3 equiv), R_f (EtOAc:*n*-hexane 2:8) = 0.27. **^{1}H NMR** (500 MHz, $CDCl_3$) δ 7.41 (dd, *J* = 8.8, 5.4 Hz, 1H, 2ArC*H*), 7.02 (t, *J* = 8.7 Hz, 2H, 2CH_{Ar}), 5.99 (dd, *J* = 10.9, 8.1 Hz, 1H, =C*H*), 5.71 (dd, *J* = 10.9, 1.0 Hz, 1H, =C*H*), 5.00 – 4.80 (m, 1H, C*H*(CH_3)), 1.35 (d, *J* = 6.4 Hz, 3H, CH_3(CH)). **^{13}C NMR** (126 MHz, $CDCl_3$) δ162.5 (d, J_{CF} = 249.9 Hz), 146.3, 133.3 (d, J_{CF} = 8.3 Hz), 119.1 (d, J_{CF} = 3.6 Hz), 115.7 (d, J_{CF} = 22.1 Hz), 109.0, 93.9, 84.7 (d, J_{CF} = 1.6 Hz), 66.4, 22.6. **IR** (ν_{max}/cm^{-1}) 3349 (s), 2971 (m), 2885 (m), 1599 (s), 1506 (s), 1406 (s), 1368 (s),

1232 (s), 1157 (s), 1060 (s), 950 (s), 815 (s), 611 (s), 528 (s). **HRMS (**ESI-OTF) *m/z* calcd for $C_{12}H_{11}FONa$ [M + Na]$^{+}$ 213.0686 found 213.0688.

(*Z*)-6-(4-Methoxyphenyl)hex-3-en-5-yn-2-ol (S5c)

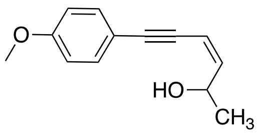

The title compound was obtained as a colourless liquid (196 mg, 90%) after flash column chromatography (EtOAc:*n*-hexane 1.5:8.5) following the synthesis procedure for **S5a** using **S4c** (200 mg, 1.07 mmol, 1 equiv) in dry THF (2.1 mL) and CH_3MgCl (3.0 M) in THF (1.08 mL, 3 equiv), R_f (EtOAc:*n*-hexane 2:8) = 0.29. **^{1}H NMR** (500 MHz, $CDCl_3$) δ 7.37 (d, *J* = 8.8 Hz, 2H, 2ArC*H*), 6.85 (d, *J* = 8.8 Hz, 2H, 2ArC*H*), 5.94 (dd, *J* = 10.8, 8.1 Hz, 1H, =C*H*), 5.71 (dd, *J* = 10.9, 1.0 Hz, 1H, =C*H*), 5.00 – 4.82 (m, 1H, C*H*(CH_3)), 3.81 (s, 3H, OC*H₃*), 1.35 (d, *J* = 6.4 Hz, 3H, C*H*$_3$(CH)), 1.85 (brs, 1H, OH). **^{13}C NMR** (101 MHz, $CDCl_3$) δ 159.7, 145.4, 132.9, 115.1, 114.0, 109.4, 95.1, 83.8, 66.4, 55.3, 22.6. **IR** (ν_{max}/cm^{-1}) 3333 (w), 2943 (m), 2834 (m), 1603 (m), 1531 (s), 1509 (m), 1454 (m), 1350 (m), 1249 (m), 1171 (s), 1108 (m), 1025 (s). 832 (m), 606 (m). **HRMS** (ESI-OTF) *m/z* calcd for $C_{13}H_{15}O_2$ [M + H]$^{+}$ 203.1072, found 203.1082.

(*Z*)-6-(Trimethylsilyl)hex-3-en-5-yn-2-ol (S5d)

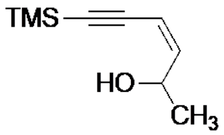

The title compound was obtained as a colourless liquid (325 mg, 59%) after flash column chromatography (ether:pentane 1.5:8.5) following the synthesis procedure for **S5a** using **(S4d)** (500 mg, 3.28 mmol, 1 equiv) in dry THF (6.5 mL) and CH_3MgCl (3.0 M) in THF (3.28 mL,

9.8 mmol, 3 equiv). R_f(ether:pentane 2:8) = 0.32. **^{1}H NMR** (500 MHz, $CDCl_3$) δ 5.96 (dd, *J* = 11.1, 7.9 Hz, 1H, =C*H*), 5.51 (dd, *J* = 11.1, 1.2 Hz, 1H, =C*H*), 4.83 (ddd, *J* = 7.9, 6.4, 1.2 Hz, 1H, (CH_3)C*H*), 1.30 (d, *J* = 6.4 Hz, 3H, (CH)CH_3), 0.19 (s, 9H, TMS). **^{13}C NMR** (126 MHz, $CDCl_3$) δ 147.8, 109.3, 100.9, 100.7, 66.5, 22.5, 0.0. **IR** (ν_{max}/cm^{-1}) 3326 (w), 2962 (m), 2150 (m), 1249 (s), 1059 (s), 956 (s), 836 (s), 758 (s), 697 (s), 632 (m). **HRMS** (ESI-OTF) *m/z* calcd for $C_9H_{16}OSiNa$ $[M + Na]^+$ 191.0863, found 191.0862.

(*Z*)-Undec-3-en-5-yn-2-ol (S5e)

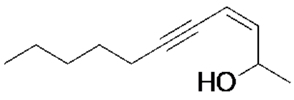

The title compound was obtained as pale-yellow liquid (193 mg, 87%) after flash column chromatography using the general synthesis procedure for **S5a** using (*Z*)-dec-2-en-4-ynal (200 mg, 1.21 mmol), CH_3MgCl (3.0 M) in THF (1.21 mL, 3.65 mmol). **^{1}H NMR** (500 MHz, $CDCl_3$) δ 5.84 (dd, *J* = 10.8, 7.9 Hz, 1H), 5.49 (dtd, *J* = 10.8, 2.2, 1.1 Hz, 1H), 4.81 (dtd, *J* = 7.6, 6.4, 1.2 Hz, 1H), 2.33 (td, *J* = 7.2, 2.3 Hz, 2H), 1.79 (brs, 1H) 1.54 (q, *J* = 7.3 Hz, 2H), 1.35 (m, 4H), 1.30 (d, *J* = 6.4 Hz, 3H), 0.91 (t, *J* = 7.2 Hz, 3H). **^{13}C NMR** (126 MHz, $CDCl_3$) δ 144.9, 109.7, 96.6, 76.2, 66.3, 31.0, 28.3, 22.5, 22.1, 19.4, 13.9. NMR data matches literature values.[9]

(*Z*)-8-Phenyloct-5-en-7-yn-4-ol (S5f)

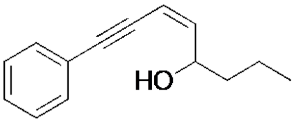

The title compound was obtained as a pale yellow liquid (24 mg, 63%) after flash column chromatography (EtOAc:*n*-hexane 2:8) following the synthesis procedure for **S5a** using **S4e** (30 mg, 0.19 mmol, 1 equiv) and propylmagnesium bromide (2.0 M) in diethyl ether (0.28 mL,

3 equiv). R_f (EtOAc:*n*-hexane 2:8) = 0.31. ^{1}**H NMR** (400 MHz, $CDCl_3$) δ 7.46 – 7.40 (m, 2H, 2ArC*H*), 7.34 – 7.29 (m, 3H, 3ArC*H*), 5.95 (dd, *J* = 10.9, 8.3 Hz, 1H, =C*H*), 5.77 (dd, *J* = 10.9, 1.0 Hz, 1H, =C*H*), 4.79 (q, *J* = 6.5 Hz, 1H, C*H*(OH)), 1.83 (brs, 1H, OH), 1.74-1.52 (m, 2H, CH_2), 1.45 (m, 2H, CH_2), 0.97 (t, *J* = 7.3 Hz, 3H, CH_3). ^{13}**C NMR** (101 MHz, $CDCl_3$) δ 145.3, 131.4, 128.4, 128.3, 123.0, 109.8, 94.7, 85.3, 70.0, 38.8, 18.4, 14.0. **IR** (ν_{max}/cm^{-1}) 3339 (w), 2958 (m), 2930 (m), 2871 (m), 1724 (m), 1596 (m), 1489 (m), 1374 (m), 1243 (m), 1121 (m), 1006 (m), 913 (s), 753 (s), 689 (s). **HRMS** (ESI-OTF) *m/z* calcd for $C_{14}H_{17}O$ $[M + H]^+$ 201.1279 found 201.1278.

(*Z*)-4-Methyl-6-phenylhex-3-en-5-yn-2-ol (S5g)

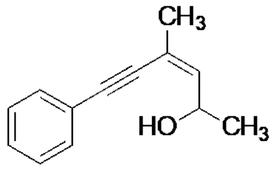

The title compound was obtained as a colorless liquid (386 mg, 93%) after flash column chromatography using the general synthesis procedure for **S5a** using (*Z*)-3-methyl-5-phenylpent-2-en-4-ynal (377 mg, 2.21 mmol), CH_3MgCl (3.0 M) in THF (2.21 mL, 6.65 mmol). ^{1}**H NMR** (400 MHz, $CDCl_3$) δ 7.50 – 7.39 (m, 2H), 7.35 – 7.28 (m, 3H), 5.75 (dd, *J* = 8.3, 1.5 Hz, 1H), 4.86 (dq, *J* = 8.4, 6.3 Hz, 1H), 2.12 (s, 1H), 1.94 (d, *J* = 1.6 Hz, 3H), 1.31 (d, *J* = 6.4 Hz, 3H, CH_3CH). ^{13}**C NMR** (101 MHz, $CDCl_3$) δ 141.0, 131.5, 131.4, 128.3, 123.1, 119.0, 94.1, 87.4, 66.9, 23.0, 22.6. NMR data matches literature values.[5]

(*Z*)-6-Phenylhex-3-en-5-yn-2-ol (S5h)

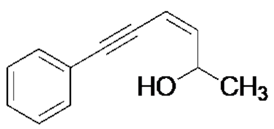

The title compound was obtained as a pale-yellow liquid (143 mg, 85%) after flash column chromatography using the general synthesis procedure for **S5a** using (*Z*)-5-phenylpent-2-en-4-ynal (180 mg, 0.97 mmol, CH_3MgCl (3.0 M) in THF (0.97 mL, 2.93 mmol). **^{1}H NMR** (500 MHz, $CDCl_3$) δ 7.48 – 7.39 (m, 2H), 7.36 – 7.30 (m, 3H), 5.99 (dd, *J* = 10.9, 8.1 Hz, 1H), 5.73 (dd, *J* = 10.9, 1.1 Hz, 1H), 4.98 – 4.92 (m, 1H), 1.87 (s, 1H), 1.36 (d, *J* = 6.4 Hz, 3H). **^{13}C NMR** (126 MHz, $CDCl_3$) δ 146.2, 131.4, 128.4, 128.3, 123.0, 109.2, 95.0, 84.9, 66.4, 22.5. NMR data matches literature values.[10]

(*Z*)-2-Methyl-7-phenylhept-4-en-6-yn-3-ol (S5i)

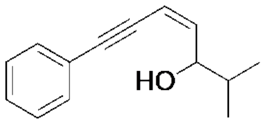

The title compound was obtained as pale yellow liquid (250 mg, 77%) after flash column chromatography (EtOAc:*n*-hexane 1.5:8.5) following the synthesis procedure for **S5a** using **S4e** (260 mg, 1.66 mmol, 1 equiv) and isopropylmagnesium chloride (2.0 M) in diethyl ether (2.5 mL, 3 equiv). R_f(EtOAc:*n*-hexane 2:8) = 0.47. **^{1}H NMR** (400 MHz, $CDCl_3$) δ 7.47 – 7.39 (m, 2H, 2ArC*H*), 7.34 – 7.29 (m, 3H, 3ArC*H*), 5.97 (dd, *J* = 11.0, 8.5 Hz, 1H, =C*H*(CH)OH), 5.82 (dd, *J* = 11.0, 0.9 Hz, 1H, =C*H*(alkyne)), 4.51 (ddd, *J* = 9.0, 6.1, 3.1 Hz, 1H, C*H*(OH)), 1.85 – 1.81 (m, 1H, C*H*(CH_3)), 1.62 – 1.58 (m, 1H, O*H*), 1.02 (d, *J* = 6.8 Hz, 3H, C*H*$_3$), 0.96 (d, *J* = 6.9 Hz, 3H, C*H*$_3$). **^{13}C NMR** (101 MHz, $CDCl_3$) δ 143.7, 131.4, 128.4, 128.3, 110.8, 94.6, 85.5, 75.1, 34.0, 18.1, 18.0. **IR** (ν_{max}/cm^{-1}) 3236 (w), 2969 (w) 1596 (m), 1488 (w), 1262 (m), 1021 (m), 752 (m), 686 (s), 524 (s). **HRMS** (ESI-OTF) *m/z* calcd for $C_{14}H_{16}ONa$ [M + Na]$^+$ 223.1099 found 223.1102.

(*Z*)-1,5-Diphenylpent-2-en-4-yn-1-ol (S5j)

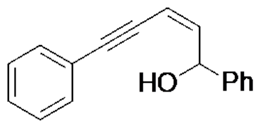

This title compound was obtained after flash column chromatography as a pale yellow oil (380 mg, 85%) using the general synthesis procedure for **S5a** using **S4e** (150 mg, 0.96 mmol) PhMgBr 1.0 M in THF (2.8 mL, 2.88 mmol). **^{1}H NMR** (500 MHz, $CDCl_3$) δ 7.51 – 7.43 (m, 4H), 7.38 – 7.31 (m, 5H), 6.14 (dd, *J* = 10.7, 8.6 Hz, 1H), 5.92 (d, *J* = 8.6 Hz, 1H), 5.83 (dd, *J* = 10.7, 0.9 Hz, 1H), 1.63 (s, 1H).. **^{13}C NMR** (126 MHz, $CDCl_3$) δ 144.0, 142.3, 131.5, 128.6, 128.5, 128.4, 128.2, 127.7, 125.8, 122.9, 110.0, 9.12, 85.2, 72.1. NMR data matches literature values.[11]

(*E*)-6-Phenylhex-3-en-5-yn-2-ol (S5k)

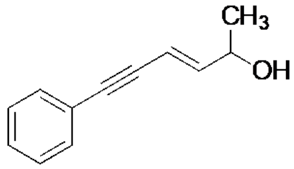

The title compound was obtained after flash column chromatography as a pale-yellow liquid (128 mg, 83%) using the general synthesis procedure for **S5a** from (*E*)-5-phenylpent-2-en-4-ynal (140 mg, 0.76 mmol, CH_3MgCl (3.0 M) in THF (0.76 mL, 2.28 mmol). **^{1}H NMR** (500 MHz, $CDCl_3$) δ 7.49 – 7.39 (m, 2H), 7.32-7.25 (m, 3H), 6.26 (dd, *J* = 15.9, 5.8 Hz, 1H), 5.92 (dd, *J* = 15.9, 1.5 Hz, 1H), 4.40 (qd, *J* = 6.5, 1.5 Hz, 1H), 2.35 (brs, 1H), 1.32 (d, *J* = 6.5 Hz, 3H). NMR data matches literature values.[10]

3. General scheme for the synthesis of tethered-enediynes (1a-u).

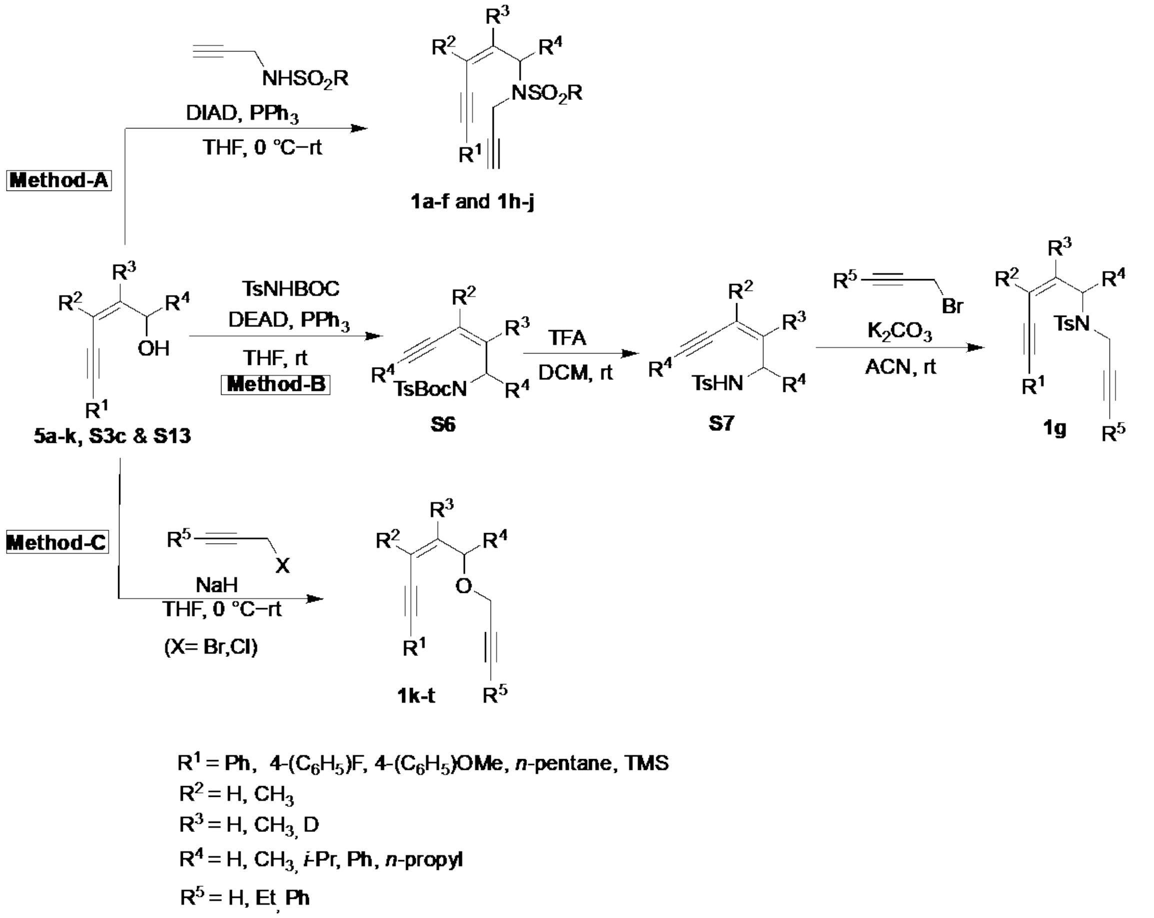

Method A: General Synthesis procedure of N-tethered enediynes (1a-j)

The N-tethered enediynes (**1a-j)** were prepared using the sulfonamide and alcohol (**S2c, S3c, S5a-e** and **S5h**) coupling procedure reported in the literature.[12,13] In an oven dried Schlenk flask, a solution of alcohols (**S2c, S3c, S5a-e** and **S5h)** (1 equiv.) in anhydrous THF (0.2 M) was added Ph_3P (1.3 equiv) and sulfonamide (1.15 equiv) at 0 °C. A solution of diisopropylazodicarboxylate (DIAD) (1.3 equiv) was added dropwise and allowed to warm to room temperature and stirred for 3 h. The reaction mixture was quenched with aqueous $NaHCO_3$ (1 mL) and extracted with EtOAc. The combined extracts were washed with brine

and dried (Na_2SO_4). The crude product was purified by flash column chromatography on silica gel (EtOAc:*n*-hexane) to obtain the pure enediynes (**1a-j**).

(*Z*)-4-Methyl-*N*-(6-phenylhex-3-en-5-yn-2-yl)-*N*-(prop-2-yn-1-yl)benzenesulfonamide (1a)

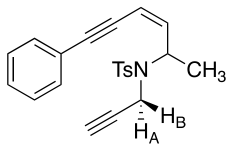

The title compound was obtained pale yellow oil (89.4 mg, 85%) after flash column chromatography (EtOAc:*n*-hexane 1.5:8.5) following the general synthesis **Method-A** using **S5h** (50 mg, 0.29 mmol, 1 equiv), 4-methyl-*N*-(prop-2-yn-1-yl)benzenesulfonamide **S10** (70 mg, 0.38 mmol, 1.15 equiv), PPh_3 (102 mg, 0.38 mmol, 1.3 equiv) and DIAD (0.07 mL, 0.38 mmol, 1.3 equiv) in dry THF (3 mL). R_f(EtOAc:*n*-hexane 2:8) = 0.57. **^{1}H NMR** (500 MHz, $CDCl_3$) δ 7.81 (d, *J* = 8.3 Hz, 2H, 2ArC*H*), 7.49 – 7.46 (m, 1H, ArC*H*), 7.36 – 7.31 (m, 3H, 3ArC*H*), 7.19 (d, *J* = 7.8 Hz, 1H, ArC*H*), 6.09 (dd, *J* = 10.8, 9.0 Hz, 1H, =C*H*), 5.66 (dd, *J* = 10.8, 0.9 Hz, 1H, =C*H*), 5.21 – 5.07 (m, 1H, C*H*(CH_3)), 4.26 (dd, *J* = 18.5, 2.5 Hz, 1H, CH_A(N)), 4.11 (dd, *J* = 18.5, 2.5 Hz, 1H, CH_B(N)), 2.36 (s, 3H, CH_3(Ts)), 2.18 (t, *J* = 2.5 Hz, 1H, C*H* alkyne), 1.43 (d, *J* = 7.0 Hz, 3H, CH_3(CH)). **^{13}C NMR** (126 MHz, $CDCl_3$) δ 143.2, 140.7, 137.5, 131.6, 129.3, 128.5, 128.3, 127.6, 122.9, 110.6, 95.7, 84.8, 79.7, 72.9, 53.4, 33.4, 21.4, 19.5. **IR** (ν_{max}/cm^{-1}) 3287 (w), 2510 (m), 2160 (m), 1597 (s), 1434 (s), 1152 (s), 1078 (s), 901 (s), 746 (s), 662 (s), 573 (s). **HRMS** (ESI-OTF) *m/z* calcd for $C_{22}H_{21}NO_2SNa$ $[M + Na]^+$ 386.1191, found 386.1183.

(*Z*)-4-Methyl-*N*-(5-phenylpent-2-en-4-yn-1-yl)-*N*-(prop-2-yn-1-yl)benzenesulfonamide (1b)

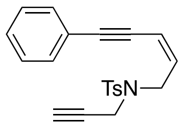

The title compound was obtained as a colorless oil (185 mg, 84%) after flash column chromatography (EtOAc/hexane 1.5:8.5) following the general procedure **Method-A** using 4-methyl-*N*-(prop-2-yn-1-yl)benzenesulfonamide **S10** (152 mg, 0.72 mmol), **S3c** (100 mg, 0.63 mmol), PPh_3 (220 mg, 0.84 mmol) and DIAD(0.16 mL, 0.84 mmol) in dry THF (3.5 mL). R_f (Et_2O/pentane 1.5:8.5) = 0.45. **^{1}H NMR** (500 MHz, $CDCl_3$) δ 7.78 (d, *J* = 8.3 Hz, 2H, C*H*Ar), 7.39-7.27 (m, 2H, C*H*Ar), 7.34 – 7.24 (m, 5H, C*H*Ar), 5.96 – 5.85 (m, 2H, (alkyne)C*H*$_2$), 4.19 (d, *J* = 5.8 Hz, 2H, =C*H*$_2$), 4.13 (d, *J* = 2.5 Hz, 2H, (CH=CH)C*H*$_2$NTs), 2.43 (s, 3H, TsC*H*$_3$), 2.01 (t, *J* = 2.5 Hz, 1H, alkyneC*H*). **^{13}C NMR** (126 MHz, $CDCl_3$) δ 143.6, 136.2, 135.7, 131.4, 129.5, 129.4, 128.5, 128.3, 127.8, 122.8, 113.9, 95.6, 84.6, 73.6, 45.8, 36.6, 21.5. **IR** (ν_{max}/cm^{-1}) 3287 (w), 2923 (m), 2160 (m), 1597 (m), 1490 (m), 1441 (m), 1345 (s), 1157 (s), 1094 (s), 1040 (s), 896 (m) 817 (m), 757 (m), 690 (m), 541 (s). **HRMS** (ESI-OTF) *m/z* calcd for $C_{21}H_{19}NO_2SNa$ [M + Na]$^+$ 372.1034 found 372.1038.

(*Z*)-*N*-(6-(4-Fluorophenyl)hex-3-en-5-yn-2-yl)-4-methyl-*N*-(prop-2-yn-1-yl)benzenesulfonamide (1c)

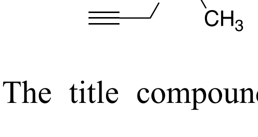

The title compound was obtained as a colorless oil (53 mg, 53%) after flash column chromatography (EtOAc:*n*-hexane 1.2:8.8) following the general synthesis procedure **Method-A** using 4-methyl-*N*-(prop-2-yn-1-yl)benzenesulfonamide (63.3mg, 0.3 mmol), (*Z*)-6-(4-fluorophenyl)hex-3-en-5-yn-2-ol **S5b** (50 mg, 0.26 mmol), PPh_3 (70.7 mg, 0.3 mmol) and

DIAD (0.08 mL, 0.3 mmol) in dry THF (2 mL). R_f (EtOAc:*n*-hexane 2:8) = 0.79. **^{1}H NMR** (500 MHz, $CDCl_3$) δ 7.80 (d, *J* = 8.4 Hz, 2H, 2ArC*H*), 7.52 – 7.41 (m, 2H, 2ArC*H*), 7.23 – 7.17 (m, 2H, 2ArC*H*), 7.04 (t, *J* = 8.7 Hz, 1H, ArC*H*), 6.08 (dd, *J* = 10.8, 9.0 Hz, 1H, =C*H*), 5.64 (dd, *J* = 10.8, 0.9 Hz, 1H, =C*H*), 5.17 (dtd, *J* = 8.9, 7.0, 6.1 Hz, 1H, C*H*(CH_3)), 4.25 (dd, *J* = 18.6, 2.5 Hz, 1H, CH_A(N)), 4.10 (dd, *J* = 18.6, 2.5 Hz, 1H, CH_B(N)), 2.37 (s, 3H, CH_3(Ts)), 2.19 (t, *J* = 2.5 Hz, 1H, C*H*-alkyne), 1.41 (d, *J* = 7.0 Hz, 3H, CH_3(CH)). **^{13}C NMR** (126 MHz, $CDCl_3$) δ162.3 (d, J_{CF} = 250.0 Hz), 143.2, 140.7, 137.4, 133.5 (d, J_{CF} = 8.4 Hz), 129.3, 127.6, 119.0 (d, J_{CF} = 3.5 Hz), 115.7 (d, J_{CF} = 22.1 Hz), 110.6, 94.7, 84.5 (d, J_{CF} = 1.5 Hz), 79.6, 72.9, 53.3, 33.2, 21.5, 19.5. **IR** (ν_{max}/cm^{-1}) 3293 (w), 2946 (w), 2834 (w), 1652 (m), 1531(s), 1506 (s), 1351 (s), 1231 (s), 1166 (s), 1095 (m), 838 (m), 582 (m). **HRMS** (ESI-OTF) *m/z* calcd for $C_{22}H_{21}NO_2FS$ $[M + H]^+$ 382.1277, found 382.1266.

(*Z*)-*N*-(6-(4-Methoxyphenyl)hex-3-en-5-yn-2-yl)-4-methyl-*N*-(prop-2-yn-1-yl)benzenesulfonamide (1d) and **(*E*)-*N*-(6-(4-Methoxyphenyl)hex-3-en-5-yn-2-yl)-4-methyl-*N*-(prop-2-yn-1-yl)benzenesulfonamide (1d')**.

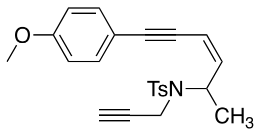

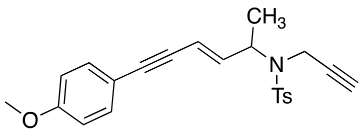

The title compounds were obtained as a colorless oil (120 mg, 80%) after flash column chromatography (EtOAc:*n*-hexane 1.5:8.5) (**1d and 1d'**= 1.2:1) following the general procedure **method-A** using 4-methyl-*N*-(prop-2-yn-1-yl)benzenesulfonamide (119 mg, 0.56 mmol), (*Z*)-6-(4-methoxyphenyl)hex-3-en-5-yn-2-ol **S5c** (100 mg, 0.49 mmol), PPh_3 (172 mg, 0.65 mmol) and diisopropylazodicarboxylate (0.12 mL, 0.6 mmol) in dry THF (3.8 mL). R_f (EtOAc/hexane 2:8) = 0.71. **^{1}H NMR** (400 MHz, $CDCl_3$) (**1d**:**1d'**= 1.2:1). δ 7.83 (d, *J* = 8.3 Hz, 2H, 2ArC*H*, *E*-isomer), 7.80 (d, *J* = 8.4 Hz, 2H, 2ArC*H*, *Z*-isomer), 7.41 (d, *J* = 8.8 Hz, 2H, 2ArC*H*, *Z*-isomer), 7.27 – 7.21 (m, 2H, 2ArC*H*, *Z*-isomer), 7.21 – 7.15 (m, 2H, 2ArC*H*,

E-isomer), 7.10 (d, *J* = 8.8 Hz, 2H, 2ArC*H*, *E*-isomer), 6.86 (d, *J* = 8.8 Hz, 2H, 2ArC*H*, *E*-isomer), 6.78 (d, *J* = 8.9 Hz, 2H, 2ArC*H*, *Z*-isomer), 6.13 – 6.06 (m, 1H, =C*H*, *E*-isomer), 6.02 (dd, *J* = 10.8, 8.9 Hz, 1H, =C*H*, *Z*-isomer), 5.64 (dd, *J* = 10.8, 0.9 Hz, 1H, =C*H*, *Z*-isomer), 5.56 (dd, *J* = 14.8, 1.6 Hz, 1H, =C*H*, *E*-isomer), 5.52 (dt, *J* = 4.8, 1.5 Hz, 1H, (CH_3)C*H*, *Z*-isomer), 5.20 – 5.10 (m, 1H, (CH_3)C*H*, *E*-isomer), 4.24 (dd, *J* = 18.6, 2.5 Hz, 1H, CH_A(N) *Z*-isomer), 4.10 (dd, *J* = 18.6, 2.5 Hz, 1H, CH_B(NTs) *Z*-isomer), 4.05 (t, *J* = 2.3 Hz, 2H, (CH_ACH_B(N), *E*-isomer), 3.82 (s, 3H, OCH_3, *E*-isomer), 3.79 (s, 3H, OCH_3, *Z*-isomer), 2.36 (s, 3H, CH_3(Ts) *E*-isomer), 2.36 (s, 3H, CH_3(Ts) *Z*-isomer), 2.18 (t, *J* = 2.4 Hz, 1H, C*H*(alkyne) *Z*-isomer), 2.12 (t, *J* = 2.5 Hz, 1H, C*H*(alkyne) *E*-isomer), 1.75 (dt, *J* = 6.4, 1.5 Hz, 3H, (CH)CH_3, *E*-isomer), 1.42 (d, *J* = 7.0 Hz, 3H, (CH)CH_3, *Z*-isomer). **^{13}C NMR** (101 MHz, CDCl3) δ 159.8, 159.7, 143.5, 143.1, 139.7, 137.6, 136.4, 133.1, 132.9, 130.9, 129.3, 129.3, 128.0, 126.7, 115.1, 114.3, 114.0, 114.0, 113.8, 111.0, 95.9, 87.7, 83.8, 81.9, 79.8, 79.5, 72.8, 72.2, 55.3, 55.3, 53.5, 52.28, 33.6, 33.4, 21.5, 19.6, 17.4. **IR** (ν_{max}/cm^{-1}) 3287 (w), 2935 (w), 2219 (w), 1601 (m), 1508 (s), 1443 (m), 1333 (m), 1290 (m), 1247 (m), 1158 (s), 1091 (m), 1029 (m), 865 (m), 747 (s), 573 (s), 543 (s). **HRMS** (ESI-OTF) *m/z* calcd for $C_{23}H_{24}NO_3S$ [M + H]$^+$ 394.1477, found 394.1471.

(*Z*)-4-Methyl-*N*-(prop-2-yn-1-yl)-*N*-(undec-3-en-5-yn-2-yl)benzenesulfonamide (1e)

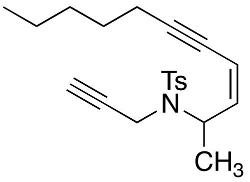

The title compound was obtained as a pale yellow liquid (122 mg, 60%) after flash column chromatography (EtOAc:*n*-hexane 1.5:8.5) following the general procedure **Method-A** using 4-methyl-*N*-(prop-2-yn-1-yl)benzenesulfonamide **S10** (133 mg, 0.63 mmol), (*Z*)-undec-3-en-5-yn-2-ol **S5e** (100 mg, 0.5 mmol), PPh_3 (194 mg, 0.73 mmol) and DIAD (0.14 mL, 0.7 mmol)

in dry THF (4.2 mL). R_f (EtOAc:*n*-hexane 1:9) = 0.51. **^{1}H NMR** 400 MHz, $CDCl_3$) δ 7.82 – 7.75 (m, 2H, C*H*Ar), 7.30 – 7.20 (m, 2H, C*H*Ar), 5.99 – 5.82 (m, 1H, =C*H*), 5.41 (dtd, *J* = 10.8, 2.3, 1.0 Hz, 1H, =C*H*), 5.12 – 4.95 (m, 1H, C*H*CH_3), 4.21 (dd, *J* = 18.6, 2.5 Hz, 1H,CH_A(N)), 4.05 (dd, *J* = 18.6, 2.5 Hz, 1H, CH_B(N)), 2.41 (3H, CH_3Ts), 2.38 – 2.28 (m, 2H, CH_2CH_2), 2.16 (t, *J* = 2.5 Hz, 1H, CH_2CH_2), 1.62 – 1.52 (m, 2H, CH_2CH_2), 1.37 (d, *J* = 7.0 Hz, 3H, CH_3CH), 0.91 (t, *J* = 7.1 Hz, 3H, CH_3CH_2). **^{13}C NMR** (101 MHz, $CDCl_3$) δ 143.1, 139.3, 137.8, 129.2, 127.9, 127.6, 111.1, 97.3, 79.8, 72.6, 53.5, 33.4, 31.1, 28.3, 22.2, 21.5, 19.5, 19.57, 13.9. **IR** (ν_{max}/cm^{-1}) 3279 (w), 2956 (m), 2859 (m), 1598 (m), 1454(s), 1333(s), 1288 (m), 1154 (s), 1035 (s), 906 (s), 813 (s), 739 (m), 544 (s). **HRMS** (ESI-OTF) *m/z* calcd for $C_{21}H_{27}NO_2SNa$ $[M + Na]^+$ 380.1160 found 380.1663.

(*Z*)-4-Methyl-*N*-(prop-2-yn-1-yl)-*N*-(6-(trimethylsilyl)hex-3-en-5-yn-2-yl)benzenesulfonamide (1f) and (*E*)-4-Methyl-*N*-(prop-2-yn-1-yl)-*N*-(6-(trimethylsilyl)hex-3-en-5-yn-2-yl)benzenesulfonamide (1f').

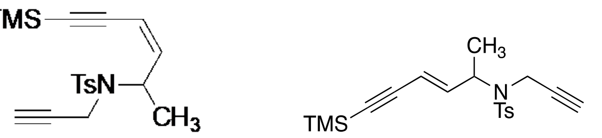

The title product were obtained as a mixture of *Z/E*-isomers **(1.6:1)** as a colorless oil (133 mg, 62%) after flash column chromatography (ether/pentane 1.5:8.5) following the general procedure **Method-A** using 4-methyl-*N*-(prop-2-yn-1-yl)benzenesulfonamide (143 mg, 0.6 mmol), (*Z*)-6-(trimethylsilyl)hex-3-en-5-yn-2-ol **S5d** (100 mg, 0.59 mmol), PPh_3 (176 mg, 0.6 mmol) and diisopropylazodicarboxylate (0.1 mL, 0.3 mmol) in dry THF (4 mL). R_f (Et_2O/pentane 1.5:8.5) = 0.8. **^{1}H NMR** (500 MHz, $CDCl_3$) δ δ 7.79 (dd, *J* = 8.4, 4.9 Hz, 3H, *ArCH*, for each isomer), 7.31 – 7.20 (m, 4H for each isomer), 6.11 – 6.08 (m, 1H, =C*H*, *Z*-isomer), 6.07 – 6.05 (m, 1H, =C*H*, *E*-isomer). 5.59 (dd, *J* = 16.1, 1.9 Hz, 1H, =C*H*, *E*-isomer),

5.44 (dd, J = 10.9, 1.0 Hz, 1H, =C*H, Z*-isomer). 5.05 (ddd, J = 8.7, 7.0, 1.1 Hz, 1H, C*H*(CH_3, *Z*-isomer),), 4.57 (ddd, J = 7.0, 5.3, 1.9 Hz, 1H, C*H*(CH_3, *E*-isomer). 4.24 (dd, J = 18.6, 2.5 Hz, 1H, CH_A(N), *Z*-isomer), 4.16 (dd, J = 18.5, 2.5 Hz, 1H, CH_B(N), *E*-isomer), 4.06 (dd, J = 18.5, 2.5 Hz, 1H, CH_A(N), *Z*-isomer)), 3.88 (dd, J = 18.5, 2.5 Hz, 1H, CH_B(N)), *E*-isomer), δ 2.43 (s, 3H, CH_3Ts, *Z*-isomer), 2.41 (s, 3H, *E*-isomer). δ 2.16 (t, J = 2.5 Hz, 1H, alkyne-C*H, Z*-isomer), 1.61 (dd, J = 2.3, 1.0 Hz, alkyne-C*H*, 1H, *E*-isomer), 1.36 (d, J = 7.0 Hz, 3H, CH_3(CH), *Z*-isomer), 1.28 (d, J = 7.0 Hz, 3H, CH_3(CH), *E*-isomer), 0.22 (s, 9H, TMS, *Z*-isomer), 0.17 (s, 9H, TMS, *E*-isomer). **^{13}C NMR** (126 MHz, $CDCl_3$) δ 143.6, 143.3, 143.0, 142.5, 137.8, 137.7, 129.7, 129.7, 129.4, 127.7, 127.7, 112.5, 110.6, 102.5, 101.7, 100.5, 96.4, 79.9, 79.7, 73.0, 72.9, 54.6, 53.8, 33.8, 32.8, 21.7, 19.3, 17.5. **IR** (ν_{max}/cm^{-1}) 3344 (w), 2970 (s), 2932 (m), 1466 (m), 1379 (m), 1306 (s), 1159 (s), 1106 (s), 950 (s), 816 (s), 659 (m). **HRMS** (ESI-OTF) *m/z* calcd for $C_{19}H_{25}NO_2SSiNa$ [M + Na]$^+$ 382.1268 found 382.1268.

(*Z*)-4-Nitro-*N*-(6-phenylhex-3-en-5-yn-2-yl)-*N*-(prop-2-yn-1-yl)benzenesulfonamide (1h)

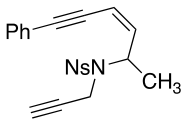

The title compound was obtained as a colorless oil (53 mg, 53%) after flash column chromatography (EtOAc:*n*-hexane 2.:8) following the general procedure **Method-A** using 4-nitro-*N*-(prop-2-yn-1-yl)benzenesulfonamide[13] (106 mg, 0.3 mmol), (*Z*)-6-phenylhex-3-en-5-yn-2-ol **S5h** (50 mg, 0.29 mmol), PPh_3 (78.1 mg, 0.38 mmol) and DIAD (0.07mL, 0.38 mmol) in dry THF (2.2 mL). R_f (EtOAc/hexane 3:7) = 0.69. **^{1}H NMR** (500 MHz, $CDCl_3$) δ 8.21 (d, J = 8.9 Hz, 2H, 2CH_{Ar}), 8.12 (d, J = 8.9 Hz, 2H, 2ArC*H*), 7.52 – 7.45 (m, 2H, 2ArC*H*), 7.40 – 7.32 (m, 3H, 3ArC*H*), 6.04 (dd, J = 10.7, 9.3 Hz, 1H, =C*H*), 5.66 (dd, J = 10.7, 0.8 Hz, 1H, =C*H*), 5.28 – 5.20 (m, 1H, C*H*(CH_3)), 4.39 (dd, J = 18.7, 2.5 Hz, 1H, CH_A(N)), 4.15 (dd, J = 18.7, 2.5 Hz, 1H, CH_B(N)), 2.20 (t, J = 2.5 Hz, 1H, C*H*-alkyne), 1.45 (d, J = 7.0 Hz, 3H,

CH_3(CH)). **^{13}C NMR** (126 MHz, CDCl$_3$) δ 146.2, 139.4, 131.5, 128.9, 128.8, 128.5, 123.8, 122.5, 111.5, 96.4, 84.5, 78.9, 53.7, 33.2, 19.7. **IR** (ν_{max}/cm^{-1}) 3351 (w), 3270 (w), 2971(s), 1646 (m), 1530 (s), 1404 (s), 1309 (s), 1161 (s), 1100 (s), 949 (s), 816 (s), 616 (s). **HRMS** (ESI-OTF) *m/z* calcd for $C_{21}H_{18}N_2O_4SNa$ [M + Na]$^+$ 417.0885, found 417.0887.

(*Z*)-4-Methyl-*N*-(6-phenylhex-3-en-5-yn-2-yl-3-*d*)-*N*-(prop-2-yn-1-yl)benzenesulfonamide (1i)

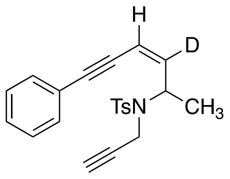

The title compound was obtained as colourless liquid (40 mg, 47%) after flash column chromatography (EtOAc:*n*-hexane 2:8) following the general procedure **Method-A** using 4-methyl-*N*-(prop-2-yn-1-yl)benzenesulfonamide (69.5 mg, 0.33 mmol, 1.1 equiv), (*Z*)-6-phenylhex-3-en-5-yn-3-*d*-2-ol **S5a (**50 mg, 0.28 mmol, 1.0 equv), PPh$_3$ (100.8 mg, 0.38 mmol, 1.3 equiv) and DIAD (0.07 mL, 0.38 mmol, 1.3 equiv). R$_f$ (EtOAc/hexane 2:8) = 0.56. **^{1}H NMR** (400 MHz, CDCl$_3$) δ 7.80 (d, *J* = 8.4 Hz, 2H, 2CH_{Ar}), 7.50 – 7.45 (m, 2H, 2CH_{Ar}), 7.35 – 7.32 (m, 3H, 3ArC*H*), 7.19 (d, *J* = 8.0 Hz, 2H, 2ArC*H*), 5.65 (s, 1H, =C*H*), 5.15 (q, *J* = 7.0 Hz, 1H, C*H*(CH$_3$)), 4.26 (dd, *J* = 18.6, 2.5 Hz, 1H, CH_A(N)), 4.10 (dd, *J* = 18.6, 2.5 Hz, 1H, CH_B(N)), 2.36 (s, 3H, CH_3(Ts)), 2.18 (t, *J* = 2.5 Hz, 1H, C*H* alkyne), 1.43 (d, *J* = 7.0 Hz, 3H, CH_3(CH)). **^{13}C NMR** (101 MHz, CDCl$_3$) δ 143.2, 140.5 (d, J_{CD} = 24.5), 137.6, 131.6, 129.3, 128.5, 128.4, 127.6, 123.0, 110.6, 95.8, 84.9, 79.7, 72.8, 53.4, 33.4, 21.5, 19.5. **IR** (ν_{max}/cm^{-1}) 2924 (w), 2863 (w), 1597(w), 1476 (w), 1402 (s), 1160 (s), 1091 (s), 1011 (s), 910 (m), 761 (m), 662 (s), 565 (s). **HRMS** (ESI-OTF) *m/z* calcd for $C_{22}H_{21}DNO_2S$ [M + H]$^+$ 365.1434, found 365.1423.

(*Z*)-4-Methyl-*N*-(2-methyl-5-phenylpent-2-en-4-yn-1-yl)-*N*-(prop-2-yn-1-yl)benzenesulfonamide (1j)

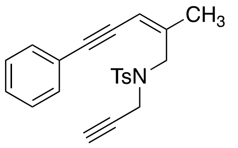

The title compound was obtained as colourless liquid (65 mg, 64%); after flash column chromatography (EtOAc:*n*-hexane 1.7:8.3) following the general procedure **Method-A** using 4-methyl-*N*-(prop-2-yn-1-yl)benzenesulfonamide **S10** (69.9 mg, 0.3 mmol, 1.15 equiv), (*Z*)-2-methyl-5-phenylpent-2-en-4-yn-1-ol **S2b** (50 mg, 0.29 mmol, 1equiv), PPh_3 (101 mg, 0.38 mmol, 1.3 equiv) and DIAD (0.07 mL, 0.3 mmol, 1.3 equiv), R_f (EtOAc:*n*-hexane 2:8) = 0.7. **^{1}H NMR** (500 MHz, $CDCl_3$) δ 7.79 (d, *J* = 8.3 Hz, 2H, 2ArC*H*), 7.35 – 7.29 (m, 4H, 4ArC*H*), 77.29 – 7.24 (m, 3H, 3ArC*H*), 5.75 (d, *J* = 1.3 Hz, 1H, =C*H*), 4.15 (s, 2H, $CH_2(CHCH_3)$), 4.06 (d, *J* = 2.5 Hz, 2H, CH_ACH_B(N)), 2.43 (s, 3H, Ts-CH_3), 1.93 (d, *J* = 1.5 Hz, 3H, CH_3(CH)), 1.89 (t, *J* = 2.4 Hz, 1H, alkyne-C*H*). **^{13}C NMR** (126 MHz, $CDCl_3$) δ 144.9, 143.6, 135.7, 131.2, 129.4, 128.2, 128.1, 127.9, 123.32, 110.6, 93.20, 85.7, 73.6, 49.1, 36.2, 21.6, 20.6. **IR** (ν_{max}/cm^{-1}) 3288 (w), 2977 (s), 2122 (s), 1597 (m), 1490 (m), 1402 (s), 1306 (m), 1152 (s), 1090 (s), 901 (m), 813 (m), 754 (s), 690 (s), 577 (s), 544 (s). **HRMS** (ESI-OTF) *m/z* calcd for $C_{22}H_{22}NO_2S$ $[M + H]^+$ 364.1371, found 364.1381.

Method-B: N-tethered enediynes using the Mitsunobu coupling reaction (1g).

***Tert*-butyl (*Z*)-(6-phenylhex-3-en-5-yn-2-yl)(tosyl)carbamate (S6)**

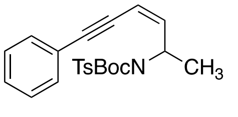

To a stirred solution of *tert*-butyl tosylcarbamate (157 mg, 0.58 mmol), (*Z*)-6-phenylhex-3-en-5-yn-2-ol (100 mg, 0.58 mmol) and PPh_3 (205 mg, 0.784 mmol) in dry THF (3 mL) at 0 °C

was added diethyl azadicorboxylate (126 mg, 0.726 mmol). The mixture was stirred at room temperature for 3 h. Water (1 mL) was added and the mixture extracted with EtOAc (2 mL × 3). The combined extracts were washed with brine (3 mL) dried (Na_2SO_4), filtered through a pad of silica gel and washed with EtOAc. The crude product was purified by column chromatography (EtOAc:*n*-hexane = 1:10 v/v) to provide as a white solid (151 mg, 62%), **MP:** 129 − 131°C, R_f (EtOAc:*n*-hexane 2:8) = 0.72. **^{1}H NMR** (500 MHz, $CDCl_3$) δ 7.83 (d, *J* = 8.4 Hz, 2H, 2CH_{Ar}), 7.54 – 7.48 (m, 2H, 2CH_{Ar}), 7.32 – 7.27 (m, 2H, 2ArC*H*), 7.22 (d, *J* = 7.7 Hz, 2H, 2ArC*H*), 6.43 (dd, *J* = 10.6, 8.7 Hz, 1H, =C*H*), 5.88 – 5.79 (m, 2H, =C*H* and C*H*), 2.38 (s, 3H, TsCH_3), 1.66 (d, *J* = 6.9 Hz, 3H, CH_3), 1.33 (s, 9H, C(CH_3)$_3$). **^{13}C NMR** (126 MHz, $CDCl_3$) δ 150.6, 143.6, 141.9, 138.0, 131.7, 129.0, 128.4, 128.3, 127.7, 122.9, 111.4, 96.0, 85.1, 84.2, 53.5, 27.9, 21.5, 20.5. **IR** (ν_{max}/cm^{-1}) 2982 (w), 2195 (m), 1719 (s), 1594 (s), 1491 (s), 1369 (s), 1249 (s), 1142 (s), 1087 (s), 994 (s), 760 (s), 693 (s), 579 (s). **HRMS** (ESI-OTF) *m/z* calcd for $C_{24}H_{27}NO_4SNa$ $[M + Na]^+$ 448.1559, found 448.1553

(*Z*)-4-Methyl-*N*-(6-phenylhex-3-en-5-yn-2-yl)benzenesulfonamide (S7)

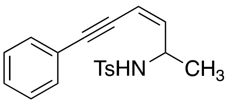

To a stirred solution of *tert*-butyl (*Z*)-(6-phenylhex-3-en-5-yn-2-yl) (tosyl)carbamate (131 mg, 0.30 mmol) in dry DCM (3 mL) at 0 °C was added trifluoroacetic acid (0.1 mL 1.53 mmol) dropwise and the reaction mixture was warm to room temperature and stirred for 3 h. The crude reaction mixture was extracted with DCM (3 mL × 3) and combined extracts were washed with aqueous $NaHCO_3$ (3 mL), dried (Na_2SO_4), filtered through pad of silica gel and concentrated under reduced pressure. The residue was purified by flash column chromatography (EtOAc:*n*-hexane = 2:8) to afford **S7** as a yellow oil (71 mg, 71%). R_f (EtOAc:*n*-hexane 2:8) = 0.35. **^{1}H NMR** (500 MHz, $CDCl_3$) δ 7.80 – 7.71 (m, 3H, 3CH_{Ar}),

7.40 – 7.36 (m, 2H, 2CH_{Ar}), 7.35 – 7.30 (m, 3H, ArC*H*), 7.24 – 7.13 (m, 2H, 2CH_{Ar}), 5.72 (dd, *J* = 10.7, 8.7 Hz, 1H, =C*H*), 5.58 (d, *J* = 10.8 Hz, 1H, =C*H*), 4.73 (br s, 1H, N*H*), 4.45 – 4.37 (m, 1H, C*H*(CH_3)), 2.33 (s, 3H, CH_3(Ts)), 1.29 (d, *J* = 6.7 Hz, 3H, CH_3(CH)). **^{13}C NMR** (101 MHz, $CDCl_3$) δ 142.9, 137.2, 131.5, 129.7, 129.5, 128.5, 128.3, 127.4, 127.3, 109.9, 95.6, 84.7, 5.65, 21.8, 21.4. **IR** (ν_{max}/cm^{-1}) 3270 (s), 2975 (s), 2199 (m), 1748 (s), 1490 (s), 1425 (s), 1321(s), 1156 (s), 1091 (s), 841 (s), 690 (s), 548 (s). **HRMS** (ESI-OTF) *m/z* calcd for $C_{19}H_{19}NO_2SNa$ [M + Na]$^+$ 348.1034, found 348.1037.

(*Z*)-4-Methyl-*N*-(pent-2-yn-1-yl)-*N*-(6-phenylhex-3-en-5-yn-2-yl)benzenesulfonamide (1g)

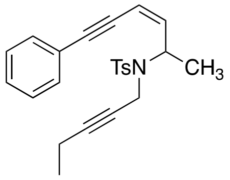

To a stirred solution of (*Z*)-4-methyl-*N*-(6-phenylhex-3-en-5-yn-2-yl)benzenesulfonamide **S7** (80 mg, 0.25 mmol, 1 equiv) in dry acetonitrile (1.2 mL) was added anhydrous K_2CO_3 (68 mg, 0.49 mmol, 2 equiv) and the resulting mixture stirred for 2 min, 1-bromopent-2-yne (0.03 mL, 0.31 mmol, 1.3 equiv) was added and the reaction stirred 80 °C for 5h. The mixture was cooled to room temperature and the solvent was removed under reduced pressure. the crude residue was purified by column chromatography (EtOAc:*n*-hexane = 1.5:8) to give pale yellow liquid (58 mg, 56%). R_f (EtOAc:*n*-hexane 2:8) = 0.6. **^{1}H NMR** (500 MHz, $CDCl_3$) δ 7.82 (d, *J* = 8.4 Hz, 2H, 2CH_{Ar}), 7.50 – 7.45 (m, 2H, 2CH_{Ar}), 7.37 – 7.31 (m, 3H, 3CH_{Ar}), 7.18 (d, *J* = 7.6 Hz, 1H, 1CH_{Ar}), 6.13 (dd, *J* = 10.8, 9.0 Hz, 1H, =C*H*), 5.63 (dd, *J* = 10.9, 1.0 Hz, 1H, =C*H*), 5.16 (dtd, *J* = 8.9, 7.0, 6.0 Hz, 1H, C*H*(CH_3)), 4.23 (dt, *J* = 18.4, 2.2 Hz, 1H, CH_A(N)), 4.07 (dt, *J* = 18.4, 2.3 Hz, 1H, CH_B(N)), 2.35 (s, 3H, CH_3(Ts)), 2.04 (qt, *J* = 7.5, 2.3 Hz, 2H, CH_2(alkyne)and(CH_3)), 1.41 (d, *J* = 7.0 Hz, 3H, CH_3(CH)), 1.00 (t, *J* = 7.5 Hz, 3H, CH_3(CH_2)).

^{13}**C NMR** (126 MHz, $CDCl_3$) δ 142.91, 141.4, 137.8, 131.5, 129.1, 128.4, 128.3, 127.7, 123.0, 110.1, 95.5, 86.4, 85.0, 75.1, 53.4, 33.9, 21.4, 19.6, 13.4, 12.2. **IR** (ν_{max}/cm^{-1}) 2976 (w), 2877 (w), 1597 (m), 1490 (s), 1332 (s), 1153 (s), 1030 (s), 904 (s), 814 (s), 754 (s), 691 (s), 544 (s). **HRMS** (ESI-OTF) *m/z* calcd for $C_{24}H_{26}NO_2S$ $[M + H]^+$ 392.1684, found 392.1677.

Method-C: General procedure for the synthesis of O-tethered enediynes (1k-s and 1t):

Synthesis of O-tethered enediynes **1k-s** and **1t** achieved via the reaction of the alcohol (S**3c, S5b,c,e** and **S5f-k**) with propargyl bromide using procedure reported in the literature.[12] To a stirred suspension of NaH 60% in mineral oil (1.2 equiv) dissolved in anhydrous THF were added enyn-ols (**S3c**, **S5b-c** and **S5e-k**) at 0 °C, then the reaction stirred for 45 min at the same temperature. Finally, a dropwise solution of commercially available propargyl bromide (2 equiv) was added dropwise and stirring was continued for another 3-4 h. Water was added, and the mixture was extracted with diethyl ether. The combined organic extracts were washed with brine and dried (Na_2SO_4). The pure products (**1k-s** and **1v**) were obtained after flash column chromatography using silica gel (ether:pentane 0.5:9.5).

(*Z*)-(5-(Prop-2-yn-1-yloxy) pent-3-en-1-yl) benzene (1k)

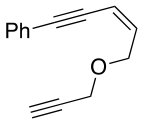

The tittle compound was obtained as yellow oil (96 mg, 79%) after flash column chromatography on silica gel using Et_2O/pentane (1:9) following general procedure **Method-C** using NaH (180 mg, 7.5 mmol, 1.2 equiv) in THF (2 mL), (*Z*)-5-phenylpent-2-en-4-yn-1-ol **S3c** (100 mg, 6.25 mmol, 1.0 equiv) propargyl bromide (80% in toluene, 0.11 mL, 2.0 equiv). R_f (Et_2O/pentane 1:9) = 0.76, ^{1}**H NMR** (500 MHz, $CDCl_3$) δ 7.44 (dd, *J* = 6.6, 3.0 Hz, 2H, 2ArC*H*), 7.32 (m, 3H, 3ArC*H*), 6.08 (dt, *J* = 10.9, 6.5 Hz, 1H, =C*H*), 5.88 (dt, *J* = 10.9, 1.5

Hz, 1H, =C*H*), 4.43 (dd, *J* = 6.5, 1.5 Hz, 2H, CH_2), 4.19 (d, *J* = 2.4 Hz, 2H, CH_2), 2.44 (t, *J* = 2.4 Hz, 1H, C*H*-alkyne). **^{13}C NMR** (126 MHz, $CDCl_3$) δ 138.2, 131.5, 128.4, 128.3, 123.1, 112.4, 112.4, 95.4, 85.0, 79.7, 74.6, 67.5, 57.5. **IR** (νmax/cm^{-1}) 3293 (m), 3032 (m), 2851 (m), 2116 (m), 1614 (s), 1489 (s), 1360 (s), 1264 (s), 1092 (s), 1026 (s), 911 (s), 689 (s), 527(s). **HRMS** (ESI-OTF) *m/z* calcd for $C_{14}H_{12}ONa$ $[M + Na]^+$ 219.0786, found 219.0793.

(*Z*)-(5-(Prop-2-yn-1-yloxy)hex-3-en-1-yn-1-yl)benzene (1l)

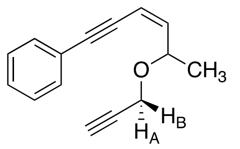

The title compound was obtained as a colourless oil (134 mg, 68%) after flash column chromatography (Et_2O/pentane 1:9) following the general synthesis procedure **Method-C** using NaH (60%, 46 mg, 1.86 mmol) THF (3 mL), (*Z*)-6-phenylhex-3-en-5-yn-2-ol **S5h** (170 mg, 0.9 mmol) and propargyl bromide (80% in toluene, 0.18 mL, 1.97 mmol, 2 equiv). R*f* (Et_2O/pentane 1:9) = 0.6. **^{1}H NMR** (500 MHz, $CDCl_3$) *δ* 7.45 – 7.44 (m, 2H, 2CH_{Ar}), 7.35 – 7.30 (m, 3H, 3CH_{Ar}), 5.91 – 5.80 (m, 1H, =C*H*), 4.76 (dq, *J* = 8.3, 6.4 Hz, 1H, C*H*(CH_3)), 4.20 (dd, *J* = 15.6, 2.4 Hz, 1H, CH_A(O)), 4.12 (dd, *J* = 15.6, 2.4 Hz, 1H, CH_B(O)), 2.40 (t, *J* = 2.4 Hz, 1H, C*H*-alkyne), 1.34 (d, *J* = 6.4 Hz, 3H, CH_3). **^{13}C NMR** (126 MHz, $CDCl_3$) *δ* 43.3, 131.4, 128.4, 128.3, 123.0, 112.0, 94.9, 85.0, 80.3, 77.2, 77.0, 76.7, 73.8, 72.5, 55.7, 20.7. **IR** (νmax/cm^{-1}) 3294 (w), 2975 (m), 2199 (m), 1725 (s), 1612 (s), 1584 (s), 1442 (s), 1283 (s), 1370 (s), 1248 (s), 1072 (s), 915 (s), 742 (s), 689 (s), 533 (s). **HRMS** (ESI-OTF) *m/z* calcd for $C_{15}H_{14}ON_a$ $[M + Na]^+$ 233.0942, found 233.0945.

(*E*)-(5-(Prop-2-yn-1-yloxy)pent-3-en-1-yn-1-yl)benzene (1l')

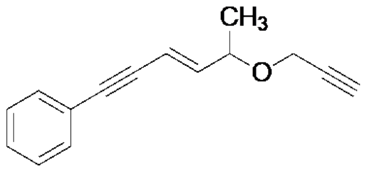

The title compound was obtained as a colourless oil (35 mg, 57 %) after flash column chromatography (Et_2O/pentane 0.5:9.5) following the general procedure **Method-C** using NaH (14 mg, 0.3 mmol) THF (2 mL), (*E*)-6-phenylhex-3-en-5-yn-2-ol (50 mg, 0.29 mmol), and propargyl bromide (80% in toluene, 0.05 mL). R*f* (Et_2O/pentane 2:8) = 0.6. **^{1}H NMR** (500 MHz, $CDCl_3$) δ 7.48 – 7.38 (m, 2H, 2CH_{Ar}), 7.33 – 7.27 (m, 3H, 3CH_{Ar}), 6.07 (dd, *J* = 15.9, 7.7 Hz, 1H, =C*H*), 5.93 (dd, *J* = 16.0, 0.8 Hz, 1H, , =C*H*), 4.21 (dd, *J* = 15.7, 2.4 Hz, 1H, CH_A(O)), 4.21– 4.19 (m, 1H, C*H*(CH_3), 4.08 (dd, *J* = 15.7, 2.3 Hz, 1H, CH_B(O)), 2.44 (t, *J* = 2.4 Hz, 1H, C*H*-alkyne), 1.33 (d, *J* = 6.4 Hz, 3H, CH_3(CH)). **^{13}C NMR** (126 MHz, $CDCl_3$) δ 143.6, 131.5, 128.3, 123.0, 112.1, 90.3, 87.0, 79.8, 74.7, 74.2, 55.47, 21.1. **IR** (vmax/cm^{-1}) 3058 (w), 2969 (m), 2853 (m), 1721 (s), 1600 (s), 1574 (s), 1442 (s), 1370 (s), 1128 (s), 1248 (s), 1070 (s), 881 (s), 760 (s), 697(s), 525 (s). **HRMS** (ESI-OTF) *m/z* calcd for $C_{15}H_{15}O$ [M + Na]$^+$ 211.1123, found 211.1124.

(*Z*)-1-Fluoro-4-(5-(prop-2-yn-1-yloxy)hex-3-en-1-yn-1-yl)benzene (1m)

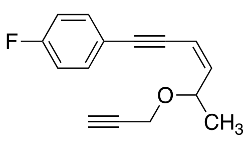

The tittle compound was obtained as a colorless oil (58 mg, 81%) after flash column chromatography (Et_2O:penatane 0.5:9.5) following the general procedure **Method-C** using NaH (15 mg, 0.37 mmol) THF (2 mL), (*Z*)-6-(4-fluorophenyl)hex-3-en-5-yn-2-ol **S5b** (60 mg, 0.31 mmol) and propargyl bromide (80% in toluene, 0.058 mL, 0.63 mmol, 2 equiv), R*f*

(Et_2O/pentane 1:9) = 0.77. **^{1}H NMR** (500 MHz, $CDCl_3$) δ 7.44 (dd, J = 8.9, 5.3 Hz, 1H, 2CH_{Ar}), 7.03 (t, J = 8.7 Hz, 2H, 2CH_{Ar}), 5.93 – 5.68 (m, 2H, 2=CH), 4.73 (dd, J = 7.1, 5.7 Hz, 1H, CH(CH_3)), 4.20 (dd, J = 15.6, 2.4 Hz, 1H, CH_A(O)), 4.11 (dd, J = 15.6, 2.4 Hz, 1H, CH_B(O)), 2.41 (t, J = 2.4 Hz, 1H, C*H-alkyne*), 1.34 (d, J = 6.4 Hz, 3H, CH_3(CH)). **^{13}C NMR** (126 MHz, $CDCl_3$) δ 162.5 (d, J_{CF} = 250.0 Hz), 143.3, 133.3 (d, J_{CF} = 8.4 Hz), 119.1 (d, J_{CF}= 3.4 Hz), 115.7 (d, J_{CF} = 22.1 Hz), 111.9, 93.8, 84.7 (d, J_{CF} = 1.5 Hz), 80.3, 73.8, 72.3, 55.7, 20.7. **IR** (ν_{max}/cm^{-1}) 3291 (w), 2977 (w), 2202 (w), 1724 (m), 1664 (m), 1599 (w), 1505 (s), 1443 (m), 1227 (s), 1156 (m), 1073 (s), 835 (s), 632 (m), 527 (s). **HRMS** (ESI-OTF) *m/z* calcd for $C_{15}H_{13}OFNa$ [M + Na]$^+$ 251.0848, found 251.0846.

(*Z*)-1-Methoxy-4-(5-(prop-2-yn-1-yloxy)hex-3-en-1-yn-1-yl)benzene (1n)

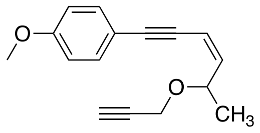

The tittle compound was obtained colourless oil (38 mg, 51%) after flash column chromatography (Et_2O/pentane 0.7:9.3) following the general procedure **Method-C** using NaH (11.8 mg, 0.29 mmol) THF (2 mL), (*Z*)-6-(4-methoxyphenyl)hex-3-en-5-yn-2-ol **S5c** (50 mg, 0.2 mmol), and propargyl bromide (80% in toluene, 0.046 mL, 0.49 mmol, 2 equiv). R*f* *(*Et_2O/pentane 1:9) = 0.5. **^{1}H NMR** (400 MHz, $CDCl_3$) δ 7.39 (d, J = 8.8 Hz, 2H, 2CH_{Ar}), 6.85 (d, J = 8.8 Hz, 2H, 2CH_{Ar}), 5.86 (d, J = 10.9 Hz, 1H, =CH), 5.79 (dd, J = 10.8, 8.7 Hz, 1H, =CH), 4.82 – 4.68 (m, 1H, CH(CH_3)), 4.20 (dd, J = 15.6, 2.4 Hz, 1H, CH_A(O)), 4.11 (dd, J = 15.6, 2.4 Hz, 1H, CH_B(O)), 3.82 (s, 3H, OCH_3), 2.39 (t, J = 2.4 Hz, 1H, CH alkyne), 1.34 (d, J = 6.4 Hz, 3H, CH_3(CH)). **^{13}C NMR** (101 MHz, $CDCl_3$) δ 159.7, 142.5, 132.9, 115.2, 114.0, 112.2, 94.9, 83.8, 80.4, 73.7, 72.5, 55.7, 55.3, 20.7. **IR** (ν_{max}/cm^{-1}) 3343 (w), 2970 (s), 2933 (m), 1648 (m), 1468 (m), 1408 (m), 1379 (s), 1305 (m), 1128 (s), 816 (m), 633 (m). **HRMS** (ESI-OTF) *m/z* calcd for $C_{16}H_{16}O_2Na$ [M + Na]$^+$ 263.1043 found 263.1042.

(*Z*)-2-(Prop-2-yn-1-yloxy)undec-3-en-5-yne (1o)

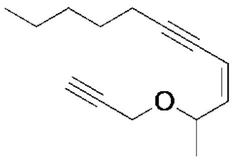

The title compound was obtained as a pale yellow liquid (28 mg, 46%) after flash column chromatography on silica gel (Et_2O:penatane 0.3:9.7) following the general procedure **Method-C** using NaH (13.2 mg, 0.33 mmol) THF (2 mL), (*Z*)-undec-3-en-5-yn-2-ol **S5e** (50 mg, 0.27 mmol) and propargyl bromide (80% in toluene, 0.058 mL, 0.5 mmol, 2 equiv); R_f (Et_2O/pentane 1:9) = 0.78. **^{1}H NMR** (500 MHz, $CDCl_3$) δ 5.69 (dd, *J* = 10.8, 8.6 Hz, 1H, =C*H*), 5.63 (dt, *J* = 11.0, 2.1 Hz, 1H, =C*H*), 4.62 (dq, *J* = 8.6, 6.4 Hz, 1H, C*H*CH_3), 4.16 (dd, *J* = 15.5, 2.4 Hz, 1H, CH_A(O)), 4.06 (dd, *J* = 15.6, 2.4 Hz, 1H, CH_B(O)), 2.39 (t, *J* = 2.4 Hz, 1H, C*H*-alkyne), 2.33 (td, *J* = 7.1, 2.1 Hz, 2H, C$H_2$$CH_2$), 1.59 – 1.51 (m, 2H, C$H_2$$CH_2$), 1.44 – 1.31 (m, 4H, C$H_2$$CH_2$), 1.29 (d, *J* = 6.3 Hz, 3H, CH_3(CH)), 0.91 (t, *J* = 7.2 Hz, 3H, CH_3(CH_2)). **^{13}C NMR** (126 MHz, $CDCl_3$) δ 142.0, 112.5, 96.4, 80.4, 76.2, 73.5, 72.5, 55.6, 31.1, 28.3, 22.2, 20.7, 19.4, 14.0. **IR** (ν_{max}/cm^{-1}) 3313 (w), 2957 (s), 2871 (s), 1645 (m), 1460 (m), 1378 (m), 1080 (m), 1014 (s), 624 (m). **HRMS** (ESI-OTF) *m/z* calcd for $C_{14}H_{20}OSNa$ [M + Na]$^+$ 227.1406 found 227.1407

(*Z*)-(5-(Prop-2-yn-1-yloxy)oct-3-en-1-yn-1-yl)benzene (1p)

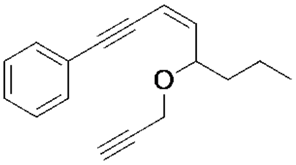

The compound was obtained as aa pale yellow liquid (36 mg, 61%) after flash column chromatography on silica gel (ether:penatane 0.5:9.5) following general procedure **Method-C**

using NaH (12 mg, 0.3 mmol) THF (2 mL), (*Z*)-8-phenyloct-5-en-7-yn-4-ol **S5f** (50 mg, 0.25 mmol) and propargyl bromide (80% in toluene, 0.047 mL, 0.5 mmol, 2 equiv), R_f (Et_2O/pentane 2:8) = 0.8. **^{1}H NMR** (400 MHz, $CDCl_3$) δ 7.49 – 7.41 (m, 2H, 2ArC*H*), 7.38 – 7.29 (m, 3H, 3ArC*H*), 5.92 (dd, *J* = 10.8, 0.7 Hz, 1H, =C*H*), 5.81 (dd, *J* = 10.9, 9.1 Hz, 1H, =C*H*), 4.70 – 4.54 (m, 1H, C*H*CH_3), 4.21 (dd, *J* = 15.6, 2.4 Hz, 1H, CH_A(O)), 4.11 (dd, *J* = 15.6, 2.4 Hz, 1H, CH_B(O)), 2.38 (t, *J* = 2.4 Hz, 1H, C*H*-alkyne), 1.74 – 1.63 (m, 1H, C*H*CH_2), 1.57 – 1.37 (m, 3H, *3CH*CH_2), 0.96 (t, *J* = 7.2 Hz, 3H, (CH_2CH_2)CH_3). **^{13}C NMR (**101 MHz, $CDCl_3$) δ 142.7, 131.4, 128.4, 128.3, 128.3, 128.3, 123.1, 112.7, 94.6, 85.3, 80.4, 76.1, 73.6, 55.8, 36.9, 18.4. **IR** (ν_{max}/cm^{-1}) 1326 (w), 3061 (m), 2959 (m), 2932 (m), 2872 (m), 1597 (m), 1490 (m), 1360 (m), 1269 (m), 1072 (s), 756 (s), 690 (s), 524 (m). **HRMS** (ESI-OTF) *m/z* calcd for $C_{17}H_{18}ONa$ $[M + Na]^+$ 261.1255 found 261.1265.

(*Z*)-(6-Methyl-5-(prop-2-yn-1-yloxy)hept-3-en-1-yn-1-yl)benzene (1q)

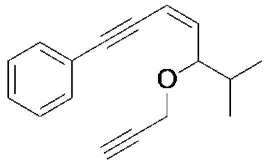

The title compound was obtained as a pale yellow liquid (18 mg, 25%) after flash column chromatography on silica gel (Et_2O:pentane 0.4:9.6) following the general procedure **Method-C** using NaH (14.4 mg, 0.36 mmol) THF (2.5 mL), (*Z*)-2-methyl-7-phenylhept-4-en-6-yn-3-ol **S5f** (60 mg, 0.3 mmol, 1 equiv) and propargyl bromide (0.56 mL, 0.6 mmol, 2 equiv), R_f (Et_2O/pentane 2:8) = 0.71. **^{1}H NMR** (400 MHz, $CDCl_3$) δ 7.49 – 7.41 (m, 2H, 2ArC*H*), 7.33 – 7.28 (m, 3H, 2ArC*H*), 5.98 (dd, *J* = 10.9, 0.7 Hz, 1H, =C*H*(CH)OH), 5.82 (dd, *J* = 11.0, 9.4 Hz, 1H, =C*H*(alkyne)), 4.34 (ddd, *J* = 9.4, 6.7, 0.7 Hz, 1H, C*H*(isopropyl)), 4.22 (dd, *J* = 15.7, 2.4 Hz, 1H, C*H*(O)propargyl), 4.10 (dd, *J* = 15.7, 2.4 Hz, 1H, C*H*(O)propargyl), 2.37 (t, *J* = 2.4 Hz, 1H, C*H*(propargyl alkyne), 1.93 – 1.80 (m, 1H, C*H*(CH_3, CH_3), 1.03 (d, *J* = 6.7 Hz, 3H, CH_3), 0.94 (d, *J* = 6.9 Hz, 3H, CH_3). **^{13}C NMR** (101 MHz, $CDCl_3$) δ 141.1, 131.4, 128.4,

128.3, 123.2, 113.7, 94.4, 85.6, 81.3, 80.6, 73.5, 55.98, 32.7, 18.6, 18.0. **IR** (ν_{max}/cm^{-1}) 2961 (w), 2921 (w), 2852 (w), 1258 (s), 1078 (s) 1011 (s), 865 (m), 791 (s), 691 (w). **HRMS** (ESI-OTF) *m/z* calcd for $C_{17}H_{18}ONa$ [M + Na]$^+$ 261.1255 found 261.1263.

(*Z*)-(5-(Prop-2-yn-1-yloxy)pent-3-en-1-yne-1,5-diyl)dibenzene (1r)

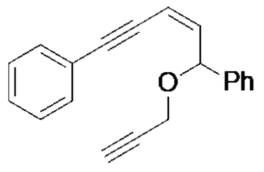

The title compound was obtained as a pale yellow liquid (32 mg, 58%) after flash column chromatography (Et_2O : penatane 0.5:9.5) following general synthesis procedure **Method-C** using NaH (10.2 mg, 0.2 mmol, 1.2 equiv) THF (2 mL), (*Z*)-1,5-diphenylpent-2-en-4-yn-1-ol **S5j** (50 mg, 0.2 mmol, 1 equiv) and propargyl bromide (0.04 mL, 0.42 mmol, 2 equiv), R_f (Et_2O/pentane 2:8) = 0.67. **^{1}H NMR** (500 MHz, $CDCl_3$) δ 7.52 – 7.47 (m, 2H, 2ArC*H*), 7.49 – 7.43 (m, 2H, 2ArC*H*), 7.40 – 7.32 (m, 5H, 5ArC*H*), 7.34 – 7.26 (m, 1H, ArC*H*), 6.08 (dd, *J* = 10.7, 9.0 Hz, 1H, =C*H*), 5.92 (d, *J* = 10.6 Hz, 1H, =C*H*), 5.77 (d, *J* = 9.0 Hz, 1H, C*H*(Ph)), 4.29 – 4.13 (m, 2H, CH_AH_B(O)), 2.43 (t, *J* = 2.3 Hz, 1H, C*H*-alkyne). **^{13}C NMR** (126 MHz, $CDCl_3$) δ 141.6, 139.9, 131.5, 128.6, 128.6, 128.5, 128.5, 128.4, 128.3, 127.9, 126.6, 123.0, 111.7, 95.2, 85.2, 79.8, 77.8, 74.4, 55.6. **IR** (ν_{max}/cm^{-1}) 3291 (w), 3060 (w), 2854 (w), 1596 (m), 1489 (m), 1442 (m), 1062 (s), 1026 (m), 914 (w), 753 (s), 690 (s), 525 (m). **HRMS** (ESI-OTF) *m/z* calcd for $C_{20}H_{16}ONa$ [M + Na]$^+$ 295.1099 found 295.1087.

(*Z*)-(3-Methyl-5-(prop-2-yn-1-yloxy)hex-3-en-1-yn-1-yl)benzene (1s)

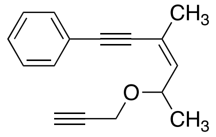

The title compound obtained as pale yellow liquid (89 mg, 74%) after flash column chromatography (Et_2O:pentane 0.8:9.2) following general synthesis procedure **Method-C** using NaH (25.7 mg, 0.64 mmol) THF (2.5 mL), (*Z*)-4-methyl-6-phenylhex-3-en-5-yn-2-ol **S5g** (100 mg, 0.53 mmol) and propargyl bromide (80% in toluene, 0.1 mL, 1.07 mmol, 2 equiv), R_f (Et_2O/pentane 2:8) = 0.8. **^{1}H NMR** (400 MHz, $CDCl_3$) δ 7.50 – 7.41 (m, 2H, 2ArC*H*), 7.35 – 7.28 (m, 3H, 3ArC*H*), 5.59 (dd, *J* = 9.1, 1.5 Hz, 1H, =C*H*), 4.67 (dq, *J* = 9.1, 6.3 Hz, 1H, C*H*CH_3), 4.19 (dd, *J* = 15.5, 2.4 Hz, 1H, CH_A(O)), 4.10 (dd, *J* = 15.6, 2.4 Hz, 1H, CH_B(O)), 2.37 (t, *J* = 2.4 Hz, 1H, C*H-alkyne*), 1.98 (d, *J* = 1.5 Hz, 3H, CH_3(alkene)), 1.31 (d, *J* = 6.4 Hz, 3H, CH_3(CH)). **^{13}C NMR (**101 MHz, $CDCl_3$) δ 138.0, 131.5, 128.3, 128.3, 123.1, 121.8, 94.0, 87.5, 80.6, 73.5, 73.3, 55.5, 23.2, 20.9. **IR** (ν_{max}/cm^{-1}) 3296 (w), 3081 (m), 2923 (m), 1629 (m), 1597 (m), 1490 (m), 1267 (m), 1141 (s), 1093 (s), 1046 (s), 926 (s), 756 (s), 835 (s), 758 (s), 668 (s), 527 (m). **HRMS** (ESI-OTF) *m/z* calcd for $C_{16}H_{17}O$ $[M + H]^+$ 225.1274 found 225.1275.

(*Z*)-(3-((5-Phenylpent-2-en-4-yn-1-yl)oxy)prop-1-yn-1-yl)benzene (1t)

This title compound was prepared according a literature synthesis procedure.[14]

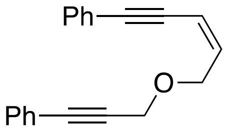

A stirred suspension of NaH (48 mg, 1.25 mmol, 2 equiv) in dry THF (3 mL) cooled to 0 °C and (*Z*)-5-phenylpent-2-en-4-yn-1-ol (**S3c**) (100 mg, 0.625 mmol, 1 equiv) THF (1 mL) and the mixture stirred for the 45 min. To this slurry was added a dropwise a solution of (3-chloroprop-1-yn-1-yl)benzene (0.1 mL, 0.75 mmol, 1.2 equiv), the reaction mixture warm to room temperature and stirred for 6 h. Ice water was added and the mixture was extracted with diethyl ether (3x2 mL). The combined extracts were dried (Na_2SO_4) and evaporated under

reduced pressure. The crude product was purified by flash column chromatography on silica gel using Et_2O/pentane (1.5:8.5) to give the tittle compound as a yellow oil (80 mg, 47%), R_f (EtOAc:*n*-hexane 2:8) = 0.75. **^{1}H NMR** (500 MHz, $CDCl_3$) δ 7.41 (m, 2H, 2ArC*H*), 7.33 – 7.19 (m, 3H, 3ArC*H*), 6.13 (dt, *J* = 10.8, 6.6 Hz, 1H, =C*H*), 5.91 (d, *J* = 10.9 Hz, 1H, =C*H*), 4.51 (dd, *J* = 6.6, 1.5 Hz, 2H, CH_2-alkene), 4.43 (s, 2H, CH_2-propagyl). **^{13}C NMR** (126 MHz, $CDCl_3$) δ 138.3, 131.8, 131.5, 128.4, 128.3, 128.3, 128.3, 128.2, 123.0, 122.6, 112.4, 95.4, 86.4, 85.0, 85.0, 67.4, 58.2. **IR** (ν_{max}/cm^{-1}) 3293 (w), 3033 (w), 2851 (w), 2117 (w), 1614 (m), 1441 (m), 1335 (m), 1092 (s), 940 (m), 754 (s), 689 (s), 633 (s), 527 (s). **HRMS** (ESI-OTF) *m/z* calcd for $C_{20}H_{17}O$ $[M + H]^+$ 273.1279 found 273.1269.

Method-D: Synthesis of C-tethered enediyne (1u):

This C-tethered Enediyne (**1u**) was prepare according to a literature procedure.[15]

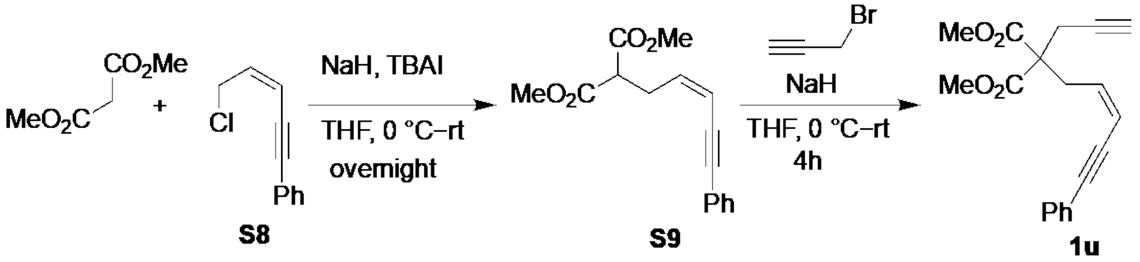

(*Z*)-(5-Chloropent-3-en-1-yn-1-yl)benzene (S8)

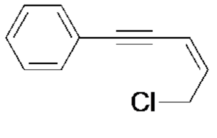

In an oven dried Schlenk flask, (*Z*)-5-phenylpent-2-en-4-yn-1-ol and MsCl (0.21 mL, 2.8 mmol, 1.15 equiv) were dissolved in dry DCM (3.1 mL) then the resulting solution was cooled to 0 °C, Et_3N (0.35 mL, 2.56 mmol, 1.6 equiv) was added dropwise and the reaction mixture was warmed to room temperature and stirred overnight. The ice water was added, and the mixture was extracted with DCM (3×2 mL). The combined extracts were washed with 1M

HCl, saturated $NaHCO_3$ and brine, dried (Na_2SO_4). The crude product was purified by flash column chromatography (ether:pentane 0.5:8.5) to give **S8** as a colorless oil (240 mg, 87%); R_f (ether:pentane 1:9) = 0.67. **^{1}H NMR** (500 MHz, $CDCl_3$) δ 7.48 – 7.43 (m, 2H, 2ArC*H*), 7.35 – 7.32 (m, 3H, 3ArC*H*), 6.09 (dt, *J* = 10.5, 7.5 Hz, 1H, =C*H*), 5.88 (d, *J* = 10.5 Hz, 1H, =C*H*), 4.38 (dd, *J* = 7.6, 1.0 Hz, 2H, C*H*$_2$(Cl)). **^{13}C NMR** (126 MHz, $CDCl_3$) δ 136.9, 131.5, 128.7, 128.4, 122.7, 113.1, 96.6, 84.0, 41.1. **IR** (ν_{max}/cm^{-1}) 3033 (w), 2862 (w), 2195 (w), 1727 (m), 1489 (m), 1254 (m), 753 (s), 687 (s). **HRMS** (ESI-OTF) *m/z* calcd for C_9H_7ClNa [M + Na]$^+$ 173.0129 found 173.0121.

Synthesis of dimethyl (*Z*)-2-(5-phenylpent-2-en-4-yn-1-yl)malonate (S9)

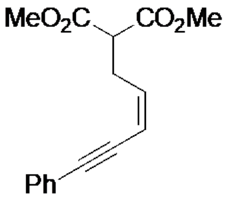

This compound was prepared according to a literature procedure to afford the title compound as a colourless oil (169 mg, 82%). **^{1}H NMR** (500 MHz, $CDCl_3$) δ 7.48 – 7.42 (m, 2H), 7.32 (dd, *J* = 5.0, 2.0 Hz, 3H), 5.99 – 5.92 (m, 1H), 5.82 – 5.72 (m, 1H), 3.75 (s, 8H), 3.57 (t, *J* = 7.5 Hz, 1H), 2.99 (td, *J* = 7.5, 1.3 Hz, 2H). **^{13}C NMR** (126 MHz, $CDCl_3$) δ 169.39, 138.30, 131.71, 128.53, 128.51, 123.44, 95.16, 85.50, 52.84, 51.22, 29.60. NMR data matches literature values.[16]

Synthesis of dimethyl (*Z*)-2-(5-phenylpent-2-en-4-yn-1-yl)-2-(prop-2-yn-1-yl)malonate (1u)

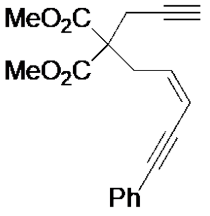

A solution of **S9** (50 mg, 0.18 mmol, 1.0 equiv) in THF (2.5 mL) was added dropwise to a slurry solution of NaH (14 mg, 0.36 mmol, 2 equiv, 60% wt) in dry THF (1.8 mL) at 0 °C. After 40 min a homogeneous suspension was formed and propargyl bromide (0.02 mL, 0.22 mmol, 1.2 equiv) was added, the resulting suspension was stirred for 4 h at 25 °C and water was added to the mixture extracted with diethyl ether (3×2 mL). The extracts were washed with brine and dried (Na_2SO_4). The crude product was purified by flash column chromatography on silica gel (0.5:9.5 Et_2O/pentane) to give as a pale-yellow oil (41 mg, 72%), R*f* (Et_2O/pentane 0.5:9.5) = 0.48. **^{1}H NMR** (400 MHz, $CDCl_3$) δ 7.48 – 7.42 (m, 2H, 2ArC*H*), 7.34 – 7.28 (m, 3H, 3ArC*H*), 5.89 – 5.81 (m, 2H, 2=C*H*), 3.75 (s, 6H, 2OC*H*3), 3.19 (dd, *J* = 4.9, 1.6 Hz, 2H, C*H*2-alkene), 2.86 (d, *J* = 2.7 Hz, 2H, C*H*2-propargyl), 2.01 (t, *J* = 2.7 Hz, 1H, propargyl-C*H*). **^{13}C NMR** (101 MHz, $CDCl_3$) δ 170.1, 136.0, 131.5, 128.3, 128.3, 128.2, 123.3, 113.5, 94.5, 85.4, 78.7, 71.6, 56.9, 52.9, 33.1, 23.2. **IR** (ν_{max}/cm^{-1}) 3292(w), 2923 (w), 1732 (s), 1595 (m), 1436 (m), 1288 (m), 1199 (s), 1029 (m), 755 (s), 690 (s), 526 (w). **HRMS** (ESI-OTF) *m/z* calcd for $C_{19}H_{18}O_4Na$ [M + Na]$^+$ 333.1103 found 333.1116.

4. General procedure for the rhodium(I)-catalyzed cycloaromatization of enediynes:

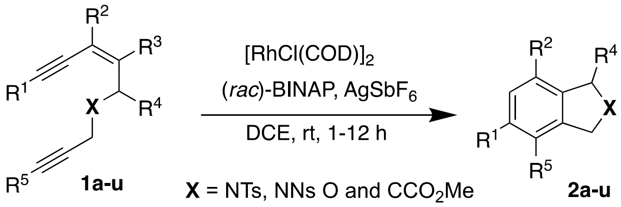

In an oven-dried Schlenk flask, $[Rh(COD)Cl]_2$ (5 mol%), (rac)-BINAP (12 mol%) was dissolved in dry DCE (0.5 mL) then immediately $AgSbF_6$ (20 mol%) was added under a N_2 atmosphere. The resulting mixture was stirred for 2 min to dissolve the catalyst to form the active cationic Rh(I)-catalyst, the enediyne **1a-u** (20 mg, 1.0 equiv) in DCE (0.5), was added and the solution was then degassed and stirred at room temperature for 1-12 h. The crude

reaction mixture was filtered through by small silica bed then concentrated under reduced pressure. The crude product was purified by silica gel flash column chromatography to provide the corresponding pure products **2a-u**.

1-Methyl-5-phenyl-2-tosylisoindoline (2a)

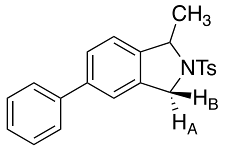

The product was obtained as a white solid (15.7 mg, 79%) after flash column chromatography (Et_2O/pentane 2:8) following the general procedure using *Z*)-4-methyl-*N*-(6-phenylhex-3-en-5-yn-2-yl)-*N*-(prop-2-yn-1-yl)benzenesulfonamide **1a** (20 mg, 0.055 mmol) in DCE (0.5 mL), $[Rh(COD)Cl]_2$ (1.3 mg, 0.002mmol) , (±)-BINAP (4.1 mg, 0.006 mmol) and $AgSbF_6$ (3.7 mg, 0.011 mmol) in DCE (0.5 mL), the solution was stirred at room temperature for 12 h. **Mp:** 131−136 °C, R_f (EtOAc/hexane 1:9) = 0.39. ^{1}**H NMR** (500 MHz, $CDCl_3$) δ 7.78 – 7.76 (d, *J* = 8.3 Hz, 2H, 2CH_{Ar}), 7.51 – 7.49 (d, *J* = 7.5 Hz, 2H, 2CH_{Ar}), 7.46 (d, *J* = 9.5 Hz, 1H, 1CH_{Ar}), 7.42 (t, *J* = 7.6 Hz, 2H, , 2CH_{Ar}), 7.34 (m, 2H, , 2CH_{Ar}), 7.30 – 7.28 (d, *J* = 8 Hz, 2H, , 2CH_{Ar}), 4.94 (dt, *J* = 8.7, 4.3 Hz, 1H, C*H*(CH_3)), 4.79 (dd, *J* = 13.9, 2.6 Hz, 1H, CH_A(N)), 4.60 (d, *J* = 13.5 Hz, 1H, CH_B(N)), 2.38 (s, 3H, CH_3(Ts)), 1.72 (d, *J* = 6 Hz, 3H, CH_3(CH)). ^{13}**C NMR** (126 MHz, $CDCl_3$) δ 143.6, 141.2, 140.6, 140.4, 135.5, 134.2, 129.8, 128.8, 127.5, 127.5, 127.0, 126.9, 122.6, 121.1, 61.7, 53.7, 23.8, 21.5. **IR** (ν_{max}/cm^{-1}) 2973 (w), 1597 (w), 1486 (w) 1344 (s), 1160 (s), 1090 (s), 758 (s), 661 (s), 553 (s). **HRMS** (ESI-OTF) *m/z* calcd for $C_{22}H_{21}NO_2N_aS$ $[M + H]^+$ 364.1371, found 364.1365.

5-Phenyl-2-tosylisoindoline (2b)

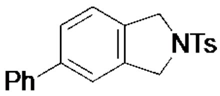

The product was obtained as a white solid (14.2 mg, 71%) after flash column chromatography (Et_2O/pentane 1.5:8.5) following the general synthesis procedure using (*Z*)-4-methyl-*N*-(5-phenylpent-2-en-4-yn-1-yl)-*N*-(prop-2-yn-1-yl)benzenesulfonamide **1b** (20 mg, 0.05 mmol) in DCE (0.5 mL), $[Rh(COD)Cl_2$ (1.41 mg, 0.002mmol), (±)-BINAP (4.27 mg, 0.006 mmol) and $AgSbF_6$ (3.9 mg, 0.01 mmol) in DCE (0.5 mL), the solution was stirred at room temperature for 12 h. R_f (EtOAc/hexane 2:8) = 0.65. **^{1}H NMR** (500 MHz, $CDCl_3$) δ 7.79 (d, *J* = 8.3 Hz, 2H), 7.52 – 7.49 (m, 2H,), 7.45 – 7.40 (m, 2H), 7.38 – 7.34 (m, 2H), 7.34 – 7.31 (m, 2H), 7.24 (d, *J* = 7.6 Hz, 1H), 4.67 (s, 2H), 4.66 (s, 2H), 2.41 (s, 3H). **^{13}C NMR** (126 MHz, $CDCl_3$) δ 143.7, 141.2, 140.4, 136.7, 135.0, 129.8, 128.8, 127.6, 127.5, 127.0, 126.8, 122.9, 121.3, 53.7, 53.5, 21.5. NMR data matches literature values.[17]

5-(4-Fluorophenyl)-1-methyl-2-tosylisoindoline (2c)

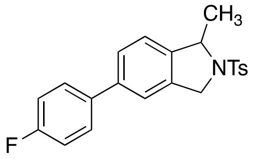

The product was obtained as a pale yellow solid (24.1 mg, 80%) after flash column chromatography (Et_2O/pentane 2:8) following the general procedure using 5-(4-fluorophenyl)-1-methyl-2-tosylisoindoline **1c** (30 mg, 0.078 mmol) in DCE (0.5 mL), $[Rh(COD)Cl_2$ (1.94 mg, 0.003 mmol), (±)-BINAP (5.87 mg, 0.009 mmol) and $AgSbF_6$ (5.4 mg, 0.15 mmol) in DCE (0.5 mL), the solution was stirred at room temperature for 12 h. **Mp:** 58−65 °C, R_f (EtOAc/hexane 2:8) = 0.66. **^{1}H NMR** (400 MHz, $CDCl_3$) δ 7.77 (d, *J* = 8.3 Hz, 2H, 2CH_{Ar}), 7.49 – 7.42 (m, 2H, 2CH_{Ar}), 7.42 – 7.36 (m, 1H, 1CH_{Ar}), 7.32 – 7.27 (m, 3H, 3CH_{Ar}), 7.16 (d,

J = 7.9 Hz, 1H, 1CH_{Ar}), 7.12 – 7.06 (m, 2H, 2CH_{Ar}), 5.00 – 4.89 (m, 1H, CH(CH_3)), 4.77 (dd, J = 13.8, 2.6 Hz, 1H, CH_A(N)), 4.61 (dd, J = 13.9, 1.1 Hz, 1H, CH_B(N)), 2.38 (s, 3H, CH_3(Ts)), 1.70 (d, J = 6.4 Hz, 3H, CH_3(CH)). **^{13}C NMR** (101 MHz, $CDCl_3$) δ 162.5 (d, J_{CF} = 246.9 Hz), 143.6, 140.7, 140.3, 136.7 (d, J_{CF} = 3.3 Hz), 135.7, 134.6, 129.8, 128.6 (d, J_{CF} = 8.1 Hz), 127.5, 126.8, 122.7, 121.0, 115.7 (d, J_{CF} = 21.6 Hz), 61.7, 53.7, 23.8, 21.4. **IR** (ν_{max}/cm^{-1}) 2966 (w), 2925 (w), 1598 (m), 1518 (m), 1490 (m), 1340 (s), 1222 (s), 1158 (s), 1092 (m), 1035 (m), 888 (m), 813 (m), 761 (m), 707 (m), 663 (m), 626 (s), 597 (s), 577 (s). **HRMS** (ESI-OTF) *m/z* calcd for $C_{22}H_{21}NO_2FSNa$ $[M + Na]^+$ 404.1096, found 404.1080.

5-(4-Methoxyphenyl)-1-methyl-2-tosylisoindoline (2d)

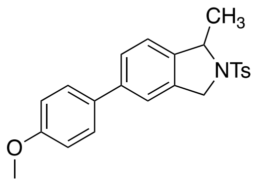

The product was obtained as a white crystalline powder (15.6 mg, 78%), after flash column chromatography (Et_2O/pentane 2:8) following the general procedure using (*E/Z*)-*N*-(6-(4-methoxyphenyl)hex-3-en-5-yn-2-yl)-4-methyl-*N*-(prop-2-yn-1-yl)benzenesulfonamide **1d** and **1d'** (20 mg, 0.050 mmol) in DCE (0.5 mL), $[Rh(COD)Cl]_2$ (1.25 mg, 0.002mmol), (±)-BINAP (3.8 mg, 0.006 mmol) and $AgSbF_6$ (3.49 mg, 0.01 mmol), in DCE (0.5 mL), the solution was stirred at room temperature for 12 h. **Mp:** 135−138 °C, R_f (EtOAc/hexane 2:8) = 0.30. **^{1}H NMR** (400 MHz, $CDCl_3$) δ 7.77 (d, J = 8.3 Hz, 2H, 2ArCH), 7.46 – 7.38 (m, 3H, 3ArCH), 7.30 – 7.26 (m, 2H, 2ArCH), 7.13 (d, J = 7.9 Hz, 1H, ArCH), 6.95 (d, J = 8.8 Hz, 2H, 2ArCH), 4.94 (qd, J = 6.4, 2.4 Hz, 1H, CH(CH_3)), 4.77 (dd, J = 13.8, 2.6 Hz, 1H, CH_A(N)), 4.60 (dd, J = 13.8, 1.0 Hz, 1H, CH_B(N)), 3.84 (s, 3H, OCH_3), 2.38 (s, 3H, CH_3(Ts)), 1.70 (d, J = 6.4 Hz, 3H, CH_3(CH)). **^{13}C NMR** (101 MHz, $CDCl_3$) δ 159.3, 143.5, 140.8, 140.1, 135.6, 134.6, 133.0, 129.7, 128.1, 127.5, 126.5, 122.6, 120.6, 114.3, 61.7, 55.3, 53.7, 23.8, 21.4. **IR** (ν_{max}/cm^{-1})

3345 (w), 2985 (w), 1065 (m), 1520 (m), 1491 (m), 1437 (m), 1251 (m), 1185 (m), 1161 (m), 1088 (m), 881 (w), 763 (s), 662 (m), 600 (m), 544 (s), 525 (s), 492 (s). **HRMS** (ESI-OTF) *m/z* calcd for $C_{23}H_{24}NO_3S$ $[M + H]^+$ 394.1477, found 394.1491.

1-Methyl-5-pentyl-2-tosylisoindoline (2e)

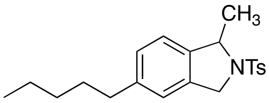

The product was obtained as a colourless liquid (16.5 mg, 83%) after flash column chromatography (Et_2O/pentane 1.5:8.5) following the general procedure using (*Z*)-4-methyl-*N*-(prop-2-yn-1-yl)-*N*-(undec-3-en-5-yn-2-yl)benzenesulfonamide **1e** (20 mg, 0.05 mmol) in DCE (0.5 mL), $[Rh(COD)Cl_2$ (3.06 mg, 0.002 mmol), (±)-BINAP (3.9 mg, 0.006 mmol) and $AgSbF_6$ (3.63 mg, 0.01 mmol) in DCE (0.5 mL), the solution was stirred at room temperature for 12 h. R_f (EtOAc/hexane 2:8) = 0.45. **^{1}H NMR** (500 MHz, $CDCl_3$) δ 7.75 (d, *J* = 8.3 Hz, 2H, 2ArC*H*), 7.29 – 7.23 (d, *J* = 8.3 Hz, 2H, 2ArC*H*), 7.04 (dd, *J* = 7.8, 1.5 Hz, 1H, ArC*H*), 6.99 (d, *J* = 7.9 Hz, 1H, ArC*H*), 6.94 (s, 1H, ArC*H*), 4.87 (qd, *J* = 6.3, 2.3 Hz, 1H, C*H*CH_3), 4.69 (dd, *J* = 13.7, 2.6 Hz, 1H, CH_A(N)), 4.52 (dd, *J* = 13.6, 1.0 Hz, 1H, CH_B(N)), 2.57 – 2.51 (m, 2H, CH_2CH_2), 2.38 (s, 3H, CH_3Ts), 1.65 (d, *J* = 6.3 Hz, 3H, CH_3CH), 1.60 – 1.49 (m, 2H, CH_2CH_2), 1.34 – 1.24 (m, 4H), 0.87 (t, *J* = 7.0 Hz, 3H, CH_3CH_2). **^{13}C NMR** (126 MHz, $CDCl_3$) δ 143.4, 142.8, 138.9, 134.9, 134.6, 129.7, 128.0, 127.5, 122.2, 122.0, 61.7, 53.7, 35.7, 31.4, 31.2, 23.8, 22.4, 21.48, 13.9. **IR** (ν_{max}/cm^{-1}) 3301 (w), 2929 (m), 2836 (m), 1599 (m), 1451 (m), 1347 (m), 1164 (s), 1097 (m), 1019 (s), 665 (m). **HRMS** (ESI-OTF) *m/z* calcd for $C_{21}H_{27}NO_2SNa$ $[M + Na]^+$ 380.1160 found 380.1671.

1-Methyl-2-tosyl-5-(trimethylsilyl)isoindoline (2f)

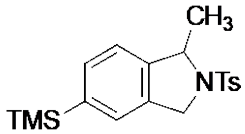

The product was obtained as a yellow solid (11 mg, 55%), after flash column chromatography (Et_2O/pentane 2:8) following the general procedure using (*Z*)-4-methyl-*N*-(prop-2-yn-1-yl)-*N*-(6-(trimethylsilyl)hex-3-en-5-yn-2-yl)benzenesulfonamide **1f** (20 mg, 0.055 mmol) in DCE (0.5 mL), [Rh(COD)Cl_2 (1.37 mg, 0.002mmol), (±)-BINAP (4.1 mg, 0.006 mmol) and $AgSbF_6$ (3.7 mg, 0.01 mmol) in DCE (0.5 mL), the solution was stirred at room temperature for 12 h. **Mp:** 251−255 °C, R_f (EtOAc/hexane 1:9) = 0.7. **^{1}H NMR** (400 MHz, $CDCl_3$) δ 7.79 – 7.69 (m, 2H, 2ArC*H*), 7.39 (dd, *J* = 7.6, 1.0 Hz, 1H, ArC*H*), 7.26 – 7.24 (m, 2H, 2ArC*H*), 7.11 – 7.07 (m, 1H, ArC*H*), 4.90 (dt, *J* = 6.5, 3.3 Hz, 1H, C*H*-CH_3(NTs)), 4.73 (dd, *J* = 13.7, 2.7 Hz, 1H, CH_A(N)), 4.55 (dd, *J* = 13.7, 1.0 Hz, 1H, CH_B(N)), 2.38 (s, 3H, CH_3(Ts)), 1.68 (d, *J* = 6.4 Hz, 3H, CH_3(CH)), 0.23 (s, 9H,CH_3-TMS). **^{13}C NMR** (101 MHz, $CDCl_3$) δ 144.6, 143.4, 141.4, 135.7, 135.5, 133.8, 130.8, 128.7, 128.3, 122.8, 63.0, 54.9, 24.8, 22.6, 1.1. **IR** (ν_{max}/cm^{-1}) 3339 (w), 2970 (s), 2932 (m), 2883 (m), 1466 (m), 1408 (s), 1379 (s), 1306 (s), 1160 (s), 1107 (s), 950 (s), 816 (s). **HRMS** (ESI-OTF) *m/z* calcd for $C_{19}H_{25}NO_2SSiNa$ [M + Na]$^+$ 382.1268 found 382.1268.

4-Ethyl-1-methyl-5-phenyl-2-tosylisoindoline (2g)

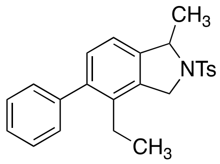

The product was obtained as a white solid (16.1mg, 81%) after flash column chromatography (Et_2O/pentane 2:8) following the general procedure using Z)-4-methyl-N-(pent-2-yn-1-yl)-N-(6-phenylhex-3-en-5-yn-2-yl)benzenesulfonamide **1g** (20 mg, 0.051 mmol) in DCE (0.5 mL),

$[Rh(COD)Cl]_2$ (1.26 mg, 0.002mmol) , (±)-BINAP (3.81 mg, 0.006 mmol) and $AgSbF_6$ (3.49 mg, 0.010 mmol) in DCE (0.5 mL), the solution was stirred at room temperature for 12 h. **Mp:** 125−127 °C, R_f (EtOAc/hexane 1.5:8.5) = 0.43. **^{1}H NMR** (500 MHz, $CDCl_3$) δ 7.79 (d, *J* = 8.3 Hz, 2H, 2CH_{Ar}), 7.41 – 7.32 (m, 3H, 3CH_{Ar}), 7.30 (d, *J* = 7.8 Hz, 1H, 1CH_{Ar}), 7.24 – 7.20 (m, 2H, 2CH_{Ar}), 7.10 (d, *J* = 7.8 Hz, 1H, 1CH_{Ar}), 6.96 (dd, *J* = 7.8, 0.9 Hz, 1H, 1CH_{Ar}), 4.94 (qd, *J* = 6.3, 2.5 Hz, 1H, C*H*(CH_3)), 4.79 (dd, *J* = 13.7, 2.7 Hz, 1H, CH_A(N)), 4.59 (d, *J* = 13.7 Hz, 1H, CH_B(N)), 2.44 (dd, *J* = 7.6, 4.1 Hz, 2H, CH_2(Ar) and (CH_3)), 2.40 (s, 3H, CH_3(Ts)), 1.71 (d, *J* = 6.4 Hz, 3H, CH_3(CH)), 0.94 (t, *J* = 7.6 Hz, 3H, CH_3(CH_2)). **^{13}C NMR** (126 MHz, $CDCl_3$) δ 143.5, 141.4, 141.0, 140.9, 136.3, 134.5, 133.9, 130.2, 129.8, 129.1, 128.0, 127.5, 127.0, 119.4, 62.0, 53.1, 23.8, 23.6, 21.5, 14.3. **IR** (ν_{max}/cm^{-1}) 2963 (w), 2866 (w), 1596 (s), 1444 (s), 1375 (s), 1340 (s), 1159 (s), 1092 (s), 834 (s), 766 (s), 656 (s), 547 (s). **HRMS** (ESI-OTF) *m/z* calcd for $C_{24}H_{26}NO_2S$ [M + H]$^+$ 392.1684, found 392.1694.

1-Methyl-2-((4-nitrophenyl)sulfonyl)-5-phenylisoindoline (2h)

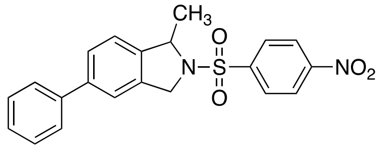

The product was obtained as a white solid (26 mg, 81%) after flash column chromatography (Et_2O/pentane 2:8) following the general procedure using (*Z*)-4-methyl-*N*-(6-phenylhex-3-en-5-yn-2-yl-3-*d*)-*N*-(prop-2-yn-1-yl)benzenesulfonamide **1h** (33 mg, 0.090 mmol) in DCE (1.0 mL), $[Rh(COD)Cl]_2$ (2.2 mg, 0.004mmol) , (±)-BINAP (6.76 mg, 0.010 mmol) and $AgSbF_6$ (6.21 mg, 0.018 mmol) in DCE (0.5 mL), the solution was stirred at room temperature for 12 h. **Mp:** 71−73 °C, R_f (EtOAc/hexane 1:9) = 0.39. **^{1}H NMR** (400 MHz, $CDCl_3$) δ 8.35 (d, *J* = 8.9 Hz, 2H, 2ArC*H*), 8.07 (d, *J* = 8.9 Hz, 2H, 2ArC*H*), 7.53 – 7.46 (m, 3H, 3ArC*H*), 7.45 – 7.39 (m, 2H, 2ArC*H*), 7.38 – 7.31 (m, 2H, 2ArC*H*), 7.18 (d, *J* = 8.0 Hz, 1H, 2ArC*H*), 5.03 (qd, *J* = 6.4, 2.4 Hz, 1H, C*H*(CH_3)), 4.83 (dd, *J* = 13.9, 2.6 Hz, 1H, CH_A(N)), 4.66 (dd, *J* = 13.8,

1.0 Hz, 1H, CH_B(N)), 1.70 (d, J = 6.4 Hz, 3H, CH_3(CH)).**^{13}C NMR** (101 MHz, $CDCl_3$) δ 150.1, 143.7, 141.7, 140.3, 140.0, 134.9, 128.8, 128.5, 127.6, 127.3, 127.1, 124.4, 122.7, 121.2, 62.2, 53.7, 23.8. **IR** (ν_{max}/cm^{-1}) 3102 (w), 2867 (w), 1607 (s), 1527 (s), 1483 (s), 1400 (m), 1347 (s), 1312 (m), 1163 (s), 1092 (m), 1101 (m), 855 (m), 760 (s), 745 (m), 733 (m), 686 (m), 638 (m), 619 (s). **HRMS** (ESI-OTF) *m/z* calcd for $C_{21}H_{19}N_2O_4S$ $[M + H]^+$ 395.1070, found 395.1081.

Synthesis of 5-phenyl-1,3-dihydroisobenzofuran from (*Z*)-(5-(prop-2-yn-1-yloxy)pent-3-en-1-yn-1-yl)benzene (2k)

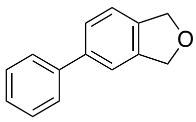

The product was obtained as a colourless liquid (13 mg, 65%) after flash column chromatography (Et_2O/pentane 1:9) following the general procedure using (*Z*)-(5-(prop-2-yn-1-yloxy)hex-3-en-1-yn-1-yl)benzene **1k** (20 mg, 0.051 mmol) in DCE (0.5 mL), $[Rh(COD)Cl]_2$ (2.34 mg, 0.004 mmol) , (±)-BINAP (7.1 mg, 011.42 mmol) and $AgSbF_6$ (6.5 mg, 0.019 mmol) in DCE (0.5 mL), the solution was stirred at room temperature for 1 h. R_f (EtOAc/hexane 8:2) = 0.76. **^{1}H NMR** (500 MHz, $CDCl_3$) δ 7.58 – 7.56 (m, 2H,), 7.50 – 7.48 (d, J = 10, 1H), 7.45 – 7.42 (m, 3H), 7.37 – 7.31 (m, 1H), 7.30 (d, J = 8.5, 1H), 5.17 – 5.16 (m, 4H).**^{13}C NMR** (126 MHz, $CDCl_3$) δ 141.0, 140.8, 139.9, 138.2, 128.8, 127.3, 127.2, 126.5, 121.2, 119.7, 73.5, 73.4. NMR data matches literature values.[17]

1-Methyl-5-phenyl-1,3-dihydroisobenzofuran from(*Z*)-(5-(prop-2-yn-1-yloxy)hex-3-en-1-yn-1-yl)benzene(2l)

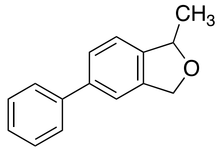

The product was obtained as a colourless liquid (13.1 mg, 66%) after flash column chromatography (Et_2O/pentane 1:9) following the general procedure using (*Z*)-(5-(prop-2-yn-1-yloxy)hex-3-en-1-yn-1-yl)benzene **1l** (20 mg, 0.051 mmol) in DCE (0.5 mL), $[Rh(COD)Cl]_2$ (2.34 mg, 0.004 mmol), (±)-BINAP (7.1 mg, 011.42 mmol) and $AgSbF_6$ (6.5 mg, 0.019 mmol) in DCE (1.0 mL), the solution was stirred at room temperature for 12 h. R*f* (EtOAc/hexane 8:2) = 0.76. **^{1}H NMR** (500 MHz, $CDCl_3$) δ 7.60 – 7.55 (m, 2H, 2ArC*H*), 7.50 (d, *J* = 7.8 Hz, 1H, ArC*H*), 7.47 – 7.40 (m, 3H, 3ArC*H*), 7.39 – 7.32 (m, 1H, ArC*H*), 7.23 (d, *J* = 7.8 Hz, 1H, ArC*H*), 5.41 – 5.26 (m, 1H, CH_A(O)), 5.19 (dd, *J* = 12.1, 2.4 Hz, 1H, CH_B(O)), 5.10 (ddt, *J* = 12.0, 1.8, 0.9 Hz, 1H, (CH_3)C*H*), 1.55 (d, *J* = 0.8 Hz, 3H, CH_3). **^{13}C NMR** (126 MHz, $CDCl_3$) δ 142.6, 141.0, 140.9, 140.0, 128.7, 127.3, 127.2, 126.6, 121.2, 119.7, 79.8, 72.2, 21.7. **IR** (ν_{max}/cm^{-1}) 3058 (w), 2969 (w), 2853 (w), 1762 (m), 1574 (s), 1481 (s), 1370 (s), 1128 (s), 1070 (s), 760 (s), 697 (s), 525 (s). **HRMS** (ESI-OTF) *m/z* calcd for $C_{15}H_{15}O$ $[M + H]^+$ 211.1123, found 211.1119.

5-(4-Fluorophenyl)-1-methyl-1,3-dihydroisobenzofuran (2m)

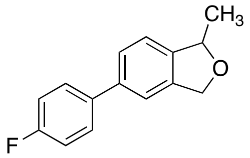

The product was obtained as a yellow liquid (13.2 mg, 66%) after flash column chromatography (Et_2O/pentane 1:9) following the general procedure using (*Z*)-1-fluoro-4-(5-(prop-2-yn-1-yloxy)hex-3-en-1-yn-1-yl)benzene **1m** (20 mg, 0.087 mmol) in DCE (0.5 mL), $[Rh(COD)Cl]_2$ (2.16 mg, 0.004 mmol), (±)-BINAP (6.5 mg, 0.01 mmol) and $AgSbF_6$ (6.01 mg, 0.017 mmol), the solution was stirred at room temperature for 12 h. R*f* (EtOAc/hexane 0.5:9.5) = 0.31. **^{1}H NMR** (500 MHz, $CDCl_3$) δ 7.53 (dd, *J* = 8.8, 5.3 Hz, 2H, 2CH_{Ar}), 7.46 – 7.44 (m, 1H, 1CH_{Ar}), 7.38 (s, 1H, 1CH_{Ar}), 7.23 (d, *J* = 7.9 Hz, 1H, 1CH_{Ar}), 7.17 – 7.09 (m, 2H,

2CH_{Ar}), 5.41 – 5.31 (m, 1H, C*H*(CH_3)), 5.18 (d, *J* = 2.4 Hz, 1H, C*H*(O)), 5.10 (d, *J* = 12.2 Hz, 1H, C*H*(O)), 1.54 (d, *J* = 6.3 Hz, 3H, CH_3(CH)). **^{13}C NMR** (126 MHz, $CDCl_3$) δ 162.5 (d, J_{CF} = 246.4 Hz), 142.6, 140.2, 139.9, 137.2, 128.7 (d, J_{CF} = 8.0 Hz), 126.5, 121.3, 119.6, 115.6 (d, J_{CF} = 21.4 Hz), 79.8, 72.2, 21.7. **IR** (ν_{max}/cm^{-1}) 3361 (w), 2936 (m), 1760 (m), 1601 (m), 1516 (m), 1488 (s), 1346 (m), 1022 (s), 824 (s), 529 (m). **HRMS** (ESI-OTF) *m/z* calcd for $C_{15}H_{13}OFNa$ [M + Na]$^+$ 229.1029, found 229.1036.

5-(4-Methoxyphenyl)-1-methyl-1,3-dihydroisobenzofuran (2n)

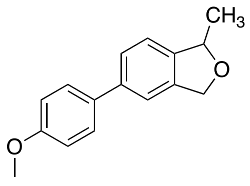

The product was obtained as a white solid (14 mg, 70%) after flash column chromatography (EtOAc/hexane 1:9) following the general procedure using (*Z*)-1-methoxy-4-(5-(prop-2-yn-1-yloxy)hex-3-en-1-yn-1-yl)benzene **1n** (20 mg, 0.08mmol) in DCE (0.5 mL), [Rh(COD)Cl]$_2$ (2.05 mg, 0.004mmol), (±)-BINAP (6.21 mg, 0.009 mmol) and $AgSbF_6$ (5.71 mg, 0.016 mmol) in DCE (1.0 mL), the solution was stirred at room temperature for 12 h. **Mp:** 94–96 °C, R$_f$ (EtOAc/hexane 0.5:9.5) = 0.58. **^{1}H NMR** (400 MHz, $CDCl_3$) δ 7.50 (d, *J* = 8.8 Hz, 2H, 2ArC*H*), 7.45 (d, *J* = 7.8 Hz, 1H ArC*H*), 7.38 (s, 1H, ArC*H*), 7.20 (d, *J* = 7.8 Hz, 1H ArC*H*), 6.97 (d, *J* = 8.8 Hz, 2H, 2ArC*H*), 5.35 (qd, *J* = 6.3, 3.1 Hz, 1H, C*H*(CH_3)), 5.18 (dd, *J* = 12.2, 2.4 Hz, 1H, CH_A(O)), 5.08 (dd, *J* = 12.3, 2.0 Hz, 1H, CH_B(O)), 3.85 (s, 3H, OCH_3), 1.53 (d, *J* = 6.4 Hz, 3H, CH_3(CH)). **^{13}C NMR** (101 MHz, $CDCl_3$) δ 159.2, 142.0, 140.5, 140.0, 133.6, 128.2, 126.1, 121.1, 119.2, 114.2, 79.8, 72.2, 55.3, 21.7. **IR** (ν_{max}/cm^{-1}) 3476 (w), 2967 (m), 2921 (m), 2839 (m), 1067 (m), 1520 (m), 1489 (m), 1373 (s), 1298 (m), 1184 (m), 1070 (s), 852 (s). **HRMS** (ESI-OTF) *m/z* calcd for $C_{16}H_{17}O_2$ [M + H]$^+$ 241.1223 found 241.1222.

1-Methyl-5-pentyl-1,3-dihydroisobenzofuran (2o)

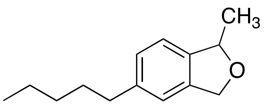

The product was obtained as a yellow liquid (15 mg, 75%) after flash column chromatography (Et_2O/pentane 1:9) following the general procedure using (*Z*)-2-(prop-2-yn-1-yloxy)undec-3-en-5-yne **1o** (20 mg, 0.091 mmol) in DCE (0.5 mL), $[Rh(COD)Cl]_2$ (2.21 mg, 0.004 mmol), (±)-BINAP (6.8 mg, 0.011 mmol) and $AgSbF_6$ (6.2 mg, 0.018 mmol) in DCE (0.5 mL), the solution was stirred at room temperature for 12 h. R_f (EtOAc/hexane 1:9) = 0.89. **^{1}H NMR (**500 MHz, $CDCl_3$**)** δ 7.94 (d, *J* = 7 1H, C*H*Ar), 7.05 (d, *J* = 7.5 Hz, 1H, C*H*Ar), 7.03 (s, 1H, C*H*Ar), 5.33 – 5.25 (m, 1H, C*H*CH_3), 5.10 (dd, *J* = 12.1, 2.5 Hz, 1H, CH_A(O)), 5.05 – 4.98 (dt, *J* = 12, 1 Hz, 1H, CH_B(O)), 2.64 – 2.58 (m, 2H, CH_2), 1.60 (s, 4H, 2CH_2), 1.48 (d, *J* = 6.3 Hz, 2H, , CH_3CH), 1.37 – 1.21 (m, 2H), 0.92 – 0.87 (m, 4H, , 2CH_2), .**^{13}C NMR (**125 MHz, $CDCl_3$**)** δ 142.3, 140.8, 139.4, 127.5, 120.7, 120.6, 79.8, 72.1, 35.8, 31.5, 31.4, 22.5, 21.8, 14.0, 14.0. **IR** (ν_{max}/cm^{-1}) 3340 (w), 2970 (s), 2883 (m), 1466 (m) 1408 (s), 1341 (s), 1305 (s), 1160 (s), 1107 (s), 950 (s), 816 (s), 637 (w), 487 (s). **HRMS** (ESI-OTF) *m/z* calcd for $C_{14}H_{20}ONa$ $[M + Na]^+$ 227.1412 found 227.1420.

5-Phenyl-1-propyl-1,3-dihydroisobenzofuran (2p)

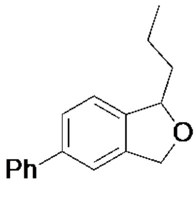

The product was obtained as a colourless liquid (13.9 mg, 70%) after flash column chromatography (Et_2O/pentane 0.6:9.4) following the general procedure using (*Z*)-(5-(prop-2-yn-1-yloxy)oct-3-en-1-yn-1-yl)benzene **1p** (20 mg, 0.08 mmol) in DCE (0.5 mL), $[Rh(COD)Cl]_2$ (2.07 mg, 0.004 mmol), (±)-BINAP (6.27 mg, 0.01 mmol) and $AgSbF_6$ (5.7

mg, 0.016 mmol) in DCE (0.5 mL), the solution was stirred at room temperature for 9 h. R*f* (EtOAc/hexane 0.5:9.5) = 0.44. ^{1}**H NMR** (500 MHz, $CDCl_3$) δ 7.60 – 7.54 (m, 2H, 2ArC*H*), 7.49 (ddd, *J* = 7.9, 1.7, 0.9 Hz, 1H, ArC*H*), 7.47 – 7.40 (m, 3H, 3ArC*H*), 7.38 – 7.29 (m, 1H, ArC*H*), 7.23 (d, *J* = 7.8 Hz, 1H, Ar), 5.30 – 5.26 (m, 1H, C*H*(propyl)), 5.17 (dd, *J* = 12.1, 2.4 Hz, 1H, CH_A(O)), 5.11 (d, *J* = 12.1 Hz, 1H, CH_B(O)), 1.92 – 1.77 ((m, 1H, CH_2), 1.77 – 1.66 (m, 1H, CH_2), 1.55 – 1.45 (m, 3H, CH_2), 0.99 (t, *J* = 7.4 Hz, 3H, CH_3). ^{13}**C NMR** (126 MHz, $CDCl_3$) δ 141.4, 141.0, 140.7, 140.2, 128.7, 127.2, 127.1, 126.4, 121.3, 119.6, 83.6, 72.4, 38.4, 18.4, 14.1. **IR** (ν_{max}/cm^{-1}) 3058 (m), 3031 (m), 2956 (m), 2869 (m), 1733 (m), 1600 (m), 1481 (m), 1349 (m), 1250 (m), 1064 (s), 830 (m), 759 (s), 696 (s), 521 (m). **HRMS** (ESI-OTF) *m/z* calcd for $C_{17}H_{18}ONa$ $[M + Na]^+$ 261.1255 found 261.1260.

1,7-Dimethyl-5-phenyl-1,3-dihydroisobenzofuran (2s)

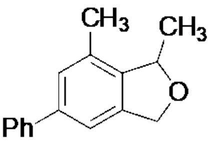

The product was obtained as a yellow liquid (13.9 mg, 70%) after flash column chromatography (Et_2O/pentane 0.5:9.5) following the general procedure using (*Z*)-(3-methyl-5-(prop-2-yn-1-yloxy)hex-3-en-1-yn-1-yl)benzene **1s** (20 mg, 0.08 mmol) in DCE (0.5 mL), $[Rh(COD)Cl]_2$ (2.2 mg, 0.004 mmol), (±)-BINAP (6.6 mg, 0.01 mmol) and $AgSbF_6$ (6.1 mg, 0.01 mmol) in DCE (0.5 mL), the solution was stirred at room temperature for 1.3 h. R*f* (EtOAc/hexane 1.5:85) = 0.66. ^{1}**H NMR** (400 MHz, $CDCl_3$) δ 7.60 – 7.52 (m, 2H, 2ArC*H*), 7.52 – 7.38 (m, 2H, 2ArC*H*), 7.38 – 7.29 (m, 1H, ArC*H*), 7.29 – 7.22 (m, 2H, 2ArC*H*), 5.49 – 5.37 (qd, *J* = 9.2, 4 HZ, 1H, C*H*CH_3), 5.22 (dd, *J* = 12.4, 2 Hz, 1H, CH_A(O)), 5.07 (dd, *J* = 12.3, 1.0 Hz, 1H, CH_B(O)), 2.36 (s, 3H, CH_3Ar), 1.52 (d, *J* = 6.3 Hz, 3H, CH_3CH). ^{13}**C NMR** (101 MHz, $CDCl_3$) δ 141.2, 141.1, 141.0, 139.8, 131.6, 128.7, 128.1, 127.2, 127.2, 117.2, 79.9, 72.1, 21.0, 18.86. **IR** (ν_{max}/cm^{-1}) 3060 (w), 3030 (w), 2864 (w), 1734 1663 (m), 1598 (m), 1490

(m), 1378 (m), 1068 (s), 1014 (m), 871 (s), 763 (s), 698 (s). **HRMS** (ESI-OTF) *m/z* calcd for $C_{16}H_{17}O$ [M + H]$^+$ 225.1274 found 225.1274.

4,5-Diphenyl-1,3-dihydroisobenzofuran (2t)

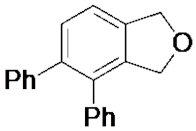

The product was obtained as a yellow solid (16.2 mg, 81%), after flash column chromatography (Et_2O/pentane 0.6:9.4) following the general procedure using (*Z*)-(3-((5-phenylpent-2-en-4-yn-1-yl)oxy)prop-1-yn-1-yl)benzene **1t** (20 mg, 0.07 mmol) in DCE (0.5), $[Rh(COD)Cl]_2$ (1.81 mg, 0.003 mmol), (±)-BINAP (5.4 mg, 0.008 mmol) and $AgSbF_6$ (5.04 mg, 0.014 mmol) in DCE (0.5 mL), the solution was stirred at room temperature for 4.5 h. R_f (EtOAc/hexane 1.5:85) = 0.63. **^{1}H NMR** (500 MHz, $CDCl_3$) δ 7.41 – 7.36 (m, 1H, ArC*H*), 7.28 (dt, *J* = 7.8, 0.9 Hz, 1H, ArC*H*), 7.25 – 7.14 (m, 6H, 6ArC*H*), 7.12 – 7.08 (m, 2H, 2ArC*H*), 7.07 – 7.03 (m, 2H, 2ArC*H*), 5.23 (s,1H, OCH_2), 5.00 (s, 1H, OCH_2). **^{13}C NMR** (126 MHz, $CDCl_3$) δ 141.0, 140.3, 139.2, 138.7, 134.7, 130.2, 130.1, 129.6, 128.2, 127.9, 127.0, 126.6, 120.1, 74.2, 73.9. NMR data matches literature values.[18]

Dimethyl 5-phenyl-1,3-dihydro-2*H*-indene-2,2-dicarboxylate (2u)

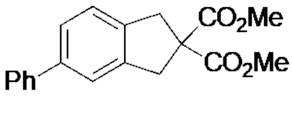

The product was obtained as a pale-yellow oil (15.4 mg, 77%) after flash column chromatography (Et_2O/pentane 0.4:9.6) following the general procedure using dimethyl (*Z*)-2-(5-phenylpent-2-en-4-yn-1-yl)-2-(prop-2-yn-1-yl)malonate **1u** (20 mg, 0.06 mmol) in DCE (0.5 mL), $[Rh(COD)Cl]_2$ (1.5 mg, 0.003 mmol), (±)-BINAP (4.8 mg, 0.007 mmol) and $AgSbF_6$ (4.4 mg, 0.012mmol) in DCE (0.5 mL), the solution was stirred at room temperature for 1.3 h.

R_f (EtOAc/hexane 1.5:8.5) = 0.5. **^{1}H NMR** (400 MHz,$CDCl_3$) δ 7.57 – 7.52 (m, 2H, ArC*H*), 7.45 – 7.38 (m, 4H, 4ArC*H*), 7.35 – 7.27 (m, 1H, 1ArC*H*), 7.28 – 7.24 (m, 1H, 1ArC*H*), 3.76 (s, 6H, OCH_3), 3.66 (s, 2H, CH_2), 3.64 (s, 2H, CH_2). **^{13}C NMR** (101 MHz, $CDCl_3$) δ 172.0, 141.2, 140.5, 140.4, 139.0, 128.7, 127.1, 127.1, 126.2, 124.4, 123.0, 60.4, 53.0, 40.6, 40.3. NMR data matches literature values.[17]

5. Deuterium isotope labelling Study:

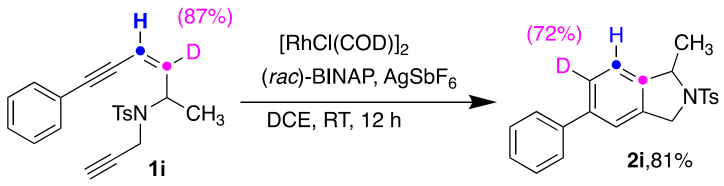

1-Methyl-5-phenyl-2-tosylisoindoline-6-*d* (2i)

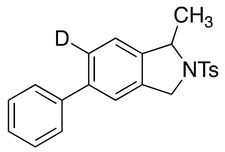

The product was obtained as a white solid (26 mg, 81%) after flash column chromatography (Et_2O/pentane 2:8) following the general procedure using (*Z*)-4-methyl-*N*-(6-phenylhex-3-en-5-yn-2-yl-3-*d*)-*N*-(prop-2-yn-1-yl)benzenesulfonamide **1i** (33 mg, 0.090 mmol) in DCE (0.5 mL), $[Rh(COD)Cl]_2$ (2.2 mg, 0.004mmol) , (±)-BINAP (6.76 mg, 0.010 mmol) and $AgSbF_6$ (6.21 mg, 0.018 mmol) in DCE (1.0 mL), the solution was stirred at room temperature for 12 h. **Mp:** 126−128 °C, R_f (EtOAc/hexane 1:9) = 0.39. **^{1}H NMR** (400 MHz, $CDCl_3$) δ 7.77 (d, *J* = 8.3 Hz, 2H, 2CH_{Ar}), 7.54 – 7.46 (m, 2H, 2CH_{Ar}), 7.41 (t, *J* = 7.5 Hz, 2H, 2CH_{Ar}), 7.36 – 7.31 (m, 2H, 2CH_{Ar}), 7.29 (d, *J* = 8.0 Hz, 2H, 2CH_{Ar}), 7.16 (s, 1H, 1CH_{Ar}), 4.96 (qd, *J* = 6.4, 2.3 Hz, 1H, C*H*(CH_3)), 4.78 (dd, *J* = 14.2, 2.7 Hz, 1H, CH_A(N)), 4.61 (d, *J* = 13.8 Hz, 1H, CH_B(N)), 2.38 (s, 3H, CH_3(Ts)), 1.71 (d, *J* = 6.4 Hz, 3H, CH_3(CH)). **^{13}C NMR** (126 MHz, $CDCl_3$) δ

143.5, 141.1, 140.7, 140.5, 135.6, 134.5, 129.8, 128.8, 127.5, 127.5, 127.1, 122.5, 121.1, 61.7, 53.7, 23.8, 21.4. **IR** (ν_{max}/cm^{-1}) 2926 (s), 2865 (w), 1597 (m), 1344 (s), 1160 (s), 1305 (m), 1091 (s), 910 (w), 814 (m), 662 (m). **HRMS** (ESI-OTF) *m/z* calcd for $C_{22}H_{21}DNO_2S$ $[M + H]^+$ 365.1434, found 365.1446.

Stacked proton NMR spectra of non-deuterated and deuterated isondoline.

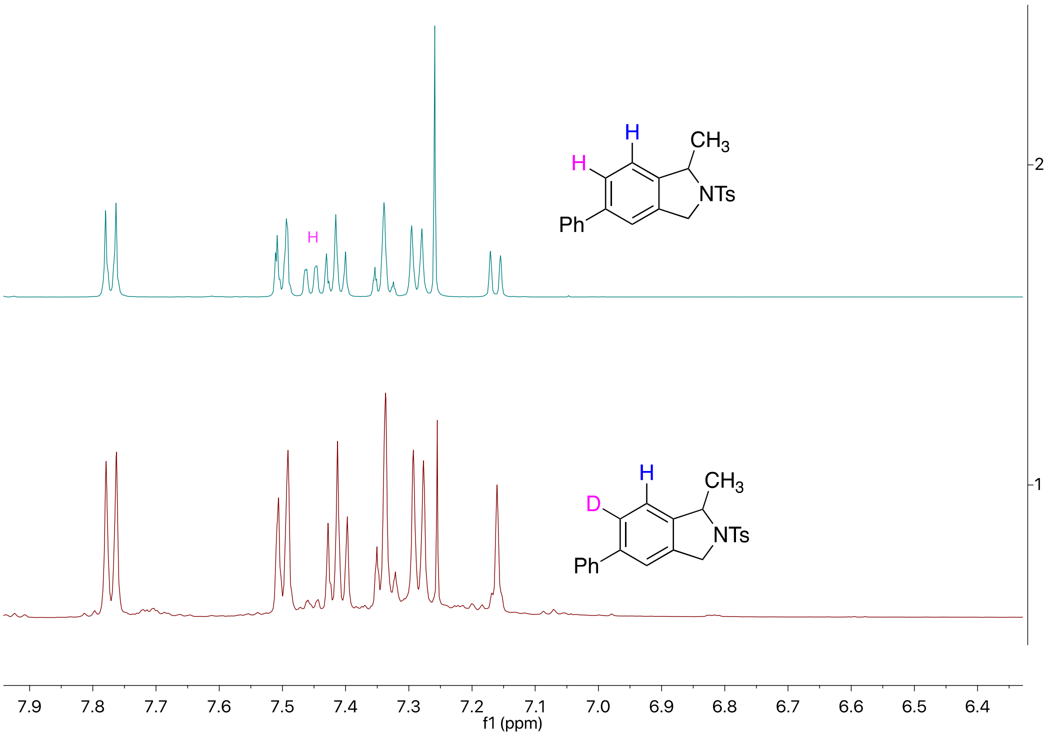

6. Mechanistic study:

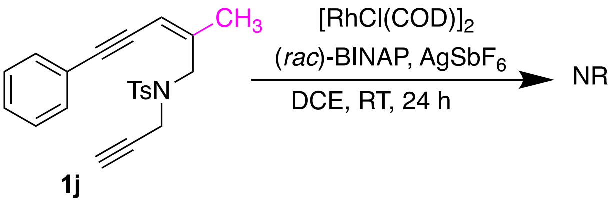

1j

We have conducted further investigation on the mechanism, where we have prepared substituted **1j** and treated this compound under Rh(I)-cycloaromatization conditions, however this reaction did not proceed, upon heating the starting material had decomposed.

7. Control experiment:

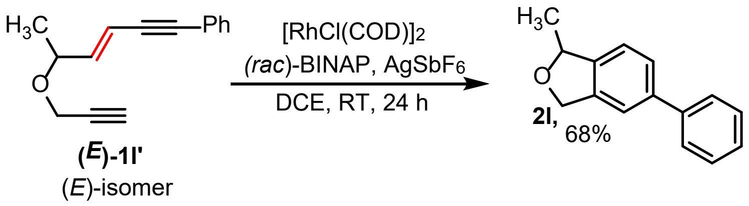

1-Methyl-5-phenyl-1,3-dihydroisobenzofuran (2l).

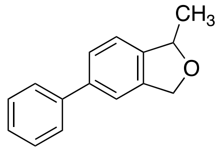

The product was obtained as a colourless liquid (13.5 mg, 68%) after flash column chromatography (Et_2O/pentane 1:9) following the general procedure using (*E*)-(5-(prop-2-yn-1-yloxy)hex-3-en-1-yn-1-yl)benzene (**1l'**) (20 mg, 0.0951 mmol) in DCE (0.5 mL), $[Rh(COD)Cl]_2$ (2.34 mg, 0.004 mmol) , (±)-BINAP (7.1 mg, 011.42 mmol) and $AgSbF_6$ (6.5 mg, 0.019 mmol) in DCE (O.5 mL). R_f (EtOAc/hexane 8:2) = 0.76. Spectroscopic data matched that reported above for **2l**.

8. Product modification reactions:

8.1. Oxidation of isoindoline to isoindolinone.

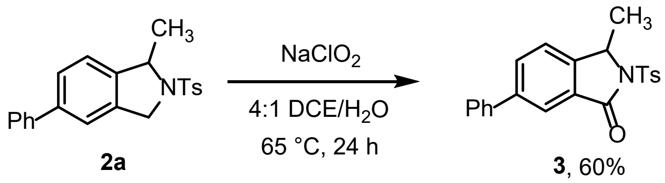

3-Methyl-6-phenyl-2-tosylisoindolin-1-one (3)

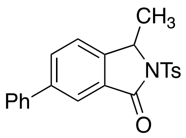

To a stirred solution of 1-methyl-5-phenyl-2-tosylisoindoline **2a** (1 mL) in DCE:H_2O 4:1 v/v at r.t was added $NaClO_2$ (14.9 mg, 0.16 mmol, 3 equiv), the reaction was mixture heated at 65 °C for 24 h. The crude reaction mixture was cooled to rt and quenched with sat. $Na_2S_2O_3$ (2 mL). The aqueous phase was extracted into DCM (2 × 2mL) and the combined extracts were washed with brine and dried (Na_2SO_4) to give **3** as a white solid (12.5 mg, 60%), **Mp:** 183-186 °C. **^{1}H NMR** (400 MHz, DCl_3) δ 8.05 (d, *J* = 8.3 Hz, 2H, 2ArC*H*), 7.97 (d, *J* = 1.8 Hz, 1H, ArC*H*), 7.86 (dd, *J* = 8.0, 1.8 Hz, 1H, ArC*H*), 7.58 – 7.52 (m, 2H, 2ArC*H*), 7.52 – 7.42 (m, 3H, 3ArC*H*), 7.38 – 7.36(m, 3ArC*H*), 5.34 (q, *J* = 6.5 Hz, 1H, C*H*CH_3), 2.42 (s, 3H, CH_3Ts), 1.82 (d, *J* = 6.5 Hz, 3H, CH_3CH). **^{13}C NMR** (101 MHz, $CDCl_3$) δ 166.3, 146.1, 145.0, 142.4, 139.4, 136.2, 133.1, 129.6, 129.6, 129.0, 128.2, 128.1, 127.1, 123.1, 122.8, 58.6, 21.6, 21.5. **IR (ν_{max}/cm^{-1}).** 2951 (w), 2867 (w), 1596 (w), 1453 (w), 1377 (m), 1344 (s), 1160 (s), 1089 (s), 887 (s), 814 (s), 692 (s), 615 (s). **HRMS** (ESI-OTF) *m/z* calcd for $C_{22}H_{20}O_3NS$ $[M + H]^+$ 378.1158 found 378.1158.

8.2. Oxidation of isobenzofuran to isobenzofuranone:

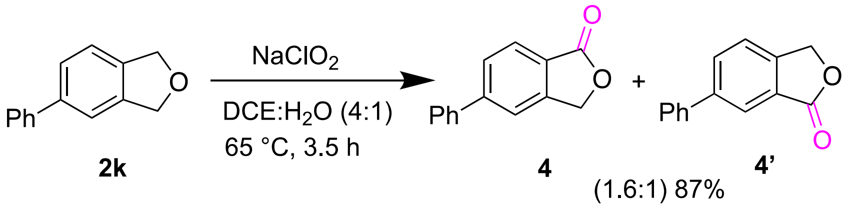

Isobenzofuranones (**4** and **4'**) were synthesized form isobenzofuran (**2k**) by following procedure for **3,** isolated white solid (23.1 mg, 87%). **^{1}H NMR** (400 MHz, $CDCl_3$) (**3'**) 7.98 (d, J = 8.0 Hz, 1H), 7.78 – 7.71 (m, 1H), 7.67 (s, 1H), 7.60 – 7.53 (m, 2H), 7.54 – 7.39 (m, 3H), 5.37 (s, 2H). **(3")** δ 8.13 (d, J = 1.7 Hz, 1H), 7.92 (dd, J = 8.0, 1.7 Hz, 1H), 7.65 – 7.58 (m, 2H), 7.57 (dd, J = 8.0, 0.8 Hz 1H), 7.54 – 7.39 (m, 3H), 5.37 (s, 2H). **^{13}C NMR** (101 MHz, $CDCl_3$) (**4 & 4'**) δ 171.0, 170.9, 147.5, 147.4, 145.2, 142.7, 139.7, 139.4, 133.2, 129.1, 129.1, 128.6, 128.5, 128.2, 127.5, 127.2, 126.5, 126.1, 124.5, 124.0, 122.4, 120.6, 69.6. proton NMR data matches the literature.[19]

9. Deprotection of the N-Ts group:

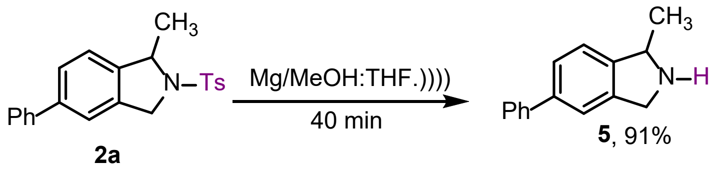

1-Methyl-5-phenylisoindoline (5)

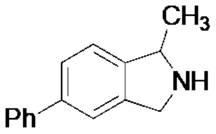

To an oven dried Schlenk flask was added 1-methyl-5-phenyl-2-tosylisoindoline **2b** (20 mg, 0.05 mmol) and powdered Mg (66.6 mg, 2.75 mmol) then the reaction flask was sealed with a rubber septum. The flask was filled with argon after evacuating under vacuum, then dry THF (0.36 mL) was added and the reaction mixture was swirled until the starting material appeared to be dissolved. Followed by addition of dry MeOH (1.1 mL) and resulting reaction mixture

was shaken vigorously before been sonicated for 45 min (sonication was paused roughly every 2 mins for the first 10 mins to shake the reaction flask). The reaction mixture was diluted with EtOAc and filtered through a pad of celite and the crude residue was concentrated under reduced pressure. The pale pink coloured liquid obtained was judged requisings to further pure purification (10.5 mg, 91.2%). (The product decomposed upon purification by column chromatography). **^{1}H NMR** (500 MHz, $CDCl_3$) δ 7.60 – 7.54 (m, 2H, 2ArC*H*), 7.47 – 7.40 (m, 4H, 4ArC*H*), 7.36 – 7.30 (m, 1H, ArC*H*), 7.24 (d, *J* = 1.1 Hz, 1H, ArC*H*), 4.48 (d, *J* = 6.7 Hz, 1H, C*H*CH_3), 4.30 (d, *J* = 14.2 Hz, 1H, CH_A(N)), 4.22 (d, *J* = 14.2 Hz, 1H, CH_B(N)), 1.47 (d, *J* = 6.5 Hz, 3H, CH_3CH). **^{13}C NMR (**126 MHz, $CDCl_3$) δ 145.4, 142.6, 141.3, 140.2, 128.7, 127.2, 127.1, 126.0, 122.0, 121.1, 58.8, 51.7, 21.5. (HRMS & IR data were not recorded due to decomposition of isoindoline product **5)**.

10. References

1 Garrais, S., Turkington, J. & Goldring, W. P. D. Synthesis of isomeric polyacetylenes based on natural hydroxy matricaria esters. *Tetrahedron* **65**, 8418-8427, doi:10.1016/j.tet.2009.07.082 (2009).

2 Ma, S., Lu, X. & Li, Z. A novel regio- and stereospecific hydrohalogenation reaction of 2-propynoic acid and its derivatives. *J. Org. Chem.* **57**, 709-713, doi:10.1021/jo00028a055 (1992).

3 Teske, J. & Plietker, B. A Redox-Neutral Fe-Catalyzed Cycloisomerization of Enyne Acetates. *ACS Catal.* **6**, 7148-7151, doi:10.1021/acscatal.6b02260 (2016).

4 Tseng, P.-Y. & Chuang, S.-C. Chemo-, Regio- and Stereoselective Tricyclohexylphosphine-Catalyzed [3+2] Cycloaddition of Enynes with [60]Fullerene Initiated by 1,4-Michael Addition: Synthesis of Cyclopenteno[60]fullerenes and their Electrochemical Properties. *Adv. Synth. Catal.* **355**, 2165-2171, doi:10.1002/adsc.201300255 (2013).

5 Akpınar, G. E., Kuş, M., Üçüncü, M., Karakuş, E. & Artok, L. Palladium-Catalyzed Alkoxycarbonylation of (Z)-2-En-4-yn Carbonates Leading to 2,3,5-Trienoates. *Org. Lett.* **13**, 748-751, doi:10.1021/ol102989q (2011).

6 Xie, H. *et al.* Total Synthesis of Polyene Natural Product Dihydroxerulin by Mild Organocatalyzed Dehydrogenation of Alcohols. *Chem. - Eur. J.* **18**, 2230-2234, doi:10.1002/chem.201103325 (2012).

7 Nicolaou, K. C. *et al.* Streamlined Total Synthesis of Uncialamycin and Its Application to the Synthesis of Designed Analogues for Biological Investigations. *J. Am. Chem. Soc.* **138**, 8235-8246, doi:10.1021/jacs.6b04339 (2016).

8 Zhu, S., Huang, X., Zhao, T. Q., Ma, T. & Jiang, H. Metal-catalyzed formation of 1,3-cyclohexadienes: a catalyst-dependent reaction. *Org. Biomol. Chem.* **13**, 1225-1233, doi:10.1039/c4ob01922a (2015).

9 Raminelli, C., Comasseto, J. V., Andrade, L. H. & Porto, A. L. M. Kinetic resolution of propargylic and allylic alcohols by Candida antarctica lipase (Novozyme 435). *Tetrahedron: Asymm.* **15**, 3117-3122, doi:10.1016/j.tetasy.2004.08.022 (2004).

10 Li, P.-F., Wang, H.-L. & Qu, J. 1,n-Rearrangement of Allylic Alcohols Promoted by Hot Water: Application to the Synthesis of Navenone B, a Polyene Natural Product. *J. Org. Chem.* **79**, 3955-3962, doi:10.1021/jo5004086 (2014).

11 Du, X., Chen, H. & Liu, Y. New Synthetic Approach for the Construction of Multisubstituted 2-Acyl Furans by the IBX-Mediated Cascade Oxidation/Cyclization ofcis-2-En-4-yn-1-ols (IBX=2-Iodoxybenzoic Acid). *Chemistry - A European Journal Chem. - Eur. J.* **14**, 9495-9498, doi:10.1002/chem.200801561 (2008).

12 Huang, S., Li, X., Lin, C. L., Guzei, I. A. & Tang, W. Rhodium-catalyzed 1,3-acyloxy migration and subsequent intramolecular [4+2] cycloaddition of vinylallene and unactivated alkyne. *Chem. Commun.,* **48**, 2204-2206, doi:10.1039/C2CC17406E (2012).

13 Subba Reddy, B. V. *et al.* Cooperative Multicatalytic System for the One-Pot Synthesis of Octahydrospiro-β-carbolines. *J. Org. Chem.* **80**, 8807-8814, doi:10.1021/acs.joc.5b01108 (2015).

14 Vinoth, P. *et al.* Palladium-Catalyzed Internal Nucleophile-Assisted Hydration-Olefin Insertion Cascade: Diastereoselective Synthesis of 2,3-Dihydro-1H-inden-1-ones. *Org. Lett.* **18**, 3442-3445, doi:10.1021/acs.orglett.6b01623 (2016).

15 Ferrer, S. & Echavarren, A. M. Total Synthesis of Repraesentin F and Configuration Reassignment by a Gold(I)-Catalyzed Cyclization Cascade. *Org. Lett.* **20**, 5784-5788, doi:10.1021/acs.orglett.8b02478 (2018).

16 Ez-Zoubir, M., Brown, J. A., Ratovelomanana-Vidal, V. & Michelet, V. Iridium-catalyzed hydroiodination of functionalized alkynes. *J. Organomet. Chem.* **696**, 433-441, doi:10.1016/j.jorganchem.2010.10.052 (2011).

17 Wang, Y.-H., Huang, S.-H., Lin, T.-C. & Tsai, F.-Y. Rhodium(I)/cationic 2,2′-bipyridyl-catalyzed [2+2+2] cycloaddition of α,ω-diynes with alkynes in water under air. *Tetrahedron* **66**, 7136-7141, doi:10.1016/j.tet.2010.06.088 (2010).

18 Yasukawa, N. *et al.* Highly-functionalized arene synthesis based on palladium on carbon-catalyzed aqueous dehydrogenation of cyclohexadienes and cyclohexenes. *Green Chem.* **20**, 1213-1217, doi:10.1039/c7gc03819d (2018).

19 Yamamoto, Y., Kinpara, K., Saigoku, T., Nishiyama, H. & Itoh, K. Synthesis of benzo-fused lactams and lactones via Ru(ii)-catalyzed cycloaddition of amide- and ester-tethered α,ω-diynes with terminal alkynes: electronic directing effect of internal conjugated carbonyl group. *Org. Biomol. Chem.* **2**, 1287-1294, doi:10.1039/B402649G (2004).

11. NMR spectra

^{1}H NMR, 500 MHz, $CDCl_3$

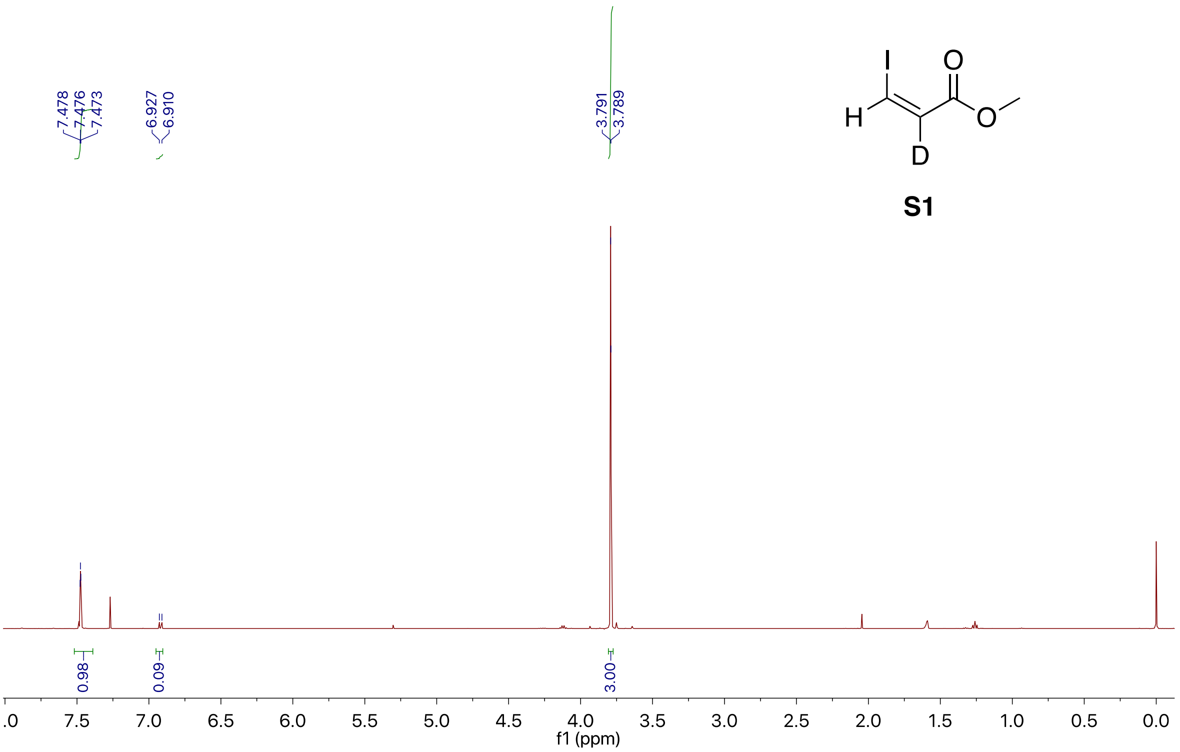

S1

^{13}C NMR, 126 MHz, $CDCl_3$

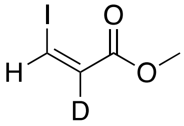

S1

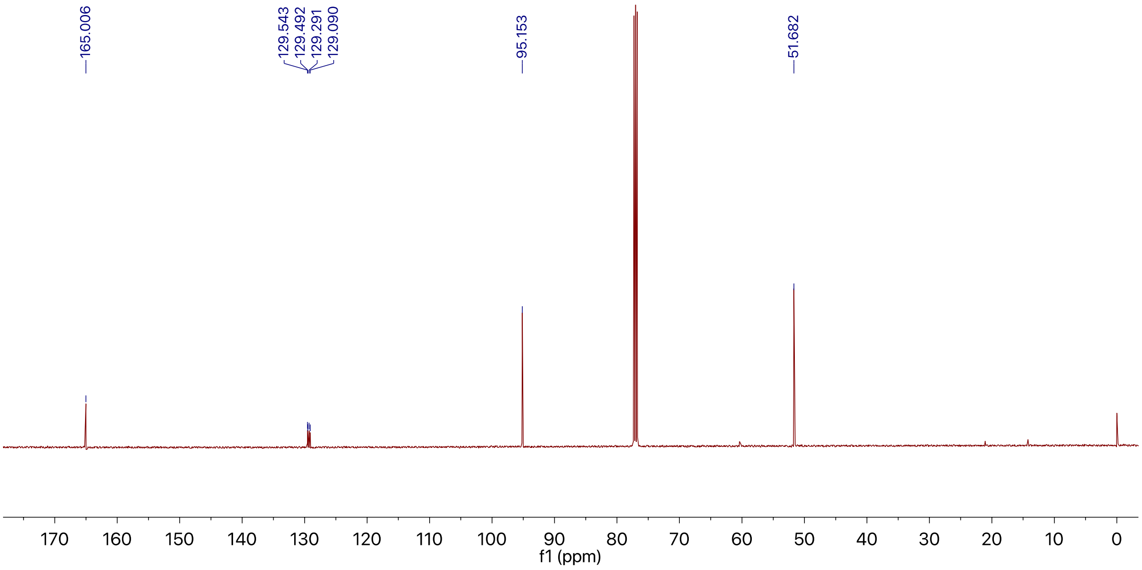

^{1}H NMR, 500 MHz, CDCl3

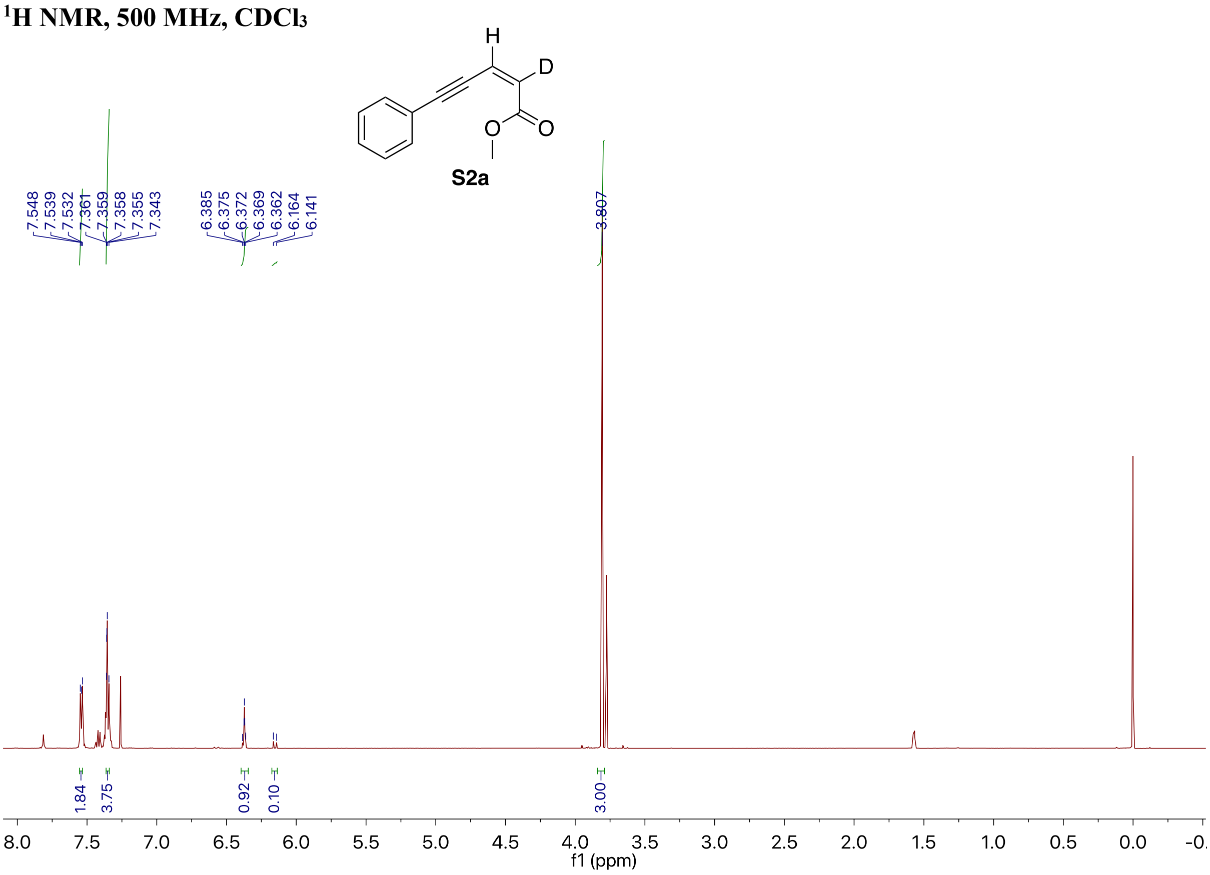

^{13}C NMR,126 MHz, CDCl3

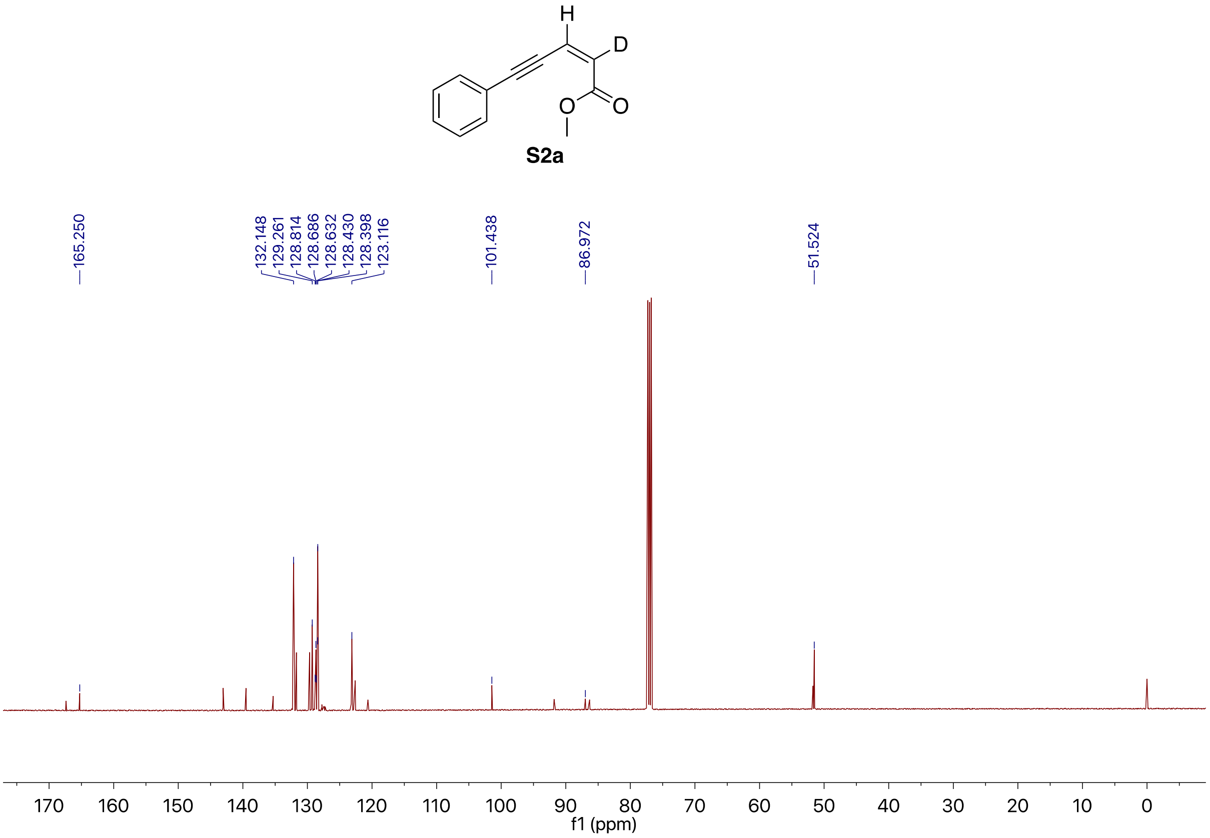

^{1}H NMR, 400 MHz, $CDCl_3$

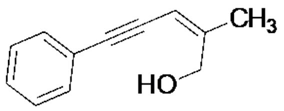

S2c

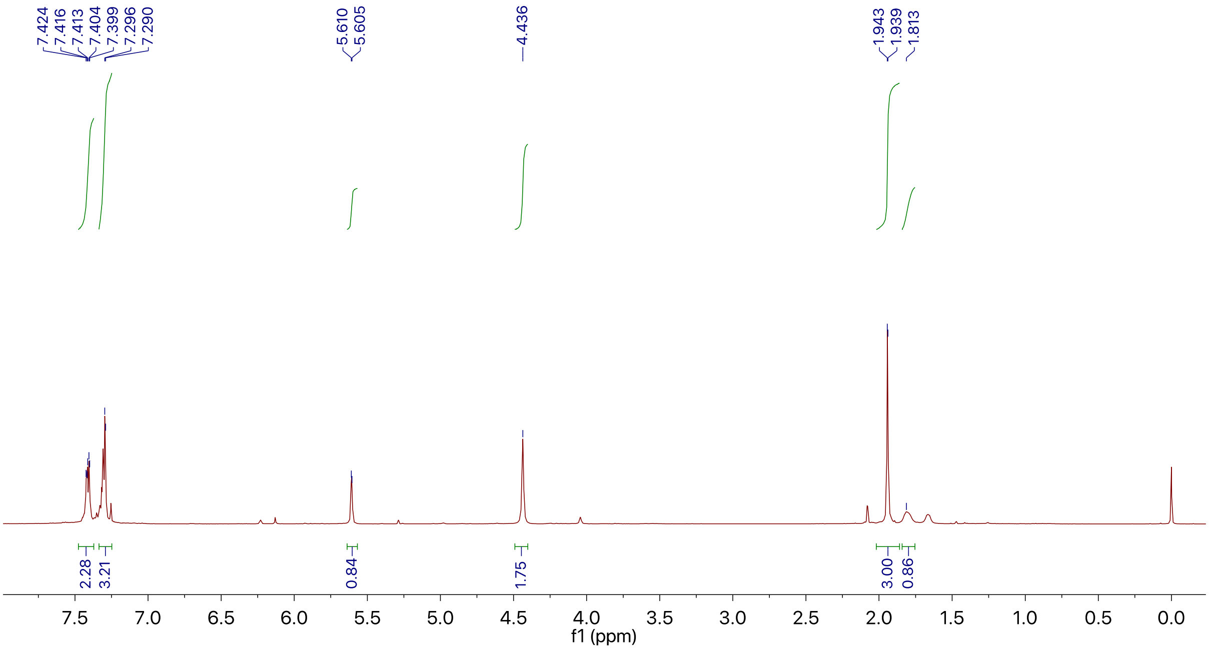

^{13}C NMR, 101 MHz, $CDCl_3$

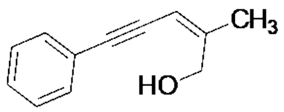

S2c

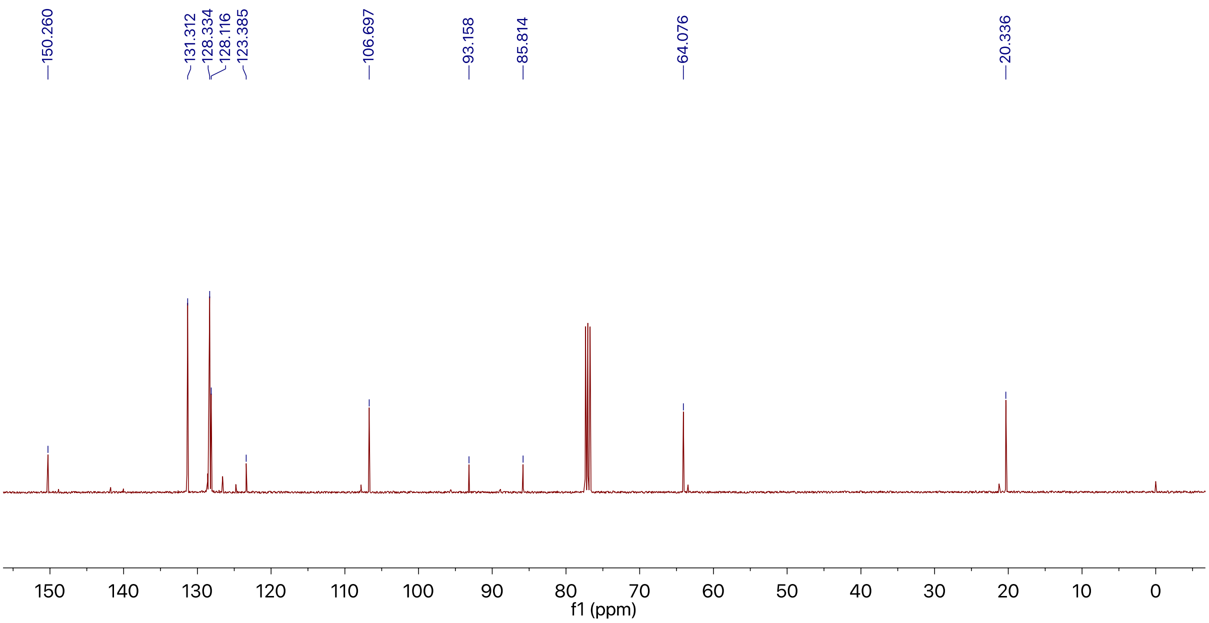

^{1}H NMR, 500 MHz, CDCl3

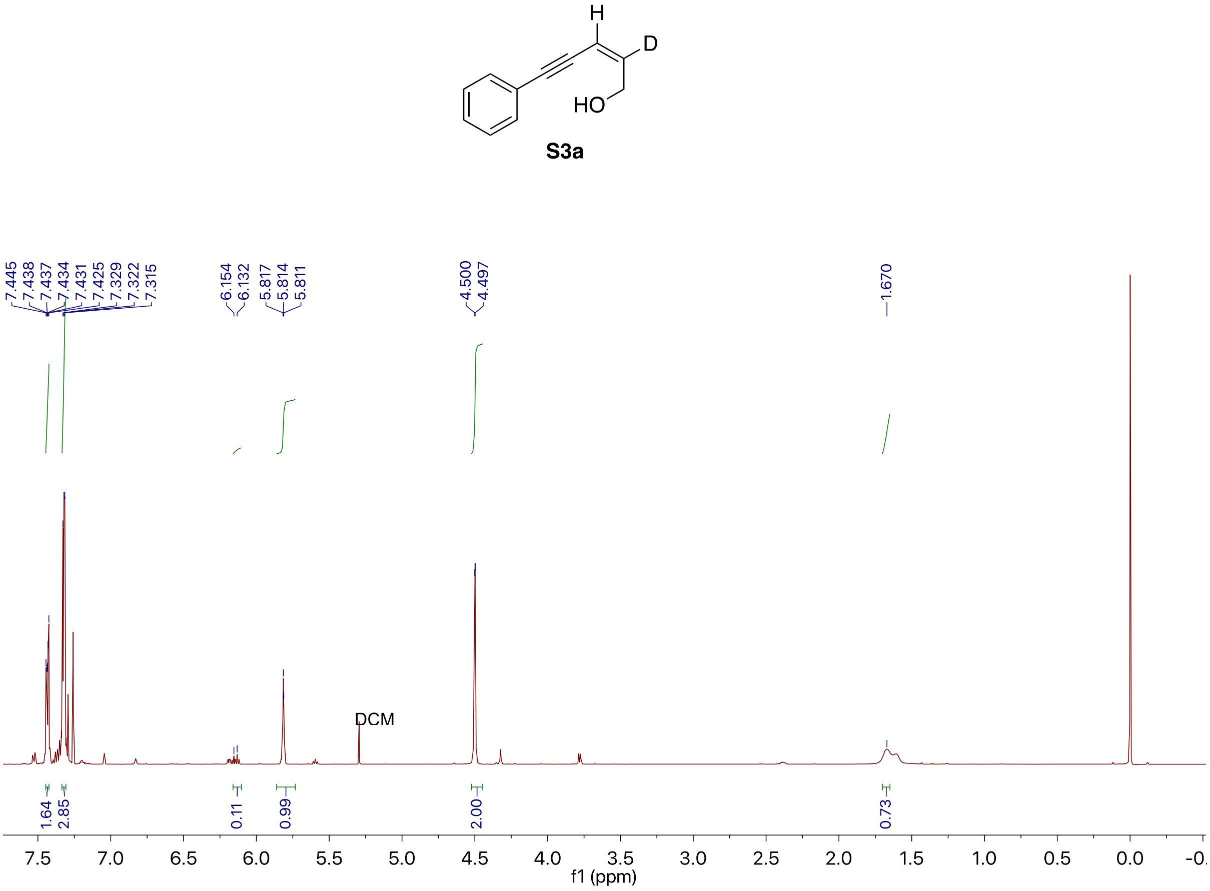

^{13}C NMR, 126 MHz, CDCl3

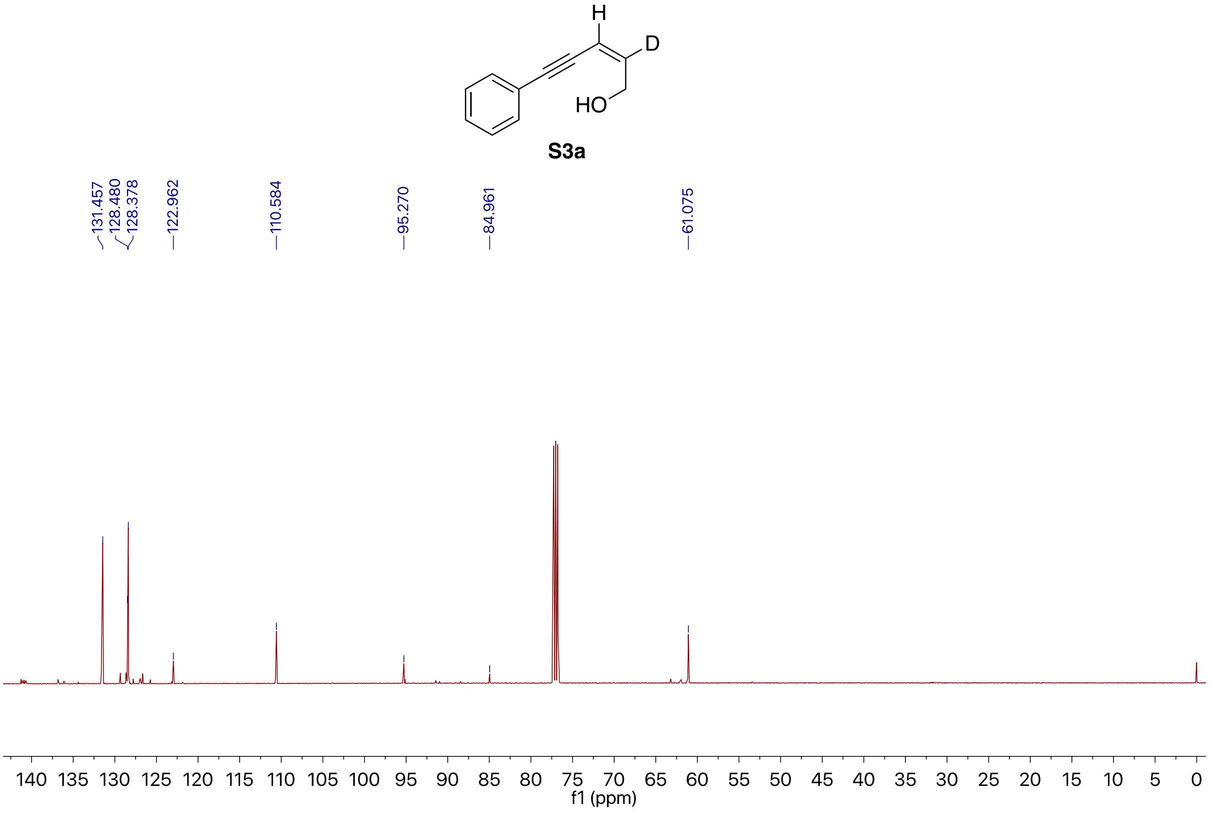

^{1}HNMR, 400 MHz, CDCl3

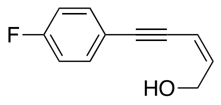

S3b

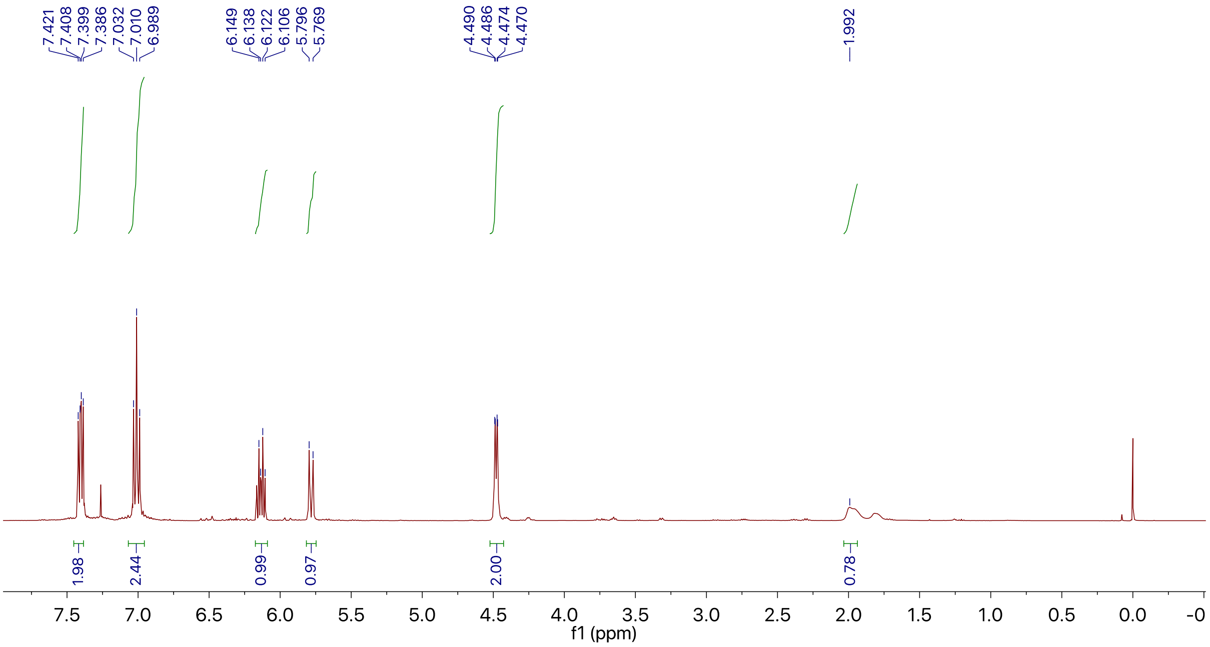

^{13}C NMR, 101 MHz, CDCl3

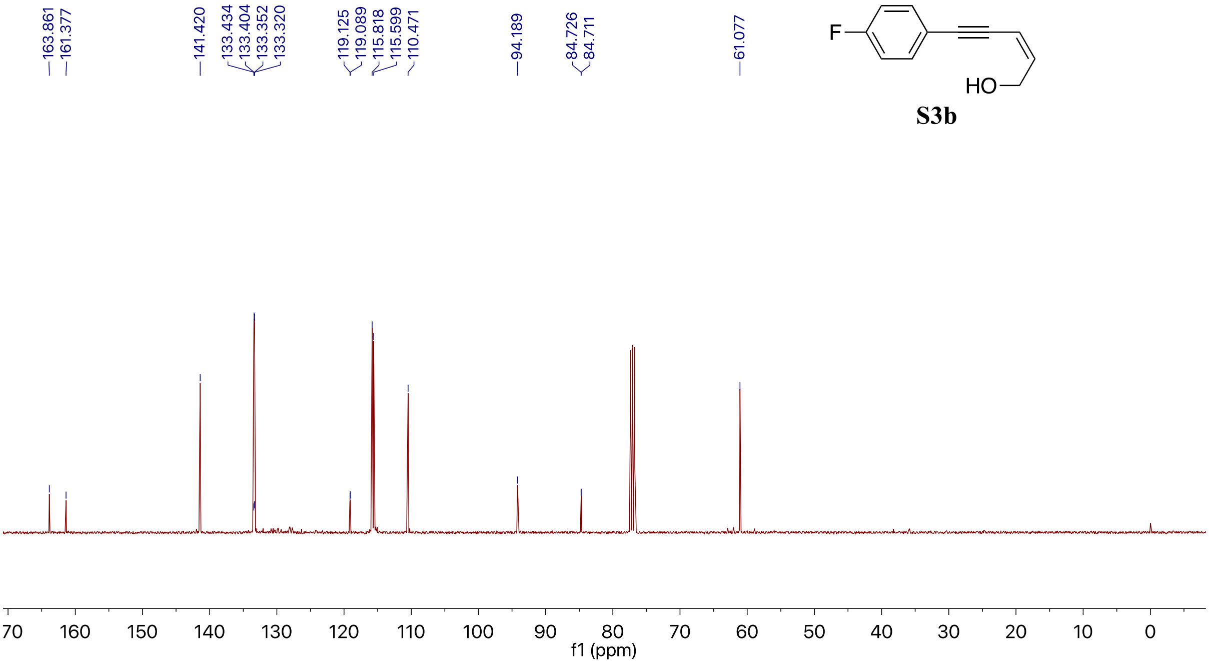

S3b

^{1}H NMR, 500 MHz, $CDCl_3$

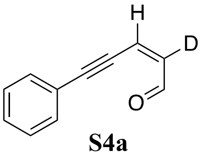

S4a

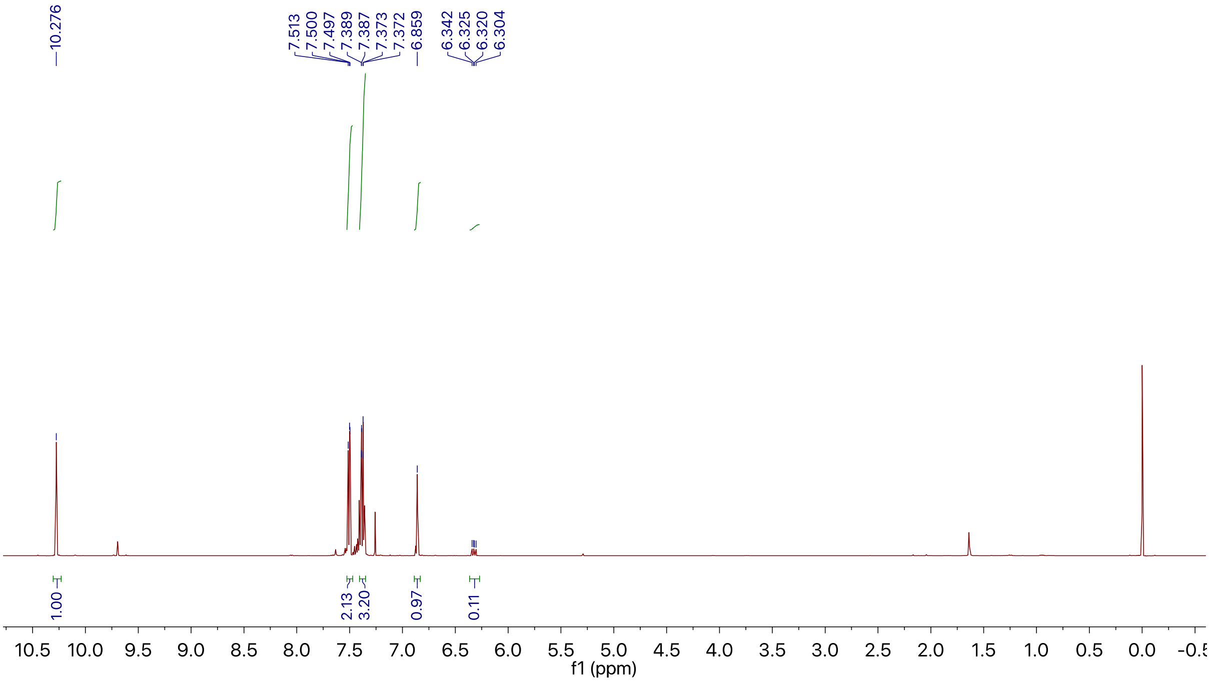

^{13}C NMR, 126 MHz, $CDCl_3$

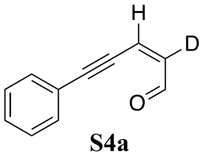

S4a

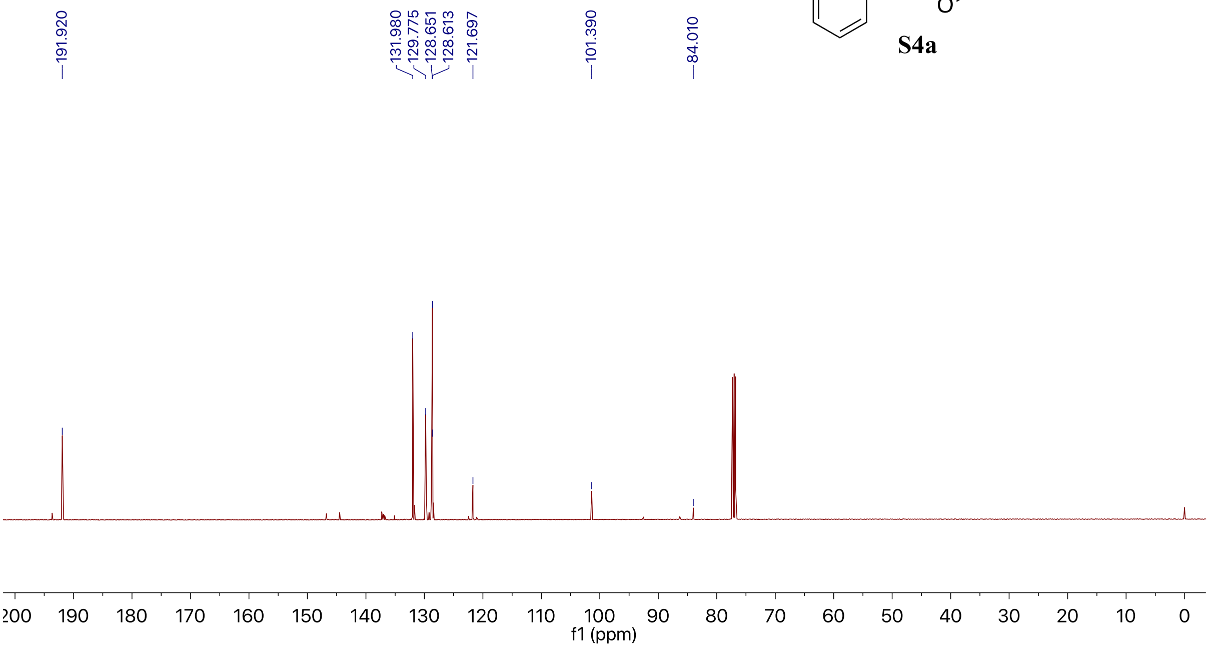

^{1}H NMR, 400 MHz, $CDCl_3$

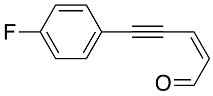

S4b

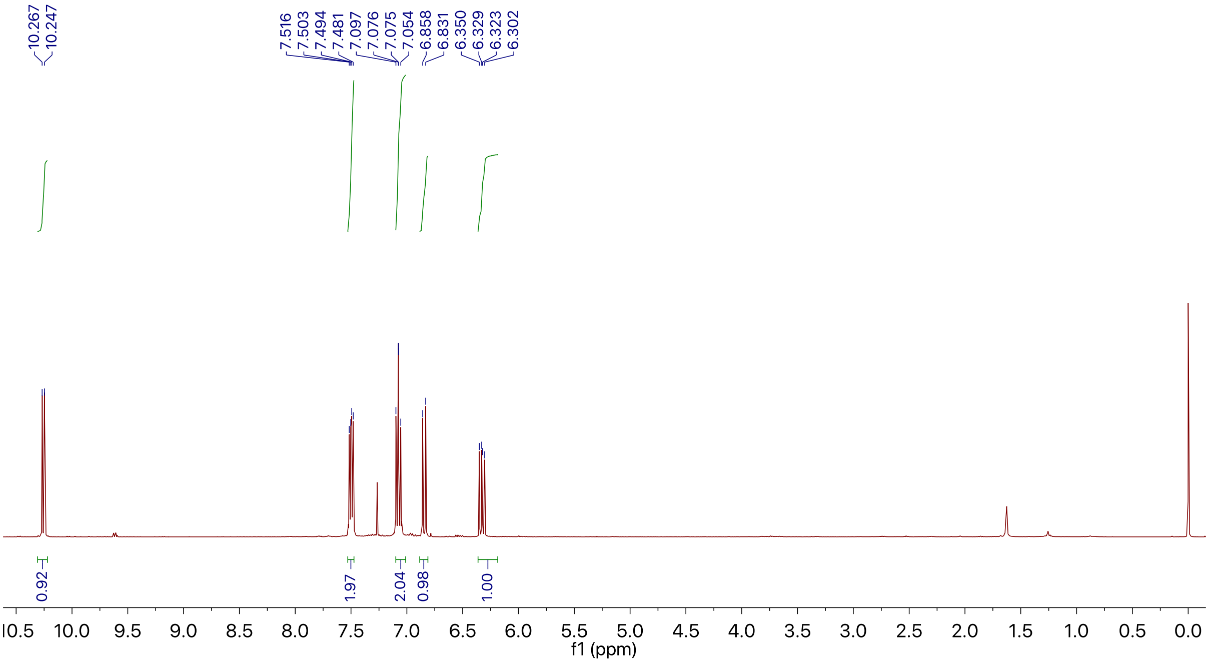

^{13}C NMR, 101 MHz, $CDCl_3$

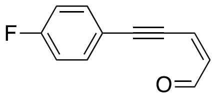

S4b

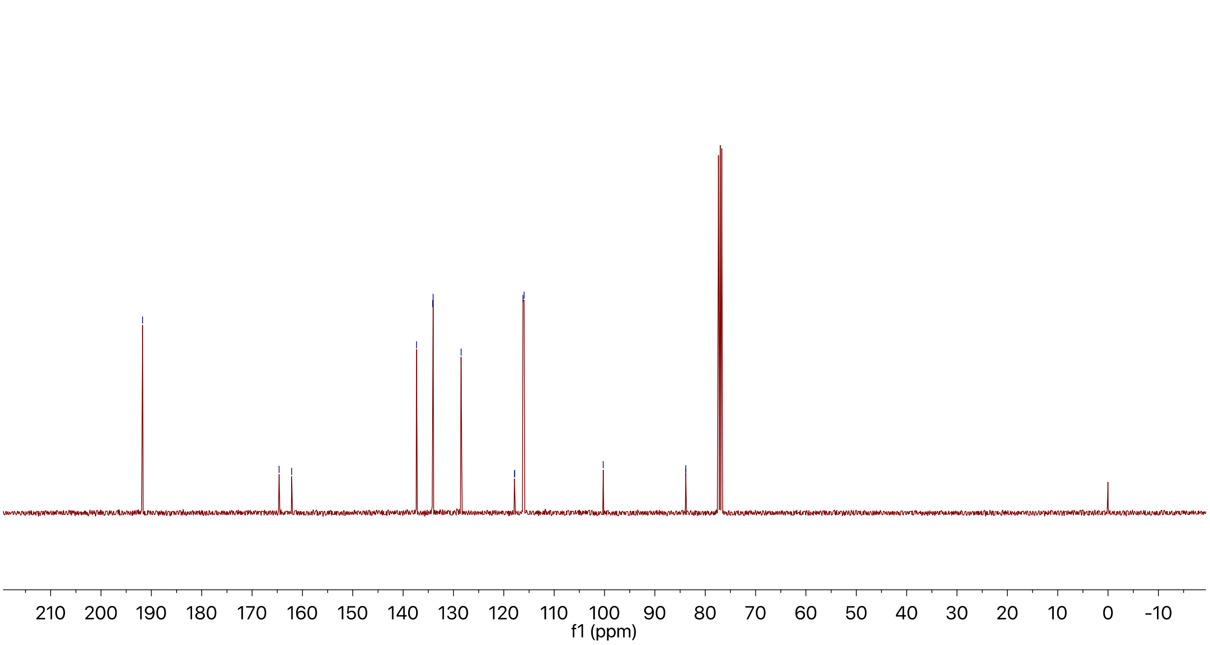

^{1}H NMR, 500 MHz, $CDCl_3$

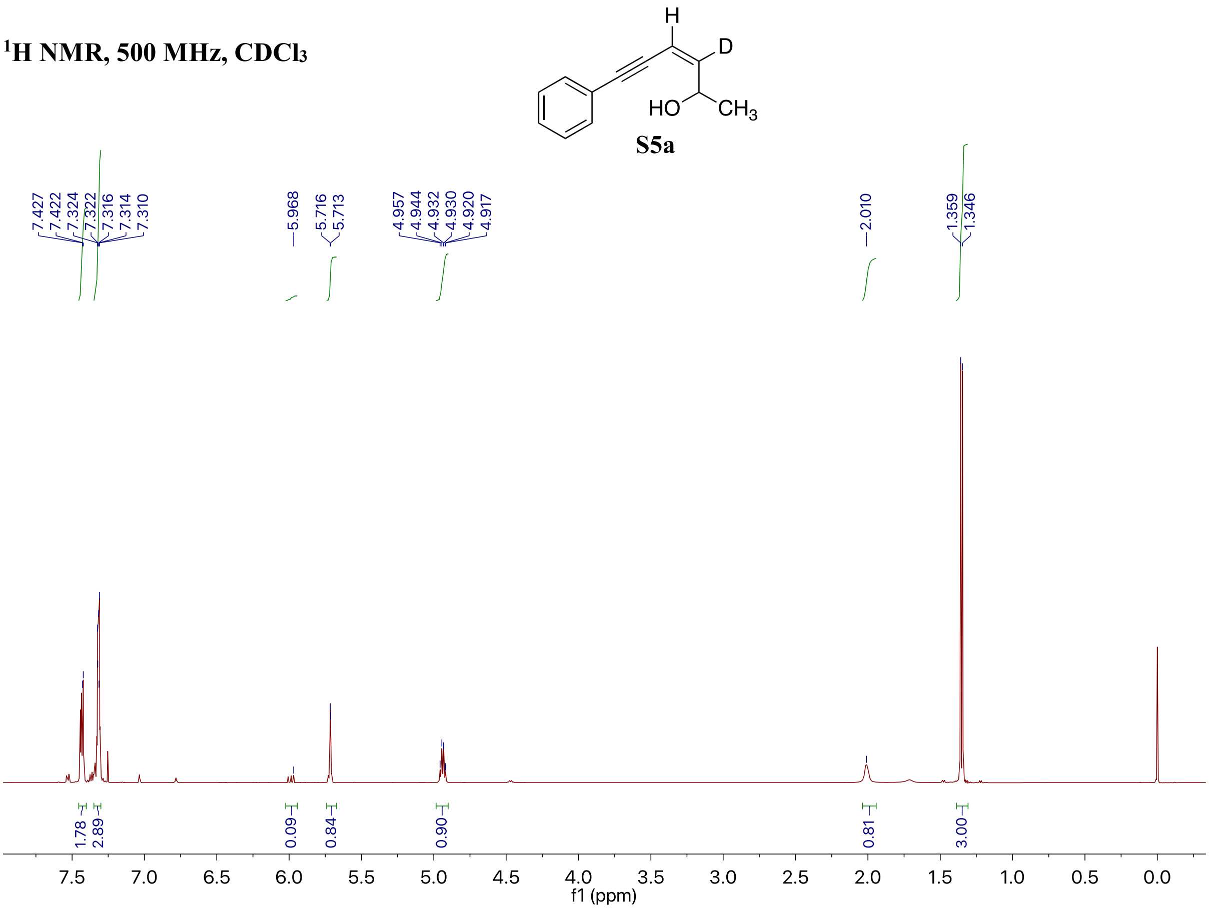

^{13}C NMR, 126 MHz, $CDCl_3$

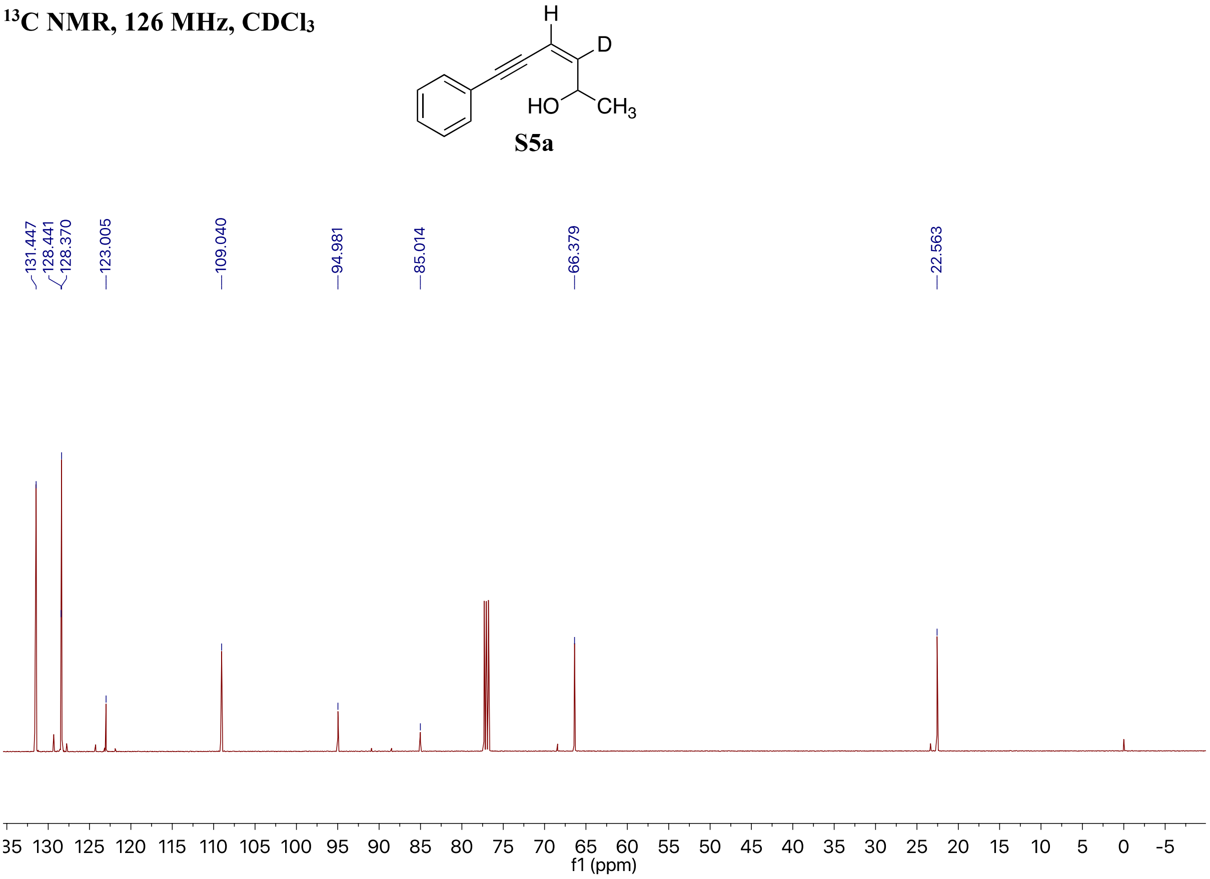

^{1}H NMR, 500 MHz, $CDCl_3$

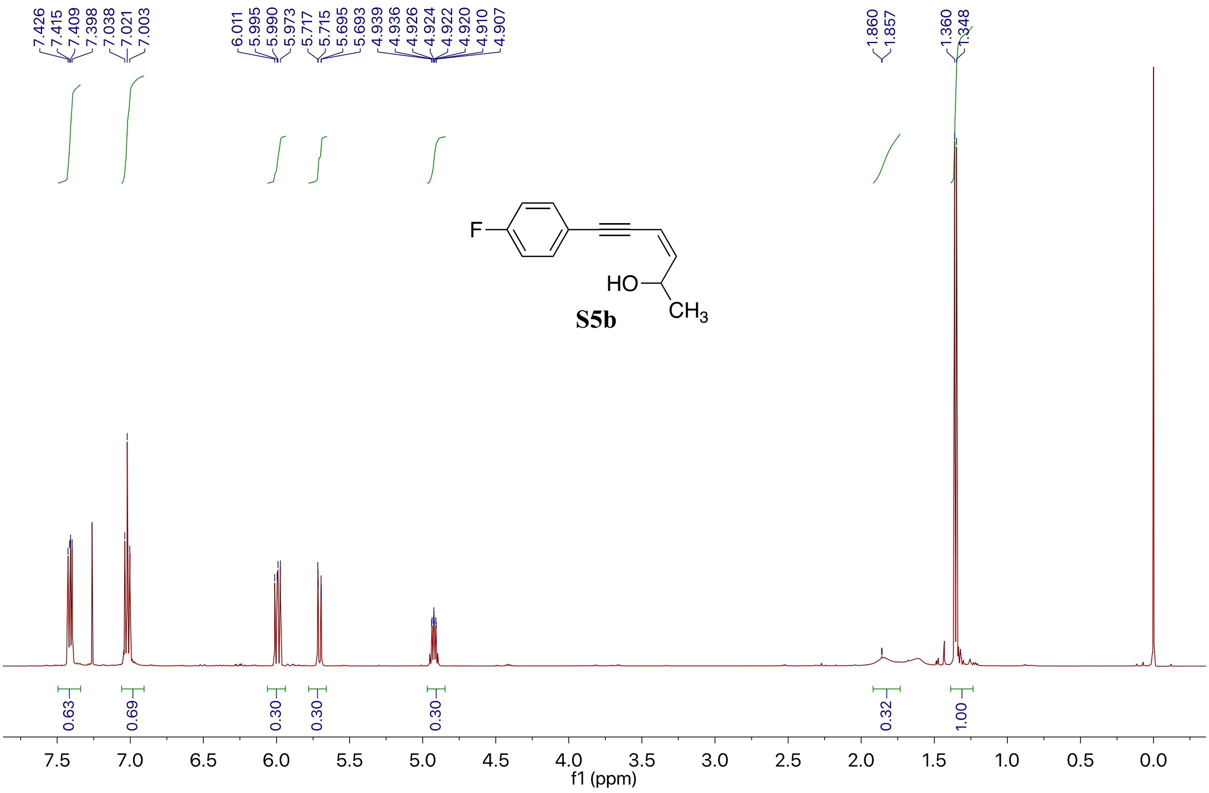

^{13}C NMR, 126 MHz, $CDCl_3$

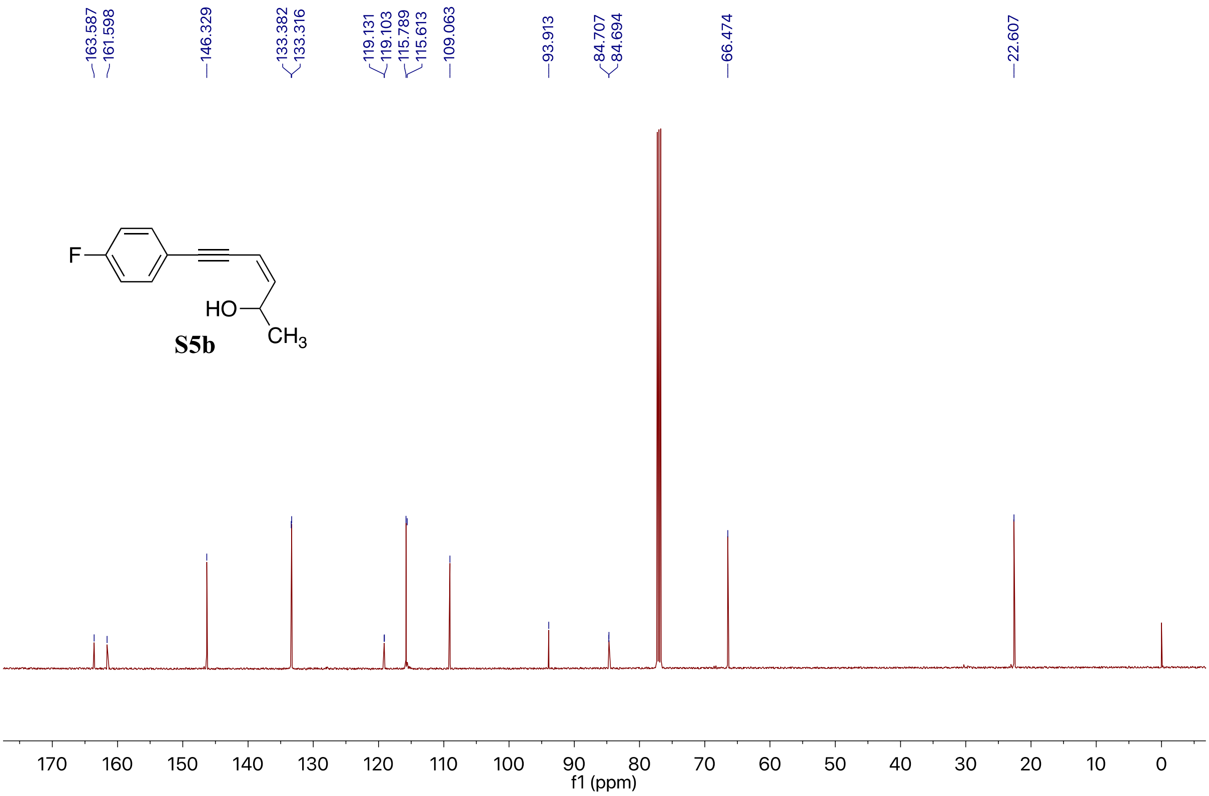

^{1}H NMR, 500 MHz, $CDCl_3$

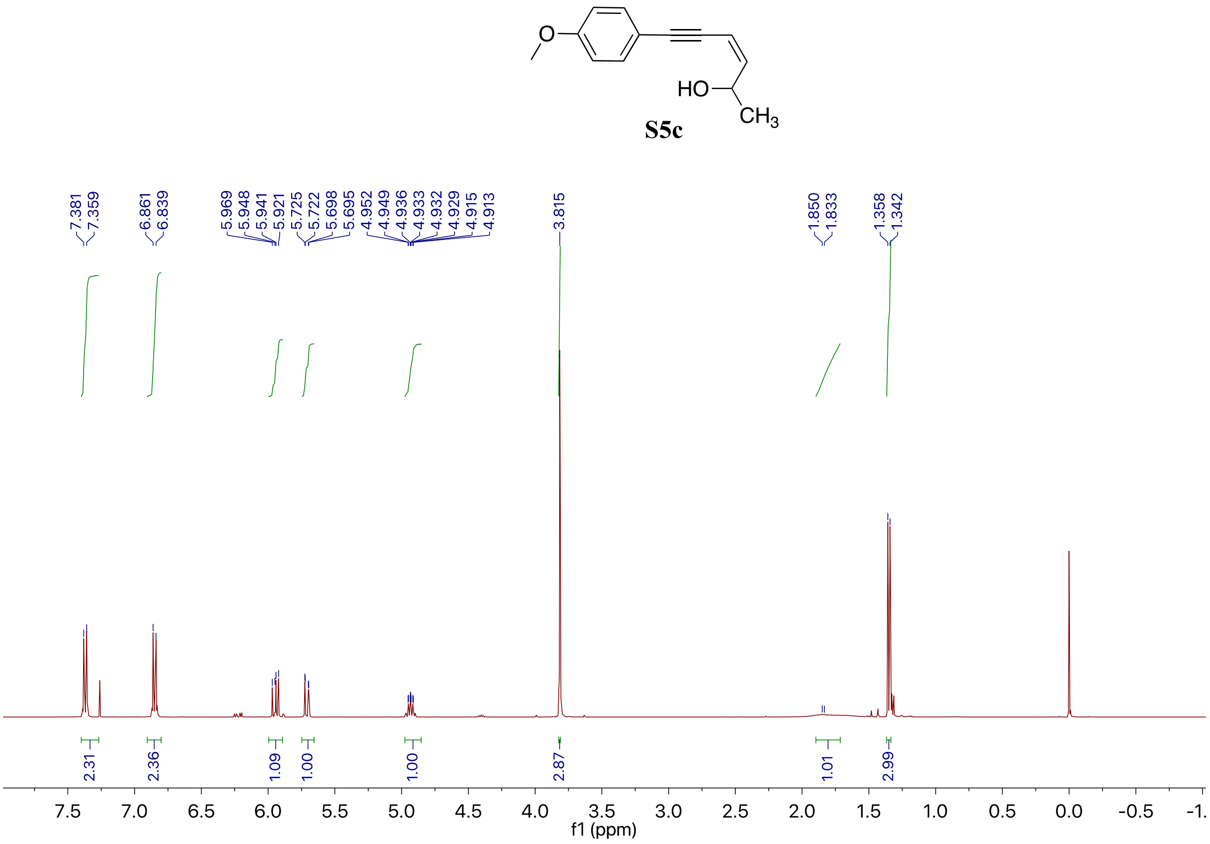

^{13}C NMR, 101 MHz, $CDCl_3$

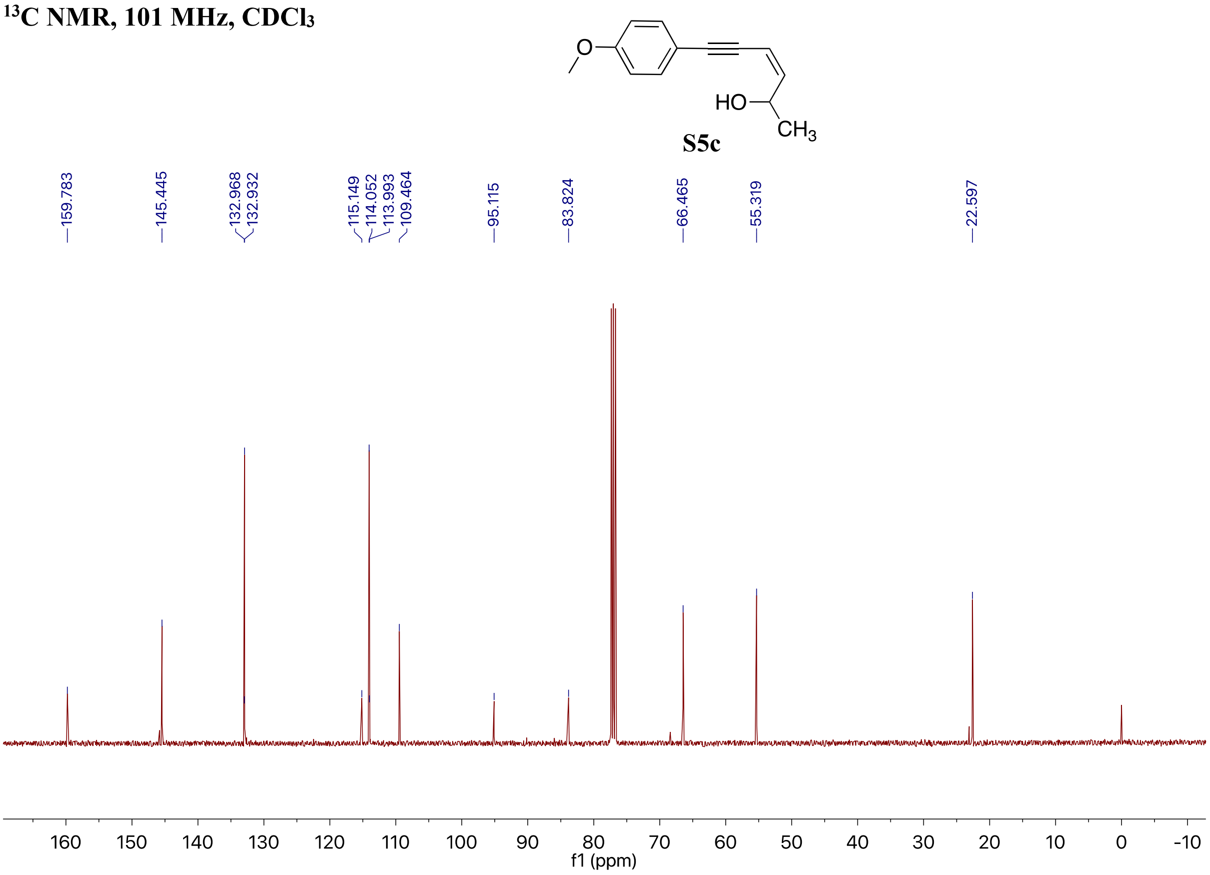

^{1}H NMR, 500 MHz, $CDCl_3$

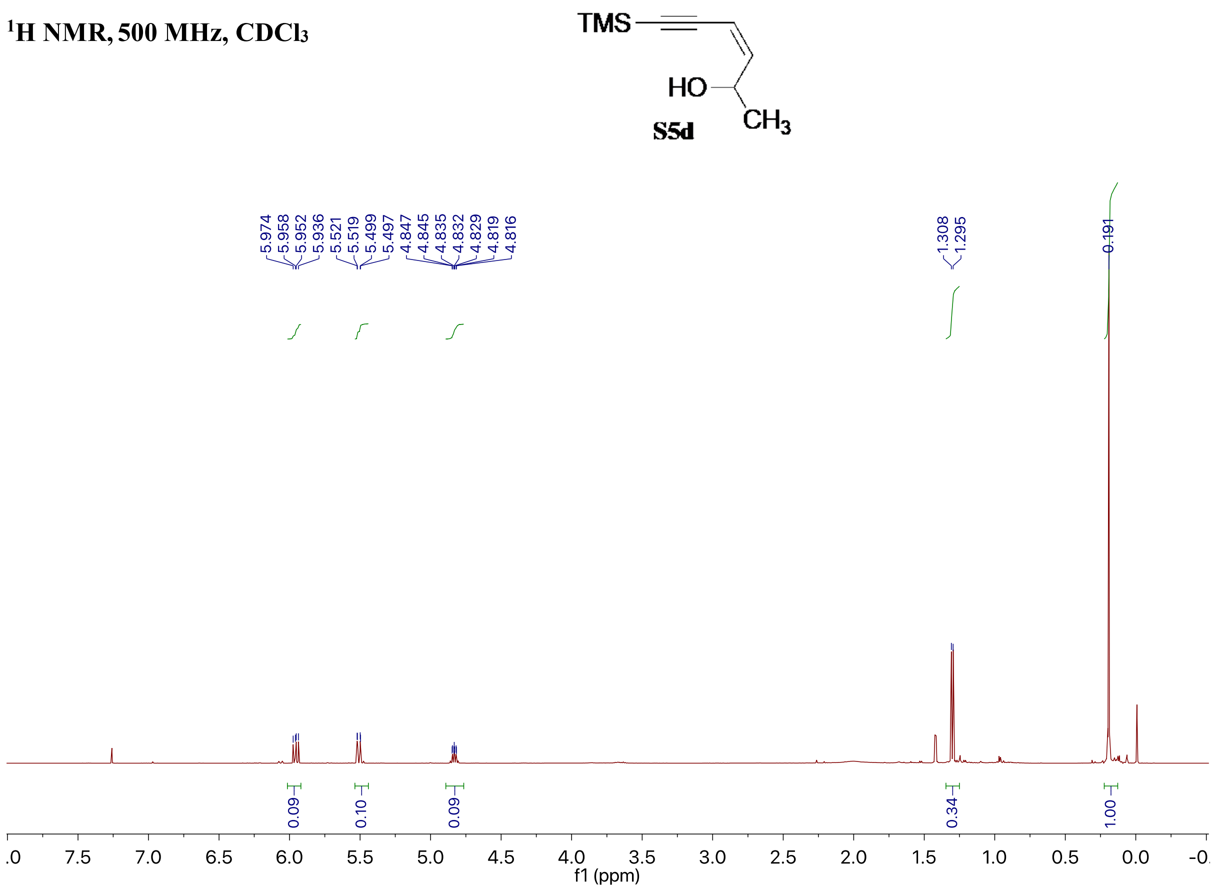

^{13}C NMR,126 MHz, $CDCl_3$

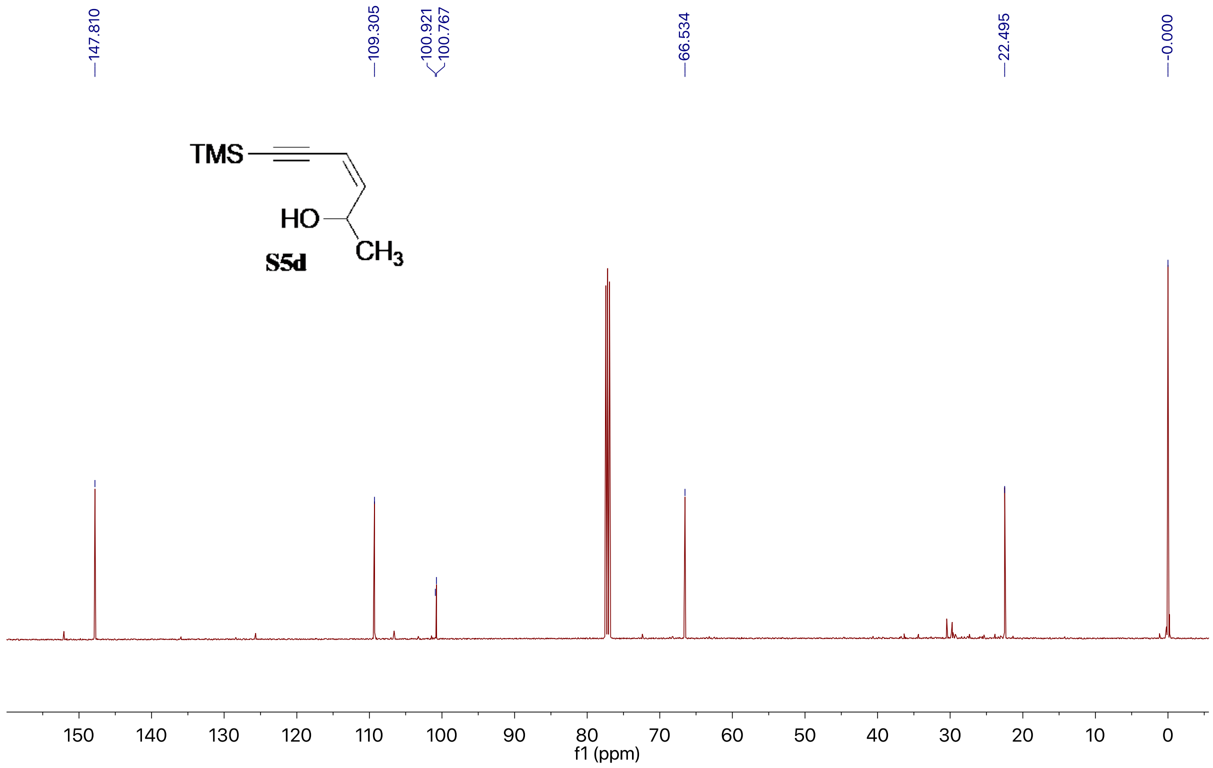

^{1}H NMR, 400 MHz, CDCl$_3$

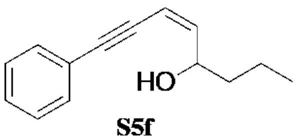

S5f

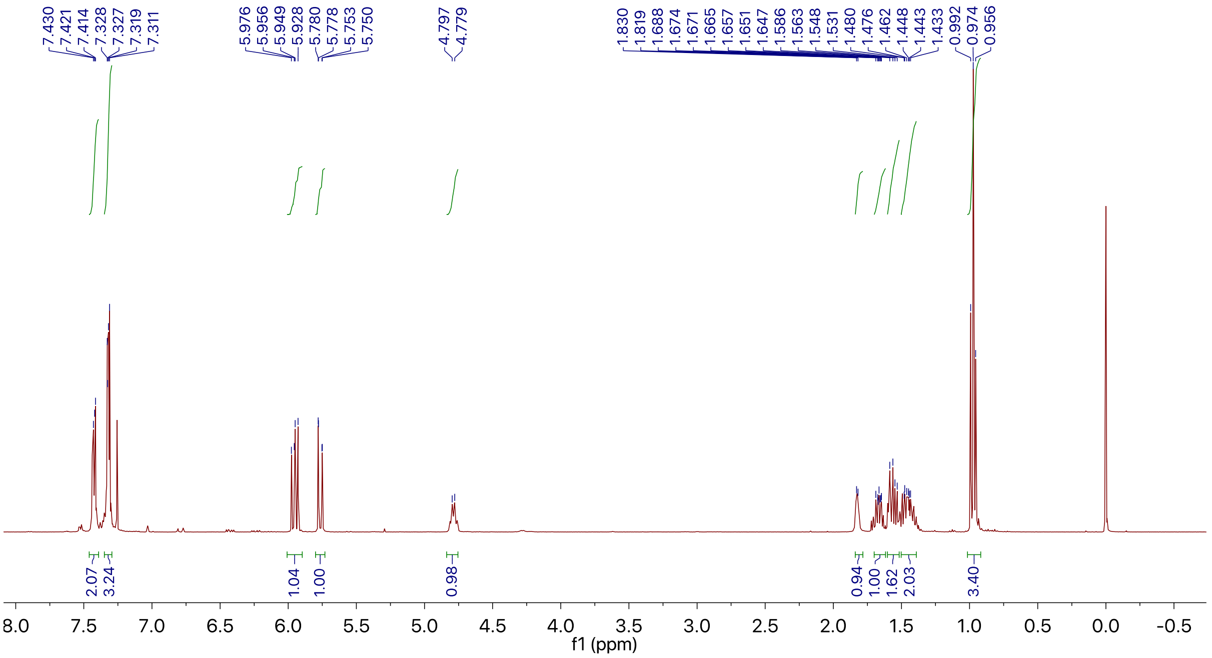

^{13}C NMR, 101 MHz, CDCl$_3$

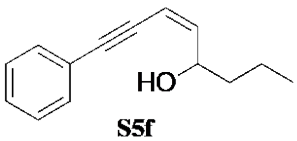

S5f

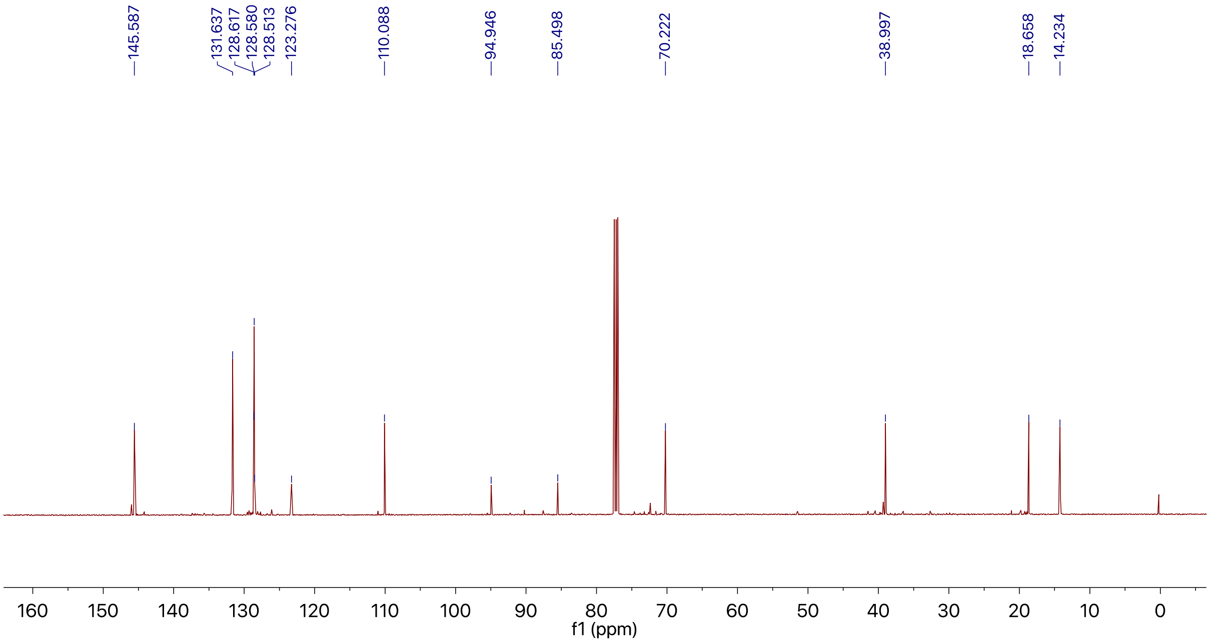

^{1}H NMR, 400 MHz, $CDCl_3$

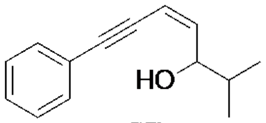

S5i

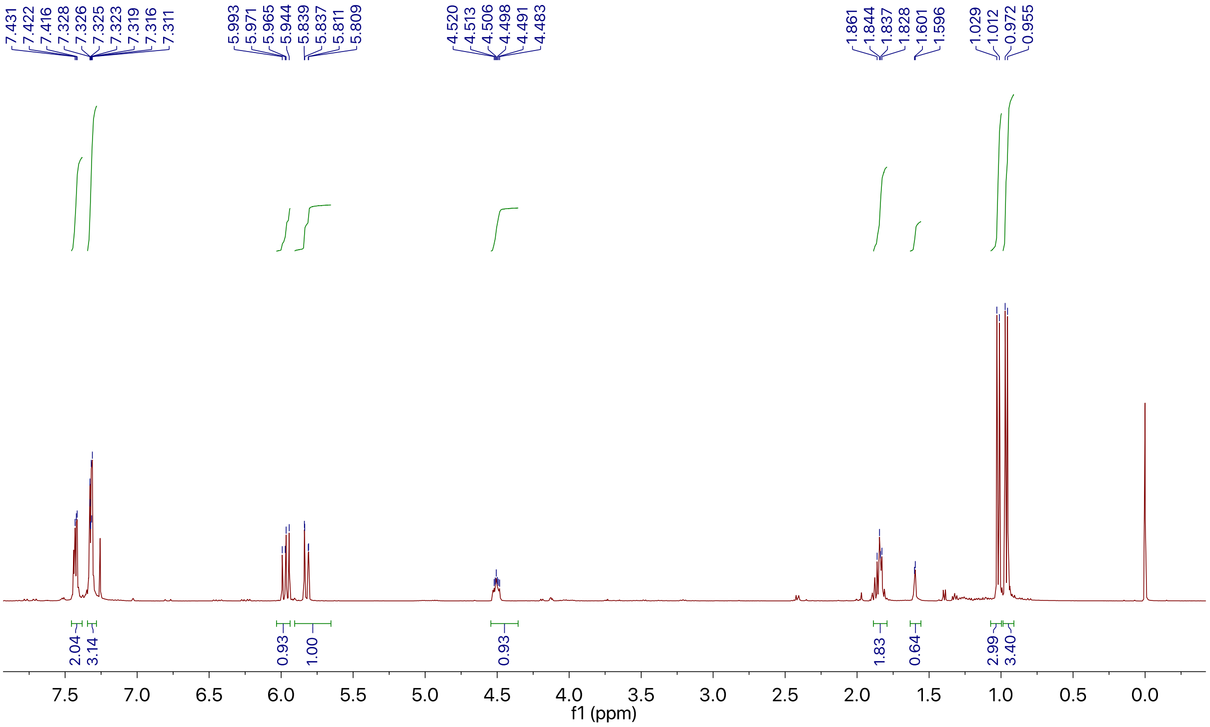

^{13}C NMR, 101 MHz, $CDCl_3$

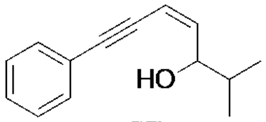

S5i

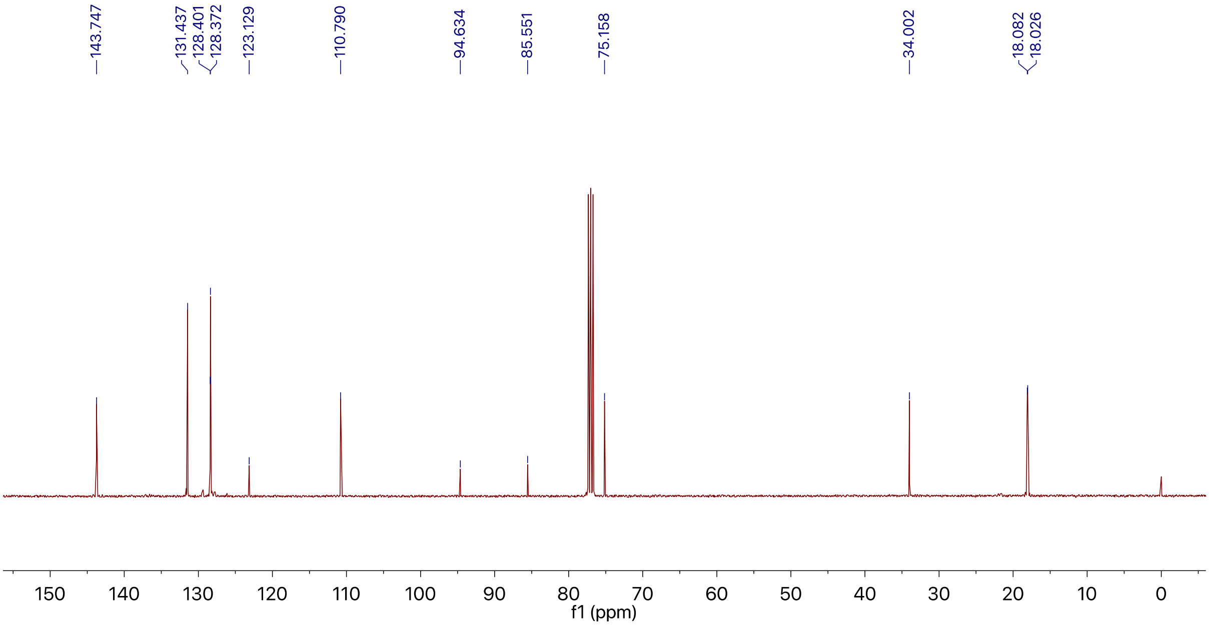

^{1}H NMR, 500 MHz, CDCl$_3$

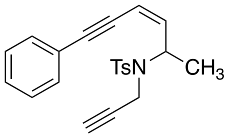

1a

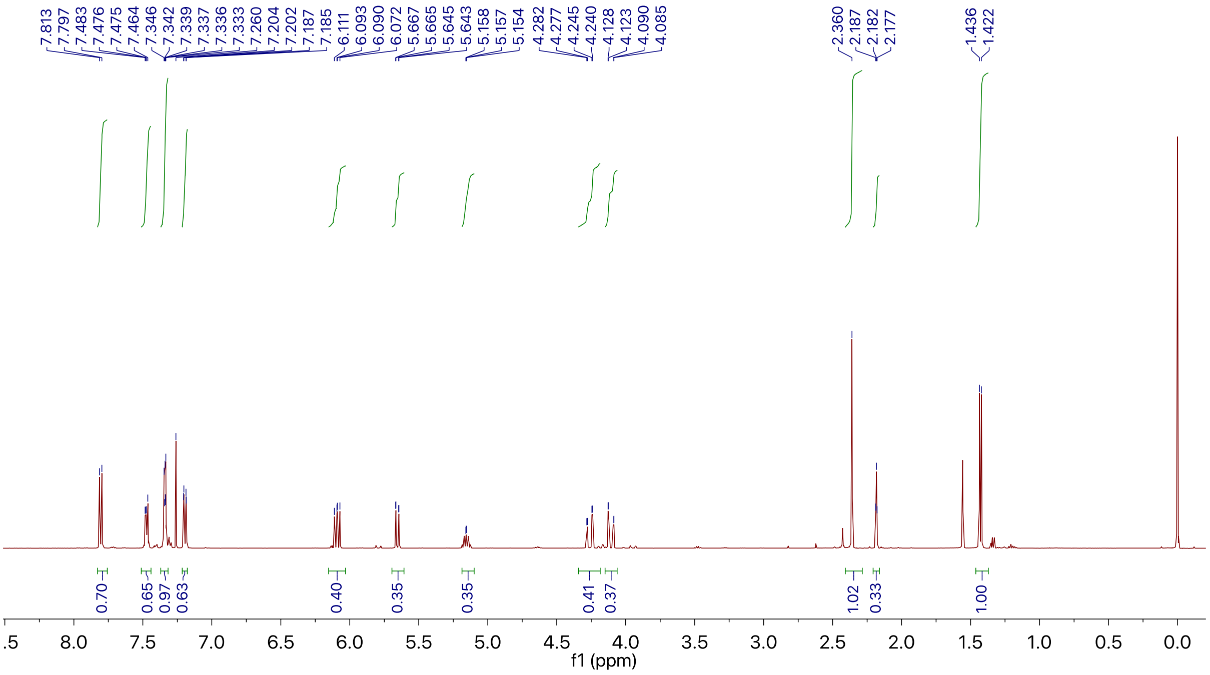

^{13}C NMR, 126 MHz, CDCl$_3$

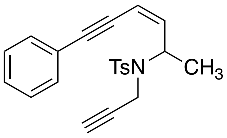

1a

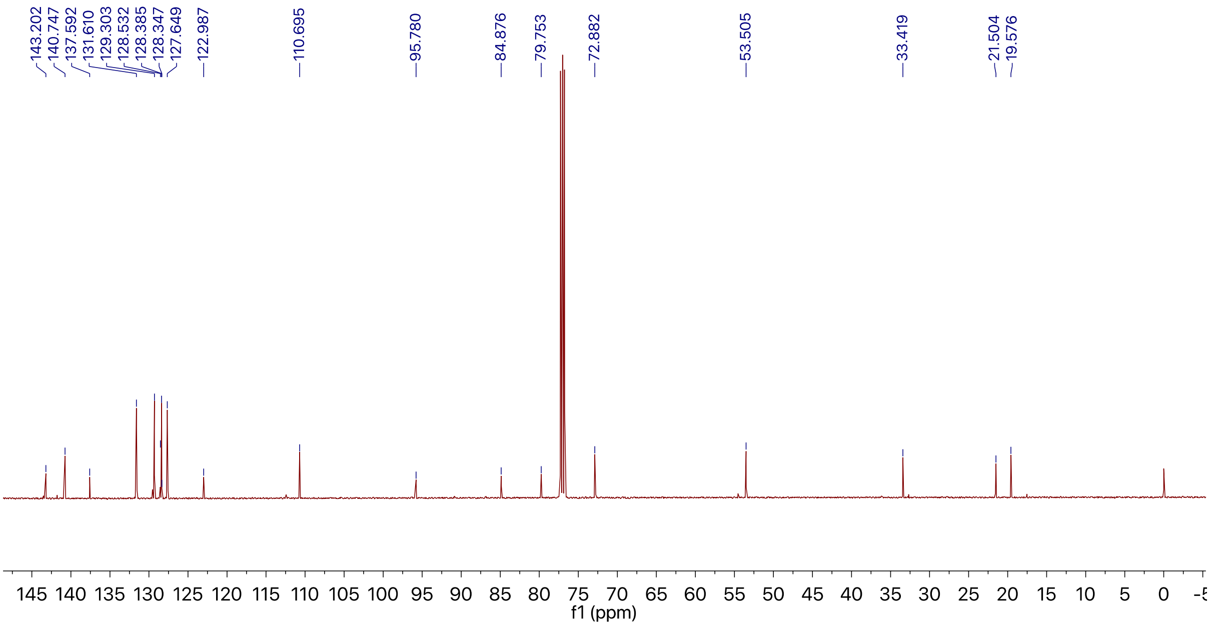

^{1}H NMR, 500 MHz, $CDCl_3$

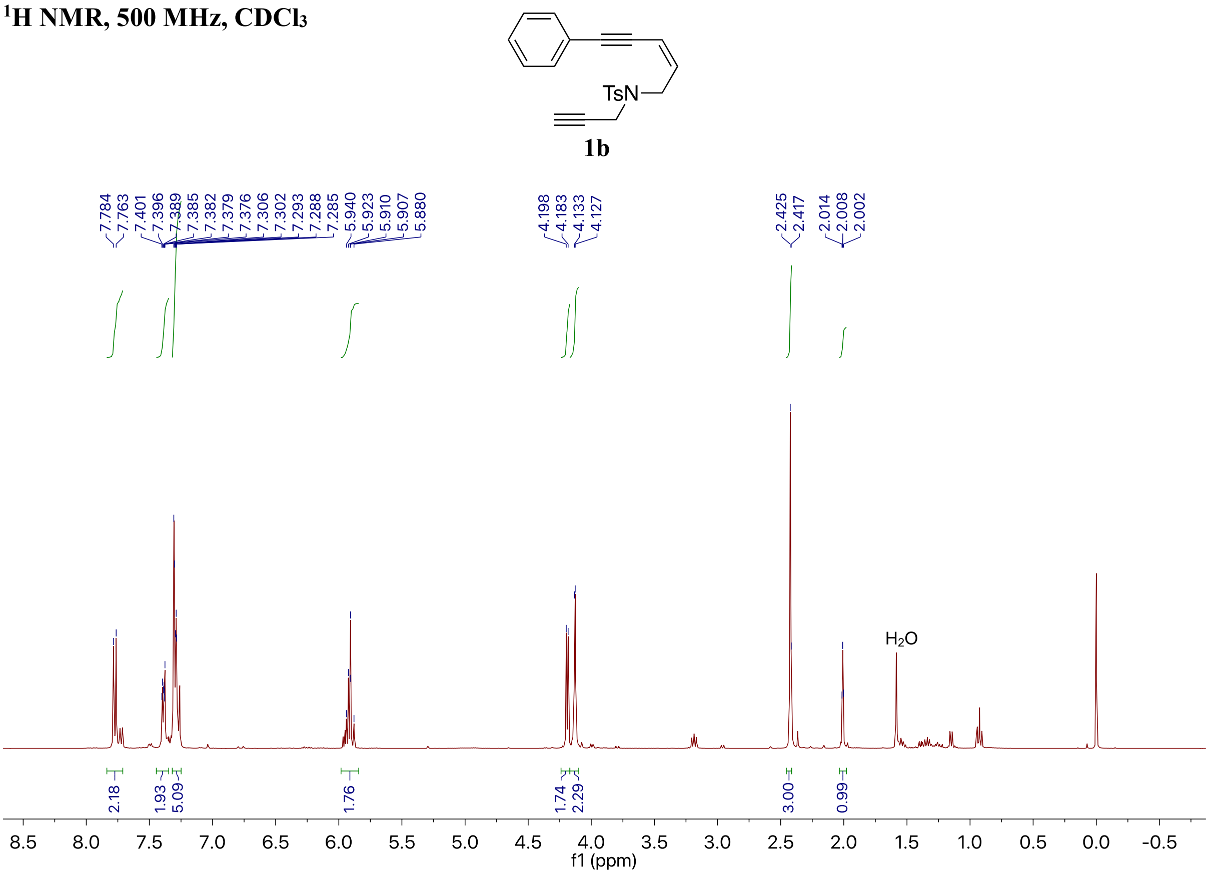

^{13}C NMR, 126 MHz, $CDCl_3$

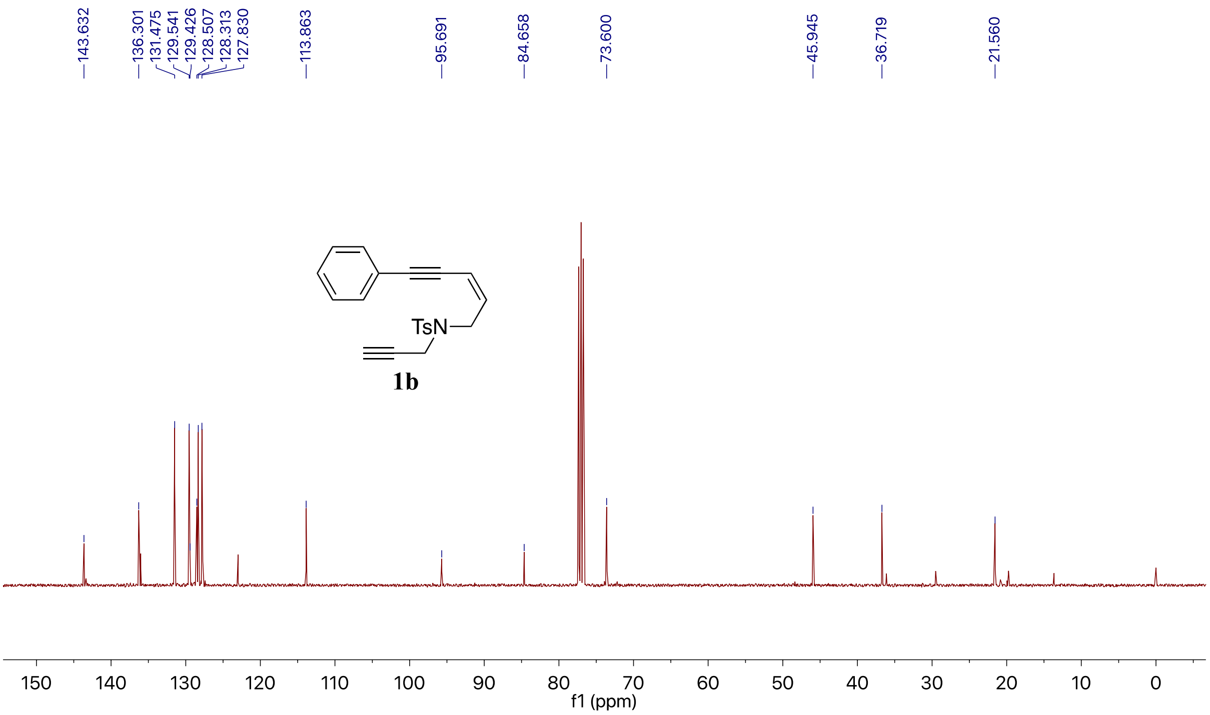

^{1}H NMR,500 MHz, $CDCl_3$

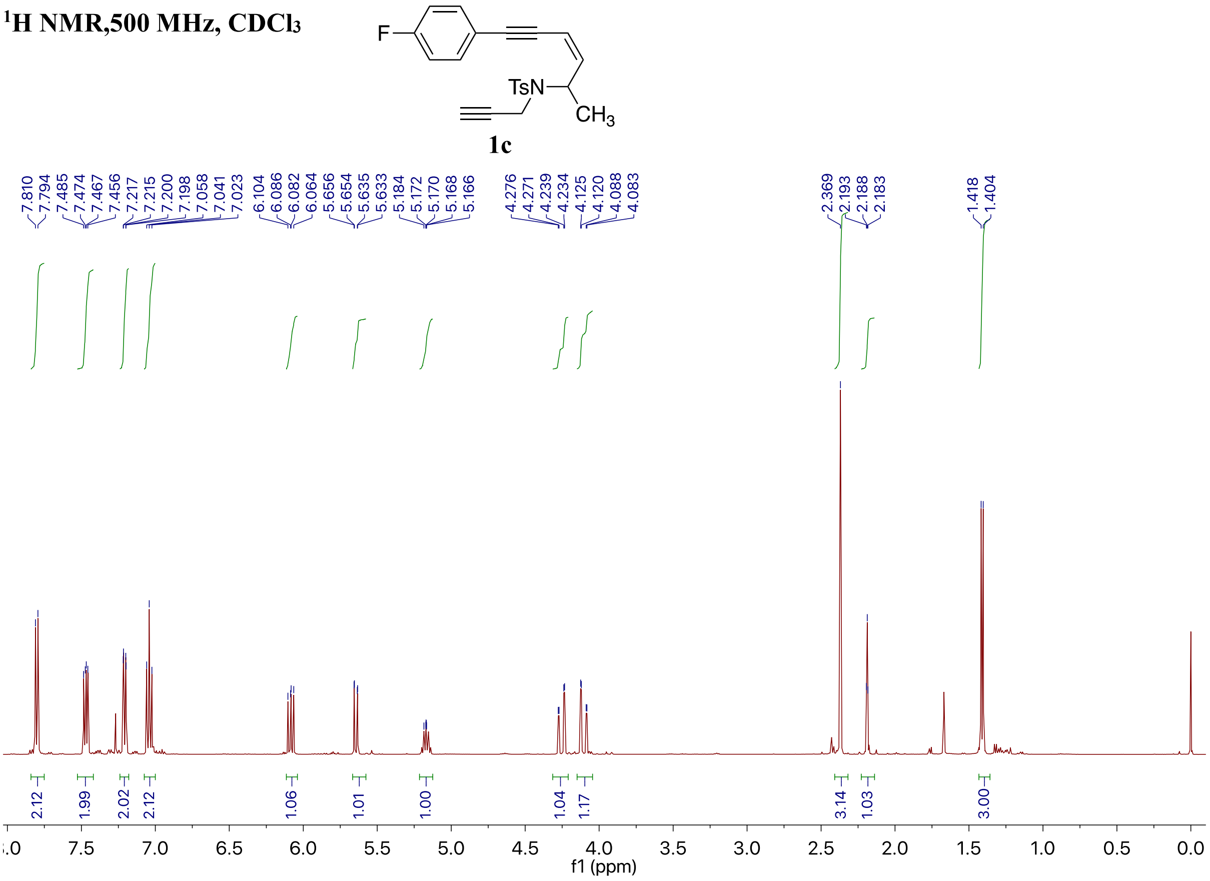

^{13}C NMR, 126 MHz, $CDCl_3$

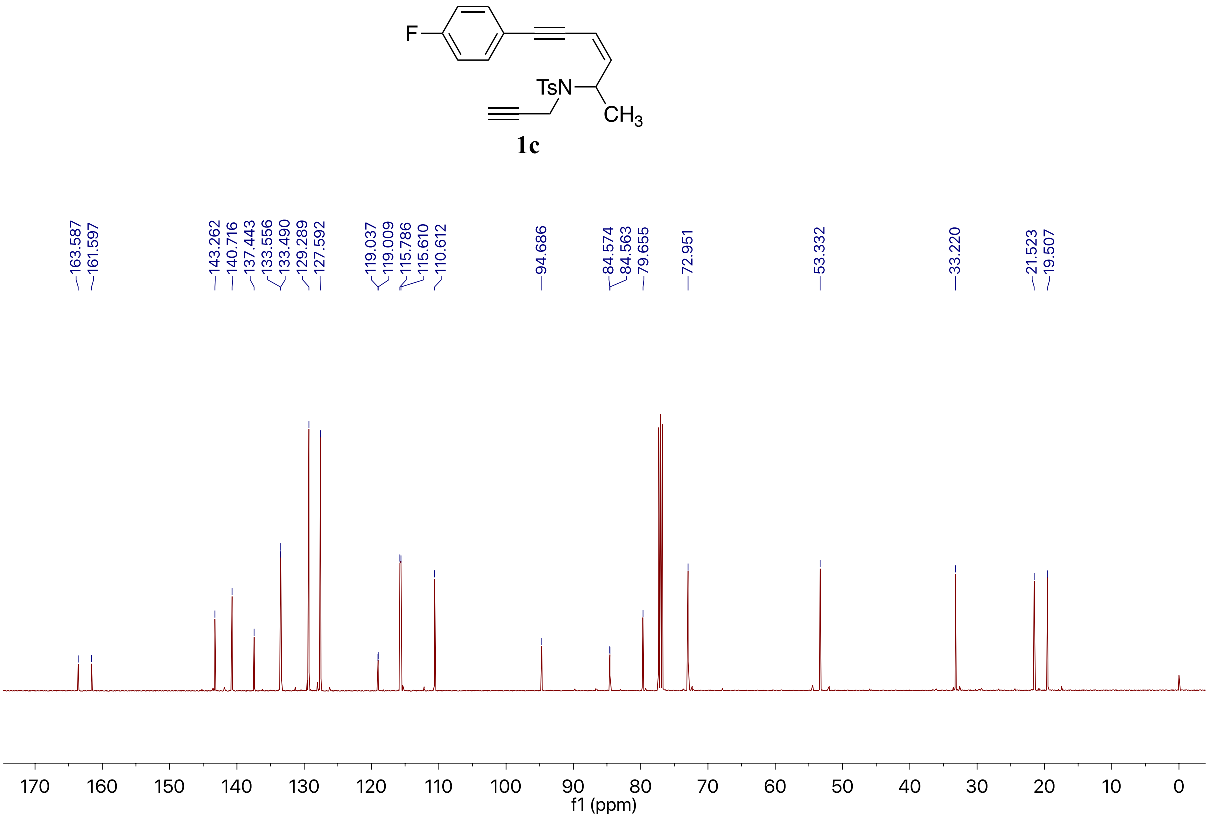

^{1}H NMR, 400 MHz, CDCl3

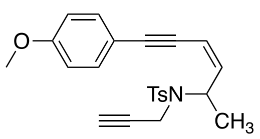

1d

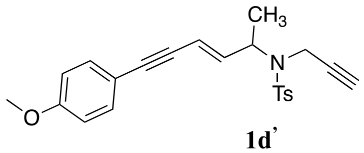

1d'

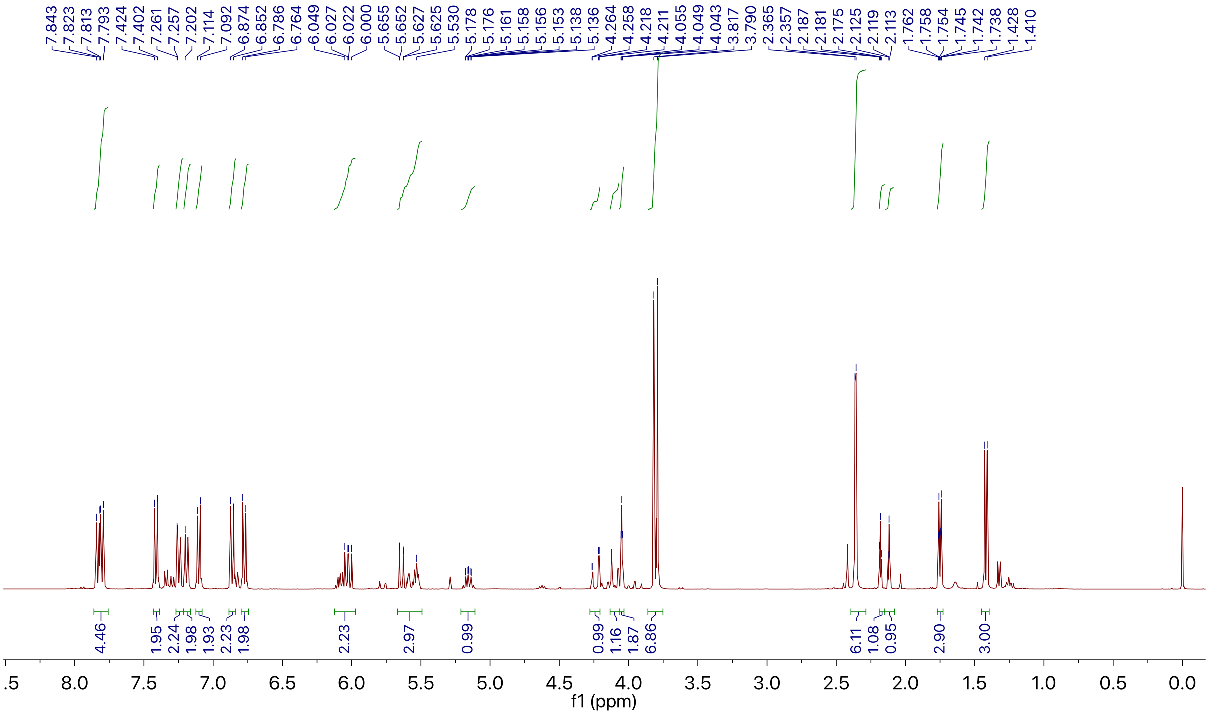

^{13}C NMR, 101 MHz, CDCl3

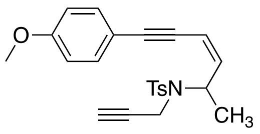

1d

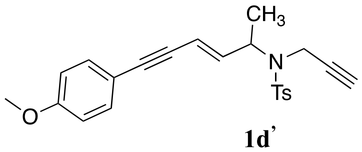

1d'

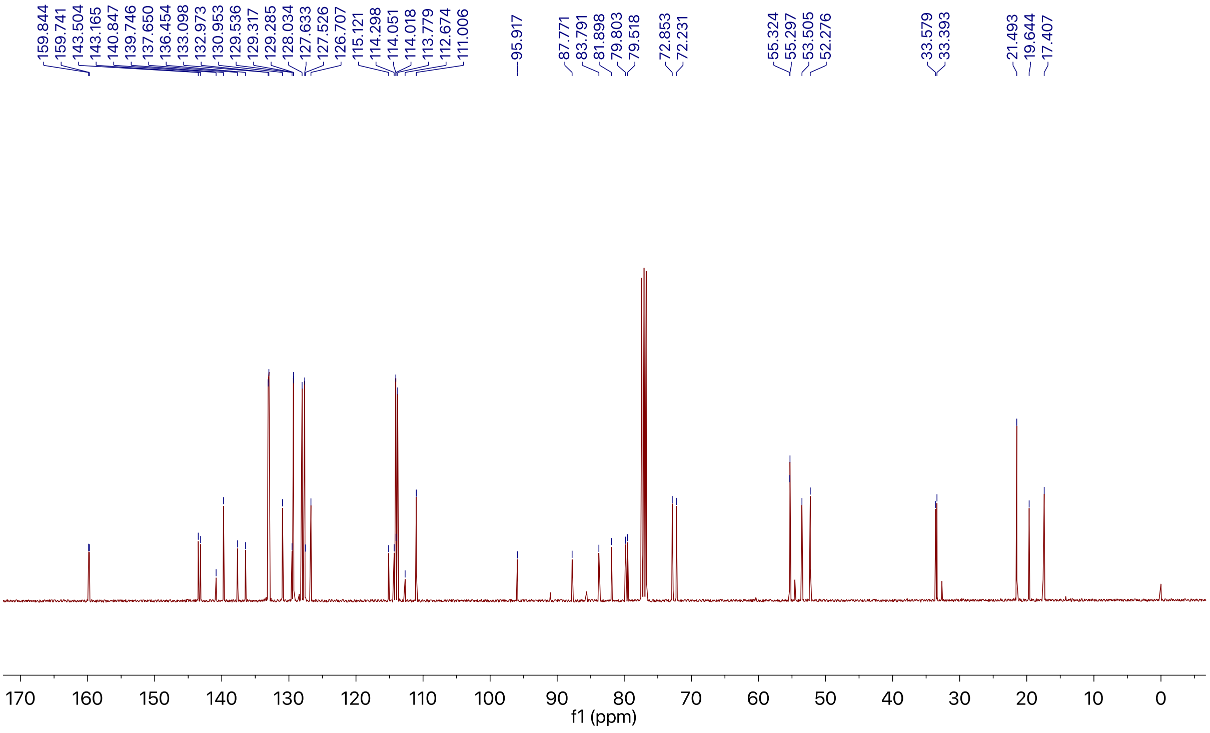

^{1}H NMR, 400 MHz, $CDCl_3$

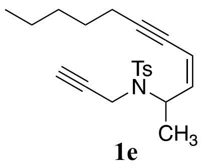

1e

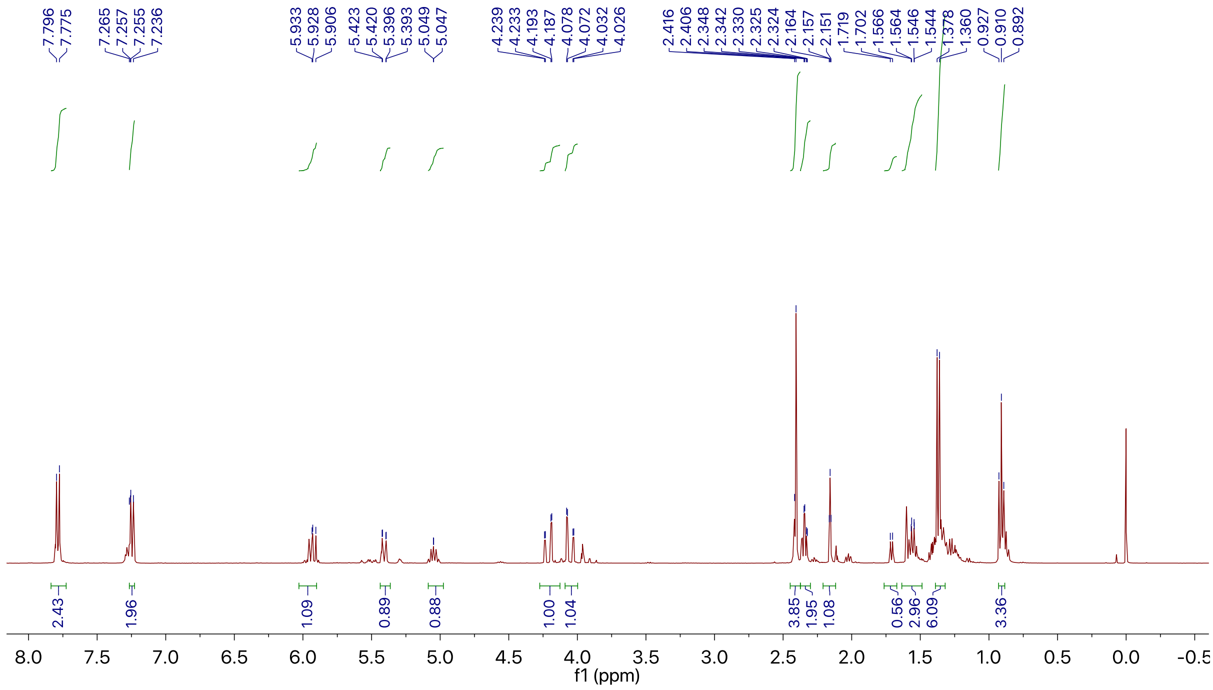

^{13}C NMR, 101 MHz, $CDCl_3$

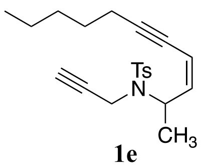

1e

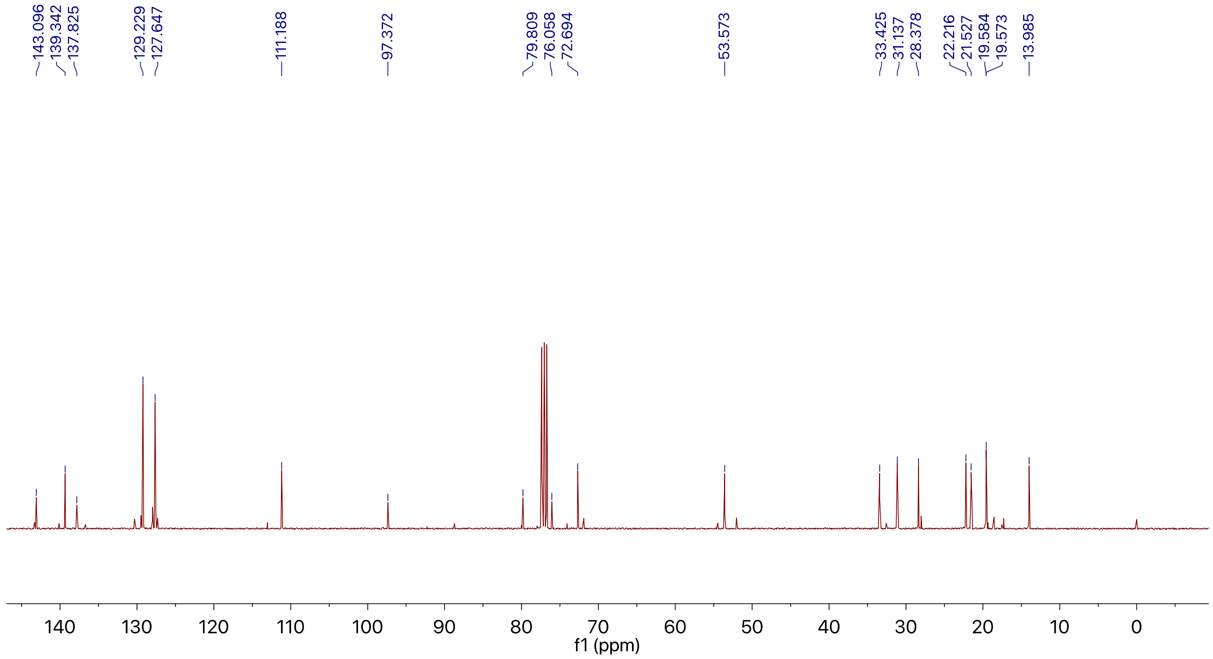

^{1}H NMR, 500 MHz, $CDCl_3$

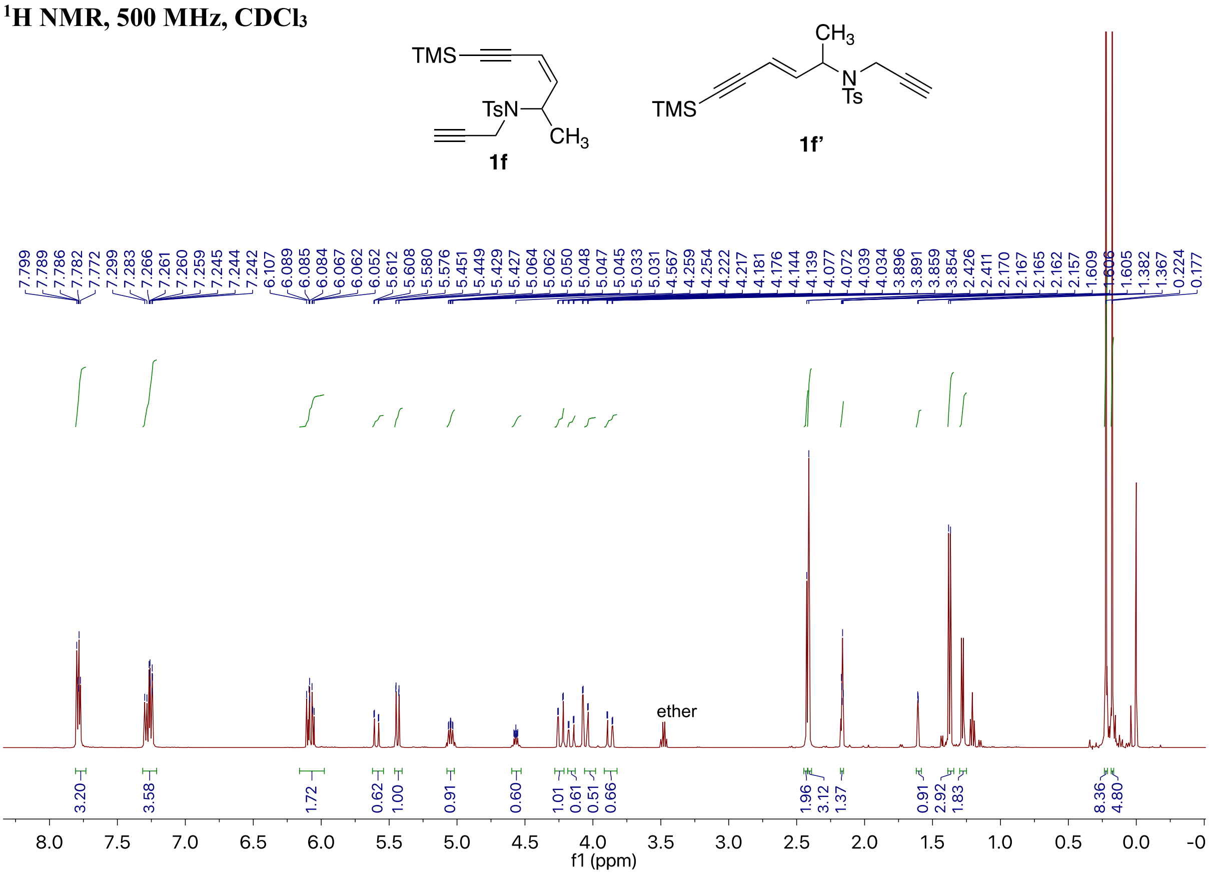

^{13}C NMR, 126 MHz, $CDCl_3$

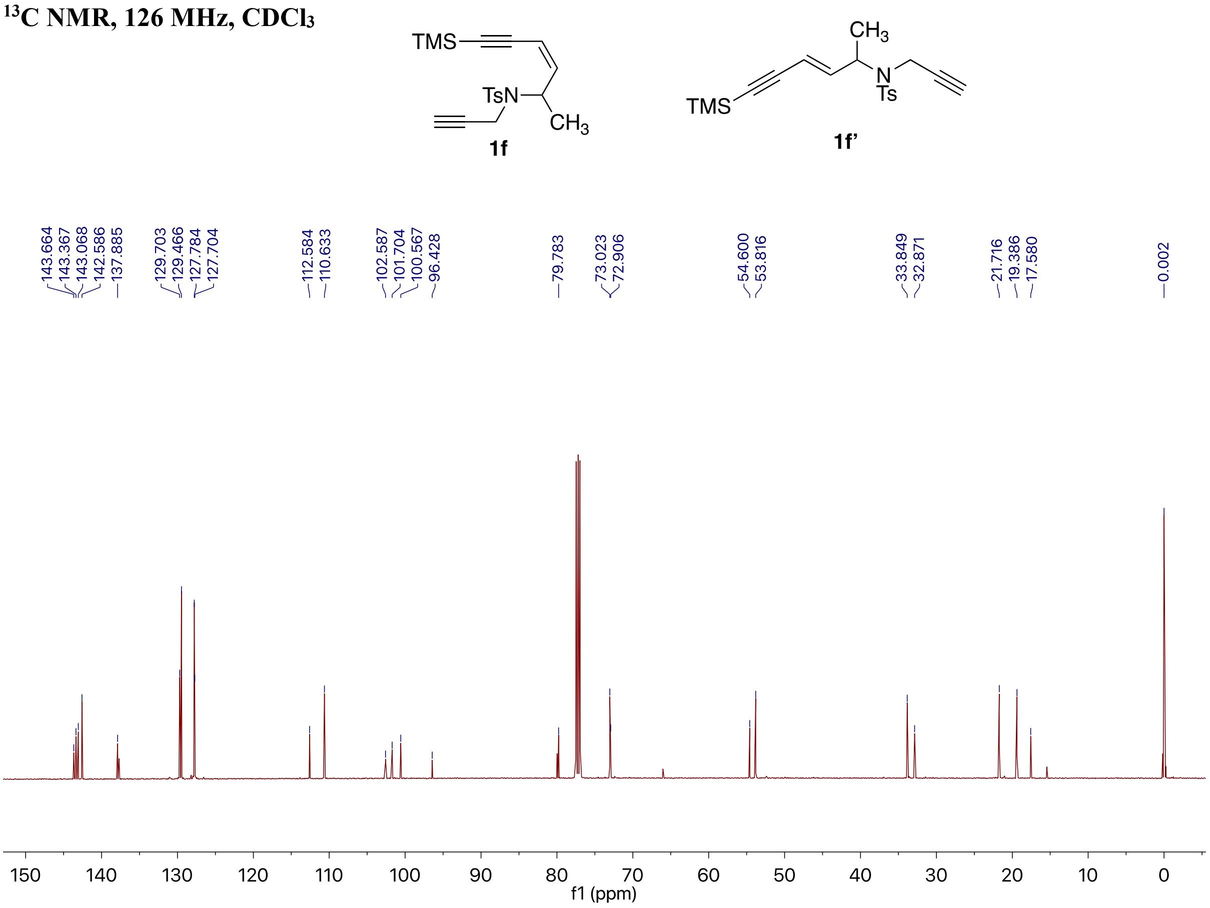

^{1}H NMR, 500 MHz, $CDCl_3$

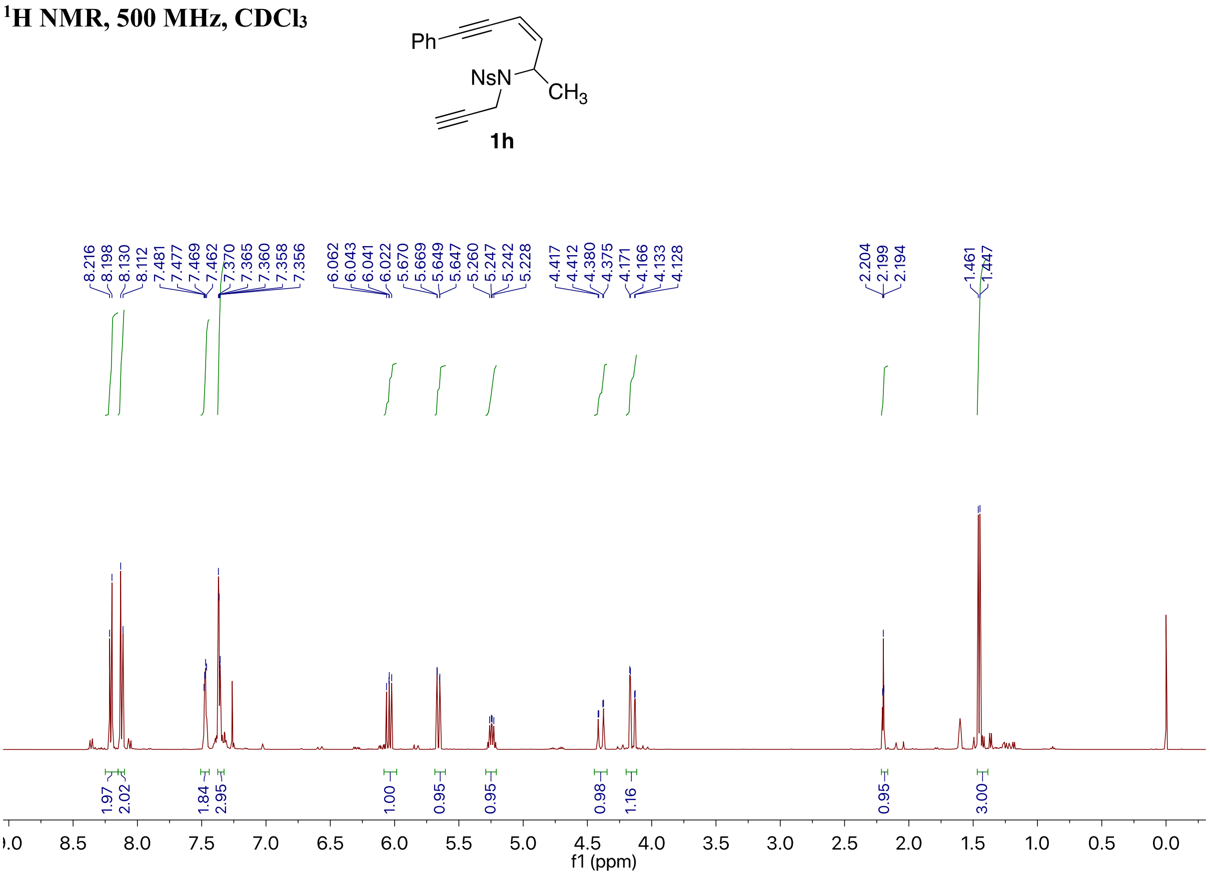

^{13}C NMR, 126 MHz, $CDCl_3$

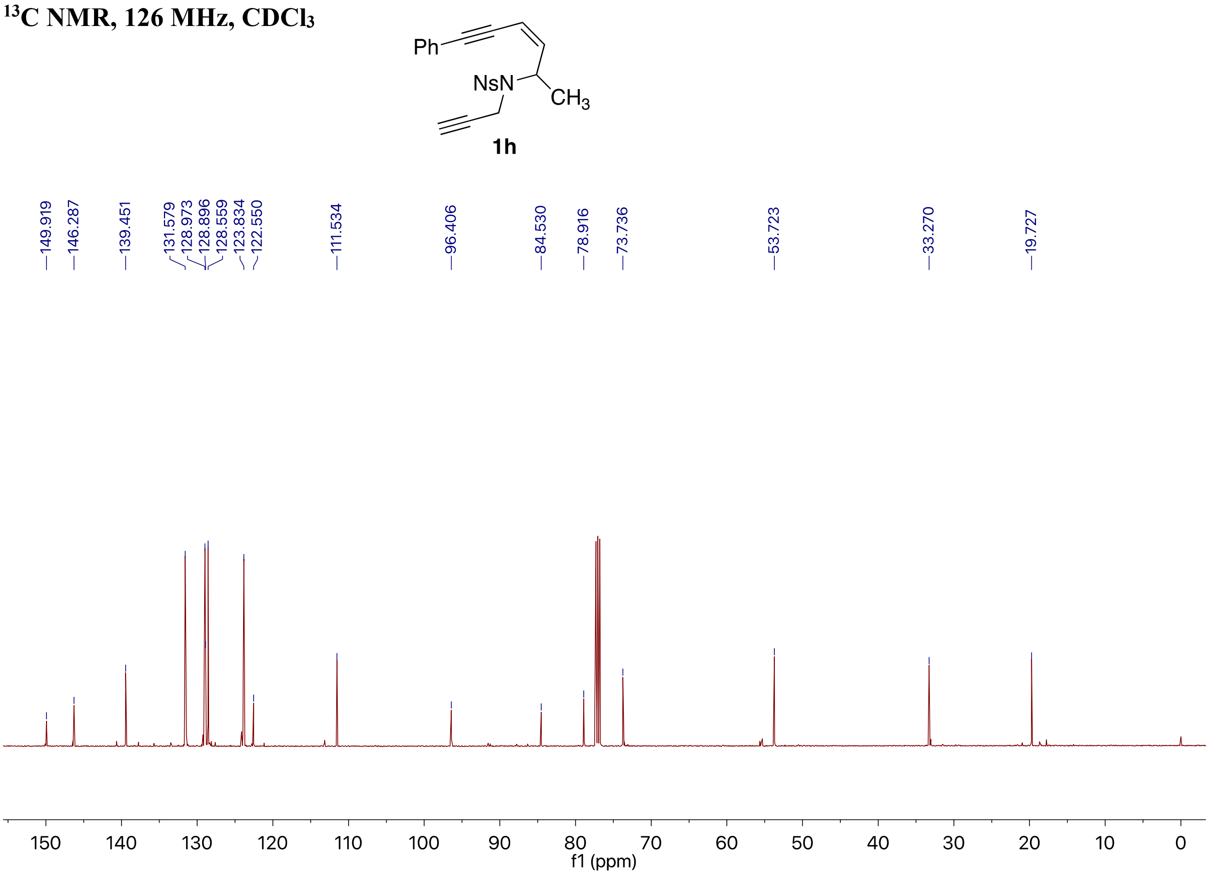

^{1}H NMR, 400 MHz, $CDCl_3$

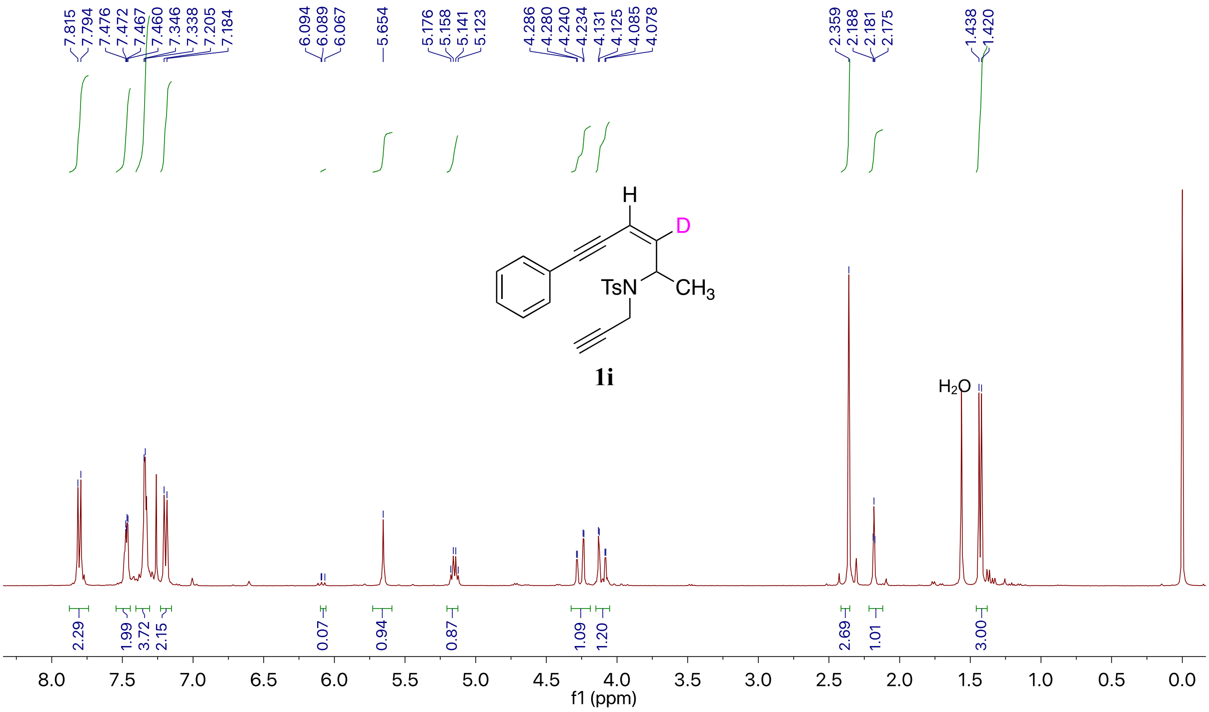

^{13}C NMR, 101 MHz, $CDCl_3$

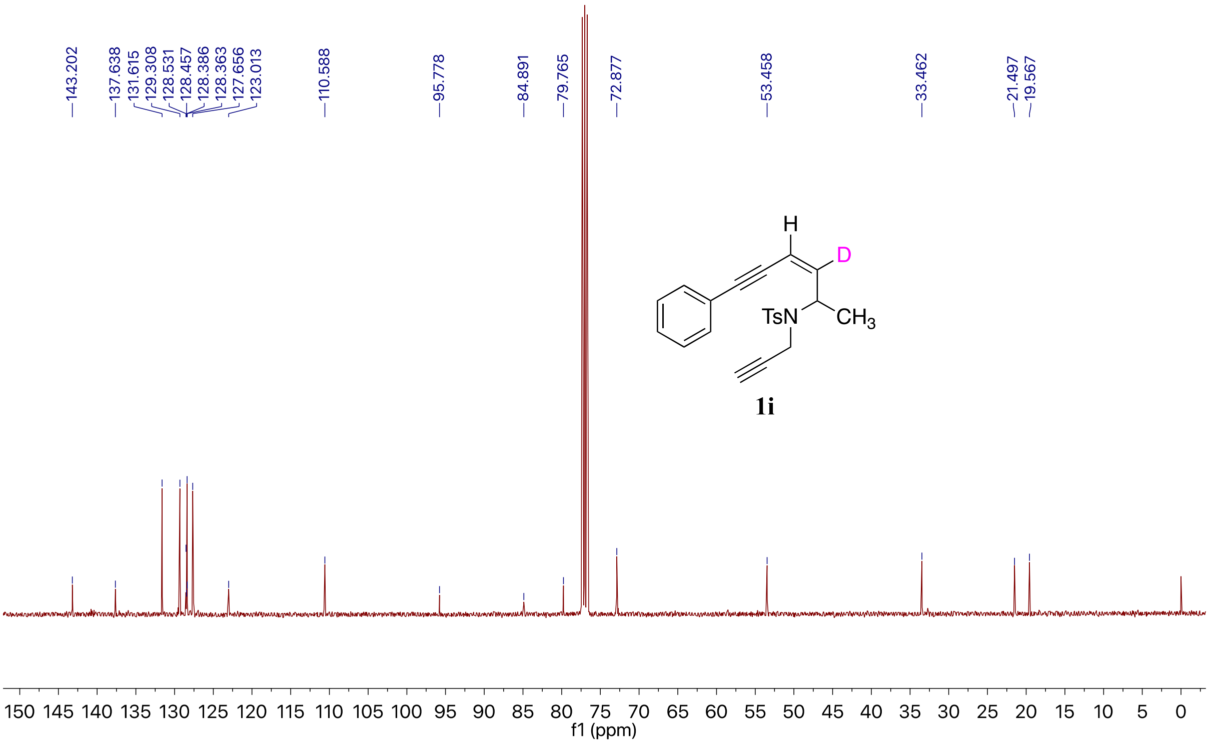

^1H NMR 500 MHz, $CDCl_3$

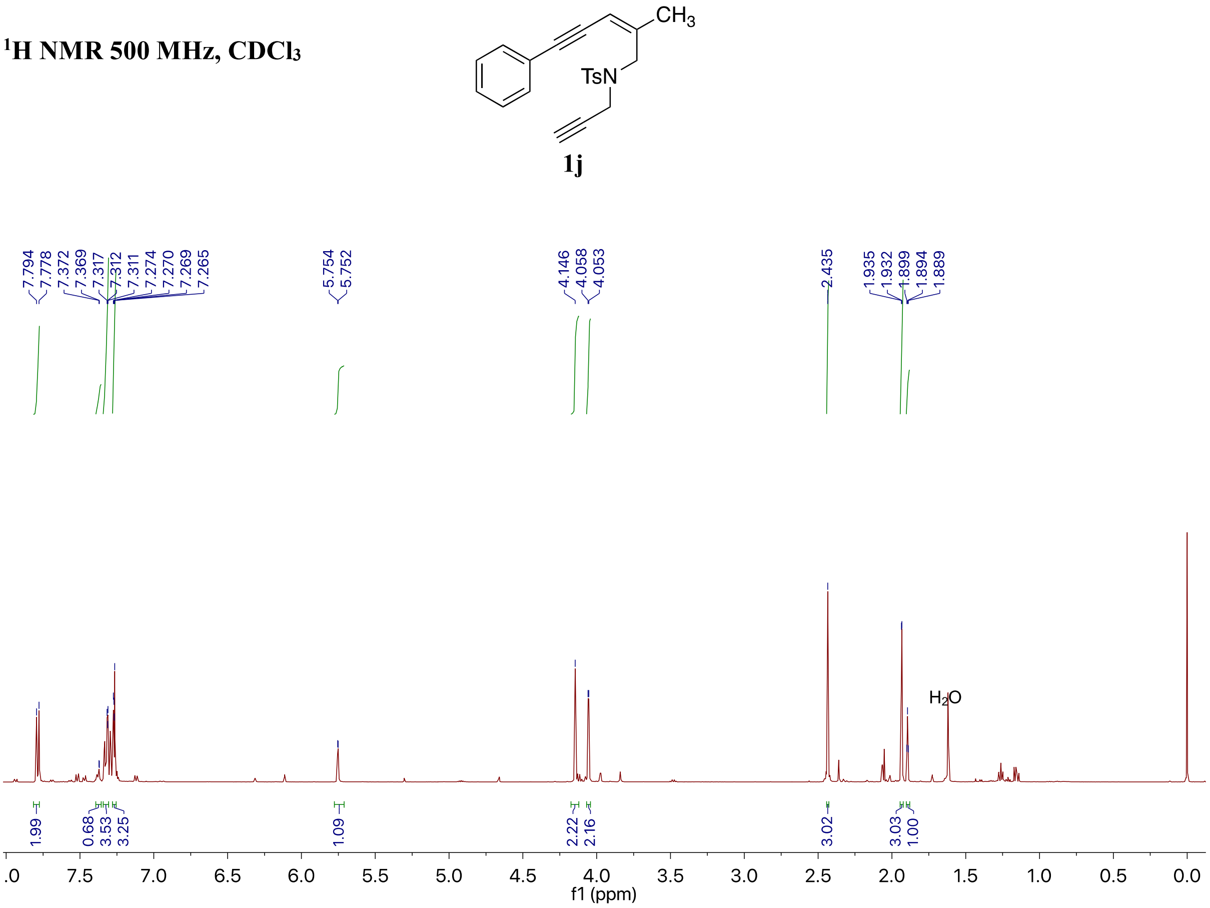

^{13}C NMR, 126 MHz, $CDCl_3$

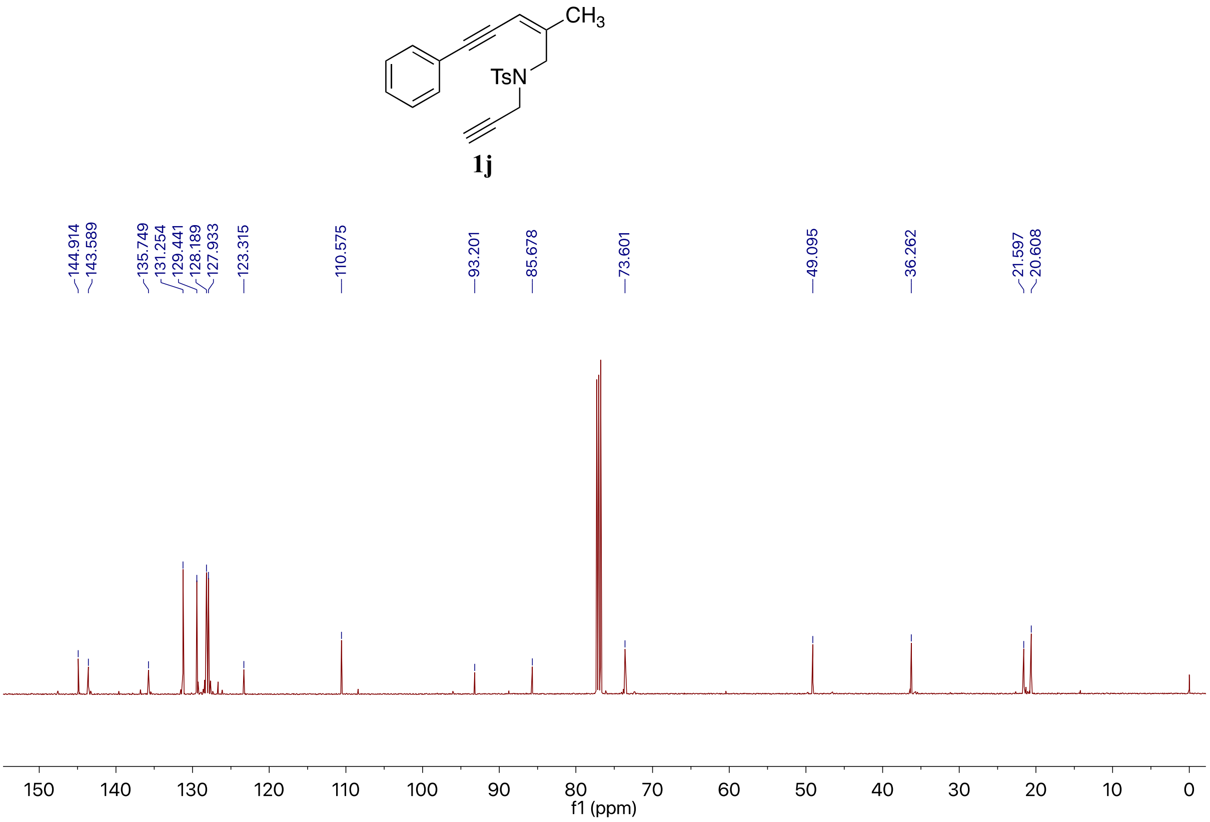

^{1}H NMR, 500 MHz, $CDCl_3$

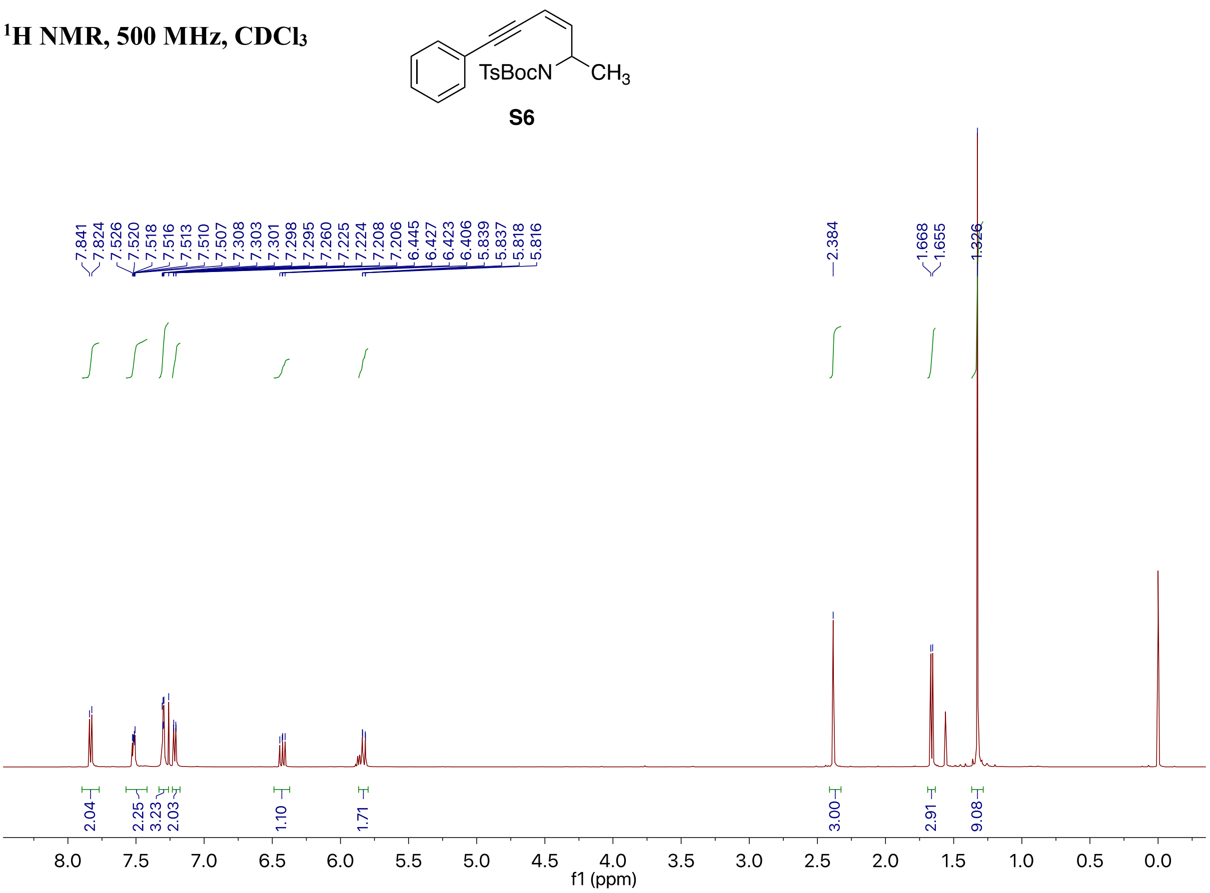

^{13}C NMR, 126 MHz, $CDCl_3$

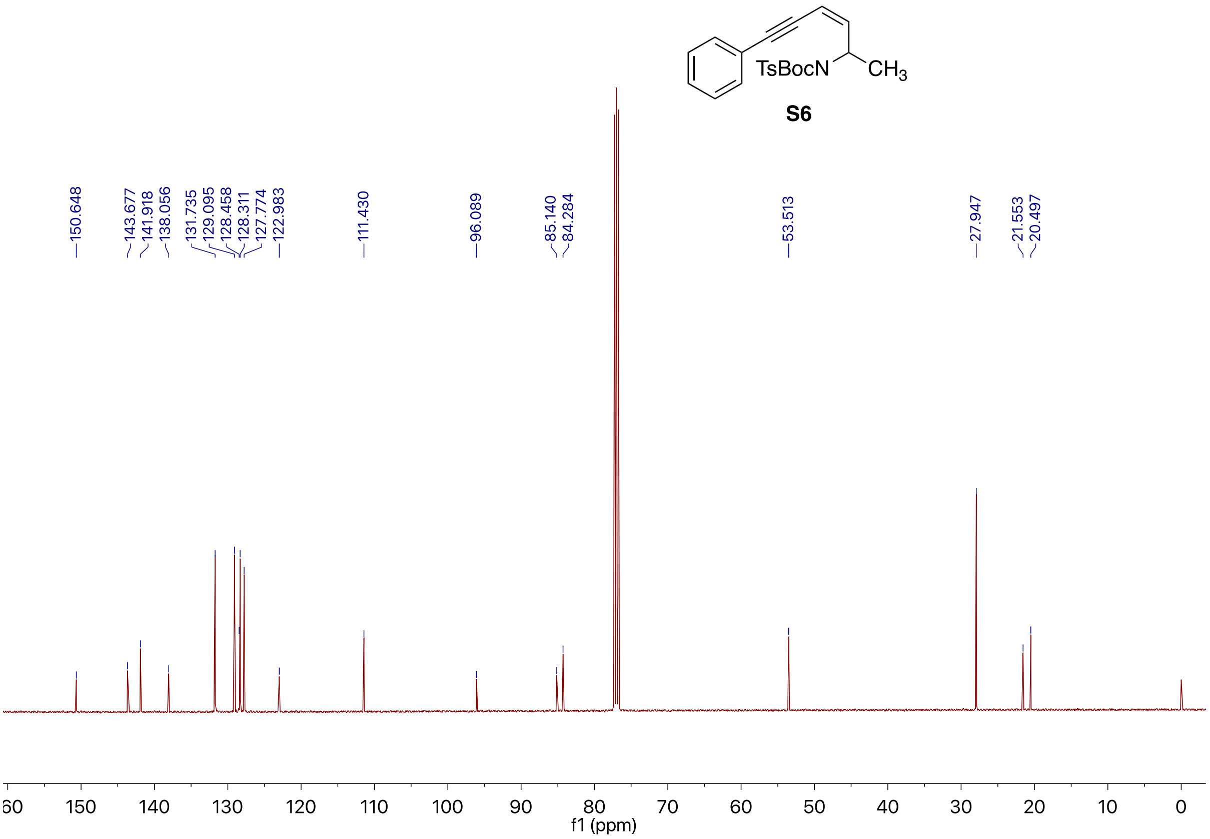

^{1}H NMR 500 MHz, $CDCl_3$

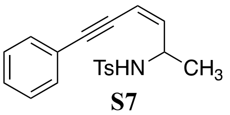

S7

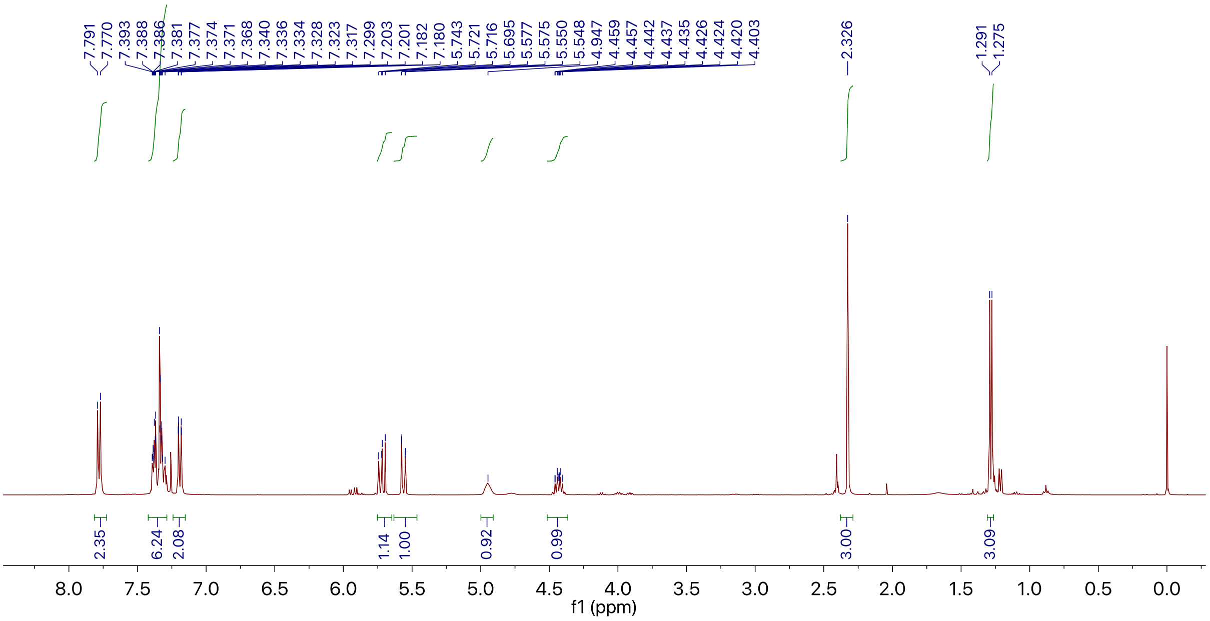

^{13}C NMR 101 MHz, $CDCl_3$

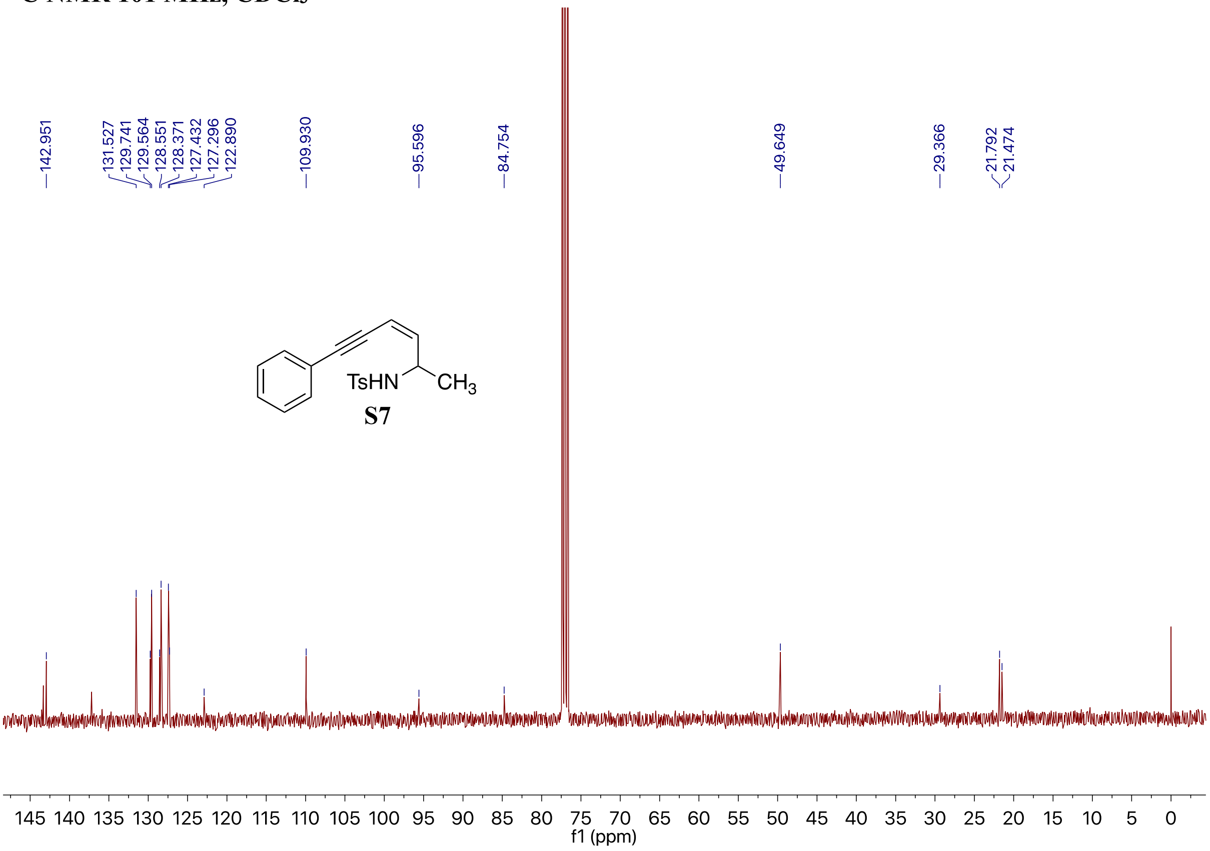

^{1}H NMR, 500 MHz, $CDCl_3$

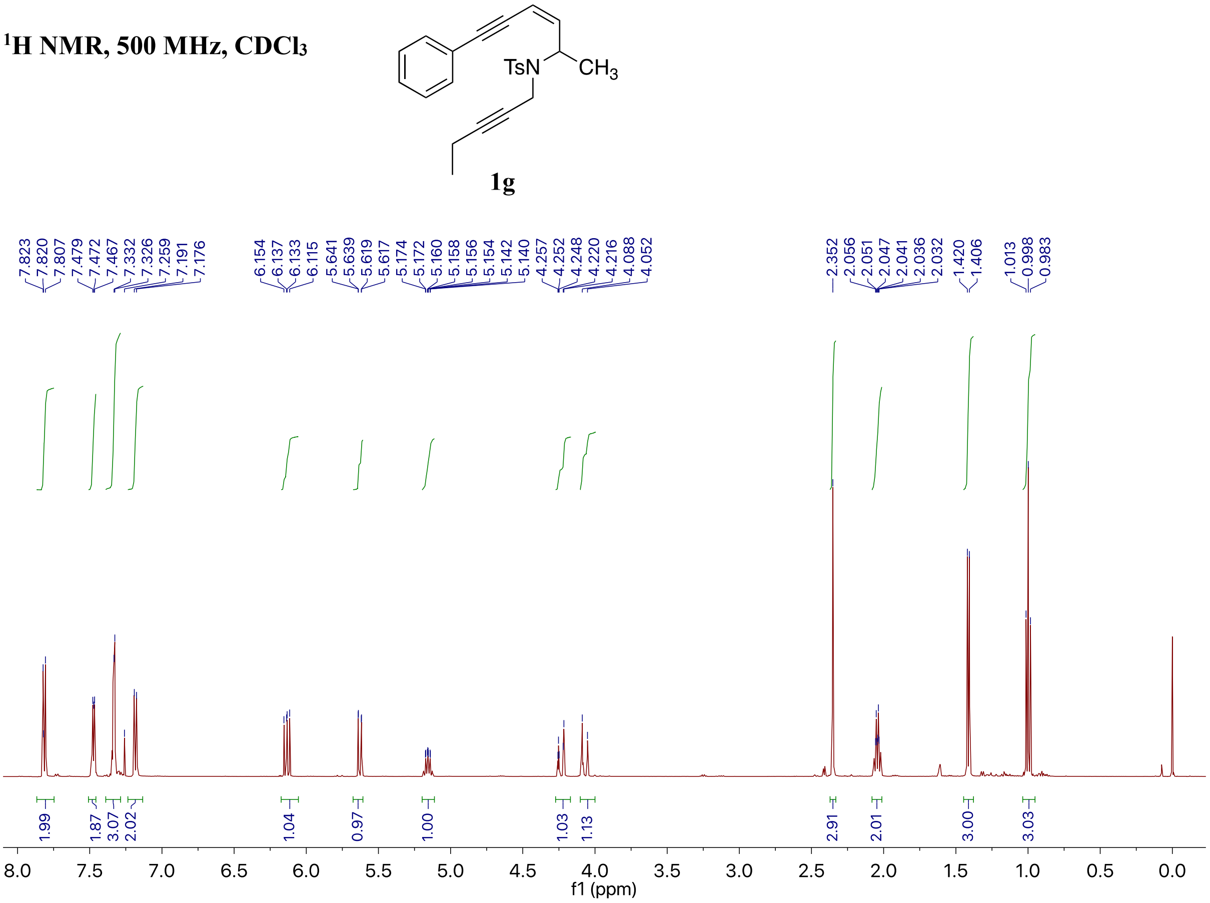

^{13}C NMR, 126 MHz, $CDCl_3$

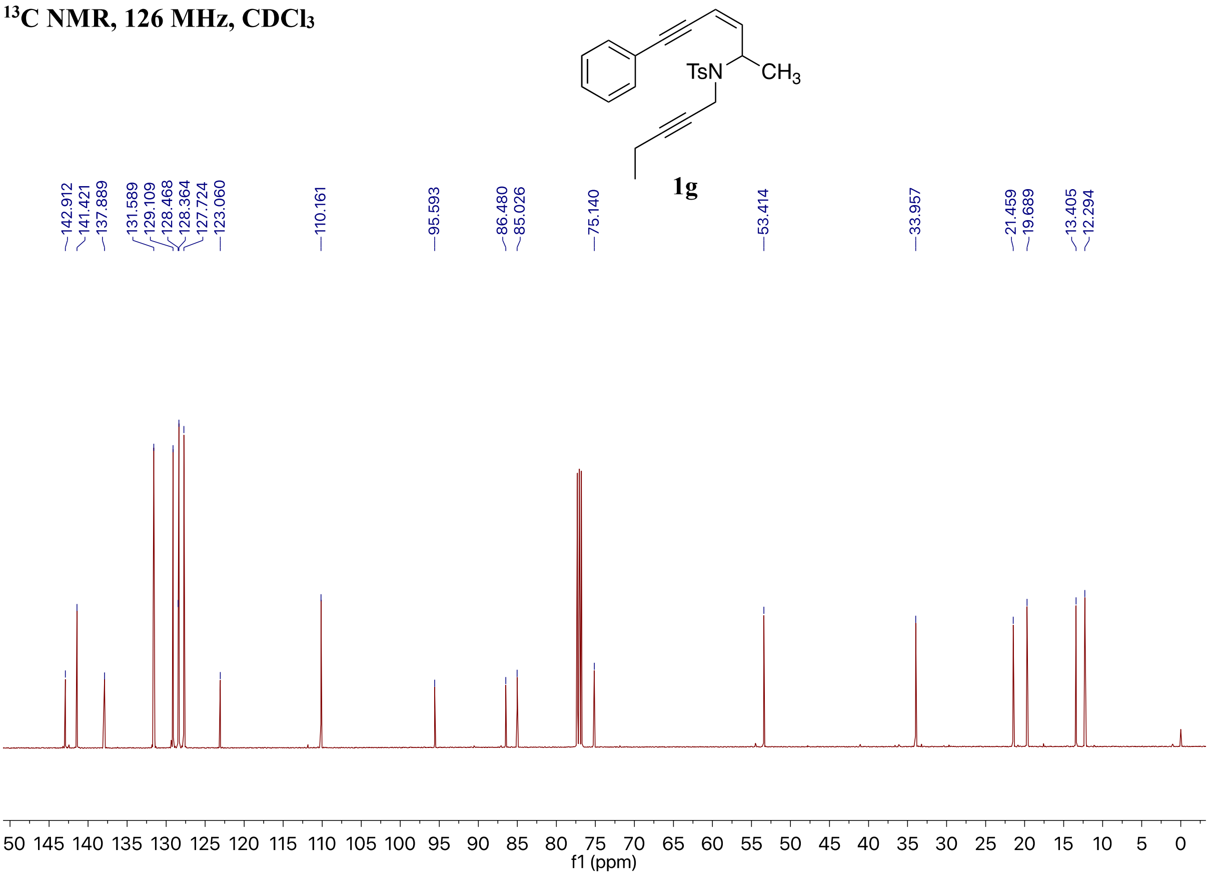

^{1}H NMR, 500 MHz, CDCl$_3$

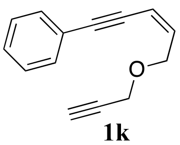

1k

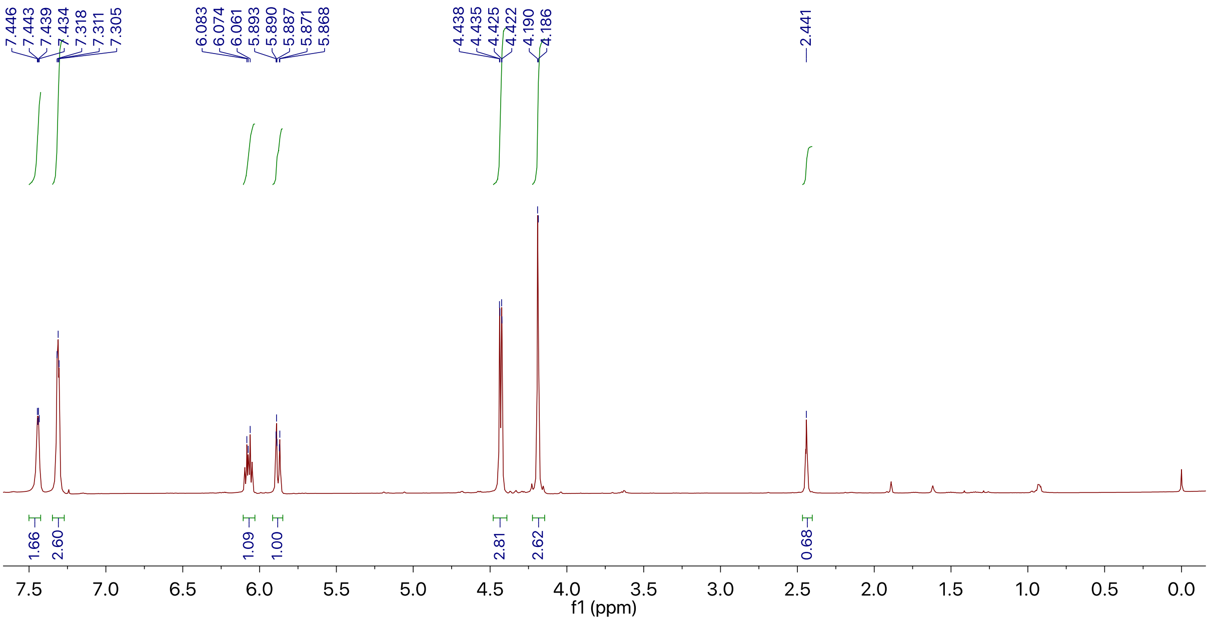

^{13}C NMR, 126 MHz, CDCl$_3$

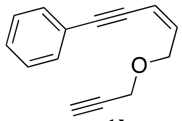

1k

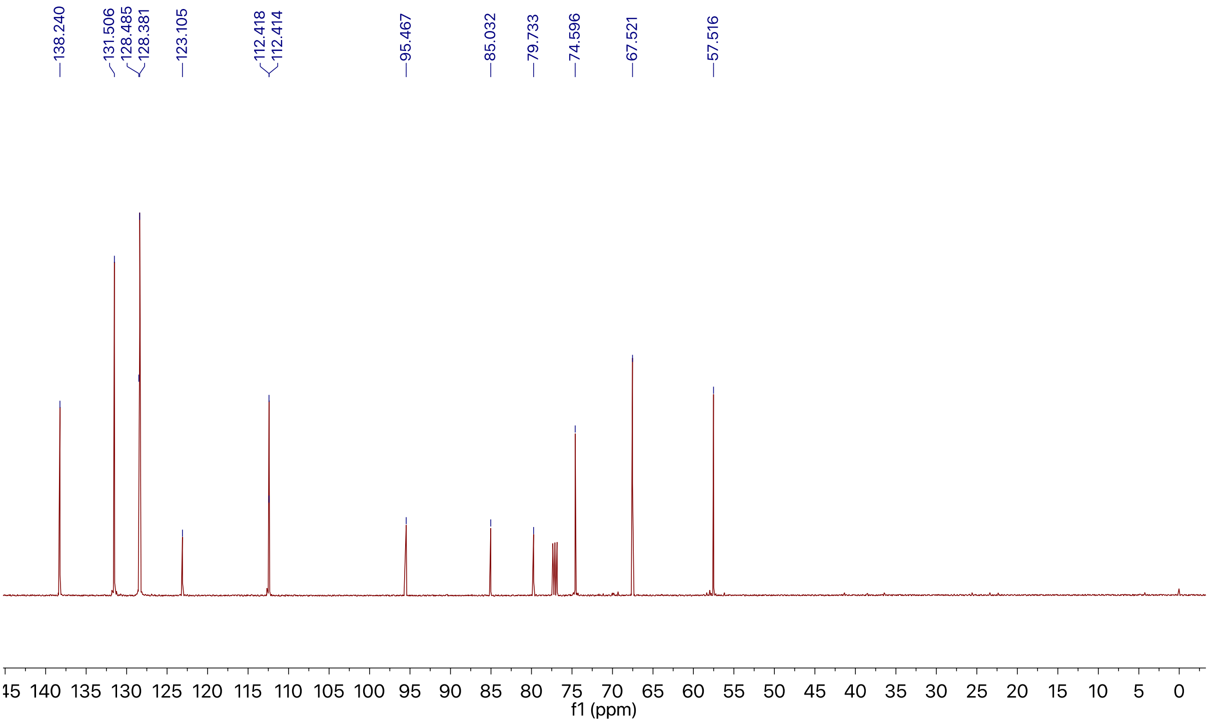

^{1}H NMR 500 MHz, CDCl$_3$

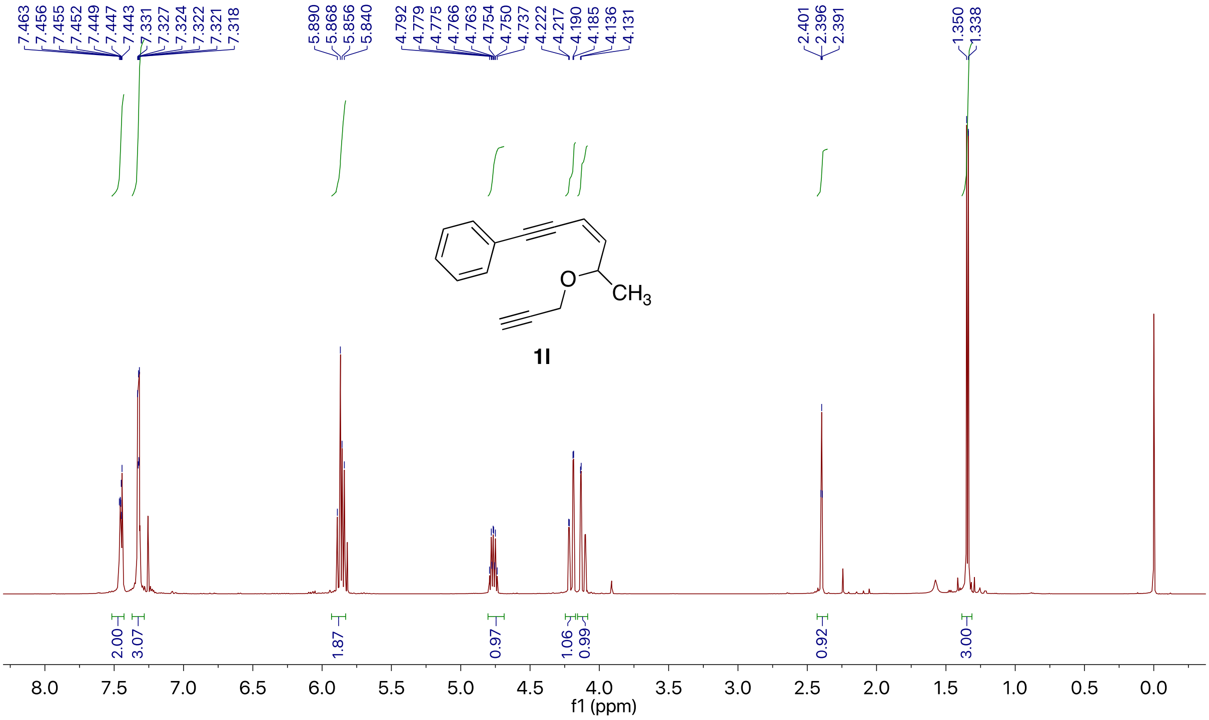

^{13}C NMR 500 MHz, CDCl$_3$

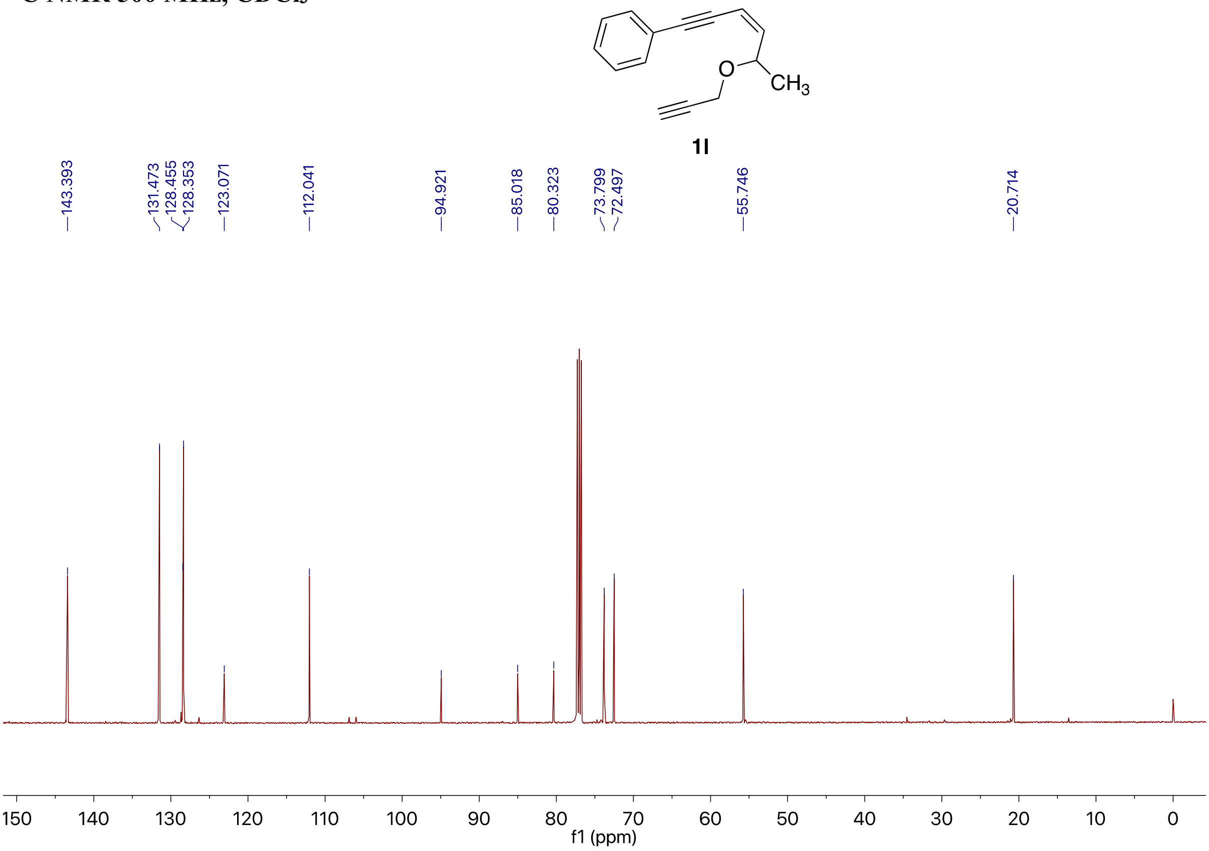

^{1}H NMR, 500 MHz, $CDCl_3$

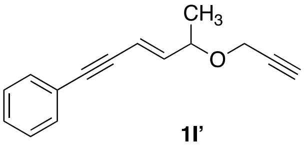

1l'

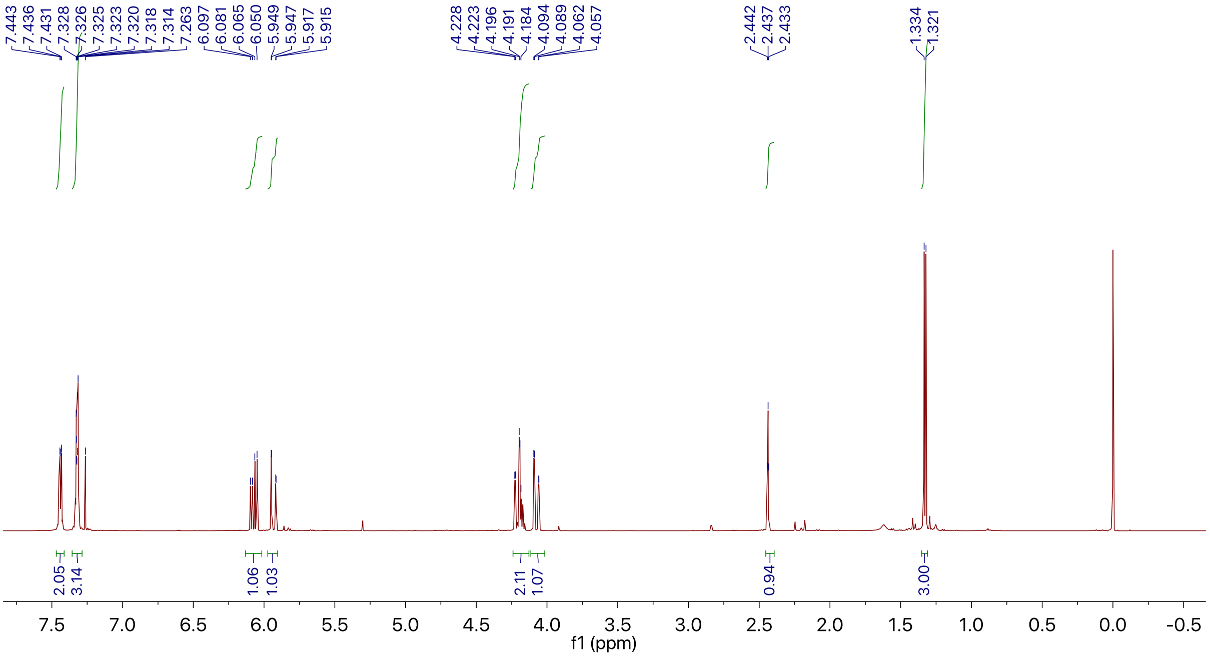

^{13}C NMR, 126 MHz, $CDCl_3$

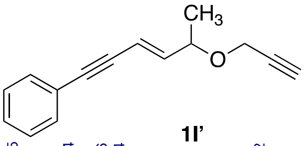

1l'

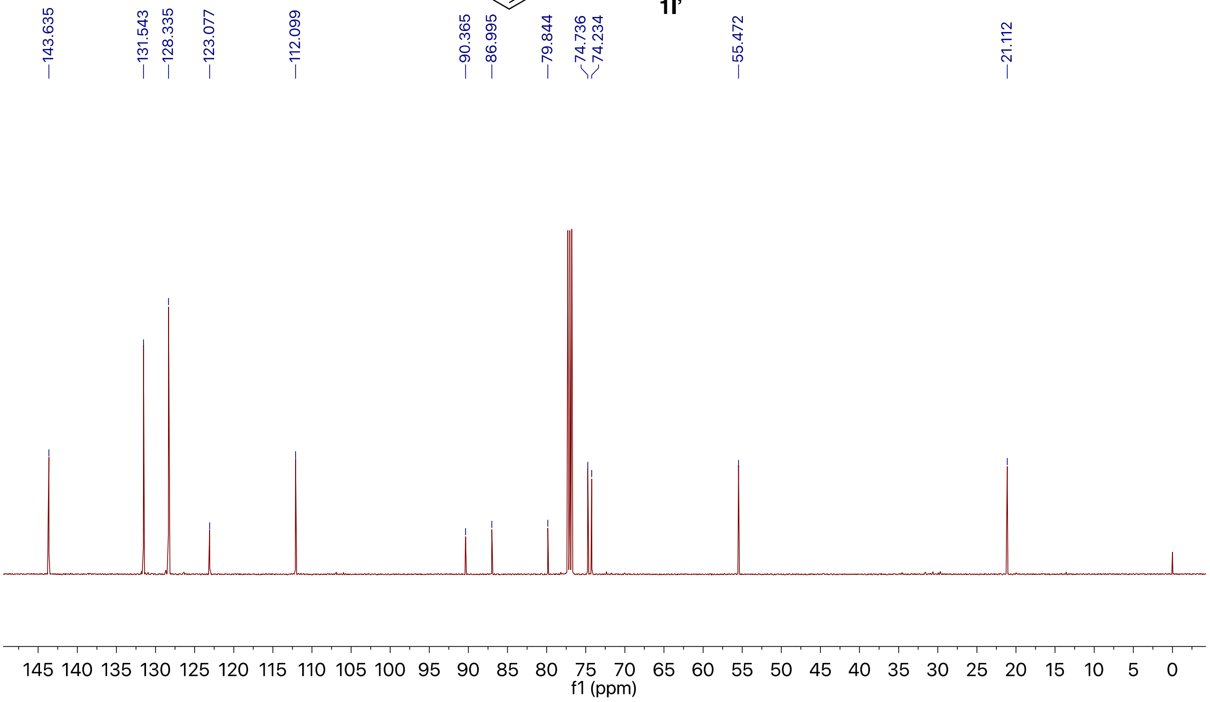

^{1}H NMR, 500 MHz, $CDCl_3$

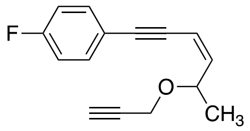

1m

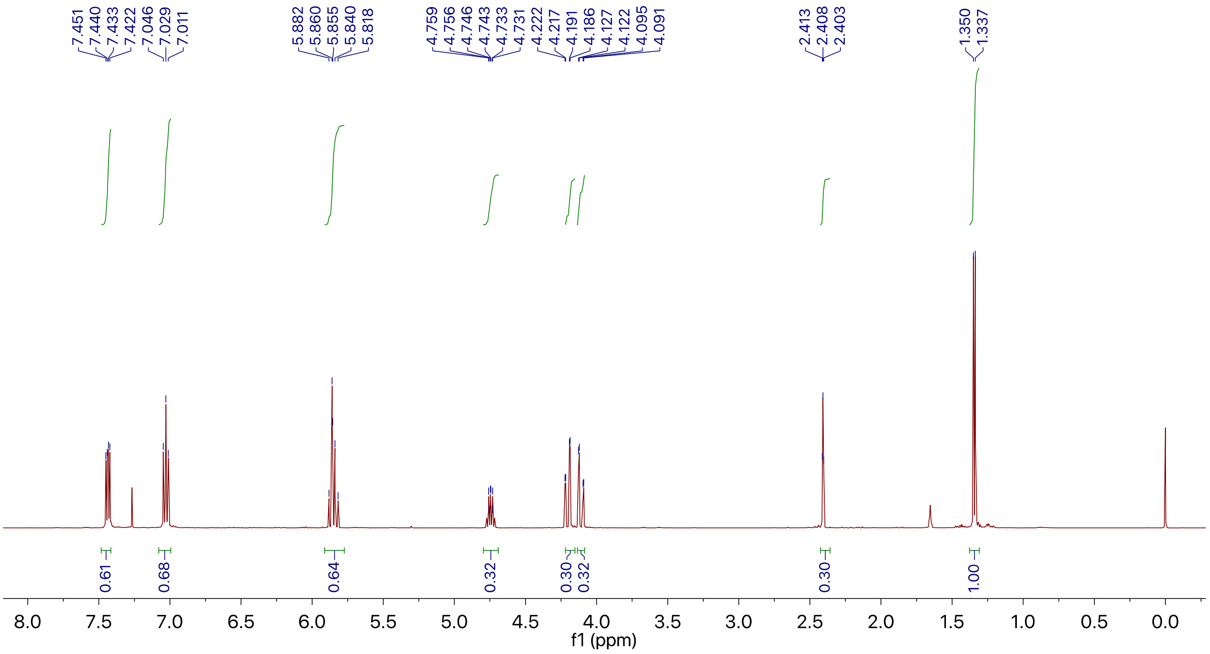

^{13}C NMR, 126 MHz, $CDCl_3$

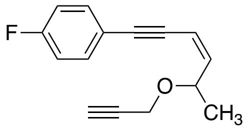

1m

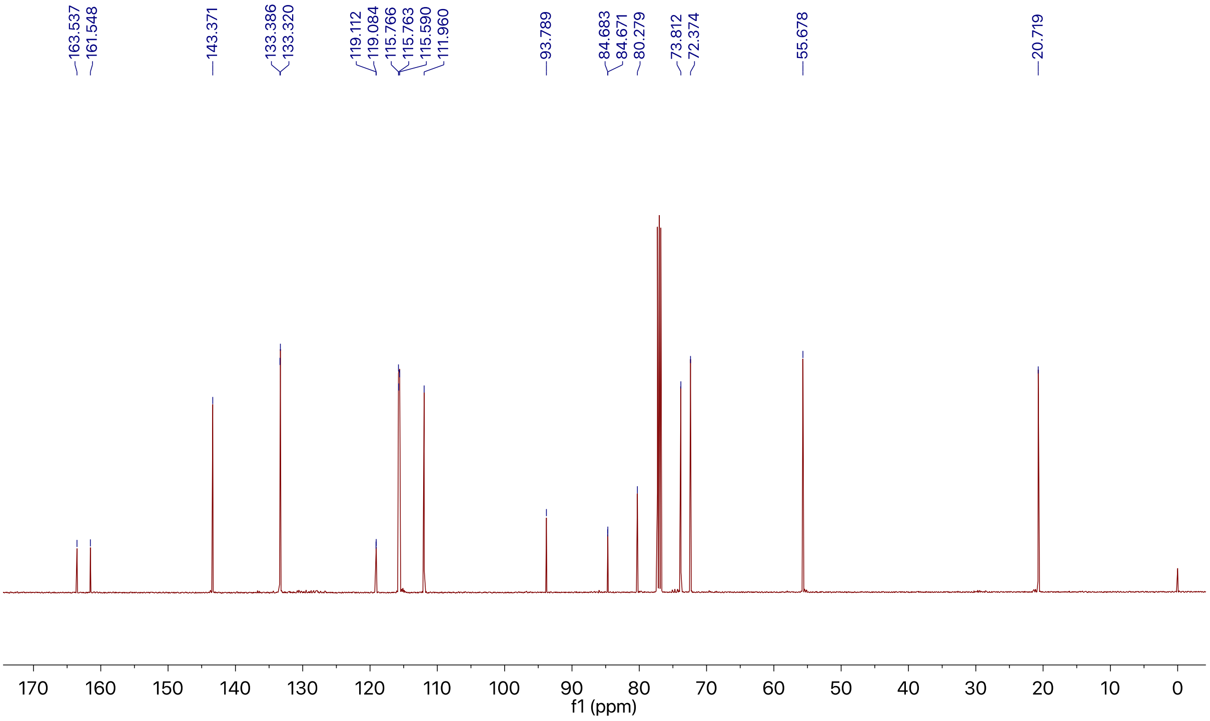

^{1}H NMR, 400 MHz, CDCl3

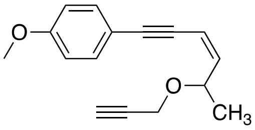

1n

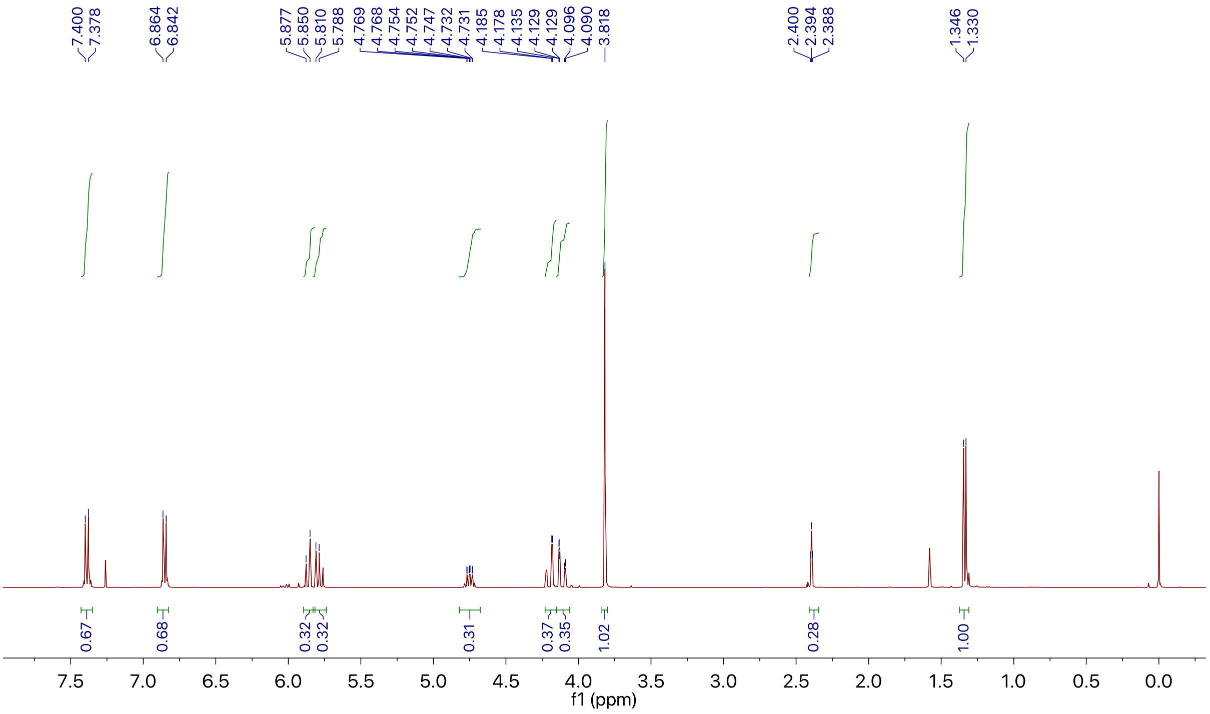

^{13}C NMR, 101 MHz, CDCl3

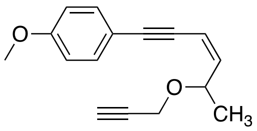

1n

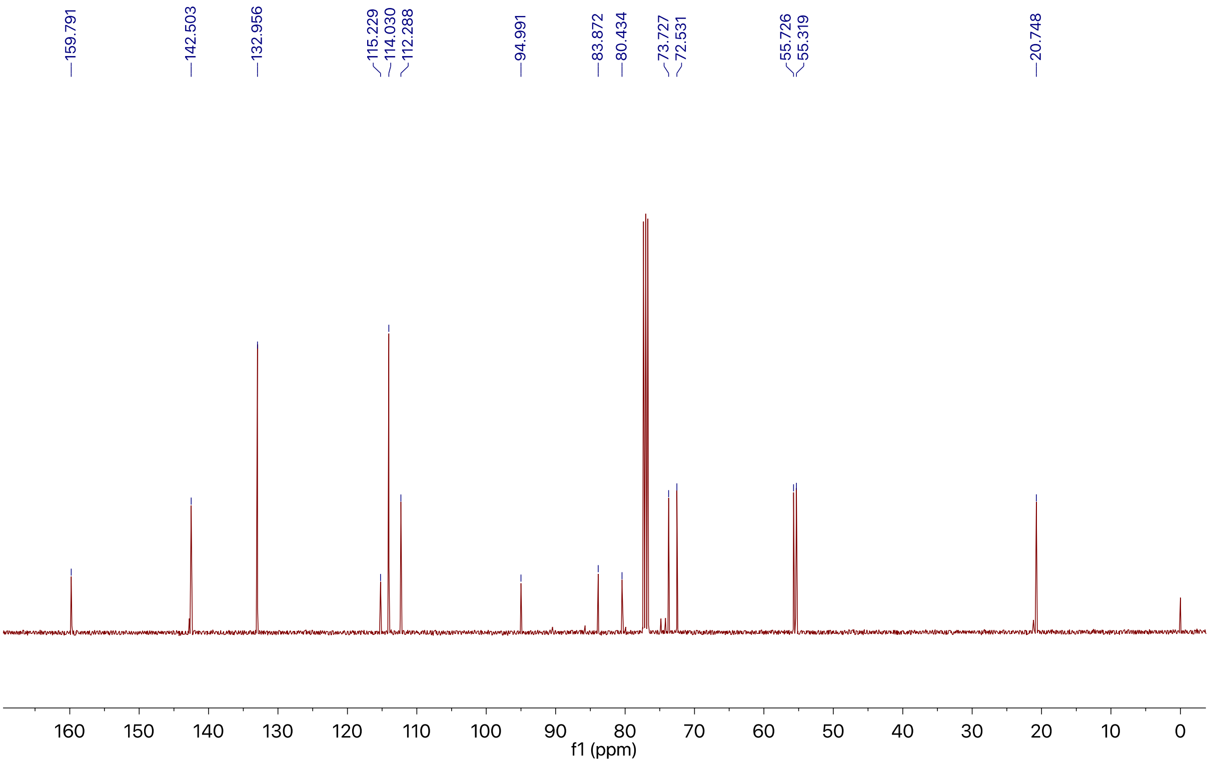

^{1}H NMR, 500 MHz, $CDCl_3$

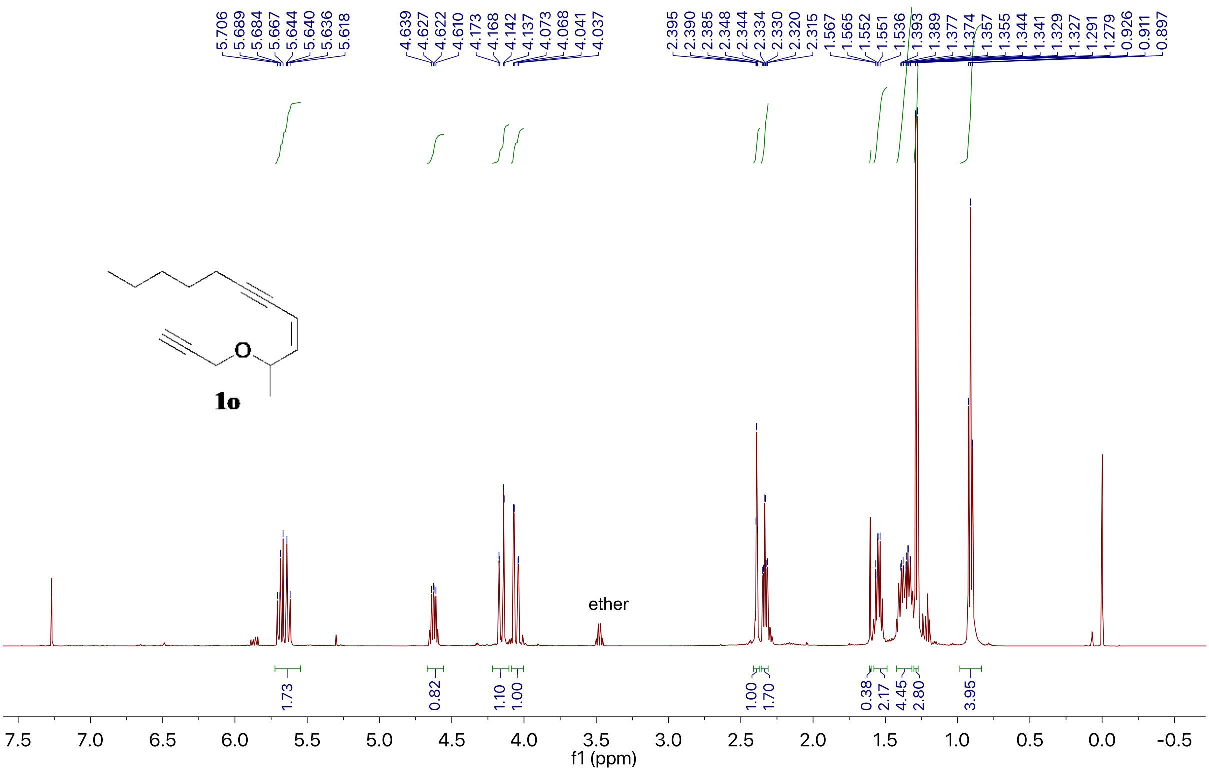

^{13}C NMR, 126 MHz, $CDCl_3$

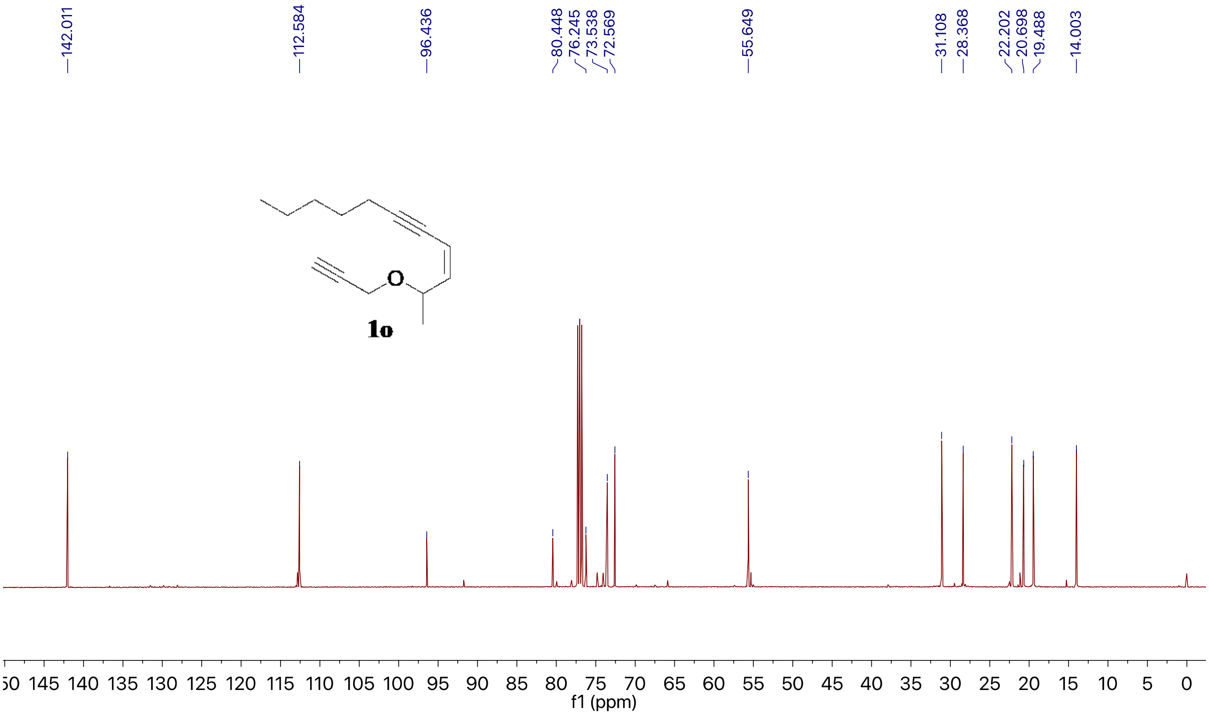

^{1}H NMR, 400 MHz, $CDCl_3$

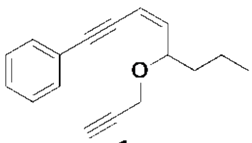

1p

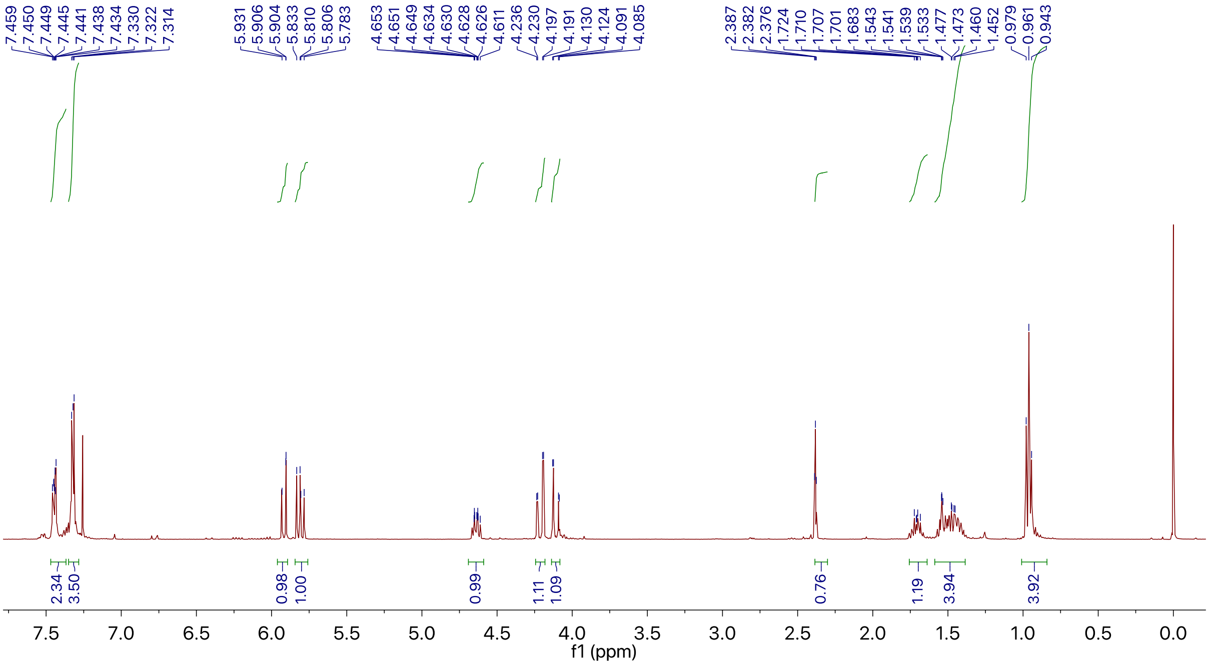

^{13}C NMR, 101 MHz, $CDCl_3$

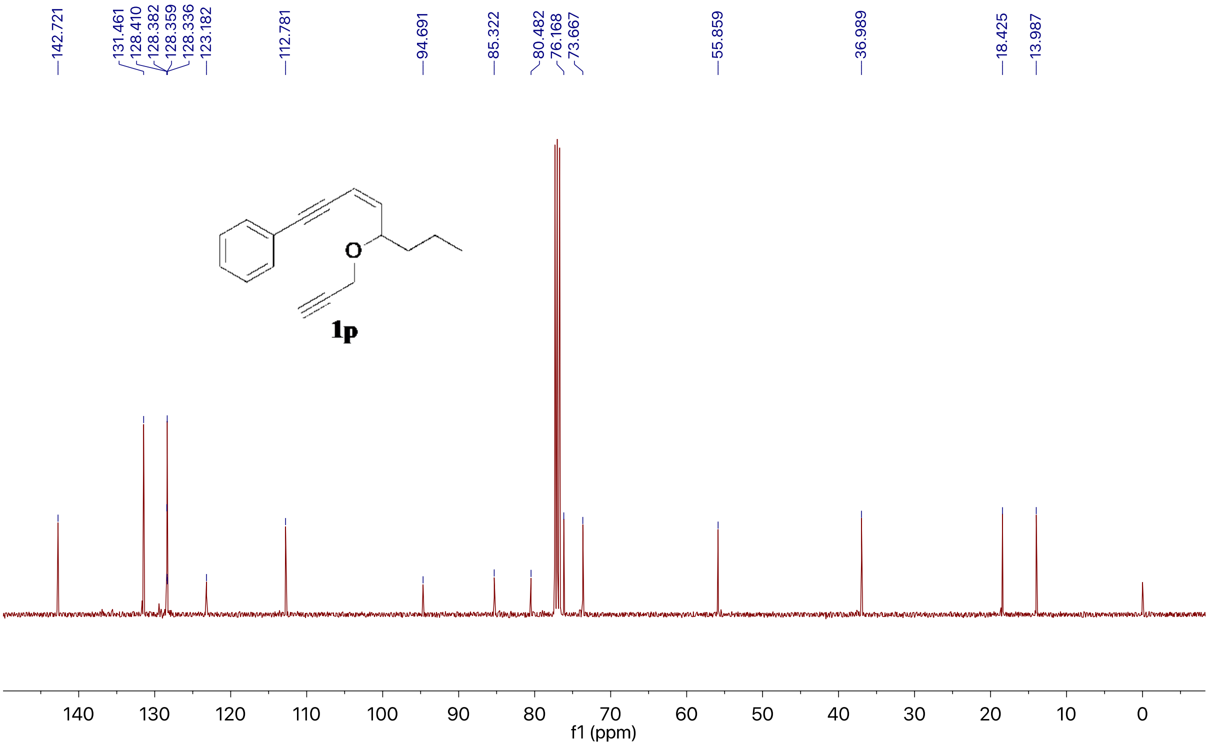

^{1}H NMR, 400 MHz, $CDCl_3$

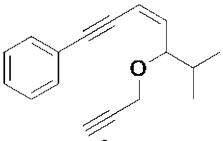

1q

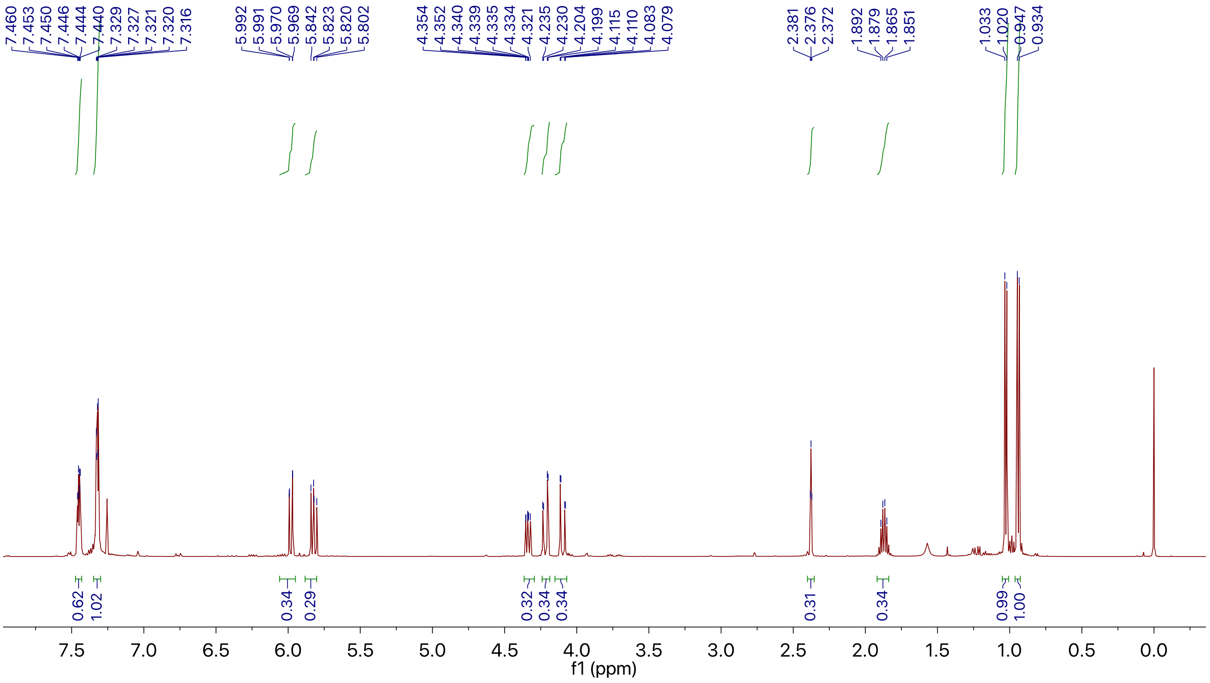

^{13}C NMR, 101 MHz, $CDCl_3$

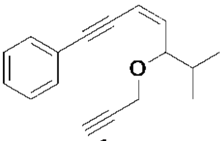

1q

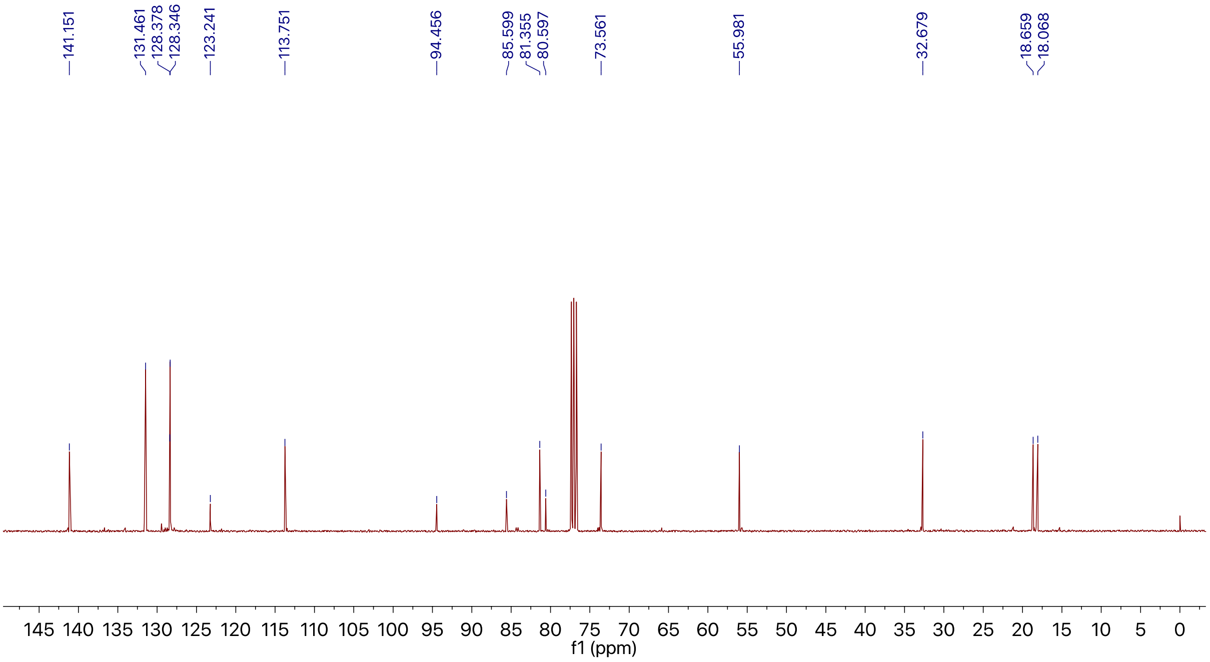

^{1}H NMR 500 MHz, $CDCl_3$

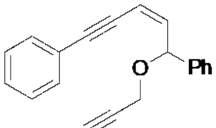

1r

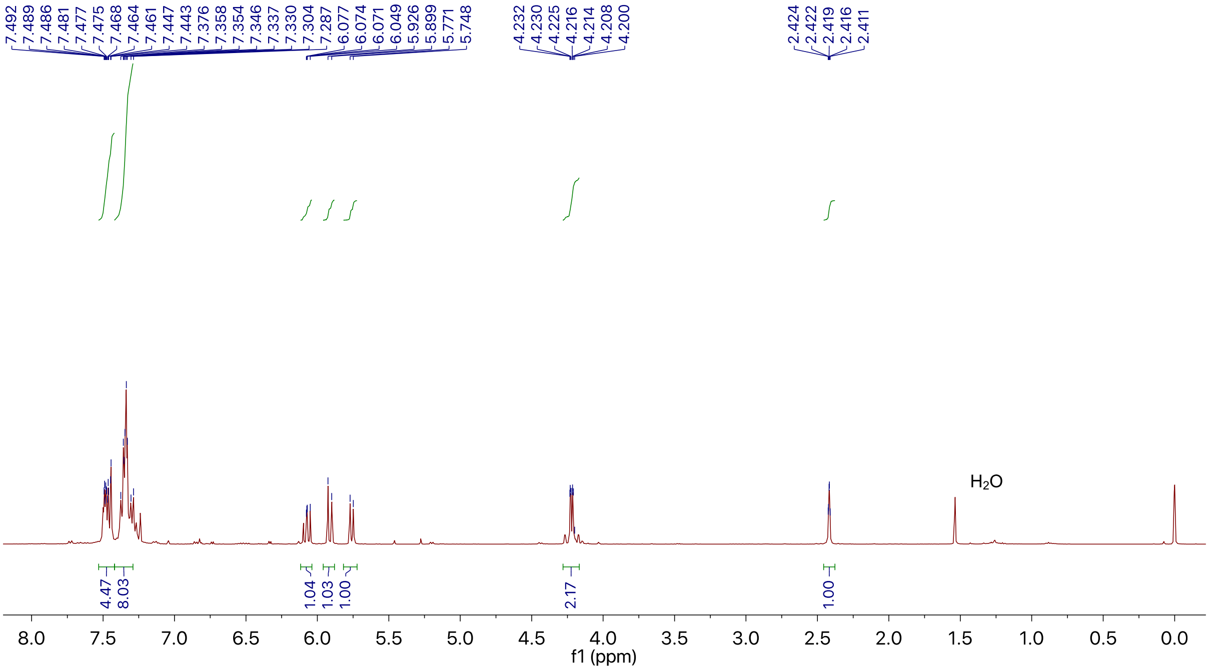

^{13}C NMR 126 MHz, $CDCl_3$

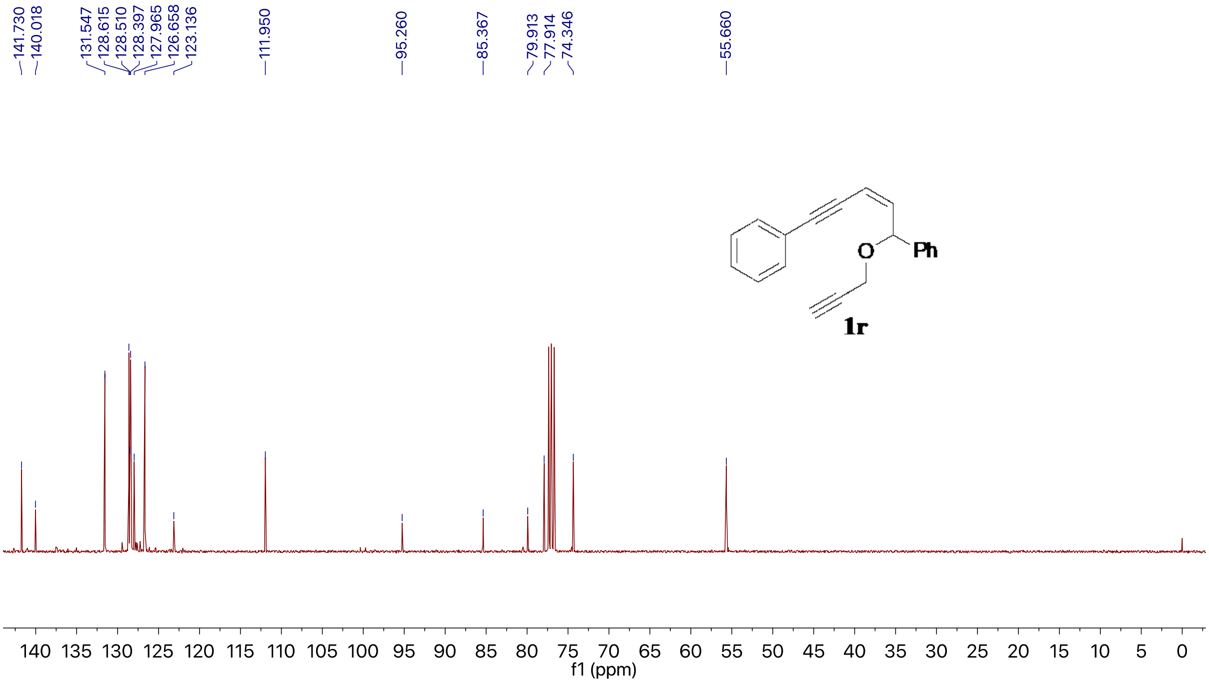

^{1}H NMR, 400 MHz, $CDCl_3$

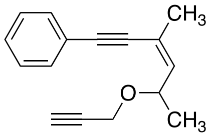

1s

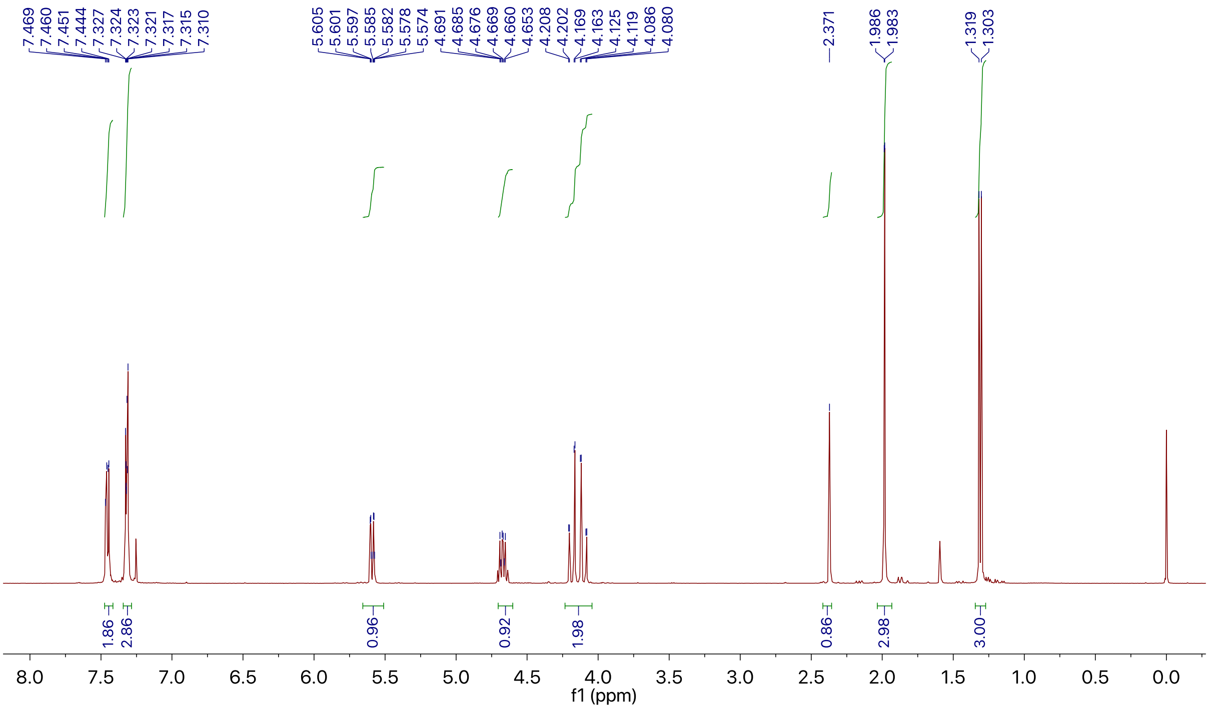

^{13}C NMR, 101 MHz, $CDCl_3$

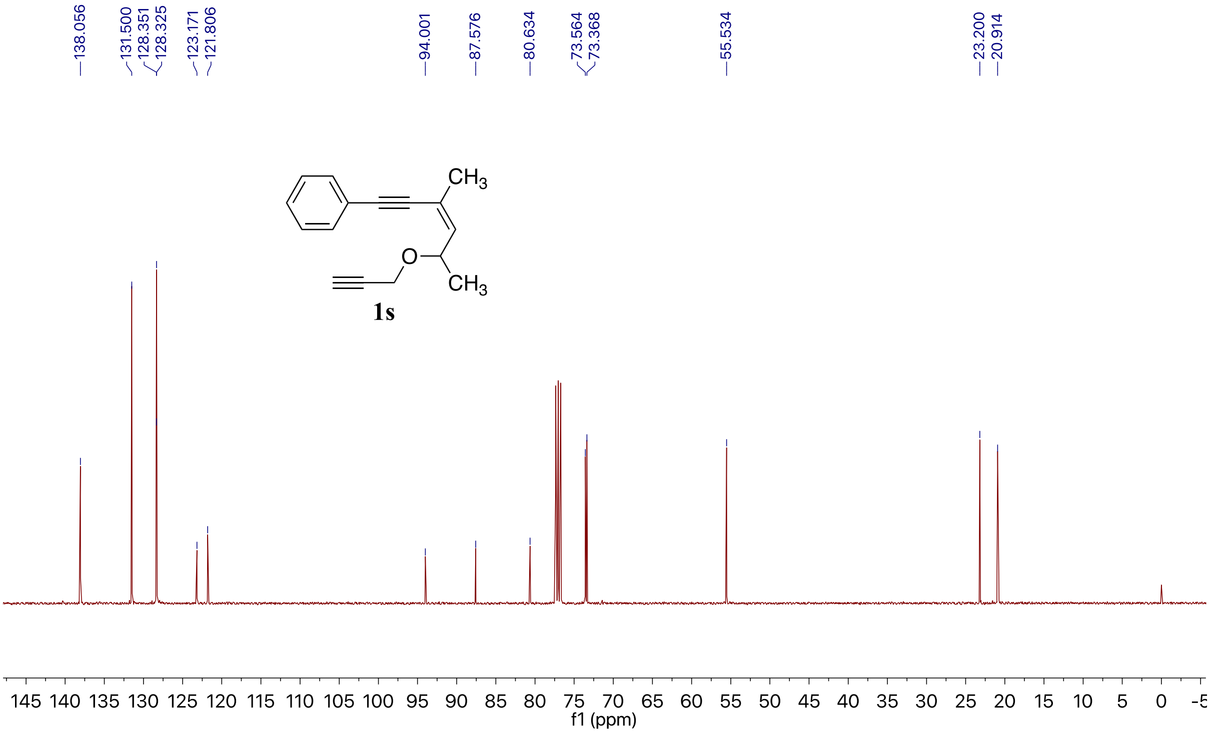

^{1}H NMR (500 MHz, $CDCl_3$)

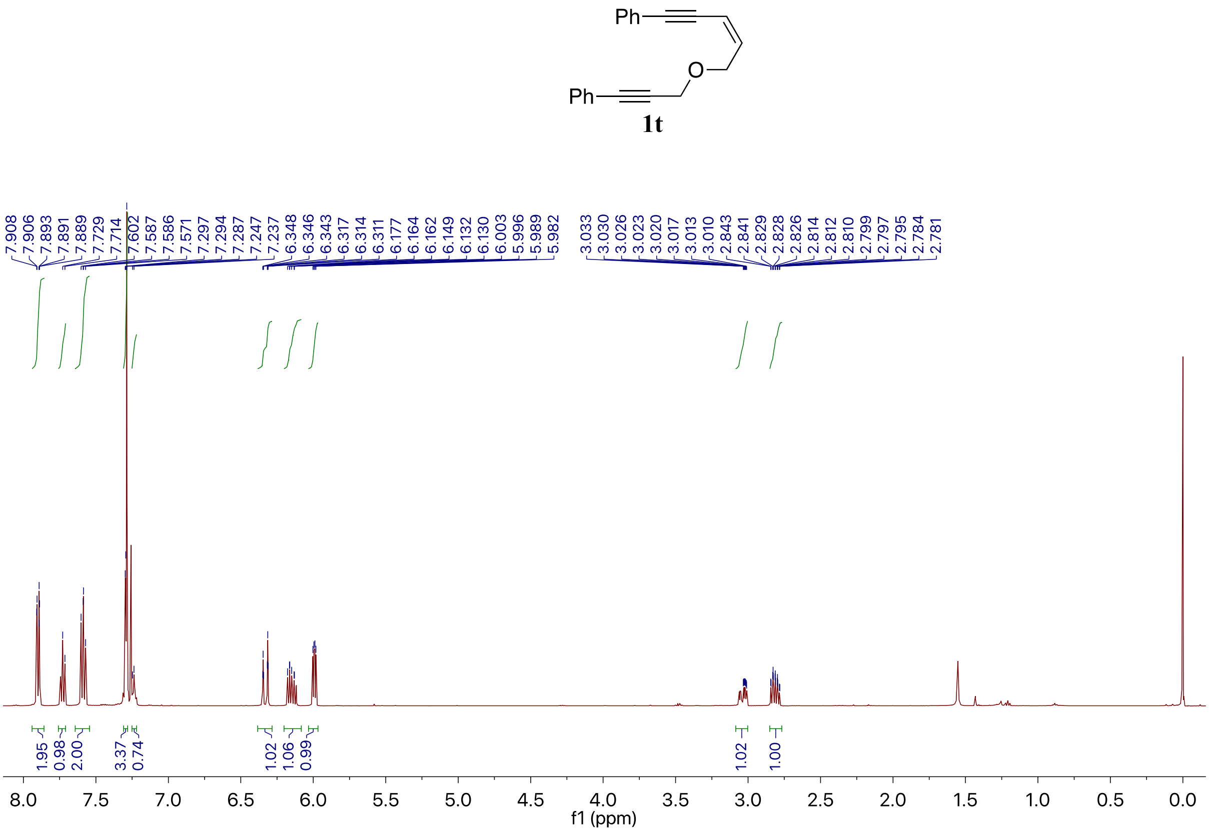

^{13}C NMR (126 MHz, $CDCl_3$)

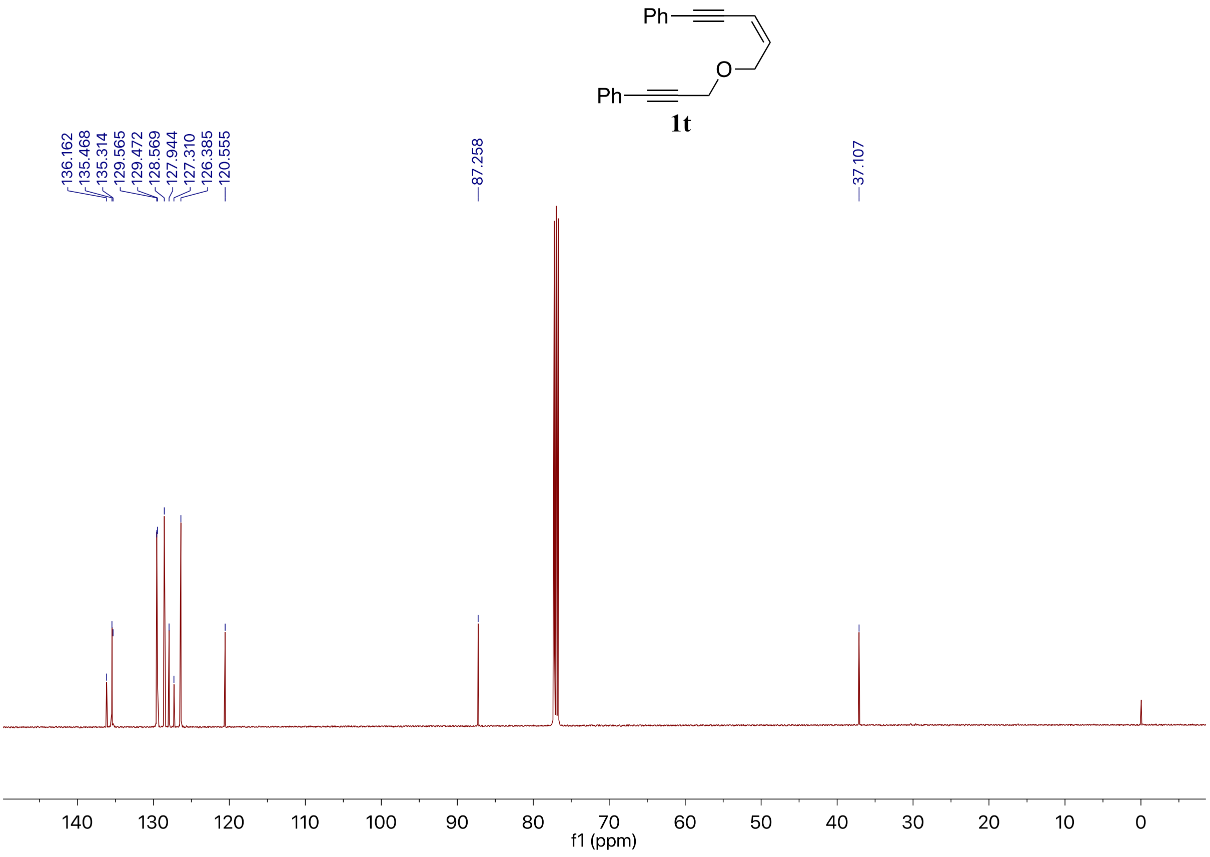

^{1}H NMR, 500 MHz, CDCl3

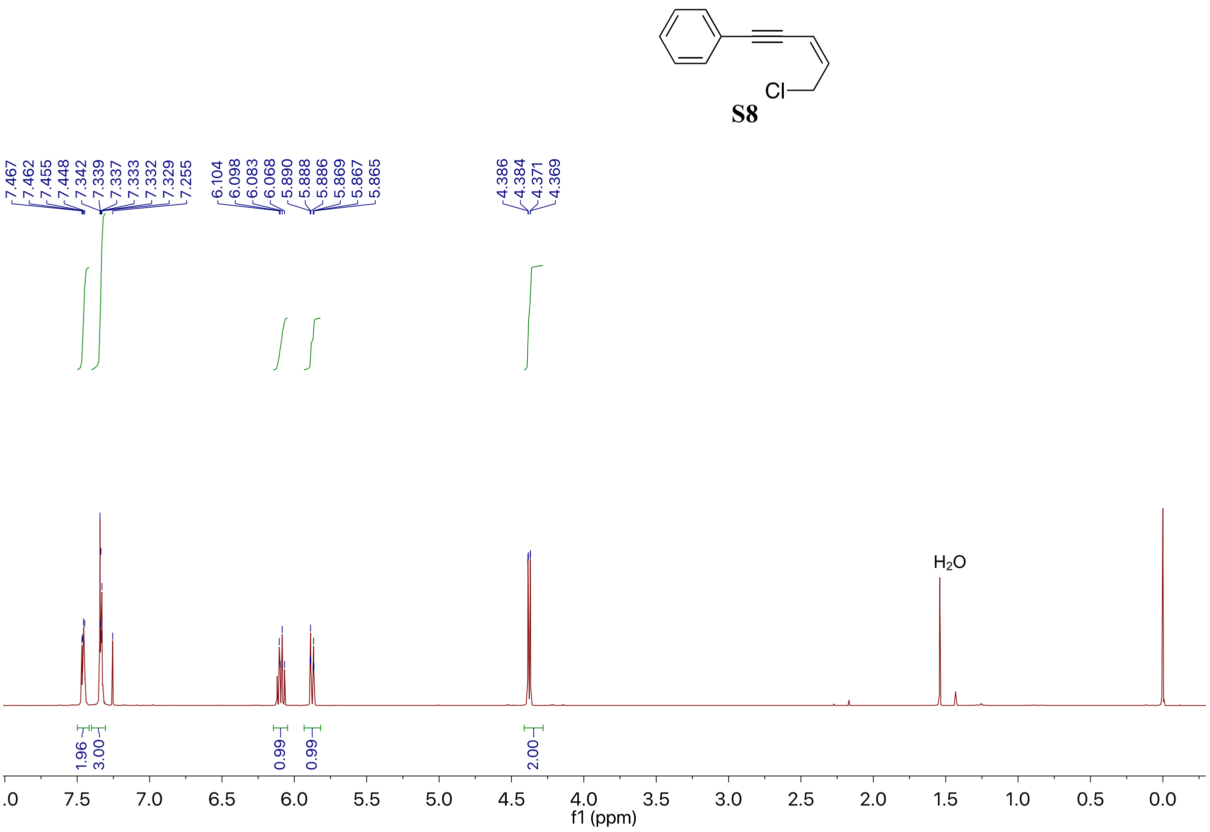

^{13}C NMR, 126 MHz, CDCl3

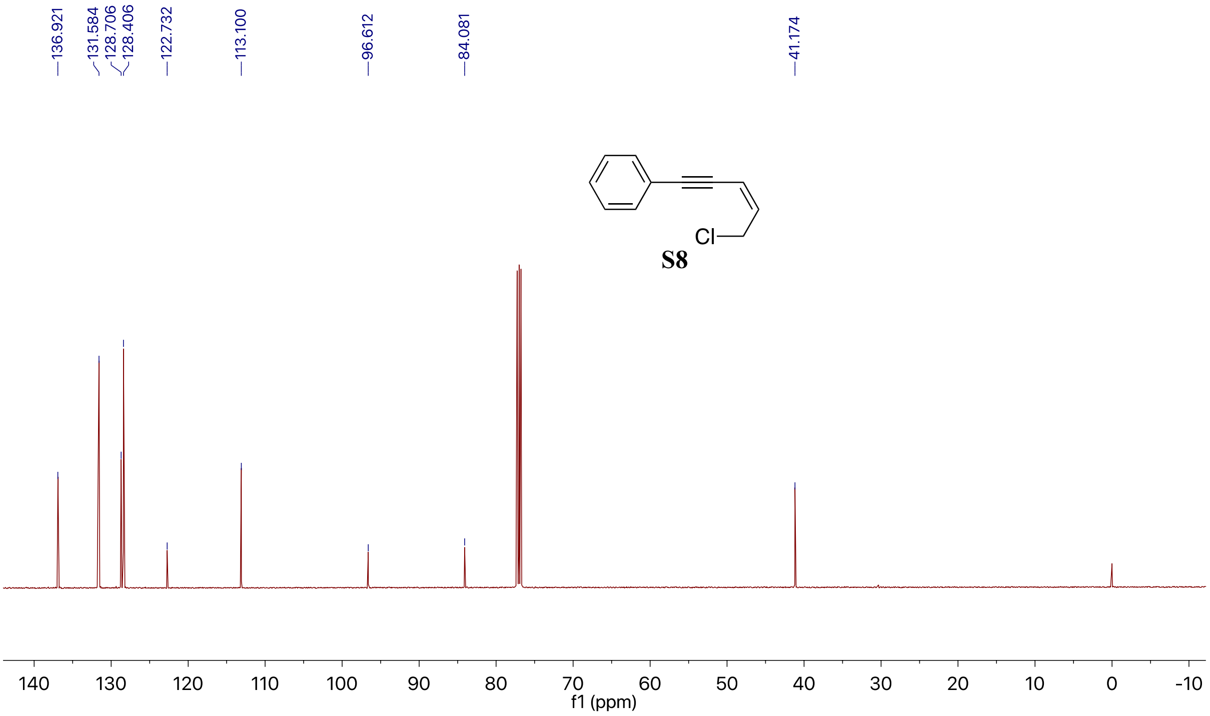

^1H NMR, 400 MHz, $CDCl_3$

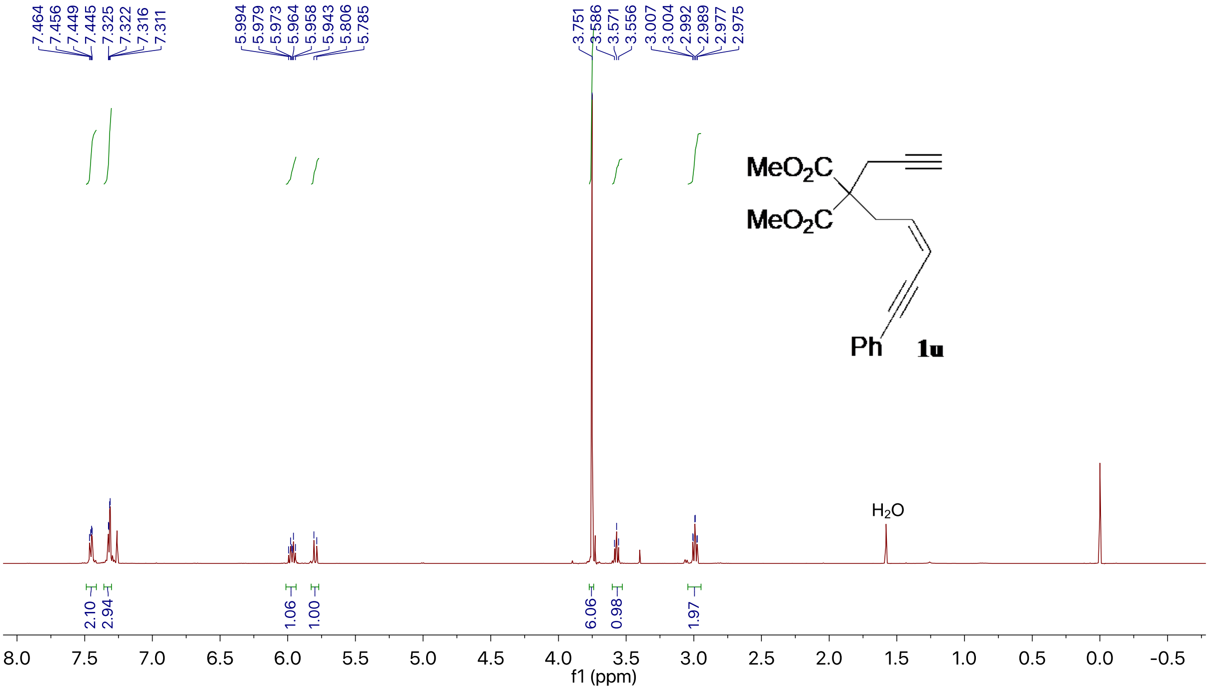

^{13}C NMR, 101 MHz, $CDCl_3$

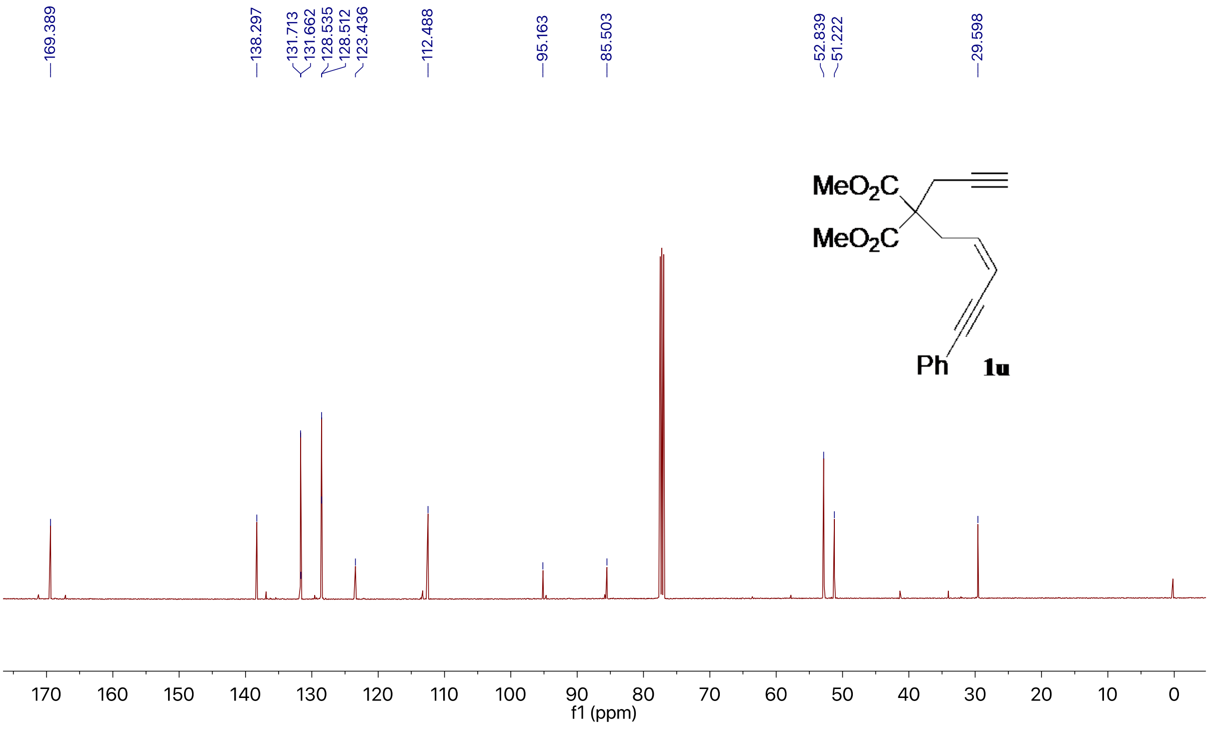

^{1}H NMR, 500 MHz, CDCl3

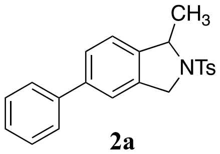

2a

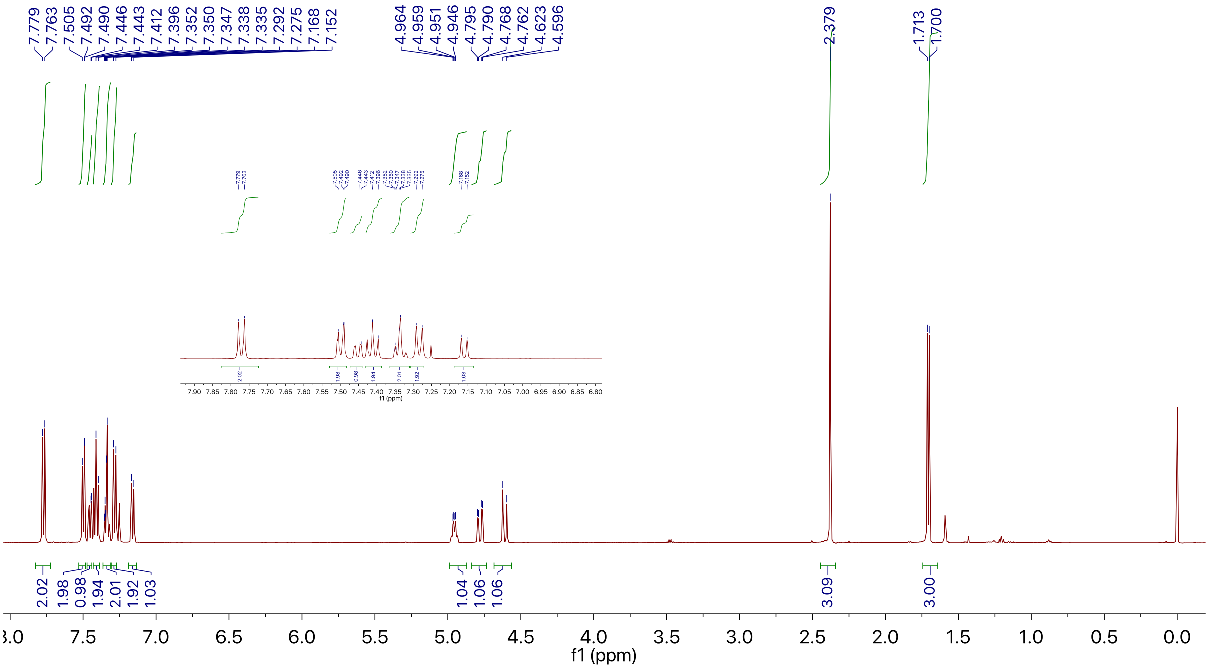

^{13}C NMR, 126 MHz, CDCl3

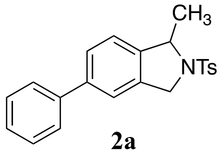

2a

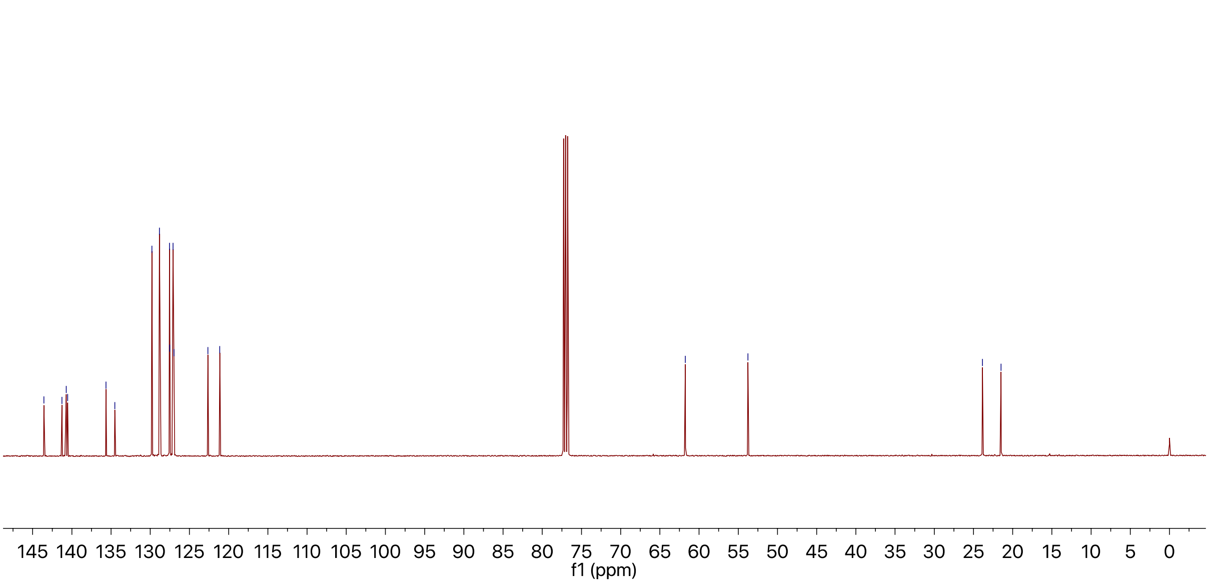

^{1}H NMR, 400 MHz, CDCl$_3$

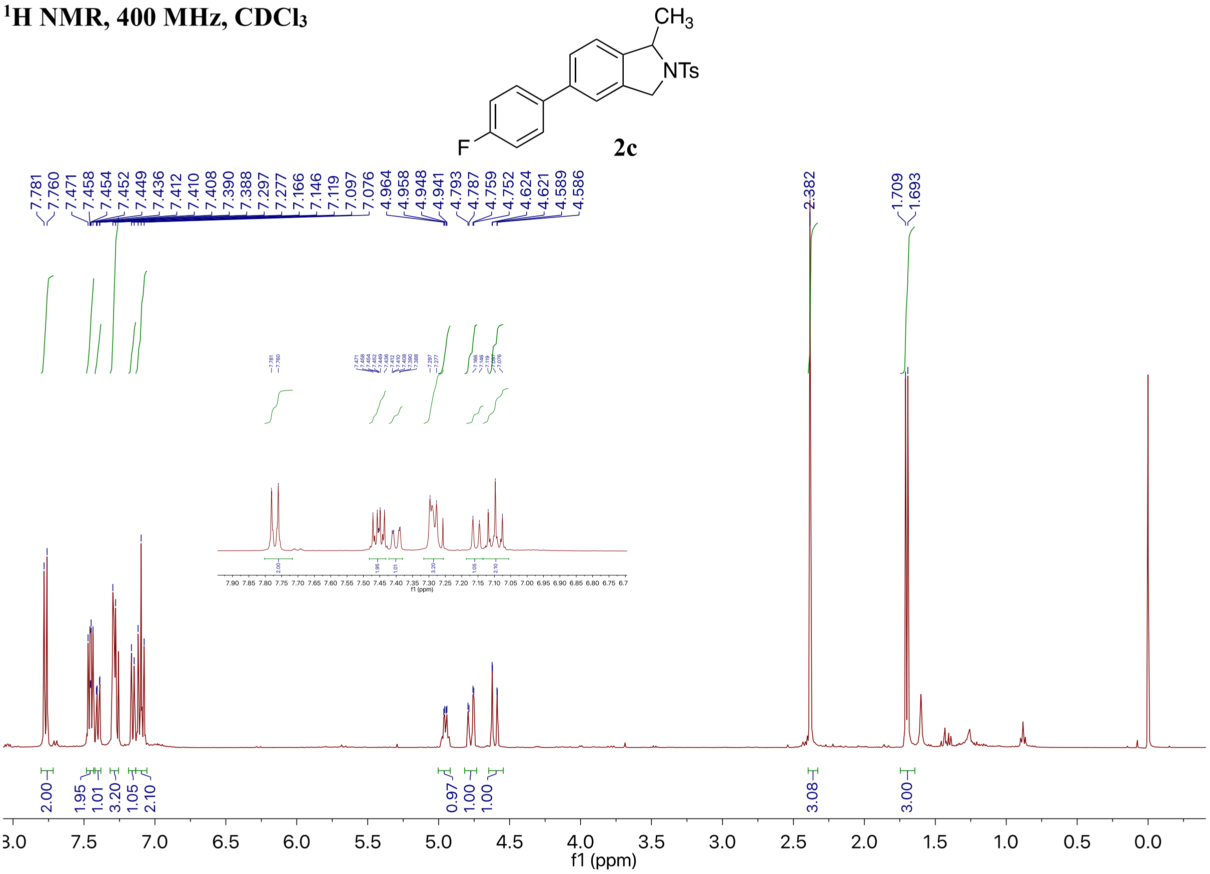

^{13}C NMR, 101 MHz, CDCl$_3$

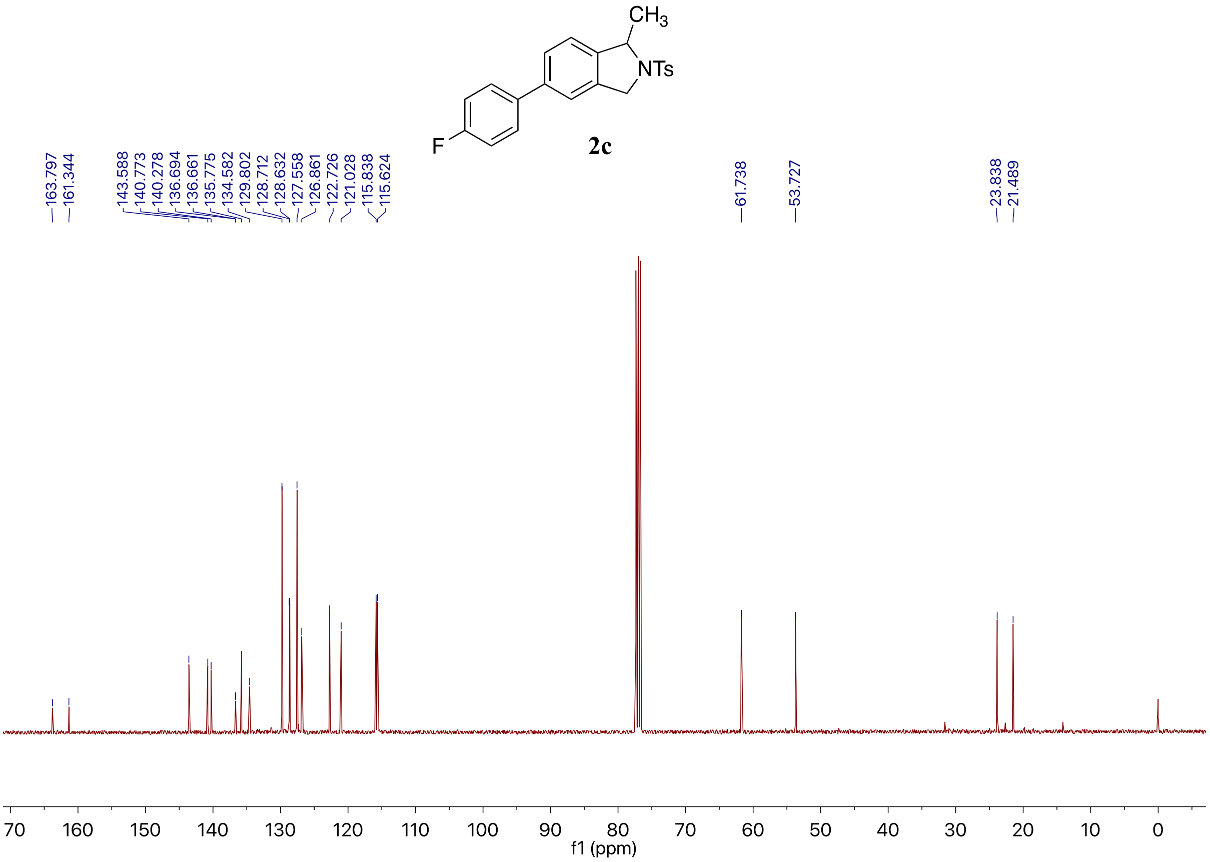

^{1}H NMR, 400 MHz, CDCl3

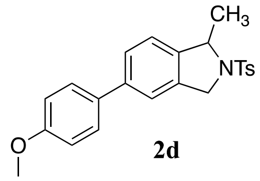

2d

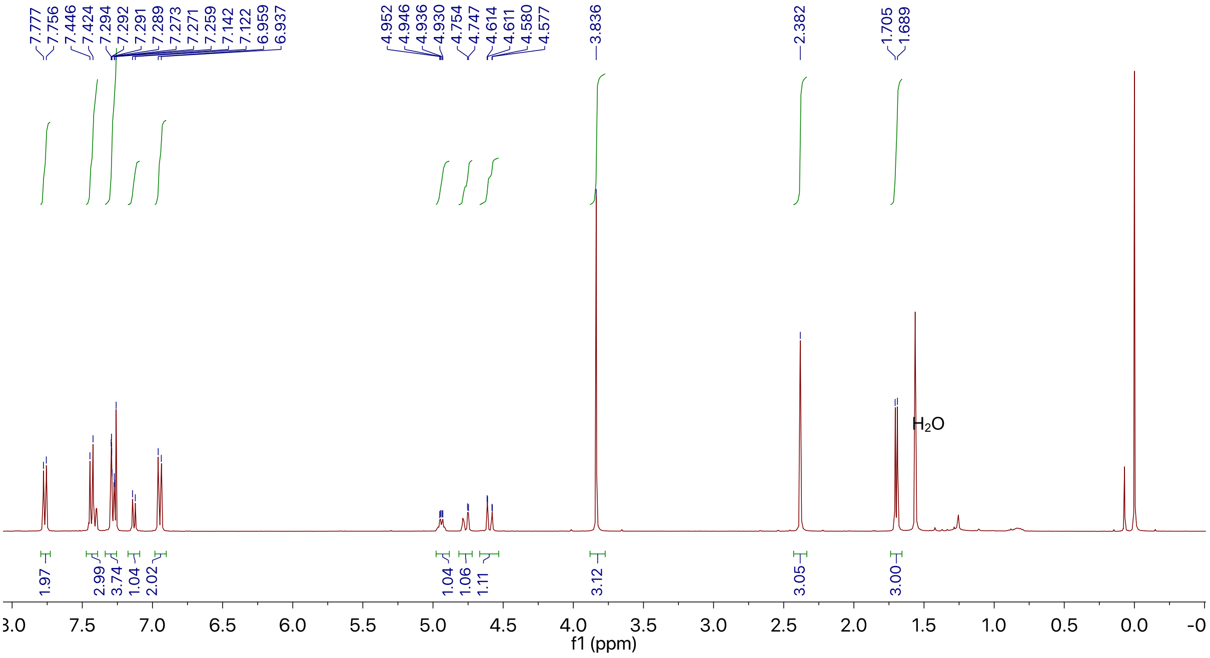

^{13}C NMR, (01 MHz, CDCl3

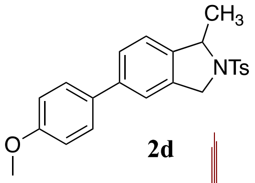

2d

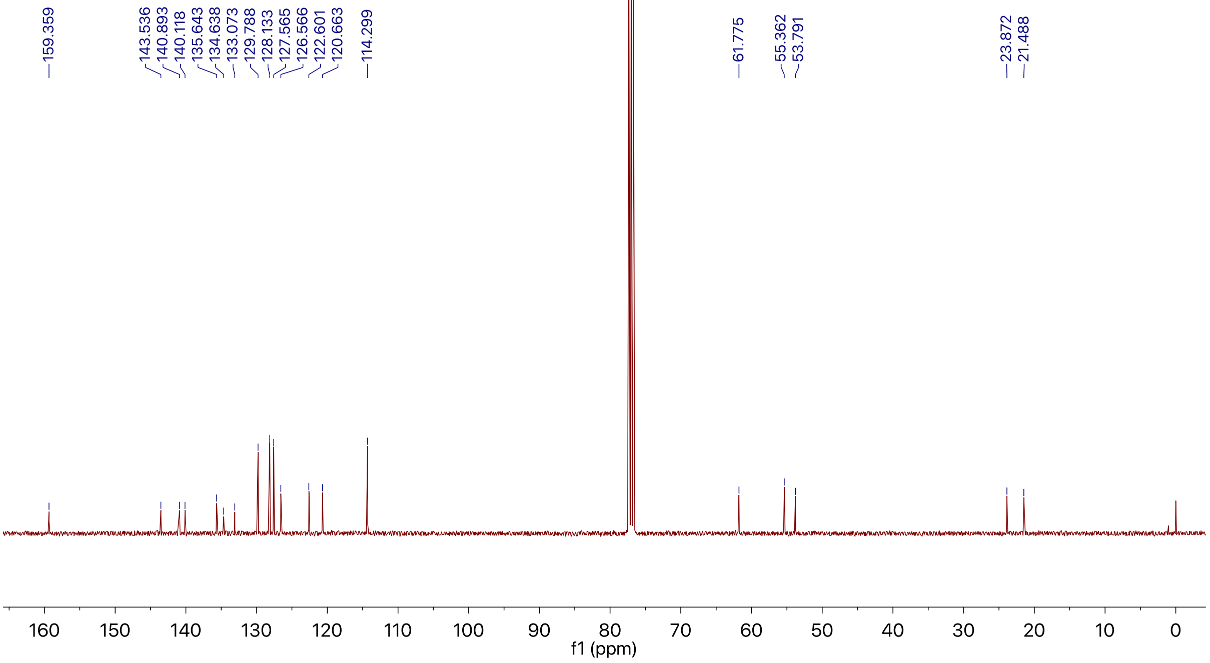

^{1}H NMR, 500 MHz, $CDCl_3$

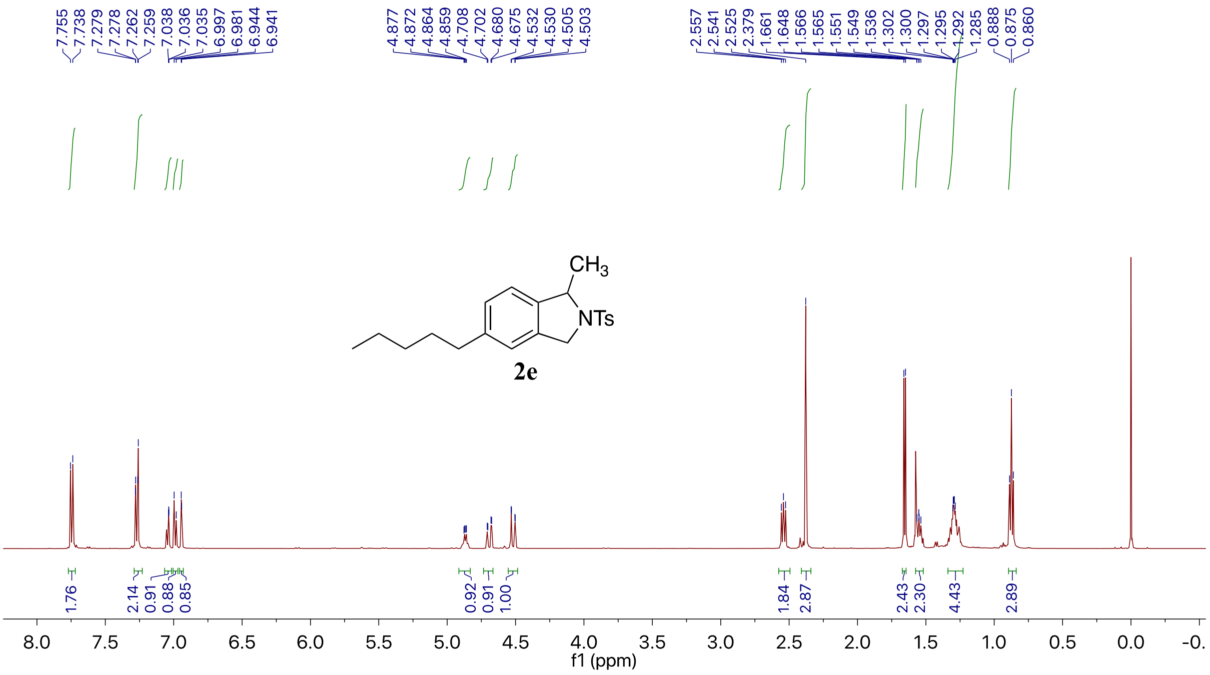

^{13}C NMR, 126 MHz, $CDCl_3$

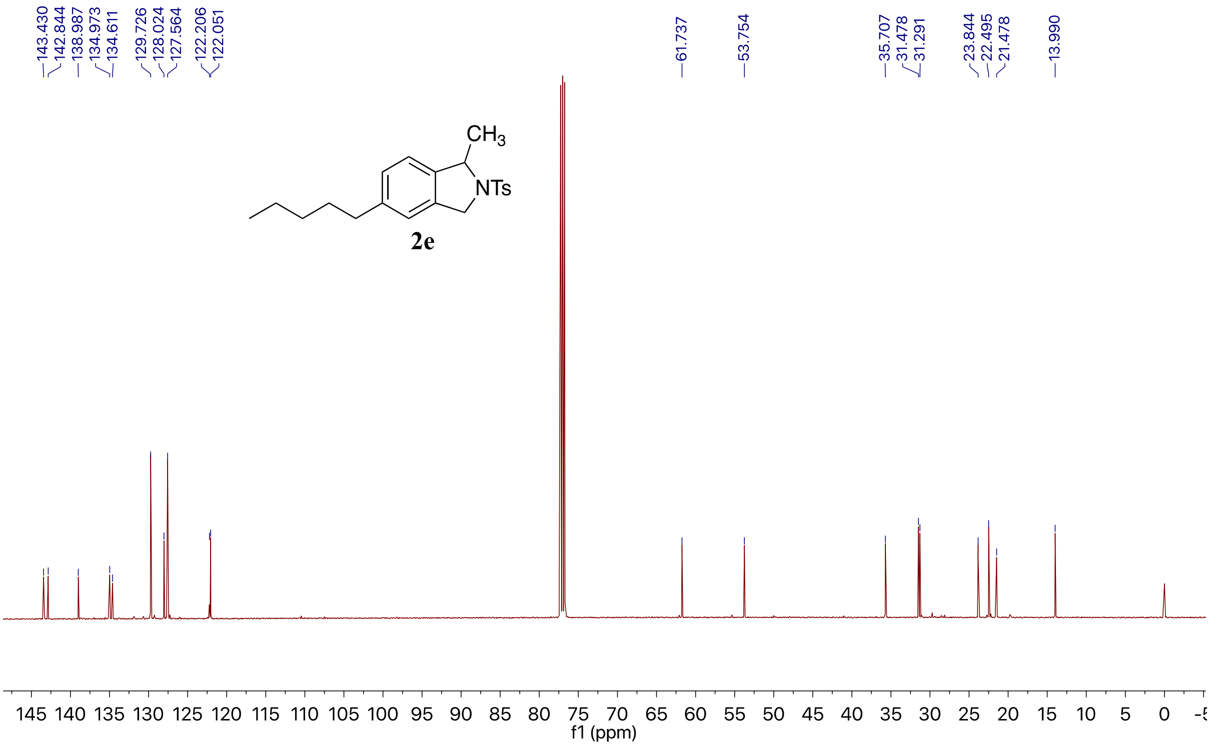

^{1}H NMR, 400 MHz, $CDCl_3$

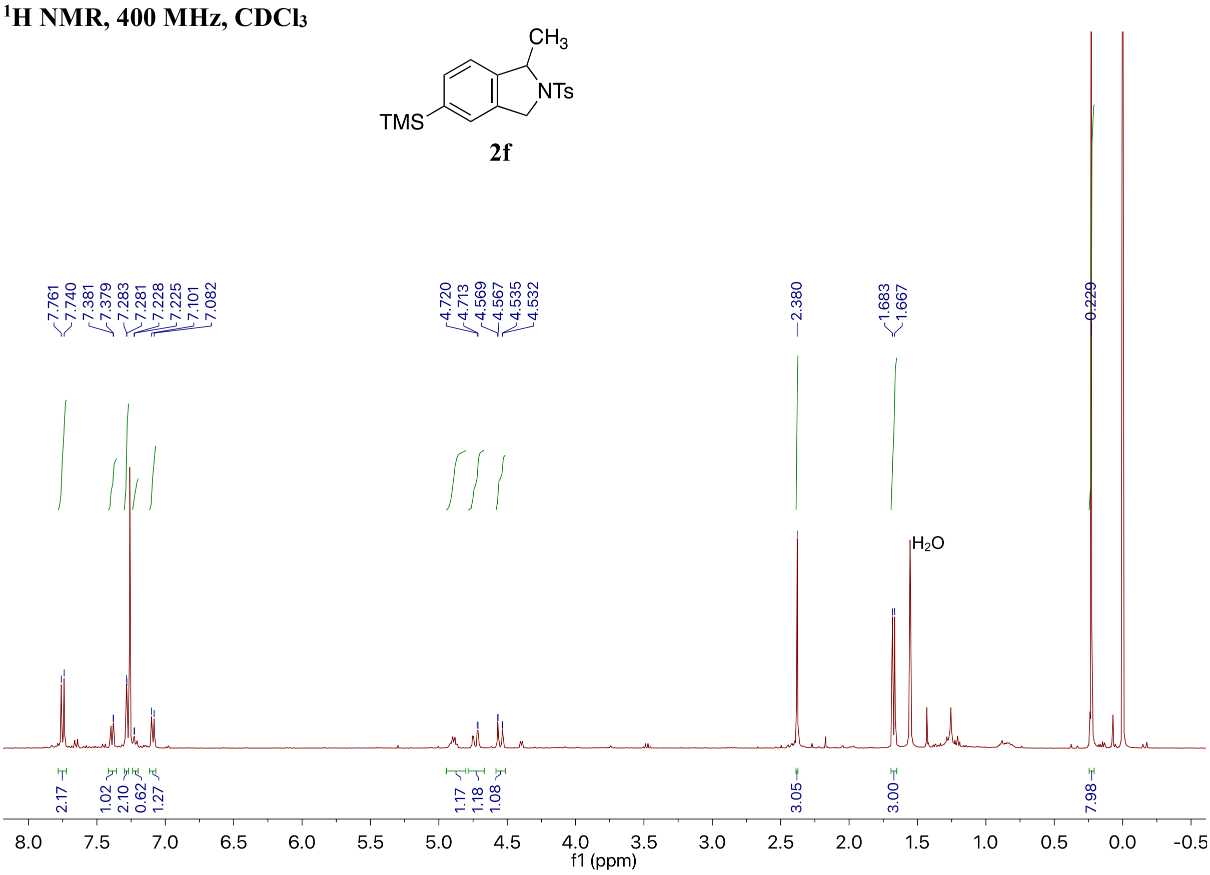

^{13}C NMR, 101 MHz, $CDCl_3$

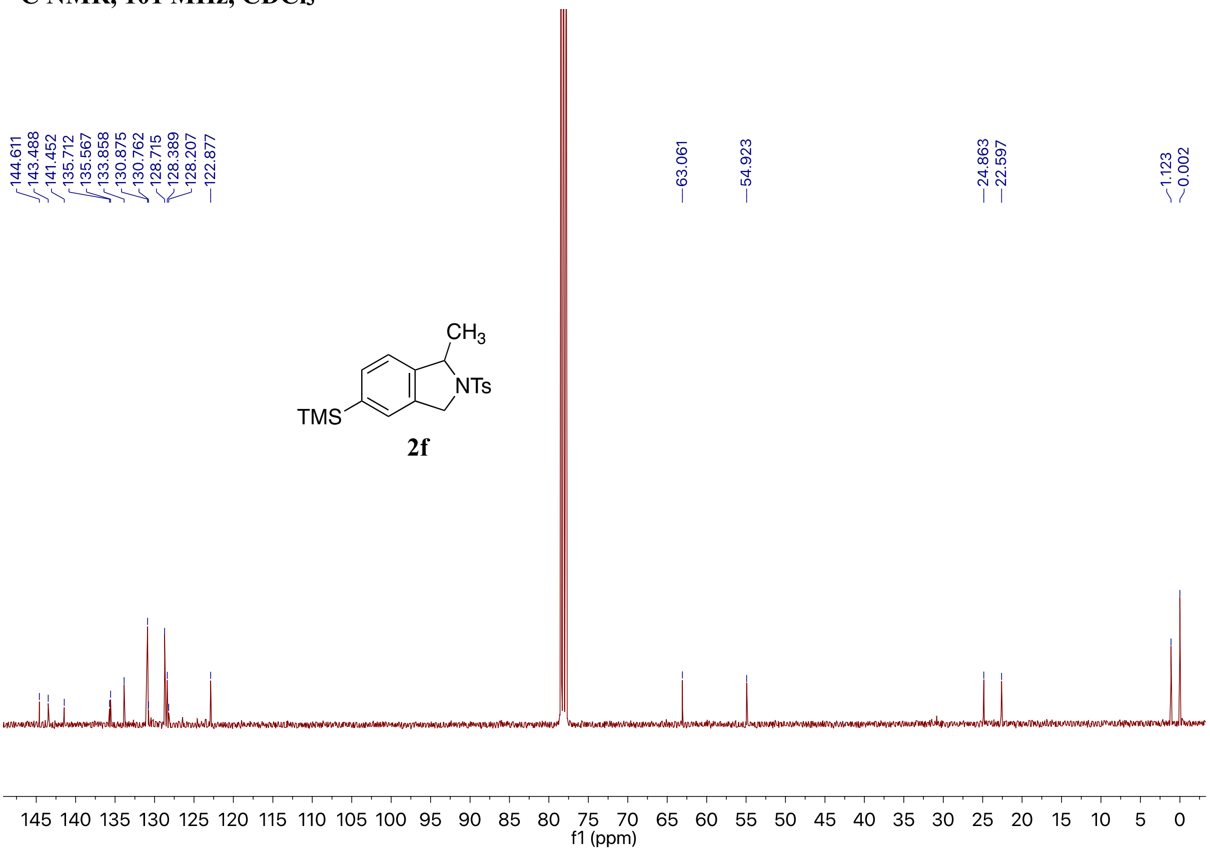

^{1}H NMR, 500 MHz, CDCl$_3$

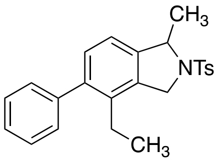

2g

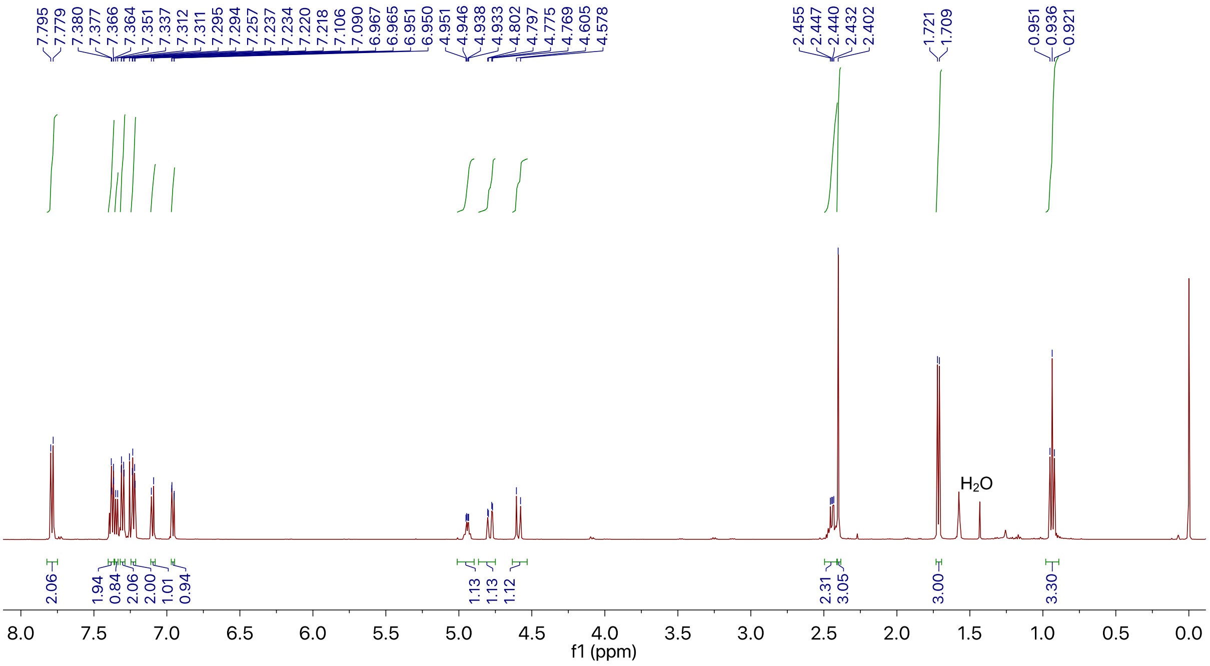

^{13}C NMR, 126 MHz, CDCl$_3$

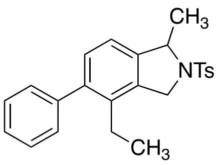

2g

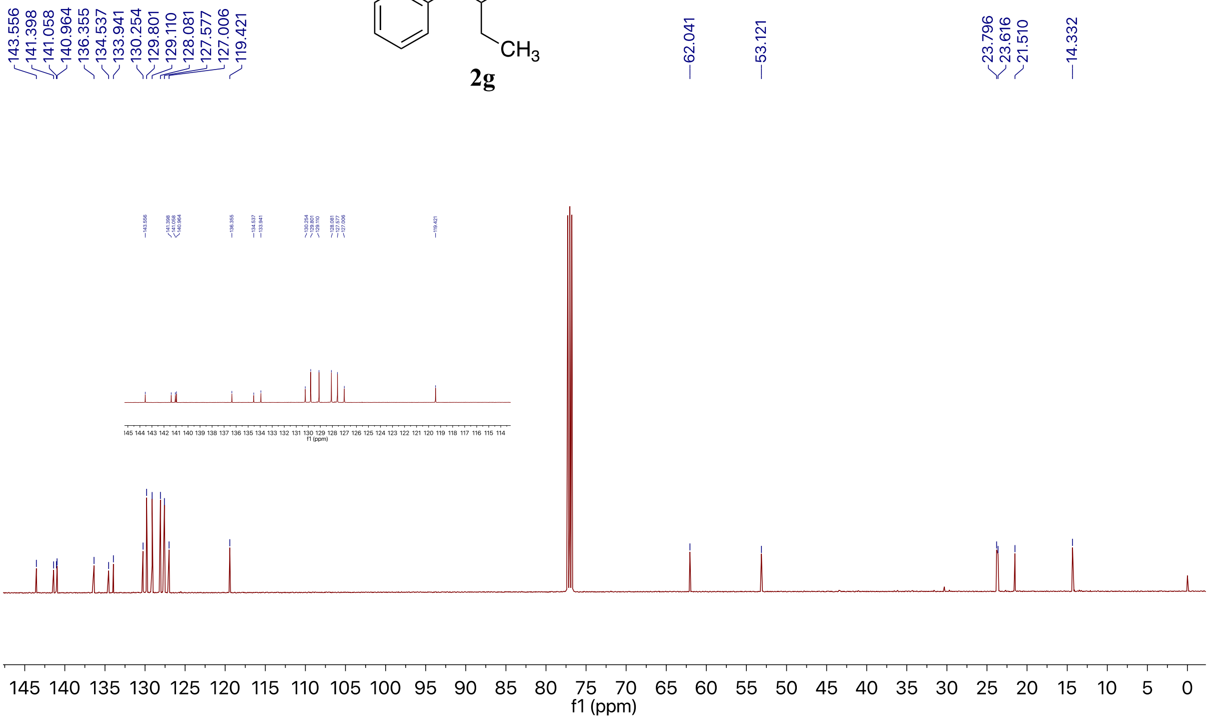

^{1}H NMR, 400 MHz, $CDCl_3$

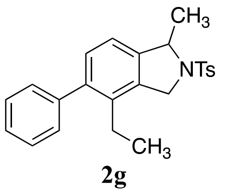

2g

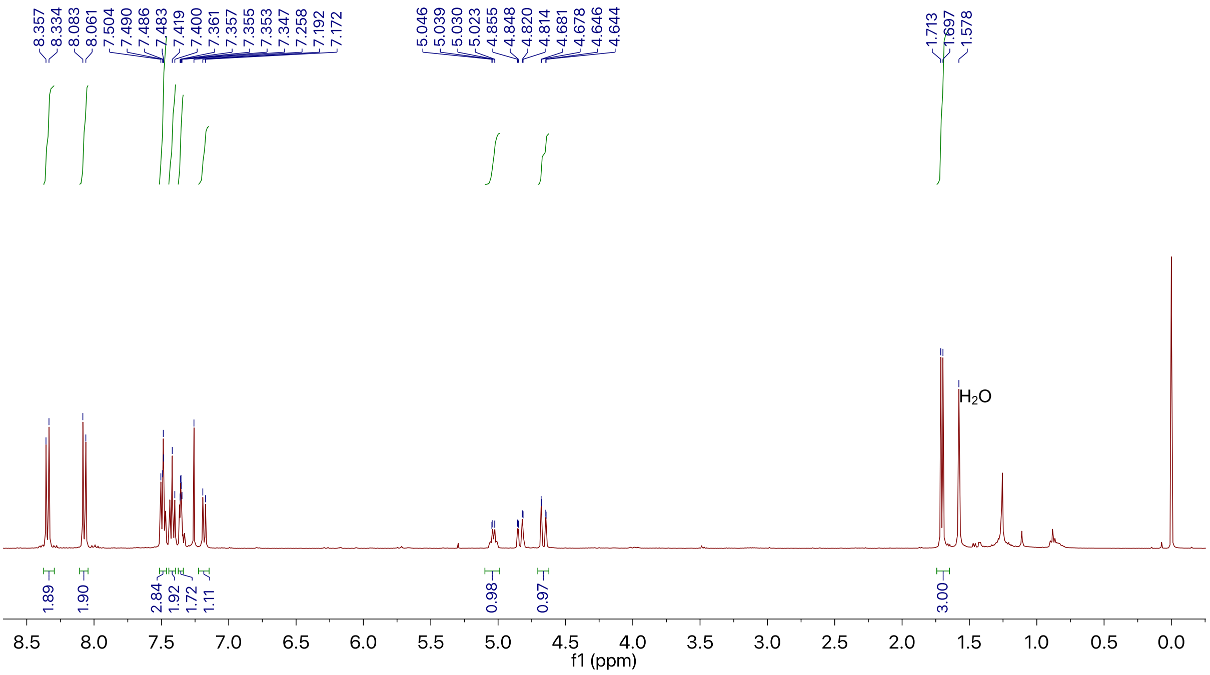

^{13}C NMR, 101 MHz, $CDCl_3$

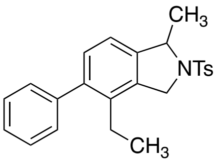

2g

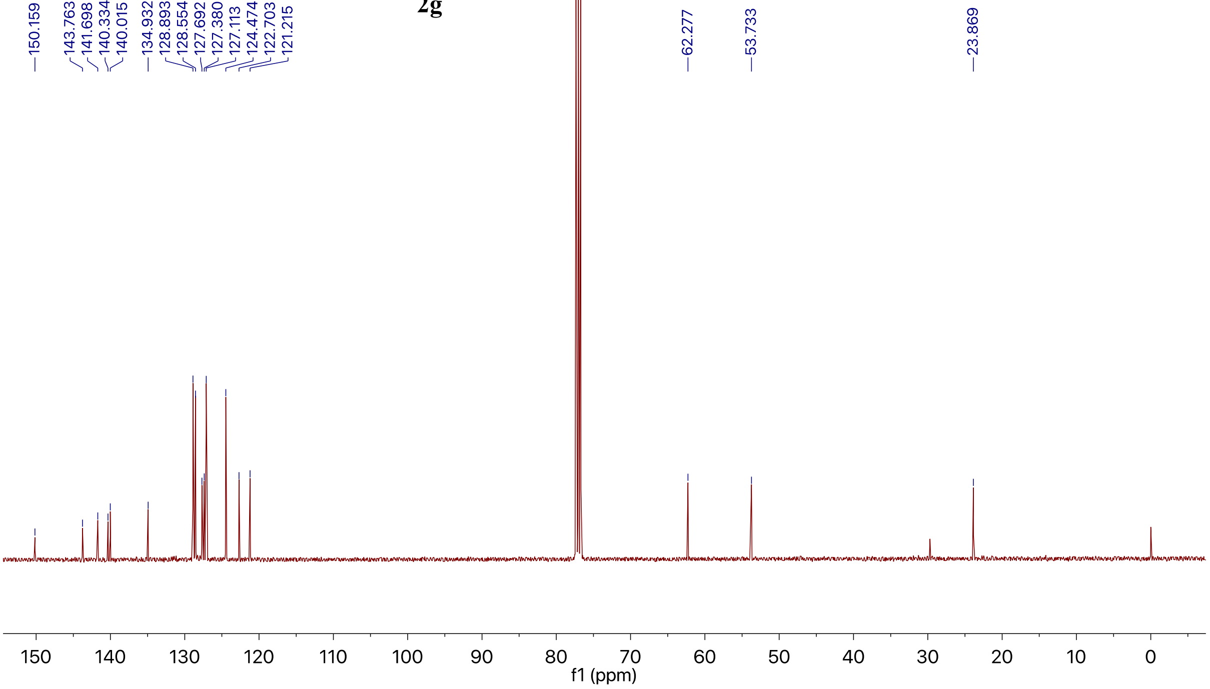

^{1}H NMR 500 MHz, $CDCl_3$

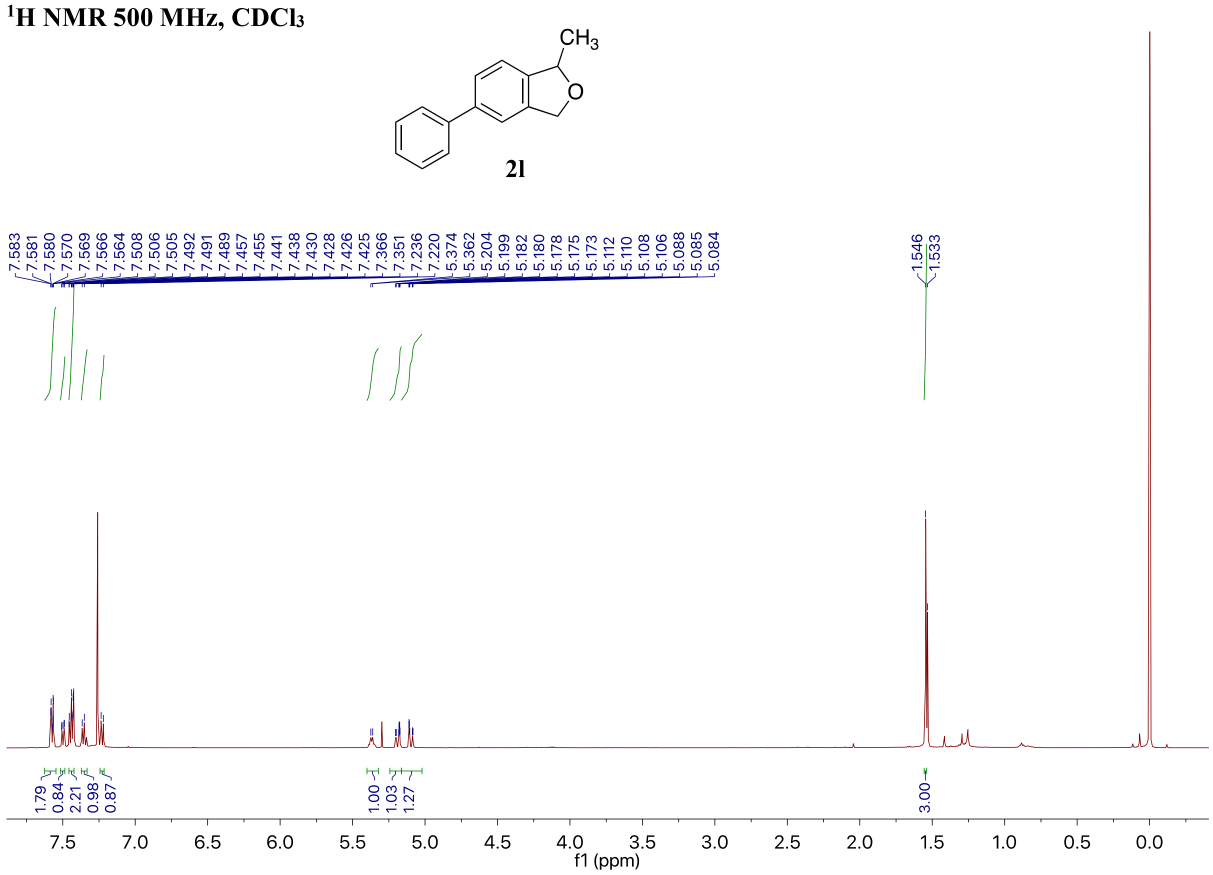

^{13}C NMR 126 MHz, $CDCl_3$

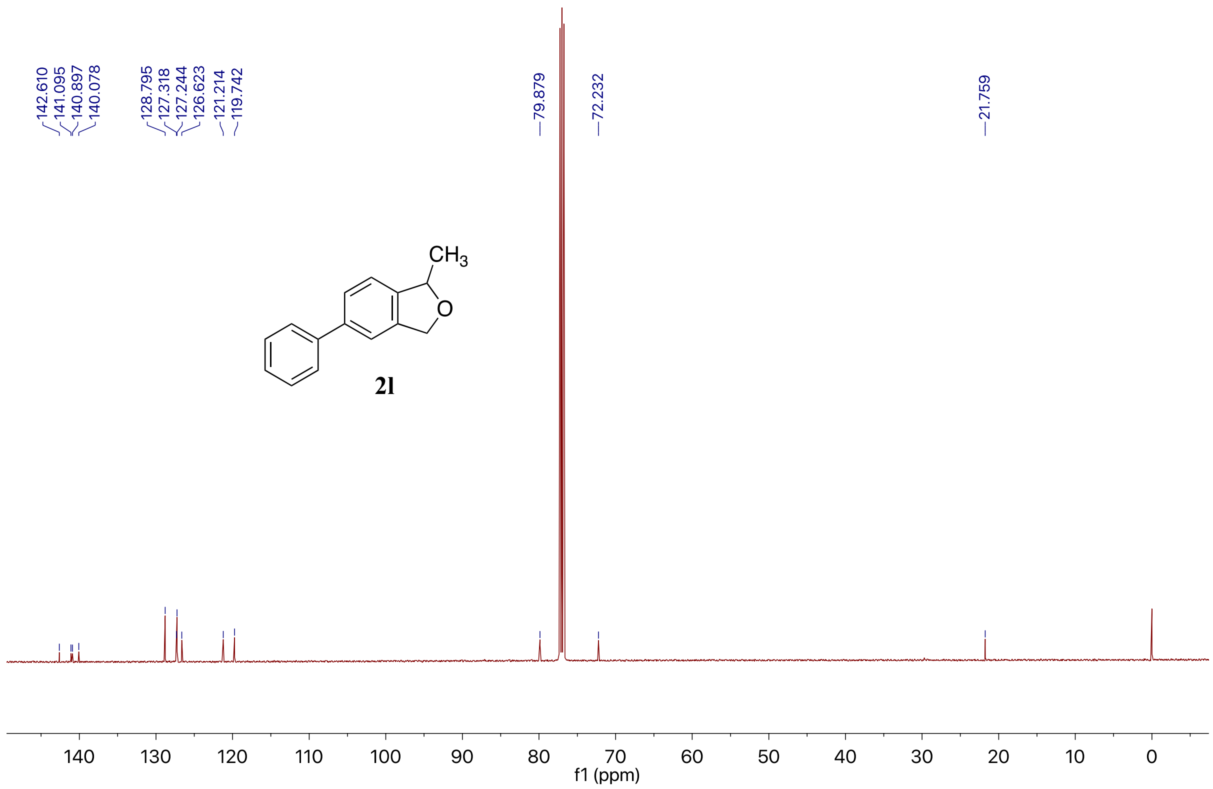

^{1}H NMR, 500 MHz, $CDCl_3$

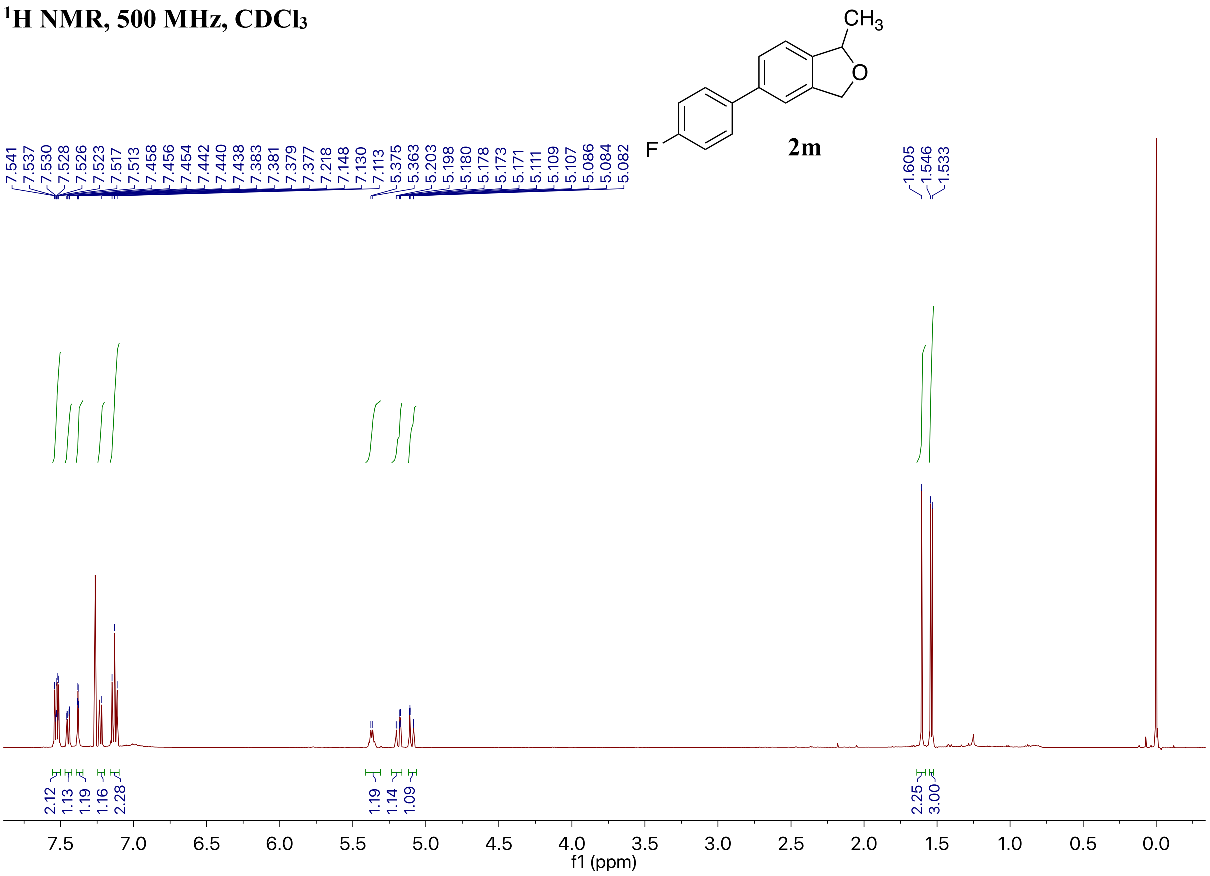

^{13}C NMR, 126 MHz, $CDCl_3$

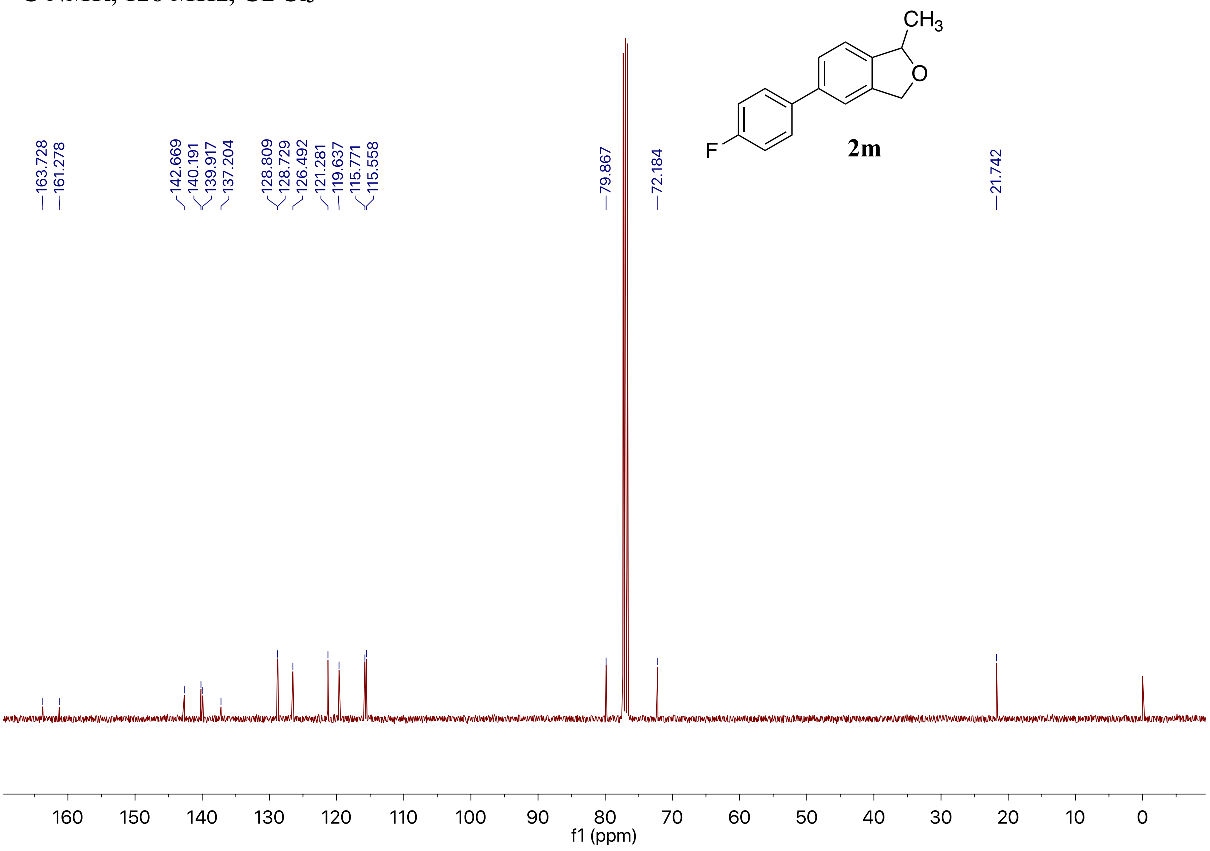

^{1}H NMR, 500 MHz, CDCl3

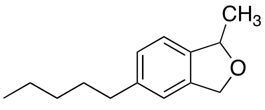

2o

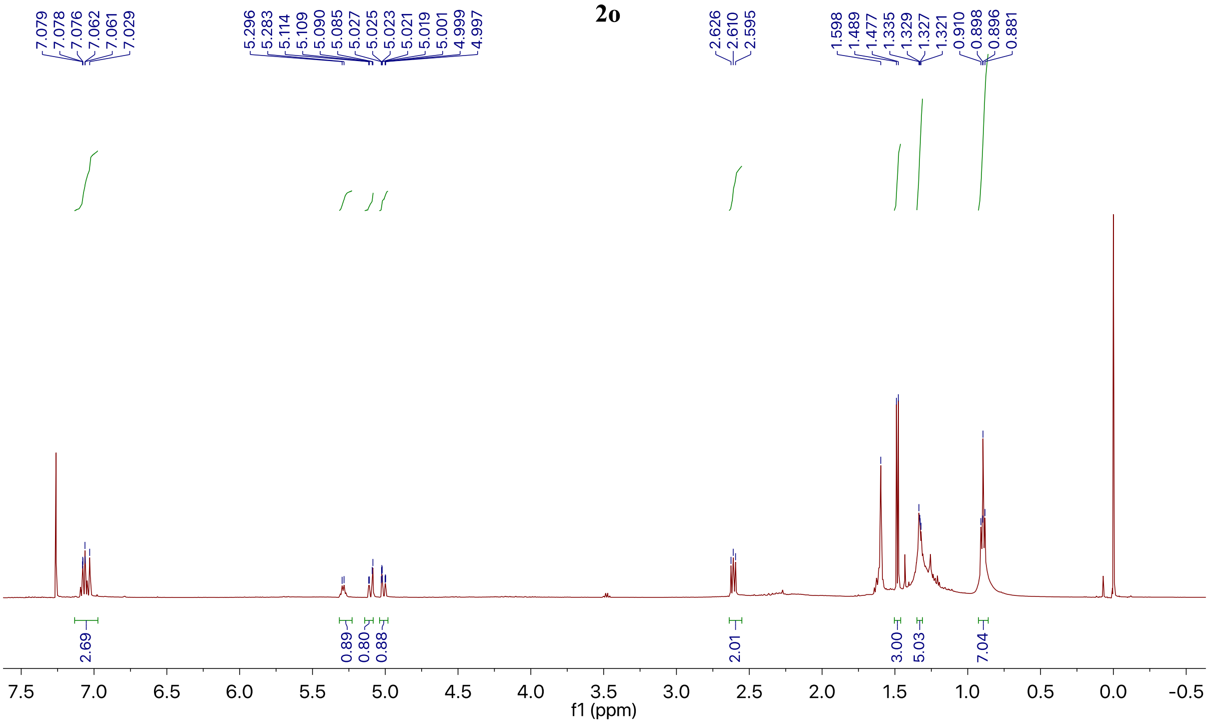

^{13}C NMR, 125 MHz, CDCl3

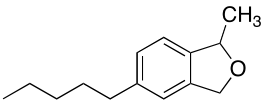

2o

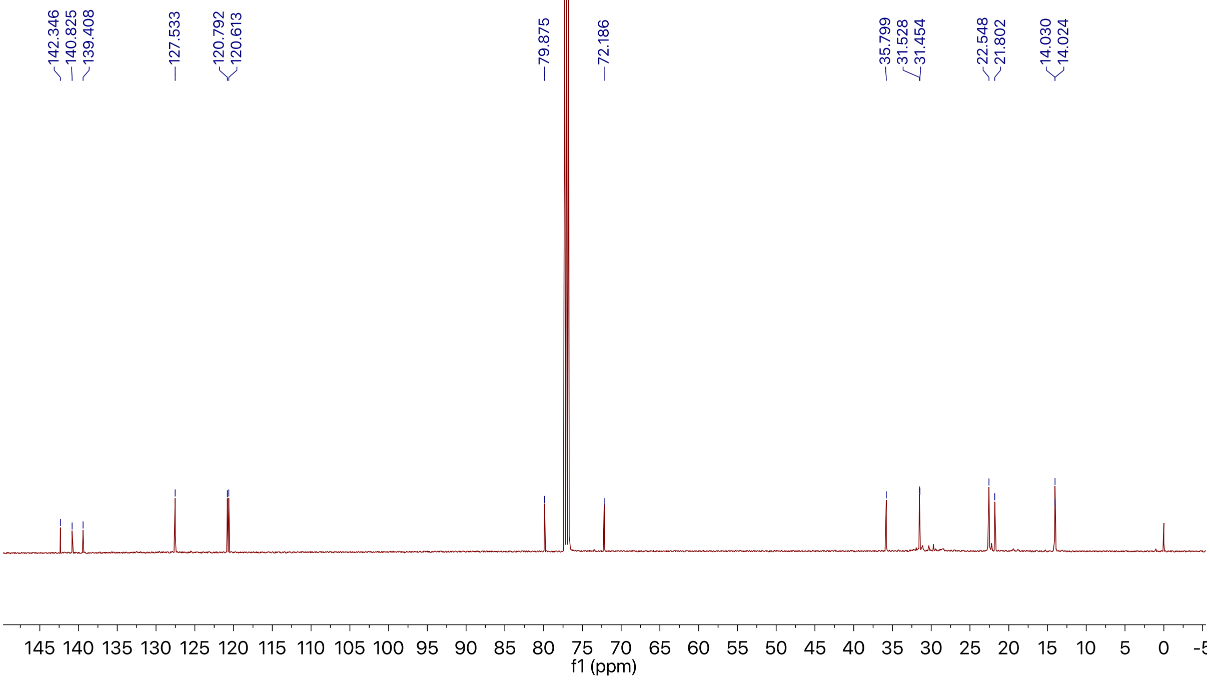

^{1}H NMR, 500 MHz, $CDCl_3$

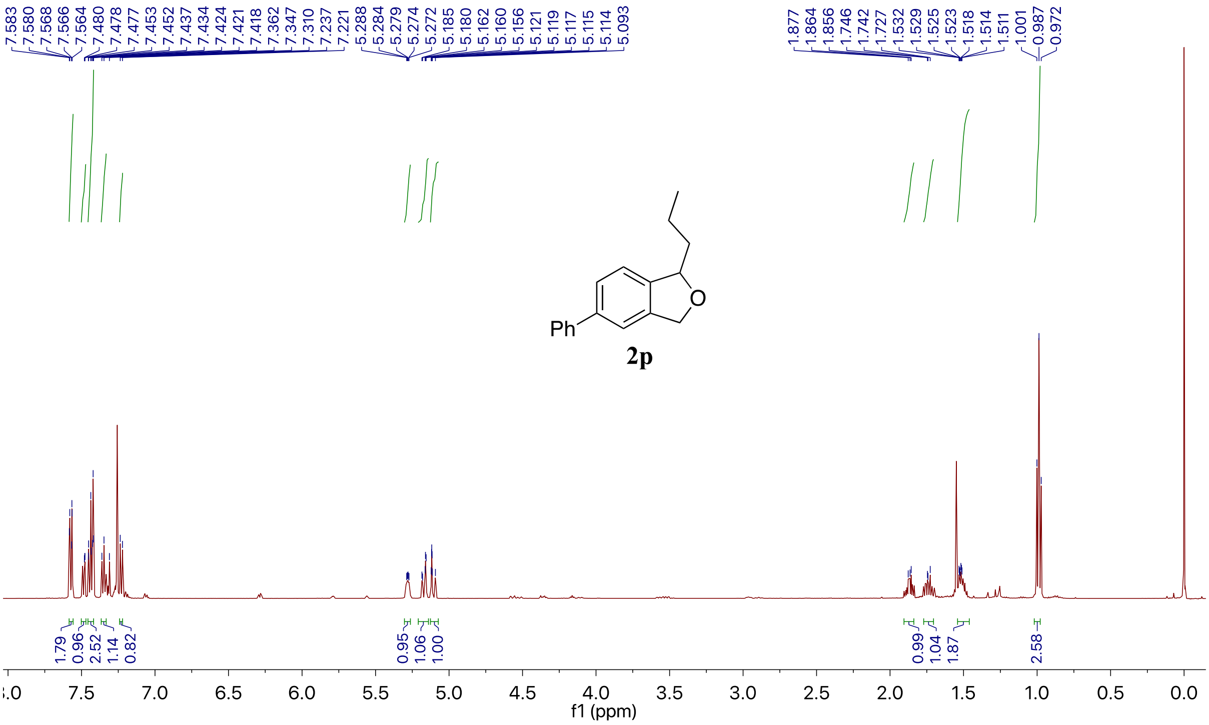

^{13}C NMR, 126 MHz, $CDCl_3$

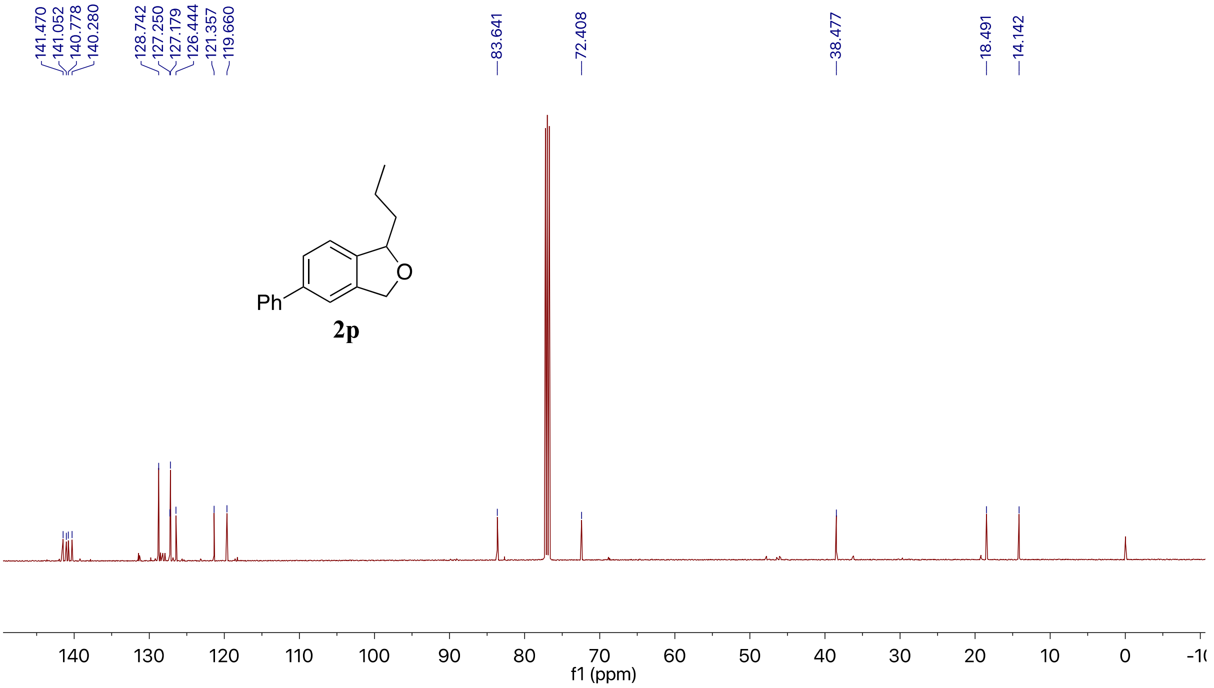

^{1}H NMR, 400 MHz, CDCl3

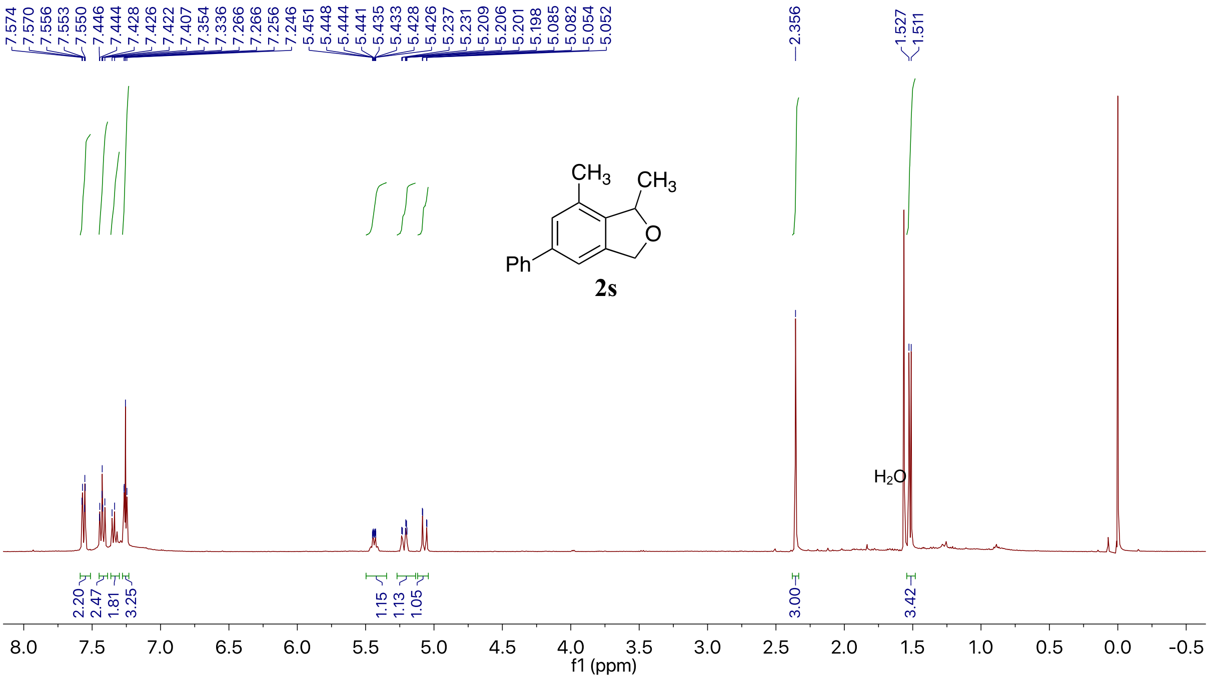

^{13}C NMR, 101 MHz, CDCl3

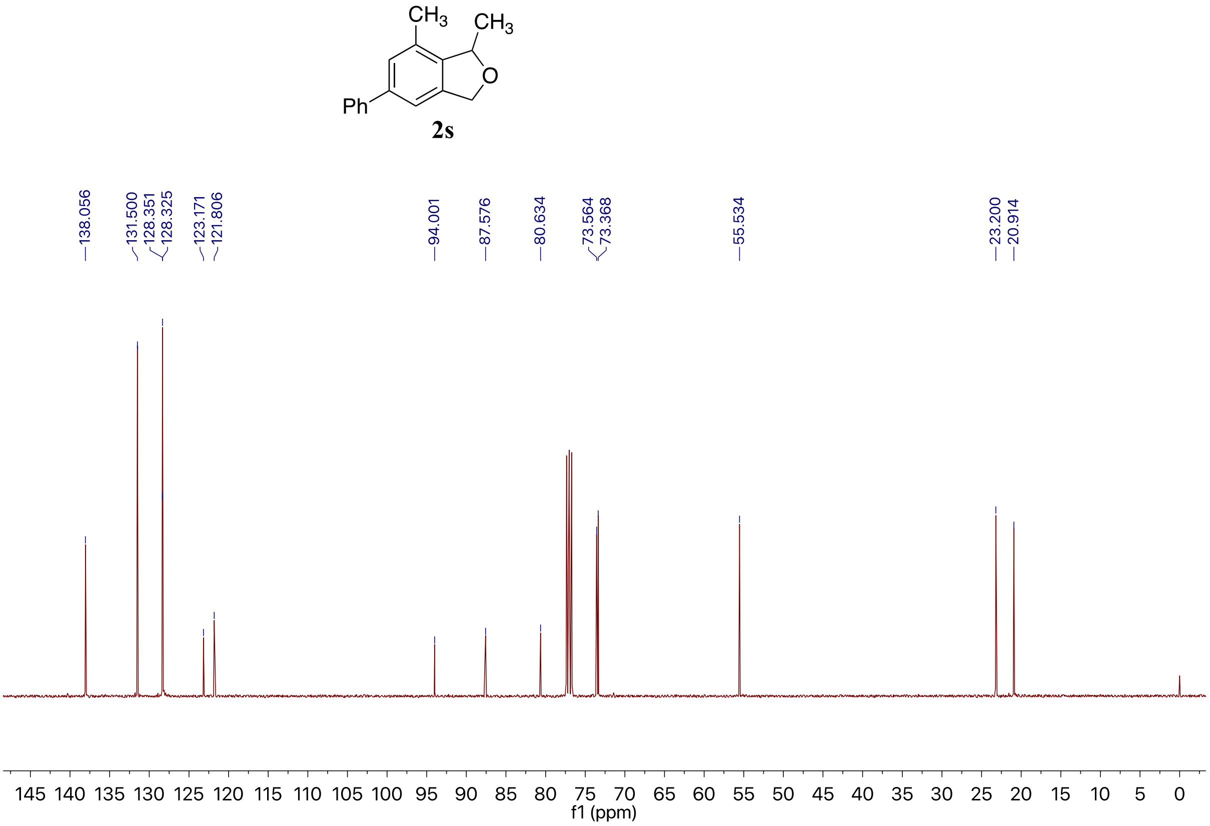

^{1}H NMR, 400 MHz, $CDCl_3$

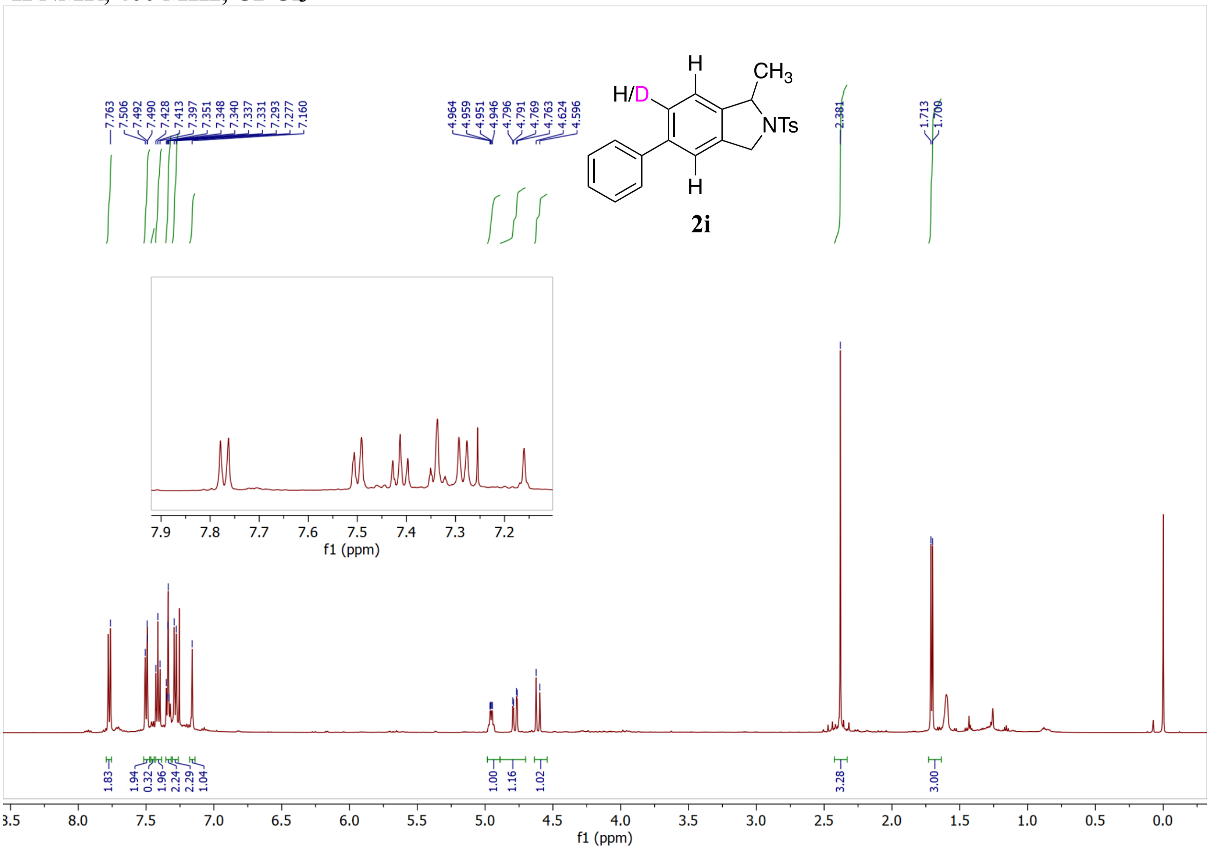

^{13}C NMR, 126 MHz, $CDCl_3$

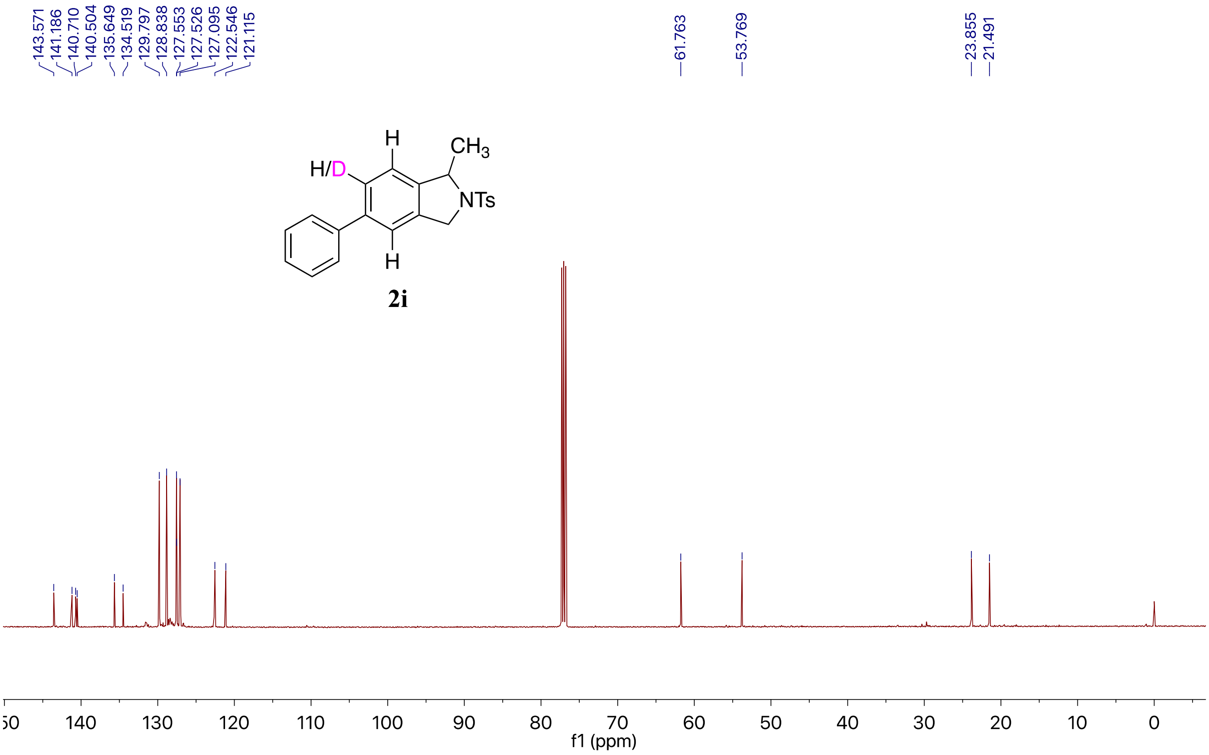

^{1}H NMR, 400 MHz, $CDCl_3$

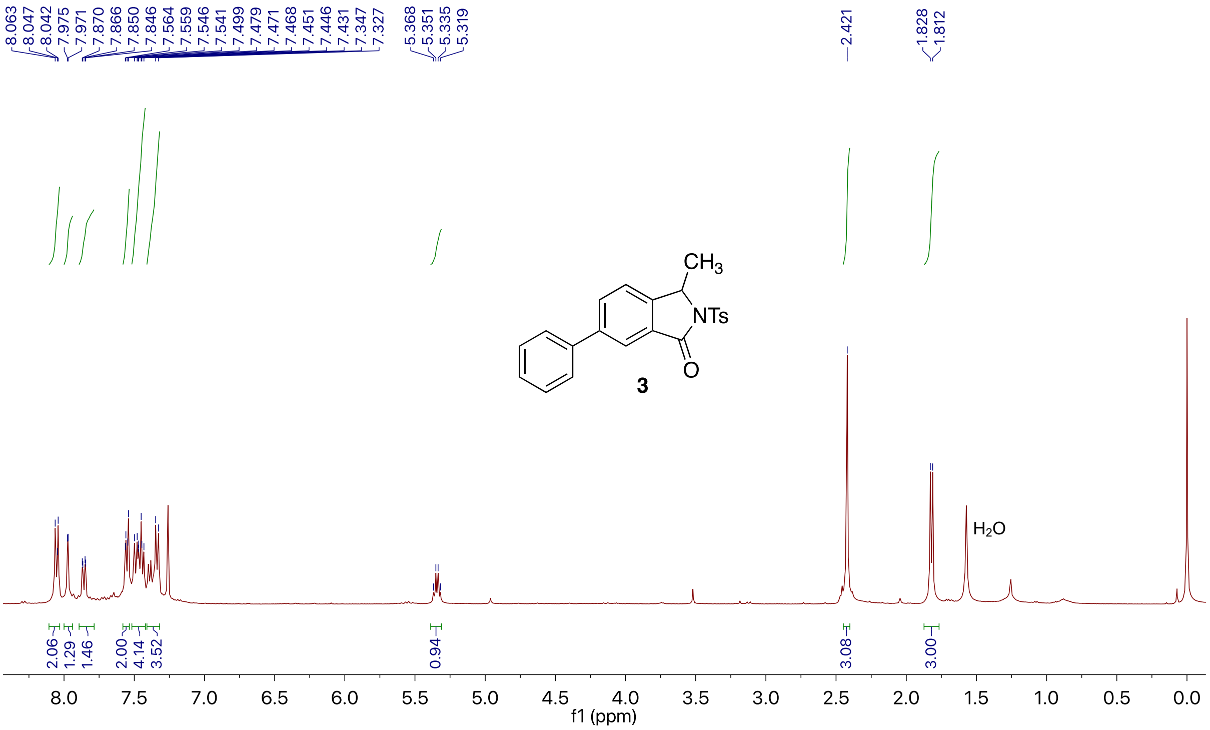

^{13}C NMR, 101 MHz, $CDCl_3$

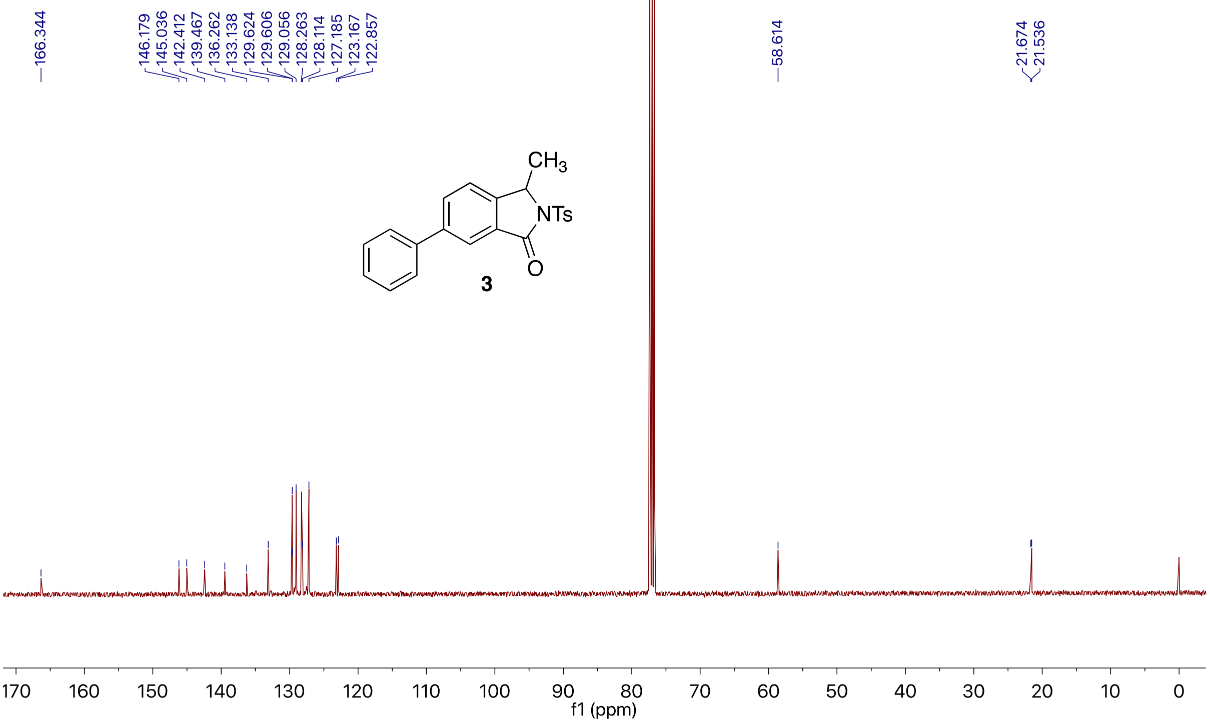

^{1}H NMR, 500 MHz, $CDCl_3$

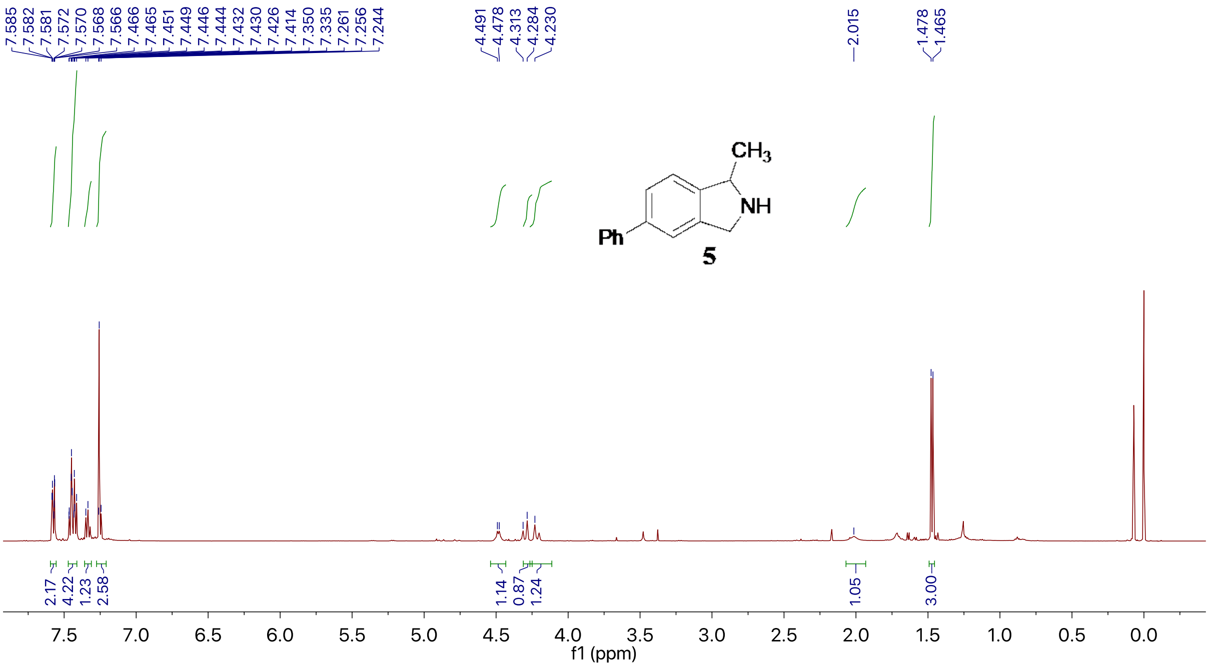

^{13}C NMR, 126 MHz, $CDCl_3$

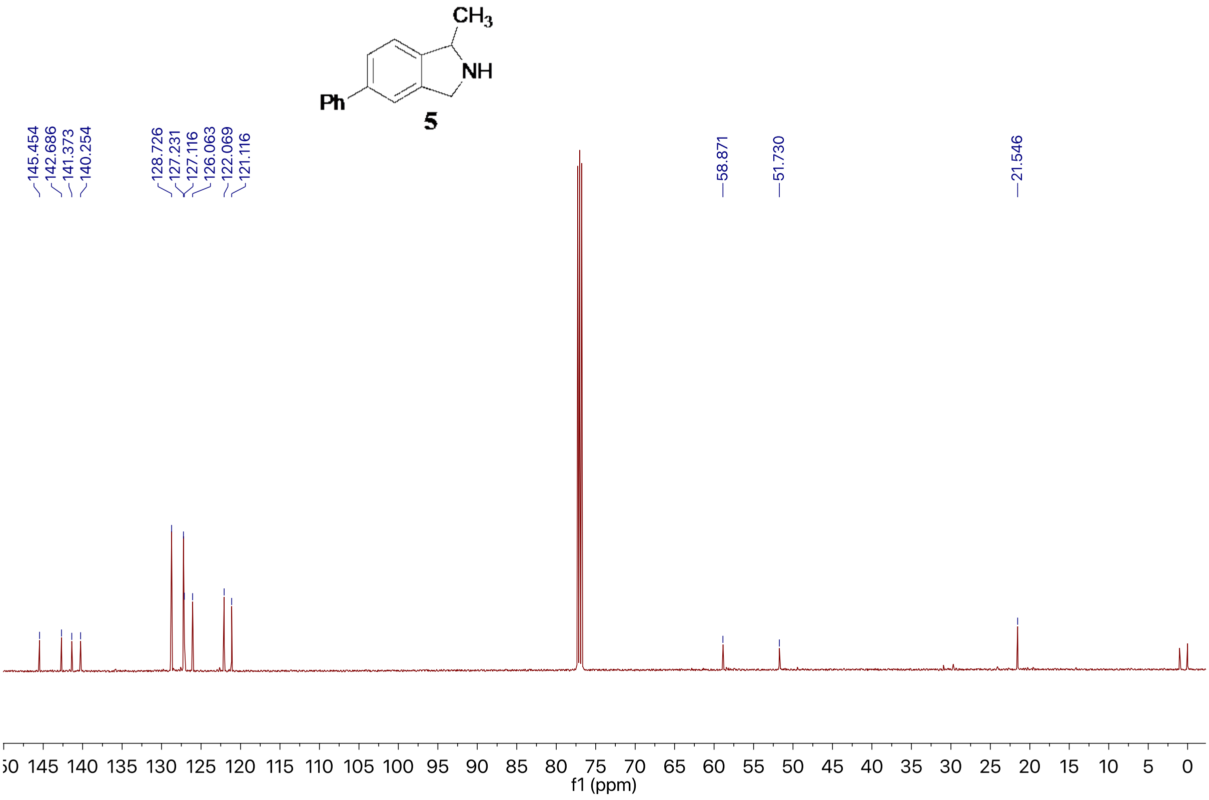

12. Computational details

Gaussian 09[1] was used to fully optimize all the structures reported in this paper at the M06 level of theory.[2] For all the calculations, solvent effects were considered using the SMD solvation model with dichloroethane as the solvent.[3] The effective core potential of Hay and Wadt with a double-ξ valence basis set (LANL2DZ) was chosen to describe rhodium.[4], [5] The [6-31G(d)] basis set was used for other atoms.[6] A polarization function was also added for Rh (ξ_d = 1.350).[7] This basis set combination will be referred to as BS1. Frequency calculations were carried out at the same level of theory as those for the structural optimization. Transition structures were located using the Berny algorithm. Intrinsic reaction coordinate (IRC) calculations were used to confirm the connectivity between transition structures and minima.[8], [9] To further refine the energies obtained from the SMD/M06/LanL2DZ,6-31G(d) calculations, we carried out single-point energy calculations using the M06 functional method with SMD solvation model in dichloroethane along with a larger basis set (BS2) for all the optimized structures. BS2 utilizes the def2-TZVP basis set[10] on all atoms. Tight convergence criterion and ultrafine integral grid were exploited to increase the accuracy of the single point calculations. In this work, the free energy for each species in solution was calculated using the following formula:

$$G = E(BS2) + G(BS1) - E(BS1) + \Delta G^{1atm\rightarrow 1M} \quad (1)$$

where $\Delta G^{1atm\rightarrow 1M}$ = 1.89 kcal/mol is the free-energy change for compression of 1 mol of an ideal gas from 1 atm to the 1 M solution phase standard state.[11]

[1] Frisch, M. J.; Trucks, G. W.; Schlegel, H. B.; Scuseria, G. E.; Robb, M. A.; Cheeseman, J. R.; Scalmani, G.; Barone, V.; Mennucci, B.; Petersson, G. A.; Nakatsuji, H.; Caricato, M.; Li, X.; Hratchian, H. P.; Izmaylov, A. F.; Bloino, J.; Zheng, G.; Sonnenberg, J. L.; Hada, M.; Ehara, M.; Toyota, K.; Fukuda, R.; Hasegawa, J.; Ishida, M.; Nakajima, T.; Honda,

Y.; Kitao, O.; Nakai, H.; Vreven, T.; Montgomery, J. A.; Peralta, Jr., J. E.; Ogliaro, F.; Bearpark, M.; Heyd, J. J.; Brothers, E.; Kudin, K. N.; Staroverov, V. N.; Kobayashi, R.; Normand, J.; Raghavachari, K.; Rendell, A.; Burant, J. C.; Iyengar, S. S.; Tomasi, J.; Cossi, M.; Rega, N.; Millam, J. M.; Klene, M.; Knox, J. E.; Cross, J. B.; Bakken, V.; Adamo, C.; Jaramillo, J.; Gomperts, R.; Stratmann, R. E.; Yazyev, O.; Austin, A. J.; Cammi, R.; Pomelli, C.; Ochterski, J. W.; Martin, R. L.; Morokuma, K.; Zakrzewski, V. G.; Voth, G. A.; Salvador, P.; Dannenberg, J. J.; Dapprich, S.; Daniels, A. D.; Farkas, O.; Foresman, J. B.; Ortiz, J. V.; Cioslowski, J.; Fox, D. J. Gaussian 09, revision D.01; Gaussian, Inc.: Wallingford, CT, 2009.

[2] Zhao, Y.; Truhlar, D. G. The M06 suite of density functionals for main group thermochemistry thermochemical kinetics noncovalent interactions excited states and transition elements: two new functional and systematic testing of four M06-class functionals and 12 other functionals., *Theoretical Chemistry Accounts* **2008**, *120*, 215-241.

[3] Marenich, A. V.; Cramer, C. J.; Truhlar, D. G., Universal Solvation Model Based on Solute Electron Density and on a Continuum Model of the Solvent Defined by the Bulk Dielectric Constant and Atomic Surface Tensions. *J. Phys. Chem. B* **2009,** *113*, 6378-6396.

[4] Hay, P. J.; Wadt, W. R., Ab Initio Effective Core Potentials for Molecular Calculations. Potentials for the Transition Metal Atoms Sc to Hg. *J. Chem. Phys.* **1985,** *82*, 270-283.

[5] adt, W. R.; Hay, P. J., Ab Initio Effective Core Potentials for Molecular Calculations. Potentials for Main Group Elements Na to Bi. *J. Chem. Phys.* **1985,** *82*, 284-298.

[6] Hariharan, P. C.; Pople, J. A., The Influence of Polarization Functions on Molecular Orbital Hydrogenation Energies. *Theor. Chem. Acta.* **1973,** *28*, 213-222.

[7] Höllwarth, A.; Böhme, M.; Dapprich, S.; Ehlers, A.; Gobbi, A.; Jonas, V.; Köhler, K.; Stegmann, R.; Veldkamp, A.; Frenking, G., A Set of d-Polarization Functions for Pseudo-Potential Basis Sets of the Main Group Elements Al-Bi and f-Type Polarization Functions for Zn, Cd, Hg. *Chem. Phys. Lett.* **1993,** *208*, 237-240.

[8] Fukui, K., The Path of Chemical Reactions-the IRC approach. *Acc. Chem. Res.* **1981,** *14*, 363-368.

[9] Fukui, K., Formulation of the Reaction Coordinate. *J. Phys. Chem.* **1970,** *74*, 4161-4163.

[10] Weigend, F.; Furche, F.; Ahlrichs, R., Gaussian Basis Sets of Quadruple Zeta Valence Quality for Atoms H–Kr. *J. Phys. Chem.* **2003,** *119*, 12753-12762.

[11] Ochterski, J., Thermochemistry, W., Gaussian, Gaussian, Inc., Wallingford, CT, **2000**, pp. 1–19.

[12] Bryantsev, V. S.; Diallo, M. S.; Goddard Iii, W. A., Calculation of Solvation Free Energies of Charged Solutes Using Mixed Cluster/Continuum Models. *J. Phys. Chem. B* **2008,** *112*, 9709-9719.

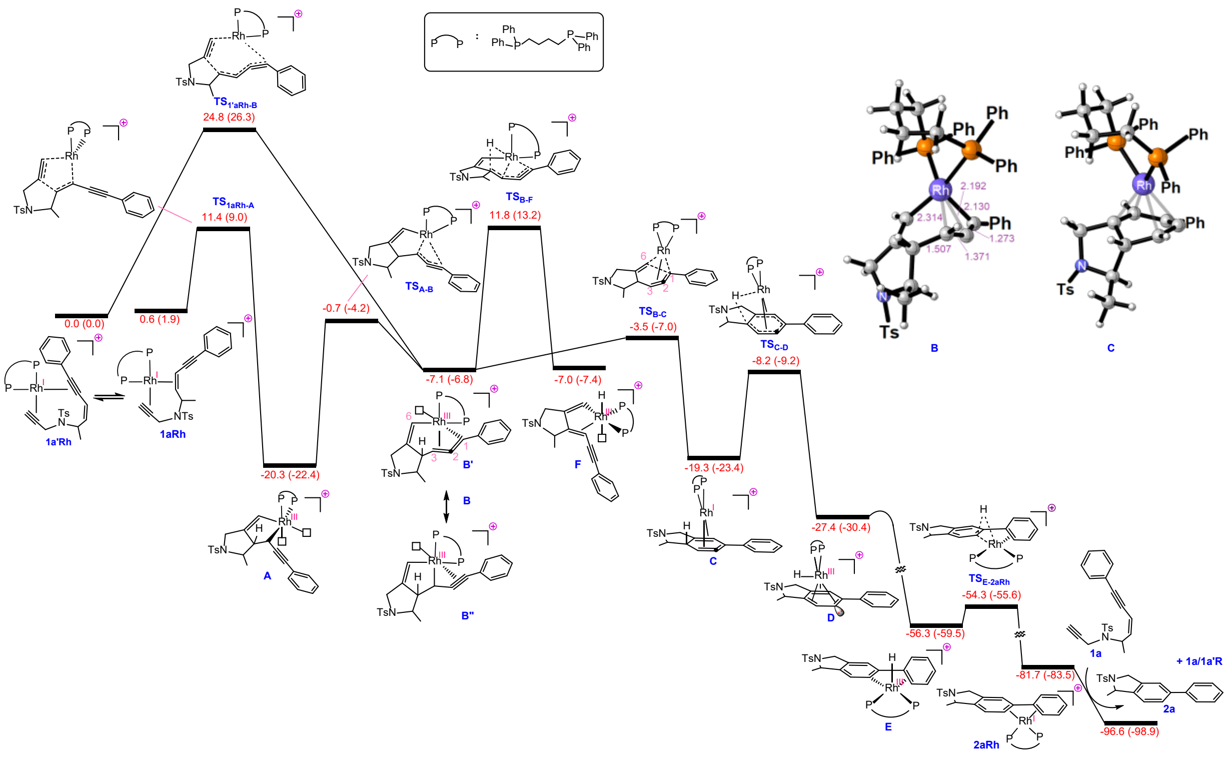

‘

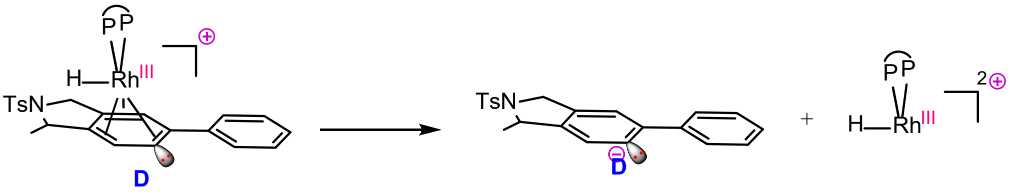

30.9 (46.1)

Table S1 Cartesian coordinates and total energies for all of the calculated structures.

Substrate **1a**

E (M06-SMD/BS1) = -1453.37740837 au

H (M06-SMD/BS1) = -1452.978904 au

G (M06-SMD/BS1) = -1453.056319 au

E (M06-SMD/BS2//M06-SMD/BS1) = -1453.86019660 au

C	5.42170300	0.68880100	-0.33444300
C	4.09922400	1.12760800	-0.28585700
C	3.06761400	0.21399400	-0.11974500

C	3.34935900	-1.15825600	-0.00747300
C	4.68284100	-1.59180200	-0.05398800
C	5.70901900	-0.66960000	-0.21570200
H	6.22961200	1.40691000	-0.46457600
H	3.87083900	2.18895400	-0.38028800
H	2.02860900	0.54452000	-0.07410600
H	4.90138500	-2.65491300	0.03365700
H	6.74130900	-1.01355800	-0.25196700
C	2.27239800	-2.07691400	0.14140500
C	1.29305900	-2.78964000	0.25633200
C	0.13262300	-3.58962200	0.40743100
H	0.26813700	-4.59788700	0.80152900
C	-1.10498100	-3.17408400	0.08936400
H	-1.94814800	-3.85400000	0.23080600
C	-1.42383500	-1.86465700	-0.57065100
H	-0.52155700	-1.22737400	-0.55596400
N	-2.51122100	-1.20065900	0.21215800
C	-2.27356700	-1.19882100	1.67223500
H	-2.33959700	-2.23982100	2.01374300
H	-3.10647700	-0.67022900	2.14889500
C	-0.99081500	-0.62785200	2.08583800
C	0.07974800	-0.16867700	2.40969500
H	1.02421600	0.24284000	2.70858500
S	-3.15208200	0.26396200	-0.35050200
O	-3.53647700	0.07373500	-1.74207300
O	-4.16696800	0.65124700	0.62406800
C	-1.84422500	1.46026600	-0.28475400
C	-0.86366900	1.46055900	-1.27508700
C	-1.77657200	2.33678100	0.79649700
C	0.20621300	2.34061300	-1.16309200
H	-0.94030400	0.79422600	-2.13303400
C	-0.70568100	3.21403500	0.88640200
H	-2.55587100	2.32667700	1.55609000
C	0.30735100	3.21790100	-0.07871900
H	0.97987600	2.34841800	-1.93165100
H	-0.64617100	3.90767400	1.72520800
C	1.48372900	4.13248100	0.06387100
H	2.18497900	3.74808900	0.81937400
H	2.03347000	4.23604100	-0.87952300
H	1.17688300	5.13261900	0.39623700
C	-1.80160300	-2.18241900	-2.01398200
H	-2.73837900	-2.75384200	-2.04976800
H	-1.92122100	-1.29079400	-2.63391900
H	-1.00177700	-2.79733200	-2.44582100

product
E (M06-SMD/BS1) = -1453.54852441 au

H (M06-SMD/BS1) = -1453.145281 au
G (M06-SMD/BS1) = -1453.223660 au
E (M06-SMD/BS2//M06-SMD/BS1) = -1454.01787534 au

C	-7.00997800	1.27566100	-0.60563000
C	-6.31620900	0.48971500	-1.52226500
C	-5.04378100	0.01860400	-1.21876000
C	-4.43719000	0.32374900	0.00675000
C	-5.14663600	1.11511700	0.91967800
C	-6.41955100	1.58593700	0.61671600
H	-8.00667900	1.64447300	-0.84269800
H	-6.77174600	0.23372000	-2.47785900
H	-4.52044200	-0.61628000	-1.93355600
H	-4.68478200	1.38534100	1.86917500
H	-6.94984600	2.20621900	1.33801700
C	-3.08239600	-0.17709700	0.32486500
C	-2.74630600	-0.55327600	1.63523900
C	-1.48039900	-1.03595300	1.94925200
H	-1.24550700	-1.33420800	2.97093200
C	-0.53083300	-1.14087900	0.94027800
C	0.87833800	-1.65014700	1.03987300
H	1.49697600	-0.94883100	1.62896200
N	1.30274400	-1.63197000	-0.39022600
C	0.32805800	-0.94404200	-1.26425700
H	0.11507400	-1.55347100	-2.15198900
H	0.70587400	0.03134300	-1.61549600
C	-0.85000600	-0.77005000	-0.36056900
C	-2.11172400	-0.29077700	-0.67929100
H	-2.34146800	0.01547000	-1.70000400
S	2.90903900	-1.41968200	-0.74711000
O	3.66042600	-2.28372400	0.15702000
O	3.02498700	-1.57603000	-2.19304200
C	0.97120800	-3.04039400	1.64059900
H	0.36135300	-3.74379600	1.05847900
H	2.00753200	-3.39476900	1.64961800
H	0.59897400	-3.02966700	2.67373500
C	3.31510800	0.26197100	-0.34577800
C	3.17957300	1.25512800	-1.31530700
C	3.72775300	0.57764500	0.94909600
C	3.45268200	2.57389900	-0.97548300
H	2.88450100	0.99377000	-2.32982400
C	3.99591600	1.90162500	1.26935000
H	3.85626700	-0.20882900	1.69057800
C	3.86253700	2.91693000	0.31650800
H	3.35251800	3.35524900	-1.72885000
H	4.32205100	2.15571400	2.27791900
C	4.18560500	4.33652200	0.66473000
H	5.25166500	4.54676300	0.49617700

H	3.61485500	5.04230800	0.04898300
H	3.97766600	4.54893600	1.72060600
H	-3.50258800	-0.49205500	2.41704300

1a'Rh
E (M06-SMD/BS1) = -3328.48870737 au
H (M06-SMD/BS1) = -3327.576226 au
G (M06-SMD/BS1) = -3327.719259 au
E (M06-SMD/BS2//M06-SMD/BS1) = -3330.50835357 au

C	1.68704900	-5.13505900	-1.52010700
C	0.69820800	-4.24787000	-1.94122200
C	0.38353100	-3.13467800	-1.17230700
C	1.04924300	-2.90261200	0.03958400
C	2.03848000	-3.80554100	0.46082400
C	2.35539300	-4.91048300	-0.31841200
H	1.93599400	-6.00277900	-2.12846000
H	0.17299700	-4.42071300	-2.87928600
H	-0.37872100	-2.43434500	-1.51066200
H	2.54647100	-3.63418400	1.40910900
H	3.12586800	-5.60230800	0.01715300
C	0.74429400	-1.77290400	0.87536800
C	0.55164400	-1.07342700	1.87942600
C	0.26979300	-0.38588200	3.09369900
H	1.10928600	0.12216000	3.57427500
C	-0.95300300	-0.31912200	3.64523000
H	-1.08879500	0.25547000	4.56399100
C	-2.17220200	-1.02156100	3.12352600
H	-1.86642700	-1.69951800	2.30765600
N	-3.10098300	0.01643900	2.57233100
C	-2.43469300	1.01998200	1.72008400
H	-1.74394800	1.58544100	2.35486700
H	-3.19338600	1.73580700	1.37910000
C	-1.74513600	0.45034700	0.54649800
C	-1.48509500	-0.17431600	-0.48794000
H	-1.64035900	-0.70994600	-1.41200700
S	-4.62515100	-0.45375000	1.98910000
O	-5.24570300	-1.28340600	3.01141700
O	-5.27984800	0.78025600	1.56868000
C	-4.34994500	-1.44491100	0.54169200
C	-3.81935000	-2.72728500	0.67572400
C	-4.59210300	-0.89302500	-0.71511400
C	-3.48859300	-3.44293200	-0.46939200
H	-3.66793900	-3.16986900	1.65947900
C	-4.28212800	-1.63436900	-1.84616100
H	-5.00649900	0.11026400	-0.80003200
C	-3.70423300	-2.90509700	-1.74189000
H	-3.05771000	-4.43973400	-0.37556500

H	-4.47103800	-1.21492000	-2.83523100
C	-3.31043600	-3.65140400	-2.97836100
H	-2.54058200	-3.09815600	-3.53755900
H	-2.91226000	-4.64557800	-2.74393500
H	-4.16389200	-3.77615100	-3.65841200
C	1.73929800	2.88878400	-2.33319900
H	1.33905700	3.35015700	-3.24760000
C	2.55221900	3.91401500	-1.55012000
H	1.94914600	4.82165900	-1.40226400
C	3.07850700	3.43776900	-0.20184600
H	3.73890400	4.21436600	0.20774700
C	3.84534900	2.11817900	-0.29297200
H	4.75234500	2.13120100	0.32737900
P	2.91099700	0.59039800	0.21345800
P	0.30227100	2.15945500	-1.42406500
Rh	0.55929200	0.40144200	0.08963400
C	-2.75469200	-1.83044600	4.27513400
H	-3.17129900	-1.16384600	5.04102800
H	-3.53807600	-2.52290900	3.95878700
H	-1.94293100	-2.41410800	4.72805300
C	-0.72572100	1.51768800	-2.79740400
C	-2.09650900	1.77176800	-2.89541000
C	-0.13614200	0.59954000	-3.67583200
C	-2.86313600	1.12356100	-3.86086600
H	-2.57629700	2.46790900	-2.20743500
C	-0.90549000	-0.05287300	-4.63337600
H	0.93059400	0.37869900	-3.60530000
C	-2.27257100	0.20600300	-4.72539700
H	-3.93008000	1.33225000	-3.92923500
H	-0.43667700	-0.76452400	-5.31129500
H	-2.87610100	-0.30700400	-5.47284800
C	-0.56236800	3.62514000	-0.76597700
C	-1.04215700	4.63078800	-1.61447500
C	-0.67390900	3.78405700	0.61742500
C	-1.64254300	5.76532700	-1.08004600
H	-0.95197800	4.52560700	-2.69625800
C	-1.27541700	4.92060800	1.15125100
H	-0.27692200	3.00702200	1.27556400
C	-1.76244400	5.90899900	0.30140100
H	-2.01808100	6.54182700	-1.74423800
H	-1.36183100	5.03368300	2.23061900
H	-2.23522600	6.79798900	0.71541700
C	3.43450300	0.38330700	1.95715500
C	3.74281300	-0.86695900	2.50287600
C	3.43565100	1.50767500	2.79239000
C	4.05887100	-0.98673600	3.85355100
H	3.75260500	-1.75262300	1.86839900

C	3.75014800	1.38510300	4.14229100
H	3.19218800	2.49226900	2.39086000
C	4.06189900	0.13672200	4.67575100
H	4.30735400	-1.96449300	4.26308100
H	3.75446900	2.26930800	4.77762000
H	4.31009000	0.04035800	5.73133900
C	3.78997800	-0.69188800	-0.74638900
C	5.11369200	-1.05152000	-0.46846100
C	3.14648700	-1.25146400	-1.85416200
C	5.76737200	-1.98059600	-1.27153500
H	5.63627400	-0.61074100	0.38106100
C	3.80778100	-2.16837100	-2.66619400
H	2.11251700	-0.97349900	-2.06895400
C	5.11538200	-2.54056400	-2.36830200
H	6.79321900	-2.26460600	-1.04275000
H	3.29518200	-2.60227600	-3.52343500
H	5.63106200	-3.26698200	-2.99429500
H	4.18698500	1.94118600	-1.32309500
H	2.24431200	3.35500200	0.51385400
H	3.40366900	4.20909400	-2.18230100
H	2.36759000	2.04574800	-2.65912100

C

E (M06-SMD/BS1) = -3328.54104925 au
H (M06-SMD/BS1) = -3327.628068 au
G (M06-SMD/BS1) = -3327.766787 au
E (M06-SMD/BS2//M06-SMD/BS1) = -3330.55682656 au

C	0.93095600	5.21170000	2.34773600
C	-0.22007000	4.53627400	2.74621400
C	-0.65191900	3.41544500	2.04319100
C	0.05214800	2.95502800	0.92139900
C	1.19656700	3.65709200	0.52052600
C	1.63748500	4.76635300	1.23155100
H	1.27126100	6.08654300	2.89953700
H	-0.79069900	4.88682100	3.60498300
H	-1.56957300	2.91193200	2.35076800
H	1.74181800	3.31282800	-0.35696100
H	2.53771800	5.28870300	0.90975800
C	-0.41463200	1.76752500	0.16506200
C	-0.16891500	1.68531300	-1.24332800
C	-0.82968800	0.59092200	-1.90589600
H	-0.68167600	0.45120300	-2.98144800
C	-1.74019300	-0.26152700	-1.25473400
C	-2.70078800	-1.28797300	-1.79498500
H	-3.31148800	-0.85874900	-2.60009400
N	-3.56881800	-1.58340400	-0.61646200
C	-2.78013500	-1.29865100	0.60595200

H	-2.27189000	-2.21996400	0.92104300
H	-3.42206500	-0.98474600	1.43685500
C	-1.80748000	-0.24602000	0.15088300
C	-1.11065500	0.74264600	0.87418400
H	-1.15810300	0.73366300	1.96598300
S	-5.10128400	-0.86957600	-0.63211900
O	-5.62989300	-1.09747500	-1.97138600
O	-5.79039300	-1.39010700	0.54172500
C	2.88440000	-2.69343600	1.18829700
H	3.11939500	-3.12324400	2.17184700
C	2.93164500	-3.82139700	0.16144900
H	2.26060400	-4.63444000	0.47147800
C	2.66513700	-3.46465200	-1.29767800
H	3.01817600	-4.30025300	-1.91729700
C	3.37224500	-2.19333900	-1.75483900
H	3.65192400	-2.26151400	-2.81468200
P	2.45141600	-0.58100400	-1.60908000
P	1.35665700	-1.69223300	1.44654800
Rh	0.49631200	-0.52031500	-0.35587200
C	-2.04575600	-2.55896900	-2.30691500
H	-1.49590800	-3.06734100	-1.50143700
H	-2.80696900	-3.24322900	-2.69966000
H	-1.33998700	-2.31762000	-3.11390300
H	4.31244400	-2.05666500	-1.20185300
H	1.58314500	-3.39908200	-1.49550000
H	3.94960100	-4.23406000	0.23209900
H	3.67070100	-1.94607800	0.99532500
C	-4.85028800	0.87136300	-0.39742100
C	-4.52630000	1.67414700	-1.49361500
C	-4.86613900	1.39427500	0.89375700
C	-4.19397600	3.00364000	-1.27993800
H	-4.54274100	1.26561500	-2.50278400
C	-4.53053100	2.72976400	1.08705600
H	-5.14644400	0.76514000	1.73661800
C	-4.17829600	3.54824500	0.01072100
H	-3.93835500	3.63767000	-2.12907000
H	-4.54336600	3.14707200	2.09396500
C	3.75410300	0.60257500	-1.14245300
C	4.88658500	0.75242700	-1.95444900
C	3.66561700	1.31023700	0.05441500
C	5.90931600	1.60718200	-1.56184700
H	4.96657300	0.20592000	-2.89482000
C	4.69290200	2.16372100	0.44764400
H	2.78354400	1.19051700	0.67976900
C	5.81441000	2.31168400	-0.36136700
H	6.78671500	1.72490300	-2.19526800
H	4.60790300	2.70938100	1.38712200

H	6.62051100	2.97821500	-0.05961300
C	2.00961900	-0.19475800	-3.33500300
C	1.49313300	-1.21406800	-4.14392400
C	2.09013200	1.10887600	-3.83090700
C	1.06111200	-0.93055800	-5.43462700
H	1.42124800	-2.23592600	-3.76726400
C	1.65969300	1.38642600	-5.12540400
H	2.48491300	1.91087100	-3.20841600
C	1.14232900	0.37139500	-5.92528300
H	0.66340000	-1.72835200	-6.05958800
H	1.72918800	2.40293900	-5.50838000
H	0.80417700	0.59363800	-6.93593900
C	1.84025200	-0.69338900	2.90398400
C	2.39067300	-1.31434800	4.03492200
C	1.68872300	0.69277800	2.90210200
C	2.78861900	-0.55094200	5.12622600
H	2.49558300	-2.39816700	4.08199600
C	2.09537900	1.45849500	3.99105600
H	1.25300800	1.18481500	2.03651400
C	2.64862000	0.83578700	5.10441400
H	3.21150500	-1.04380300	5.99974700
H	1.97282400	2.54094400	3.96191900
H	2.96858100	1.42782900	5.96003300
C	0.06927000	-2.84089700	2.04826600
C	-0.33159500	-3.90506400	1.22838800
C	-0.58172700	-2.63884800	3.26947100
C	-1.33754000	-4.77186700	1.64072700
H	0.12779000	-4.04813200	0.24919100
C	-1.59808900	-3.50193500	3.67249200
H	-0.29879900	-1.80727000	3.91396800
C	-1.97174400	-4.57176500	2.86527200
H	-1.63422900	-5.59860400	0.99788900
H	-2.09798000	-3.33466500	4.62490400
H	-2.76523100	-5.24502500	3.18414800
C	-3.78930200	4.97800600	0.21984400
H	-3.70922300	5.22416200	1.28541900
H	-2.82364200	5.19605000	-0.25654100
H	-4.52609100	5.65830700	-0.22969500
H	0.49779400	-1.98782700	-0.87954900

1aRh

E (M06-SMD/BS1) = -3328.48585980 au

H (M06-SMD/BS1) = -3327.573284 au

G (M06-SMD/BS1) = -3327.718500 au

E (M06-SMD/BS2//M06-SMD/BS1) = -3330.50525363 au

C	4.60786000	-2.60502000	-4.32373100
C	3.92609100	-3.05310800	-3.19362600

C	2.90293800	-2.28858600	-2.64572600
C	2.54424800	-1.06657900	-3.23657000
C	3.23309900	-0.62286500	-4.37558100
C	4.25971700	-1.39016700	-4.91112100
H	5.41082100	-3.20478900	-4.74857000
H	4.19575700	-4.00240400	-2.73318300
H	2.37354400	-2.62728600	-1.75396400
H	2.95595400	0.32717600	-4.82963900
H	4.79109400	-1.03848600	-5.79368500
C	1.51472800	-0.26290400	-2.66365200
C	0.66357700	0.43980800	-2.15041300
C	-0.30997000	1.26981400	-1.52243000
H	-1.26950800	1.34419900	-2.03991200
C	0.03436500	2.24676400	-0.57746800
H	-0.71260500	3.01236200	-0.36063400
C	1.45895000	2.74256800	-0.47503900
H	2.11983200	1.97812300	-0.91427100
N	1.89445900	3.00439600	0.93221300
C	1.26341900	2.13774500	1.93849500
H	0.25910800	2.52526100	2.15976800
H	1.83012800	2.20297600	2.87593500
C	1.18367200	0.74572000	1.47567700
C	1.31336100	-0.39297600	1.02393900
H	1.66444600	-1.39015700	0.81814800
S	3.57377700	3.15414900	1.16569700
O	4.06270000	4.09074000	0.16383000
O	3.74801000	3.44695300	2.58376000
C	4.28484900	1.56313300	0.83487200
C	4.54713500	1.18501400	-0.48209800
C	4.51527900	0.68642000	1.89500000
C	5.02336400	-0.09645800	-0.73332700
H	4.40281100	1.88853600	-1.30091000
C	5.00213500	-0.58416500	1.62443600
H	4.32653300	1.00325800	2.91912400
C	5.25141400	-0.99845300	0.31034700
H	5.23799300	-0.39972400	-1.75865000
H	5.19858900	-1.27234500	2.44677400
C	5.75054700	-2.38280700	0.03693600
H	6.57969900	-2.64636700	0.70655900
H	4.95801300	-3.12661500	0.20590600
H	6.09553200	-2.49060400	-0.99850100
C	-3.18571700	-1.83874100	2.15658700
H	-3.29541100	-2.86435000	2.53664700
C	-3.46254900	-0.85491000	3.28941600
H	-2.81450600	-1.08575600	4.14765500
C	-3.32959100	0.62061800	2.92868000
H	-3.72837200	1.21672200	3.76066500

C	-4.07296200	0.99245800	1.64533800
H	-4.49730100	2.00119700	1.71755900
P	-3.02861200	0.97568200	0.10116200
P	-1.50914500	-1.69859200	1.39662900
Rh	-0.78975100	0.37746100	0.43779700
C	1.50337800	4.01318600	-1.31899400
H	0.84073800	4.77524200	-0.88505700
H	2.51305600	4.42527600	-1.38927100
H	1.14675300	3.78406400	-2.33199300
H	-4.92390700	0.31640400	1.48023100
H	-2.26490700	0.90163300	2.83764300
H	-4.49289900	-1.04140900	3.62880300
H	-3.91638500	-1.71923100	1.34110100
C	-3.98180300	0.03284000	-1.13771200
C	-5.36003600	0.23909600	-1.28183700
C	-3.33239400	-0.88186900	-1.96976100
C	-6.07566200	-0.47633500	-2.23411700
H	-5.87285300	0.96964500	-0.65446100
C	-4.05234400	-1.59978000	-2.92192900
H	-2.25784200	-1.03651000	-1.86498200
C	-5.42287900	-1.39917000	-3.05104800
H	-7.14650900	-0.31270100	-2.34212500
H	-3.53818700	-2.31816400	-3.55870300
H	-5.98737000	-1.96015500	-3.79407100
C	-3.15472600	2.71189500	-0.47199200
C	-2.94165400	3.74422800	0.45169600
C	-3.29935100	3.02693200	-1.82573300
C	-2.87976700	5.06632700	0.02759200
H	-2.79847200	3.51298000	1.50954900
C	-3.23074400	4.35266900	-2.24867100
H	-3.46952400	2.23731900	-2.55844000
C	-3.01883500	5.37163300	-1.32572600
H	-2.71648200	5.86055200	0.75395700
H	-3.34653700	4.58711900	-3.30544600
H	-2.96386900	6.40651700	-1.65869000
C	-0.44385300	-2.38382800	2.72085800
C	0.01918100	-3.70197000	2.69106200
C	-0.11506900	-1.56733200	3.81029000
C	0.79328300	-4.19652200	3.73832000
H	-0.22631900	-4.35183600	1.85136500
C	0.64917700	-2.06677700	4.85872900
H	-0.45478400	-0.53044900	3.83487900
C	1.10702200	-3.38244400	4.82206800
H	1.15012900	-5.22460100	3.70499100
H	0.89455100	-1.42528100	5.70347700
H	1.71195400	-3.77135200	5.63944300
C	-1.51063700	-2.97240700	0.07962300

C	-2.47538200	-3.98351800	0.02092300
C	-0.52802500	-2.91598600	-0.91663700
C	-2.46305500	-4.90827600	-1.02042800
H	-3.25003500	-4.06218500	0.78135800
C	-0.51606500	-3.84076900	-1.95477900
H	0.21383000	-2.11595600	-0.90212100
C	-1.48793100	-4.83776600	-2.01051200
H	-3.22421700	-5.68582100	-1.05693500
H	0.25050800	-3.77337700	-2.72663900
H	-1.48546000	-5.55776200	-2.82716400

F

E (M06-SMD/BS1) = -3328.50092148 au
H (M06-SMD/BS1) = -3327.589699 au
G (M06-SMD/BS1) = -3327.730728 au
E (M06-SMD/BS2//M06-SMD/BS1) = -3330.52017502 au

C	0.55744000	5.71877300	1.75850900
C	0.00238200	4.70887400	2.54330000
C	-0.37355100	3.50392600	1.96489700
C	-0.19855400	3.30241700	0.58473500
C	0.36349300	4.32244800	-0.19956900
C	0.73892400	5.52198200	0.39005100
H	0.84821300	6.66349900	2.21477500
H	-0.13556600	4.86103000	3.61272900
H	-0.80974000	2.70364400	2.56364200
H	0.49192200	4.16268200	-1.26955000
H	1.17039900	6.31209400	-0.22220800
C	-0.62716500	2.07690900	0.00892800
C	-1.08464700	1.02405800	-0.40919400
C	-1.69540500	-0.18688500	-0.82896100
H	-2.01735000	-0.26099200	-1.87056000
C	-2.08523500	-1.16563800	0.04957800
H	0.73485100	-2.35244700	-0.03644400
C	-3.11520400	-2.23040400	-0.21573600
H	-3.79410100	-1.96238200	-1.03507000
N	-3.85715900	-2.21768800	1.08215500
C	-2.88958000	-1.93194100	2.17417000
H	-2.60875400	-2.87504800	2.66140200
H	-3.34965100	-1.30083600	2.94310100
C	-1.72093400	-1.28931400	1.46262100
C	-0.42708800	-1.10396800	1.76652000
H	-0.02428800	-1.33110400	2.75789000
S	-5.29661900	-1.34361600	1.10969800
O	-6.07171000	-1.82482500	-0.02770900
O	-5.80586000	-1.47330800	2.46950500
C	-4.89841000	0.36328100	0.82542400
C	-4.88995900	0.85526200	-0.47949500

C	-4.51909500	1.17082700	1.89791500
C	-4.48019800	2.16232800	-0.70604300
H	-5.20665400	0.22239600	-1.30662900
C	-4.10778700	2.47437700	1.65046500
H	-4.56069100	0.79025900	2.91677100
C	-4.07477500	2.98674700	0.34942200
H	-4.46918300	2.55384800	-1.72341500
H	-3.80936300	3.11150700	2.48327700
C	-3.63568200	4.39441200	0.09110900
H	-4.49630300	5.07911700	0.09192300
H	-2.93793300	4.74767300	0.86175500
H	-3.14910100	4.48855000	-0.88815400
C	4.02000000	-1.16258700	-0.14173700
C	4.19240800	-2.56672500	-0.71719700
C	3.08971400	-3.08803600	-1.63420100
C	2.69192400	-2.13268100	-2.75585500
P	1.45967200	-0.80103700	-2.30306300
P	2.57599100	-0.91006800	0.97257300
Rh	0.55917600	-0.82447900	-0.02316100
C	-2.52907400	-3.60917700	-0.45944700
H	-1.85727000	-3.90588400	0.35801000
H	-3.33347700	-4.35006600	-0.53732600
H	-1.95396100	-3.61153800	-1.39472900
H	3.96121500	-0.41274400	-0.94456800
H	4.90796500	-0.90707700	0.45682900
H	5.13451200	-2.54900800	-1.28627300
H	4.34877500	-3.28225200	0.10372300
H	3.45604900	-4.01827000	-2.09119800
H	2.20024300	-3.37752100	-1.05285300
H	2.24883600	-2.69275100	-3.58982100
H	3.58334000	-1.63020800	-3.15924300
C	2.24387600	0.74676000	-2.88618900
C	2.79177400	0.83856000	-4.17288400
C	2.32372700	1.84783500	-2.03098200
C	3.41811700	2.00853800	-4.58540200
H	2.72116000	-0.00771600	-4.85792300
C	2.96376200	3.01636200	-2.44090400
H	1.88913500	1.79087100	-1.03088500
C	3.51112300	3.09611800	-3.71712300
H	3.83898600	2.07277200	-5.58739800
H	3.03433300	3.86032000	-1.75443400
H	4.01138100	4.00755300	-4.04028700
C	2.82817500	-2.18236300	2.25788500
C	3.92018700	-2.04818200	3.12569100
C	2.03503400	-3.33014100	2.32590400
C	4.20405900	-3.04561500	4.05115100
H	4.54988400	-1.15881900	3.08377200

C	2.32443400	-4.32828500	3.25214700
H	1.18025200	-3.44286600	1.65713700
C	3.40654500	-4.18608200	4.11503900
H	5.05120000	-2.93141700	4.72503800
H	1.69883300	-5.21777700	3.29945400
H	3.62961400	-4.96583000	4.84114900
C	2.88521300	0.69057700	1.78517300
C	3.66750100	1.67709300	1.17405900
C	2.26773000	0.96465600	3.01306300
C	3.83008800	2.91792100	1.78480700
H	4.15539100	1.49008100	0.21839100
C	2.44633300	2.19942500	3.62452200
H	1.65480400	0.20509600	3.49882100
C	3.22373300	3.17929800	3.00952600
H	4.43840200	3.68007100	1.30010500
H	1.96874800	2.40136300	4.58218700
H	3.35313900	4.14994600	3.48578200
C	0.08217400	-1.07844900	-3.47639400
C	-0.47919100	-2.35845700	-3.55474100
C	-0.52757600	-0.02235600	-4.15911900
C	-1.62919800	-2.57852300	-4.30537900
H	-0.02261800	-3.19044600	-3.01367300
C	-1.67786100	-0.24602900	-4.91261200
H	-0.11070700	0.98326200	-4.09678000
C	-2.23253300	-1.52099500	-4.98405600
H	-2.05633600	-3.57897500	-4.35931300
H	-2.14288000	0.58242200	-5.44459300
H	-3.13396200	-1.69279600	-5.56973100

A
E (M06-SMD/BS1) = -3328.52956670 au
H (M06-SMD/BS1) = -2123.246836 au
G (M06-SMD/BS1) = -2123.321317 au
E (M06-SMD/BS2//M06-SMD/BS1) = -3330.54405134 au

C	-2.63486600	-3.16842300	4.84895300
C	-3.37157300	-2.09622900	4.34787700
C	-2.74752200	-1.10792200	3.59814000
C	-1.36958600	-1.18858600	3.33605600
C	-0.63477700	-2.27521000	3.84152500
C	-1.26645300	-3.25280400	4.59690200
H	-3.12786700	-3.93722100	5.44131000
H	-4.43816900	-2.02341700	4.55269800
H	-3.31461000	-0.25347200	3.23019700
H	0.43368700	-2.33854600	3.63274000
H	-0.68947800	-4.08996800	4.98640100
C	-0.69115100	-0.17272500	2.60699300
C	-0.05418300	0.78248400	2.16149500

C	0.80618900	1.66334100	1.47027300
H	1.78380500	1.78372700	1.95045000
C	0.27346300	2.92630700	0.80746700
H	1.05824100	3.70385200	0.83224400
C	-1.01569300	3.53698300	1.38648300
H	-1.63671900	2.71618700	1.77769400
N	-1.69687200	4.14134900	0.19201700
C	-0.93755300	3.82577000	-1.04875600
H	-0.31619500	4.69678800	-1.31028000
H	-1.60074500	3.62049300	-1.89658500
C	-0.10600100	2.66308000	-0.61292900
C	0.10671700	1.47622900	-1.18210700
H	-0.14669900	1.23708300	-2.21804500
S	-3.35047500	3.92298400	0.10343300
O	-3.86006800	4.13607600	1.45381700
O	-3.81610100	4.74780200	-1.00579100
C	-3.65496500	2.22352300	-0.33562800
C	-3.65464300	1.23433200	0.64849300
C	-3.89756800	1.89739100	-1.67011400
C	-3.89397300	-0.08576200	0.28358300
H	-3.50762800	1.49434400	1.69638400
C	-4.14829000	0.57505600	-2.01301600
H	-3.92767900	2.68103600	-2.42461800
C	-4.14956100	-0.43670800	-1.04627000
H	-3.91054100	-0.86194000	1.05139100
H	-4.36852200	0.32215400	-3.05025400
C	-4.42590400	-1.85870900	-1.42488400
H	-4.81294500	-2.43342600	-0.57356300
H	-5.15114400	-1.92087000	-2.24633600
H	-3.50887700	-2.36021400	-1.77161200
C	1.99691200	-2.65912500	-1.95985500
H	1.90421000	-3.74701100	-2.09186600
C	2.37793900	-2.02017700	-3.29280400
H	1.70027000	-2.39846300	-4.07230300
C	2.36263300	-0.49843700	-3.35193600
H	2.62758600	-0.20826200	-4.37859200
C	3.34171200	0.21401000	-2.42453900
H	3.56439100	1.20809200	-2.83153100
P	2.85806300	0.51449600	-0.65494300
P	0.41668300	-1.99872800	-1.26166700
Rh	0.75315400	0.06097000	0.04413200
C	-0.77440100	4.56114300	2.46955900
H	-0.18476000	5.40057200	2.07630800
H	-1.71951300	4.95212800	2.86304200
H	-0.21559900	4.10351600	3.29857200
C	-0.12640000	-3.28291800	-0.07133900
C	-1.46064500	-3.22742500	0.35487300

C	0.70368900	-4.29360200	0.41930200
C	-1.97158700	-4.19342700	1.21001100
H	-2.10300300	-2.41768600	0.00022800
C	0.19313900	-5.25213300	1.29565000
H	1.74780900	-4.35312700	0.11257800
C	-1.14367400	-5.21433500	1.67770200
H	-3.01467400	-4.14331500	1.52218500
H	0.84507800	-6.04108300	1.66762500
H	-1.54040300	-5.97053500	2.35357600
C	-0.75416400	-2.26942700	-2.65135100
C	-0.93734600	-3.56768800	-3.14777300
C	-1.43540800	-1.20954600	-3.25061500
C	-1.77724500	-3.79333400	-4.23122600
H	-0.42201800	-4.40844300	-2.68130500
C	-2.27072600	-1.43612400	-4.34381700
H	-1.33240500	-0.20089200	-2.85355300
C	-2.44095400	-2.72549800	-4.83497200
H	-1.91390500	-4.80566100	-4.60791700
H	-2.79291700	-0.60009400	-4.80714900
H	-3.09504300	-2.90312500	-5.68697500
C	3.45147500	2.21510700	-0.34238000
C	4.15337100	2.53012100	0.82572200
C	3.11675900	3.23937800	-1.23713200
C	4.51770800	3.84756800	1.09062500
H	4.42170400	1.74677000	1.53465500
C	3.48233600	4.55352500	-0.96899500
H	2.55873000	3.01798800	-2.14688300
C	4.18148200	4.86000500	0.19676400
H	5.06911600	4.08092000	1.99973500
H	3.21994300	5.34093600	-1.67355900
H	4.46682900	5.88947000	0.40600900
C	3.92644700	-0.56372400	0.36786600
C	5.29610800	-0.67713300	0.10015500
C	3.37641000	-1.28518700	1.43494300
C	6.09448200	-1.50798300	0.87822500
H	5.74859800	-0.10137900	-0.70810200
C	4.17690800	-2.12202900	2.20720900
H	2.31273400	-1.20014800	1.67495200
C	5.53521700	-2.23511500	1.92696200
H	7.15892600	-1.58735300	0.66505600
H	3.73604400	-2.68324800	3.02950200
H	6.16285300	-2.88853400	2.53034200
H	4.29991900	-0.32693400	-2.39921200
H	1.34398900	-0.11279200	-3.19419100
H	3.38373700	-2.37733700	-3.56281700
H	2.78012100	-2.49737800	-1.20291800

B

E (M06-SMD/BS1) = -3328.50559365 au
H (M06-SMD/BS1) = -3327.591702 au
G (M06-SMD/BS1) = -3327.736694 au
E (M06-SMD/BS2//M06-SMD/BS1) = -3330.51918672 au

C	-0.11570400	-2.61775900	4.30363600
C	-0.66087600	-2.98048100	3.07165800
C	-0.82652800	-2.02770500	2.07653000
C	-0.44987100	-0.69729600	2.31267900
C	0.09488600	-0.33392100	3.55244500
C	0.25614800	-1.29604900	4.54357900
H	0.01951000	-3.36889100	5.07983400
H	-0.95516600	-4.01236200	2.88666200
H	-1.23673300	-2.29971100	1.10328100
H	0.39353800	0.69987700	3.72473700
H	0.67883000	-1.01333000	5.50595900
C	-0.55599400	0.27418100	1.26145700
C	-0.99191500	1.35662800	0.75285700
C	-1.08468600	2.14900500	-0.36240100
H	-0.55922200	3.10842300	-0.38305000
C	-2.13465300	1.88205800	-1.40998100
H	-1.82026300	2.40038000	-2.33331600
C	-3.56302700	2.35965500	-1.06248600
H	-3.66848600	2.35160100	0.03507600
N	-4.44496100	1.30995600	-1.65110700
C	-3.64909700	0.22110800	-2.26844800
H	-3.64360900	0.37115300	-3.35861100
H	-4.08542700	-0.76544400	-2.07626700
C	-2.28732900	0.40306900	-1.67115700
C	-1.37335000	-0.50295700	-1.31859700
H	-1.53422500	-1.57879800	-1.44296300
S	-5.76414800	0.86099300	-0.73473300
O	-6.42708100	2.10139100	-0.34704500
O	-6.47436200	-0.15175100	-1.50748900
C	-5.15111200	0.09488900	0.74674000
C	-4.93965600	0.87573000	1.88447100
C	-4.87587700	-1.27174000	0.75615400
C	-4.45651200	0.27431100	3.03772300
H	-5.18625300	1.93566500	1.87377700
C	-4.39898100	-1.85696300	1.92334400
H	-5.07029900	-1.87940000	-0.12590000
C	-4.19038400	-1.09874700	3.07941100
H	-4.30648300	0.87381200	3.93600500
H	-4.20362700	-2.92950200	1.94618800
C	-3.71177500	-1.72973500	4.34916100
H	-4.37948500	-1.48145400	5.18530000
H	-3.66087100	-2.82186500	4.26683000

H	-2.71107700	-1.36384800	4.62138100
C	3.06407500	-1.09293600	-2.39595300
C	2.88111700	-0.11996200	-3.56081600
C	2.10928600	1.17429100	-3.33408700
C	2.67933500	2.18501400	-2.33357400
P	2.45203600	1.77793800	-0.53533400
P	1.64574200	-1.54706500	-1.28375700
Rh	0.34092100	0.35296300	-0.66936700
C	-3.88283300	3.73365300	-1.60471400
H	-3.80808500	3.73701600	-2.70074700
H	-4.89621700	4.04194600	-1.32460600
H	-3.17174600	4.47131300	-1.20635700
H	3.72620800	2.42326700	-2.57167000
H	2.11978000	3.12516100	-2.45186700
H	2.05112000	1.68685300	-4.30558900
H	1.05193100	0.94740200	-3.08090900
H	3.89441600	0.13922200	-3.90928200
H	2.41156800	-0.64616300	-4.40384200
H	3.47490800	-2.03909200	-2.77897800
H	3.81320900	-0.70588400	-1.68951800
C	2.53501500	-2.46729400	0.02098600
C	3.26917200	-3.61611500	-0.30596400
C	2.52273700	-2.01681400	1.34033200
C	3.96957900	-4.30030400	0.67962900
H	3.28564900	-3.98432900	-1.33208300
C	3.22509500	-2.70441100	2.32772300
H	1.95554600	-1.12372300	1.59817600
C	3.94691300	-3.84584300	1.99807800
H	4.53528500	-5.19317700	0.41889700
H	3.20221200	-2.34249700	3.35528300
H	4.49445300	-4.38646700	2.76843300
C	0.67503800	-2.82032700	-2.15735000
C	0.32727500	-2.62806800	-3.49845300
C	0.13759000	-3.90877900	-1.46168800
C	-0.52281500	-3.52257000	-4.14051600
H	0.70541000	-1.76473100	-4.04631400
C	-0.71813200	-4.79838000	-2.10496700
H	0.38762400	-4.06629200	-0.41178300
C	-1.04736300	-4.60880000	-3.44459400
H	-0.77959600	-3.36716800	-5.18702400
H	-1.12780600	-5.64470200	-1.55612300
H	-1.71600000	-5.30640400	-3.94595300
C	4.06966900	1.21020700	0.12946000
C	4.11421900	0.90527700	1.49525400
C	5.23720100	1.09447900	-0.62920500
C	5.30187500	0.49877400	2.09159100
H	3.20786600	0.99217400	2.09983900

C	6.42478600	0.67632400	-0.03215000
H	5.23315700	1.32100300	-1.69524000
C	6.46005600	0.37926000	1.32635800
H	5.32120800	0.26597800	3.15529300
H	7.32702500	0.58702600	-0.63506800
H	7.38949100	0.05385000	1.79046900
C	2.36491800	3.42950600	0.26566200
C	3.15298000	4.49793700	-0.17728600
C	1.54649500	3.60392000	1.38562600
C	3.10574000	5.72325500	0.47878800
H	3.81677100	4.37271600	-1.03337800
C	1.50415900	4.83028200	2.04478300
H	0.93459200	2.77423500	1.74417500
C	2.28052200	5.89115200	1.58924300
H	3.72105500	6.54939100	0.12592800
H	0.86152600	4.95564500	2.91470500
H	2.24682600	6.85093800	2.10203900

C

E (M06-SMD/BS1) = -3328.53562134 au
H (M06-SMD/BS1) = -3327.619917 au
G (M06-SMD/BS1) = -3327.759636 au
E (M06-SMD/BS2//M06-SMD/BS1) = -3330.54558116 au

C	-1.81079000	3.41937700	-4.33594500
C	-2.56146000	3.12741100	-3.19776000
C	-2.17934000	2.09181400	-2.35644000
C	-1.03490100	1.32984000	-2.64295900
C	-0.27808700	1.64584800	-3.78336000
C	-0.67085000	2.67330100	-4.62920300
H	-2.11082500	4.23626300	-4.99003400
H	-3.45046600	3.71207000	-2.96659500
H	-2.79080500	1.85278000	-1.48605100
H	0.63529800	1.08506900	-3.97638900
H	-0.07605200	2.90718100	-5.51023400
C	-0.61135800	0.23734600	-1.77202100
C	0.39723300	-0.67821800	-2.14907900
C	0.46245200	-1.92896400	-1.58215500
H	1.31256200	-2.58939300	-1.75446100
C	-0.58542100	-2.38515400	-0.59900900
H	-0.06525100	-3.08210900	0.08466900
C	-1.95289100	-3.01421500	-0.89670600
H	-2.43152600	-2.45124500	-1.71783000
N	-2.65077100	-2.73245900	0.40430200
C	-1.93950500	-1.69682300	1.21661200
H	-1.43496100	-2.17835700	2.06473700
H	-2.62508500	-0.93677200	1.61060800
C	-0.98126000	-1.17057500	0.19548700

C	-1.10617600	0.09359900	-0.42441400
H	-1.68027100	0.88243500	0.06746700
S	-4.31523600	-2.64065900	0.42767900
O	-4.79753100	-3.65883800	-0.49781600
O	-4.70129400	-2.66791400	1.83359100
C	2.38230600	1.14097200	3.10083200
H	2.19425900	1.96225100	3.80657700
C	2.68026400	-0.13074600	3.89001200
H	1.89476800	-0.29399400	4.64048400
C	2.88176500	-1.40043700	3.07229000
H	3.18670300	-2.20373800	3.75664100
C	3.94409900	-1.24250700	1.98229300
H	4.55378900	-2.15023400	1.88746900
P	3.27978200	-0.87209400	0.28789100
P	0.98113900	1.13433200	1.89812700
Rh	0.99186400	-0.25990800	0.04409400
C	-1.88197000	-4.48805000	-1.21348600
H	-1.44142300	-5.03497900	-0.36891600
H	-2.87260300	-4.90439300	-1.42158000
H	-1.24716200	-4.64298000	-2.09698500
H	4.64099700	-0.43229400	2.24015000
H	1.92433300	-1.72850500	2.63003600
H	3.60078500	0.06484100	4.46155500
H	3.25313700	1.44756300	2.49851000
C	-4.76713100	-1.04844900	-0.21816800
C	-4.92551700	-0.88395200	-1.59379300
C	-4.95686500	0.02072200	0.65927900
C	-5.28927200	0.36328100	-2.08724000
H	-4.79674700	-1.72827100	-2.26851100
C	-5.31364400	1.26032100	0.14649100
H	-4.85281800	-0.12585600	1.73349400
C	-5.49727200	1.44683700	-1.22857300
H	-5.42316000	0.49975300	-3.16033400
H	-5.47432200	2.09769200	0.82626700
C	4.41869100	0.36421200	-0.42218300
C	5.80203700	0.27037400	-0.22701200
C	3.90188800	1.40951800	-1.19136300
C	6.64885100	1.22423000	-0.77942900
H	6.22099300	-0.55475500	0.35029400
C	4.75179900	2.36455600	-1.74300100
H	2.82519100	1.46935200	-1.36298900
C	6.12414500	2.27387600	-1.53244400
H	7.72359500	1.14832700	-0.62332200
H	4.33835800	3.17985600	-2.33458700
H	6.79101100	3.02104200	-1.95950600
C	3.62247300	-2.40578300	-0.64708800
C	3.32712200	-3.64478100	-0.06615700

C	4.04627500	-2.35322600	-1.97772300
C	3.46470600	-4.81476800	-0.80558500
H	2.97750900	-3.70261700	0.96652400
C	4.18388100	-3.52711000	-2.71355300
H	4.26498900	-1.39288600	-2.44596300
C	3.89313400	-4.75742300	-2.13026000
H	3.23673100	-5.77475100	-0.34523100
H	4.52074200	-3.47836800	-3.74767500
H	3.99994600	-5.67379900	-2.70814100
C	0.98935600	2.86617500	1.30073700
C	1.11781400	3.94304500	2.18722800
C	0.83228100	3.12538300	-0.06361400
C	1.09406500	5.24874900	1.71019000
H	1.21933800	3.76952000	3.25859600
C	0.80438500	4.43259300	-0.54067800
H	0.73256400	2.28858000	-0.75600200
C	0.93735900	5.49546900	0.34730800
H	1.19373100	6.07875800	2.40751000
H	0.67466900	4.61592200	-1.60668800
H	0.91650900	6.51994500	-0.02044600
C	-0.54569000	1.00195200	2.90403400
C	-0.72967000	-0.10768700	3.73918700
C	-1.57681600	1.94251000	2.79066300
C	-1.90753600	-0.26071500	4.46313200
H	0.03927300	-0.87686800	3.81061300
C	-2.76030300	1.77912800	3.50593000
H	-1.46050200	2.80876600	2.13957700
C	-2.92696200	0.68049400	4.34579600
H	-2.03343100	-1.12655500	5.11085200
H	-3.55358300	2.51911600	3.40933300
H	-3.85185600	0.55636400	4.90649200
C	-5.92212600	2.78085200	-1.75763900
H	-5.33122700	3.59358200	-1.31361600
H	-5.82537700	2.83309900	-2.84862300
H	-6.97364900	2.98242700	-1.50814800

E
E (M06-SMD/BS1) = -3328.59105958 au
H (M06-SMD/BS1) = -3327.677431 au
G (M06-SMD/BS1) = -3327.816530 au
E (M06-SMD/BS2//M06-SMD/BS1) = -3330.60318921 au

C	-2.26536400	4.28210900	-1.40640200
C	-1.92333600	3.57002200	-2.55957100
C	-0.91556200	2.61750200	-2.52195200
C	-0.23067700	2.35017500	-1.32375900
C	-0.59910400	3.05598000	-0.16278100
C	-1.60882300	4.02144500	-0.21235900

H	-3.04824100	5.03805600	-1.44421400
H	-2.44355700	3.77033500	-3.49506600
H	-0.63714700	2.07271200	-3.42457000
H	-0.02160300	2.91573100	0.75142300
H	-1.85993500	4.58393300	0.68523800
C	0.89760900	1.38785800	-1.27445300
C	0.82393100	0.35036000	-0.32238100
C	1.92554100	-0.50200300	-0.18902200
H	1.93611000	-1.31676800	0.53407400
C	3.01923800	-0.34563400	-1.02945100
C	4.25322600	-1.20103200	-1.11193500
H	4.70576300	-1.37477600	-0.12592400
N	5.17274400	-0.37870100	-1.95455900
C	4.35643800	0.57359400	-2.74845000
H	4.22465400	0.16524100	-3.76145800
H	4.87597800	1.53247200	-2.86139400
C	3.05890900	0.65262600	-1.99822900
C	1.99547700	1.53262200	-2.13183400
H	2.01307000	2.34385600	-2.86218300
S	6.49325600	0.25747800	-1.14067000
O	7.20797800	-0.88537100	-0.58330600
O	7.15481200	1.16211000	-2.07468700
C	-2.47150100	-2.42712300	2.07161700
H	-2.25029400	-3.49444800	2.22820900
C	-2.65150800	-1.75978600	3.43047900
H	-1.72978800	-1.85328800	4.02320800
C	-3.11467300	-0.30631800	3.41461900
H	-3.48319100	-0.05870000	4.41966000
C	-4.22144100	-0.02108700	2.40058200
H	-4.82762700	0.83114200	2.73041900
P	-3.58293300	0.41976800	0.70586800
P	-1.09901300	-1.83874600	0.99106100
Rh	-1.13950400	0.30360700	0.28349600
C	3.98583000	-2.54270900	-1.77229000
H	3.55642200	-2.40356500	-2.77473000
H	4.91483200	-3.11895000	-1.86507300
H	3.27101600	-3.12305900	-1.17110500
H	-4.91226400	-0.87305300	2.32056800
H	-2.26521700	0.37041800	3.23108900
H	-3.40937400	-2.35915000	3.95731600
H	-3.40417400	-2.38269300	1.49213400
C	5.84829800	1.21238900	0.21093600
C	5.68701600	0.61183600	1.45835300
C	5.39567900	2.51140500	-0.02231000
C	5.04889300	1.31576100	2.47184000
H	6.06307900	-0.39445900	1.63335800
C	4.75913100	3.19833100	1.00178800

H	5.54157800	2.98126600	-0.99357800
C	4.56905700	2.61095300	2.25824200
H	4.91917200	0.85167700	3.44961200
H	4.40175400	4.21347800	0.82848300
C	-4.33265900	-0.70332400	-0.52195500
C	-5.55317700	-1.36365000	-0.35789500
C	-3.61649300	-0.86812600	-1.71312800
C	-6.03742500	-2.19122300	-1.36718700
H	-6.13367600	-1.23107900	0.55545300
C	-4.11000900	-1.67963700	-2.72964900
H	-2.65637000	-0.35512100	-1.83678400
C	-5.31921200	-2.34783400	-2.55093800
H	-6.98486600	-2.71027500	-1.23189300
H	-3.54493600	-1.80273100	-3.65230100
H	-5.70434900	-2.99408400	-3.33799600
C	-4.43337000	2.01600000	0.39514000
C	-4.30255300	3.03296200	1.34913500
C	-5.18109300	2.25722400	-0.75974300
C	-4.93761700	4.25596600	1.16820200
H	-3.69850400	2.86643000	2.24456000
C	-5.80955900	3.48752600	-0.94326100
H	-5.28907400	1.48136600	-1.51759700
C	-5.69599500	4.48436800	0.02085900
H	-4.83808700	5.03444600	1.92324800
H	-6.39825900	3.66159100	-1.84260900
H	-6.19598800	5.44110700	-0.12097000
C	-1.19585200	-2.93890100	-0.47094100
C	-2.19503500	-3.91320000	-0.58870400
C	-0.27319100	-2.78533100	-1.51637700
C	-2.26593100	-4.71484600	-1.72431300
H	-2.93520300	-4.05975900	0.19435300
C	-0.34407000	-3.59410300	-2.64457800
H	0.51145400	-2.03185300	-1.44834500
C	-1.34319400	-4.55874200	-2.75312900
H	-3.05221300	-5.46390400	-1.80149500
H	0.38488000	-3.46682300	-3.44332700
H	-1.40145400	-5.18718000	-3.64004400
C	0.36380200	-2.35761200	1.95286100
C	0.86182700	-1.52548500	2.96101300
C	0.97387700	-3.59279100	1.71281600
C	1.96665300	-1.91669400	3.70839000
H	0.40319600	-0.55272500	3.14519600
C	2.08599700	-3.97586300	2.45770500
H	0.59437800	-4.25271600	0.93273100
C	2.58596300	-3.13748200	3.45002700
H	2.35170700	-1.26065300	4.48701300
H	2.56525700	-4.93236700	2.25668400

H	3.46093100	-3.43623700	4.02468400
C	3.89216300	3.36958500	3.35696100
H	4.60380500	4.02968700	3.87345100
H	3.08765000	4.00638200	2.96774100
H	3.46766200	2.69400300	4.10951500
H	-0.66251100	0.61638000	1.70305900

2aRh

E (M06-SMD/BS1) = -3328.63192045 au
H (M06-SMD/BS1) = -3327.715184 au
G (M06-SMD/BS1) = -3327.859623 au
E (M06-SMD/BS2//M06-SMD/BS1) = -3330.64134678 au

C	2.34874300	-3.15007600	3.59896100
C	1.77070700	-3.60935400	2.42319800
C	0.77934800	-2.86407100	1.78608100
C	0.33864900	-1.63840100	2.32091900
C	0.94497800	-1.18656600	3.51239300
C	1.93061900	-1.93208900	4.13995700
H	3.12062700	-3.73439200	4.09701100
H	2.08659700	-4.55668000	1.98932700
H	0.34662700	-3.25181800	0.86527300
H	0.60977100	-0.26085900	3.97701800
H	2.36916300	-1.56690500	5.06702200
C	-0.70752400	-0.83914900	1.63008200
C	-0.85197900	0.54565000	1.87909900
C	-1.81333600	1.31303600	1.20611100
H	-1.89765900	2.37793700	1.42585200
C	-2.63889300	0.70383800	0.28211400
C	-3.70090300	1.32512500	-0.57701700
H	-4.54175600	1.67768500	0.04591000
N	-4.12855600	0.16801800	-1.41422100
C	-3.50804100	-1.10510700	-0.99380800
H	-3.04945800	-1.61387800	-1.85315700
H	-4.24434500	-1.79908700	-0.55452700
C	-2.51114300	-0.66425300	0.02786300
C	-1.56655200	-1.43425700	0.67485900
H	-1.50930400	-2.50264300	0.47033600
S	-5.70481300	0.10069000	-1.93620200
O	-6.05044100	1.44363600	-2.38823200
O	-5.77609500	-1.03570700	-2.84761700
C	3.09605900	-0.17056300	-2.59251300
H	3.80511700	-0.80820100	-3.14003500
C	1.88107700	0.07935300	-3.48854500
H	1.56220500	-0.87629300	-3.93054500
C	0.65159100	0.74319800	-2.87779400
H	-0.05796500	0.93351200	-3.69657200
C	0.85928700	2.05944000	-2.13414900

H	-0.13352200	2.49995700	-1.96619100
P	1.58210300	1.88450500	-0.43886400
P	2.78710900	-0.97756200	-0.96289800
Rh	1.23384800	-0.15730500	0.42985200
C	-3.18120800	2.46833200	-1.42963700
H	-2.37119300	2.10856300	-2.08073900
H	-3.97745000	2.87984400	-2.05930500
H	-2.79073700	3.27323600	-0.79104400
H	1.42769800	2.78132700	-2.73787600
H	0.14289700	0.04713300	-2.19092700
H	2.23570400	0.69459600	-4.32973900
H	3.62614200	0.76884100	-2.37469100
H	-0.24833000	1.03283700	2.64117300
C	0.84032000	3.25017000	0.54486100
C	1.07779500	3.26368500	1.92470000
C	0.10628200	4.29180800	-0.02847000
C	0.56161500	4.27879400	2.72086600
H	1.66503900	2.46416300	2.38153100
C	-0.41360200	5.30768200	0.77237900
H	-0.06531700	4.33076400	-1.10330700
C	-0.19498200	5.29913200	2.14600500
H	0.74909500	4.27411400	3.79335700
H	-0.98833700	6.11093700	0.31406400
H	-0.60561000	6.09164600	2.76925300
C	3.32301000	2.48011900	-0.49587300
C	3.75480300	3.44389300	-1.41372200
C	4.21799300	2.01099700	0.46987200
C	5.06925700	3.89902600	-1.38444200
H	3.06966800	3.85106500	-2.15715400
C	5.53118700	2.47091200	0.50159800
H	3.88241700	1.26920800	1.19556300
C	5.96050200	3.40913600	-0.43210400
H	5.39833900	4.64203200	-2.10902700
H	6.21948800	2.08728800	1.25353700
H	6.98940400	3.76458900	-0.41614000
C	4.44751500	-1.19940100	-0.23492200
C	5.63144700	-1.13394600	-0.97434400
C	4.51162000	-1.46371100	1.13819700
C	6.85918500	-1.32250500	-0.34364800
H	5.61148900	-0.93352100	-2.04424100
C	5.73613800	-1.66147200	1.76392400
H	3.58725200	-1.50265000	1.71835300
C	6.91381300	-1.58679600	1.02178900
H	7.77743800	-1.26332600	-0.92548700
H	5.77016200	-1.86876400	2.83284200
H	7.87603300	-1.73369100	1.50964100
C	2.23254600	-2.67553900	-1.38483500

C	3.05396600	-3.78881400	-1.17880700
C	0.93401500	-2.86991400	-1.87624700
C	2.58119500	-5.07035500	-1.44978500
H	4.06602400	-3.66222600	-0.79560000
C	0.46706900	-4.15058500	-2.15109500
H	0.27211700	-2.01700600	-2.02973300
C	1.28819200	-5.25449000	-1.93050000
H	3.22908700	-5.92892900	-1.28073700
H	-0.54396000	-4.28589100	-2.53259600
H	0.92009600	-6.25829100	-2.13568900
C	-6.70245800	-0.27185800	-0.51604400
C	-6.95722900	-1.60242100	-0.18357200
C	-7.18497100	0.76946300	0.27739500
C	-7.69326100	-1.88440000	0.95986100
H	-6.60185700	-2.40640300	-0.82550200
C	-7.91881800	0.46667700	1.41640000
H	-7.00614700	1.80481900	-0.00687500
C	-8.18245800	-0.85926500	1.77539300
H	-7.89999000	-2.92135100	1.22440100
H	-8.30294600	1.27461500	2.03902900
C	-9.00122800	-1.17283800	2.98845600
H	-10.07007200	-1.22257400	2.73529400
H	-8.72578800	-2.14257300	3.42064600
H	-8.88776100	-0.40238700	3.76089000

$TS_{1'aRh\text{-}B}$

E (M06-SMD/BS1) = -3328.45033328 au
H (M06-SMD/BS1) = -3327.540668 au
G (M06-SMD/BS1) = -3327.683323 au
E (M06-SMD/BS2//M06-SMD/BS1) = -3330.46646085 au

C	-0.15943100	-1.20362600	4.96509900
C	-0.06488300	-0.03611800	4.20949400
C	0.03692100	-0.10547200	2.82527200
C	0.03225400	-1.34981300	2.18140900
C	-0.05149200	-2.52266400	2.94689400
C	-0.14675600	-2.44467900	4.33085700
H	-0.24460500	-1.14679300	6.04885500
H	-0.07520400	0.93726900	4.69955400
H	0.10938900	0.80510100	2.22680900
H	-0.04772400	-3.48913500	2.44397500
H	-0.21656700	-3.35913000	4.91733000
C	0.10311200	-1.44701900	0.74636400
C	0.34131200	-2.05964700	-0.32552400
C	0.70136000	-2.35283100	-1.63688200
H	-0.10973300	-2.53320500	-2.34913300
C	1.98251700	-2.22296200	-2.12673800
H	2.09315900	-2.35108000	-3.20776400

C	3.24285700	-2.47301400	-1.34921000
H	3.01471300	-2.39459100	-0.27278900
N	4.22479000	-1.41352500	-1.74250400
C	3.56437200	-0.24015000	-2.33972400
H	3.51181500	-0.36495600	-3.42933000
H	4.15650300	0.67105600	-2.16506100
C	2.23986000	-0.04985500	-1.74714100
C	1.34248700	0.54691100	-1.06314000
H	1.60760900	1.50208800	-0.59458000
S	5.43601600	-1.05265200	-0.63205200
O	5.93546300	-2.33085200	-0.14221100
O	6.32803100	-0.11678500	-1.30649400
C	4.68207300	-0.20127600	0.72952000
C	4.09951100	-0.93559200	1.76358500
C	4.68465900	1.19230600	0.75079500
C	3.51859800	-0.25847500	2.82710600
H	4.13466300	-2.02365200	1.75780400
C	4.10440700	1.85157000	1.82709600
H	5.16822800	1.75226300	-0.04746700
C	3.52085300	1.14015100	2.88101600
H	3.07740700	-0.82436300	3.64839700
H	4.12999900	2.94093100	1.86682200
C	2.92122500	1.85744100	4.04866800
H	1.85652100	2.07133800	3.87099700
H	2.98194300	1.25539100	4.96395400
H	3.42015100	2.81805500	4.22962900
C	-3.21391700	2.53885000	-0.88336000
H	-3.34951000	3.59620800	-0.61061700
C	-3.78816100	2.28486800	-2.27415100
H	-3.33242200	2.98178000	-2.99297900
C	-3.65090400	0.85753400	-2.79425800
H	-4.21546300	0.77866200	-3.73357200
C	-4.16414700	-0.19098200	-1.80842700
H	-4.62952900	-1.03586400	-2.33262000
P	-2.86403100	-0.91058200	-0.68093800
P	-1.41313000	2.16025000	-0.72522100
Rh	-0.67932300	-0.03305900	-0.72783600
C	3.77335200	-3.85738500	-1.68469900
H	3.97432100	-3.93211100	-2.76187700
H	4.69974900	-4.06779000	-1.14175500
H	3.02665900	-4.61561500	-1.41598200
C	-0.95336700	2.95697900	0.85679800
C	0.33781600	3.46814900	1.04108000
C	-1.85484700	2.98578900	1.92693100
C	0.71528700	3.99861400	2.26963600
H	1.04699400	3.47459800	0.21217200
C	-1.46936900	3.50558000	3.15993700

H	-2.86818000	2.60330800	1.80979400
C	-0.18476900	4.01173400	3.33445800
H	1.71706100	4.40750400	2.39672400
H	-2.18190400	3.52119700	3.98340400
H	0.11635000	4.42209900	4.29721000
C	-0.70103000	3.23768400	-2.02326000
C	-0.82041500	4.63015200	-1.93094900
C	-0.12913300	2.67306800	-3.16574000
C	-0.36017900	5.44074600	-2.96226700
H	-1.27115600	5.08522100	-1.04809700
C	0.32616000	3.48684500	-4.20015900
H	-0.03776400	1.58849800	-3.24188400
C	0.21358000	4.86981100	-4.09701100
H	-0.45107700	6.52260200	-2.88104300
H	0.77063800	3.03806500	-5.08691800
H	0.57246100	5.50709600	-4.90353300
C	-2.72974700	-2.64013700	-1.26860500
C	-2.51143700	-3.69151200	-0.37255800
C	-2.71501500	-2.90238700	-2.64407400
C	-2.30285400	-4.98466700	-0.84348900
H	-2.51322700	-3.50128300	0.70129900
C	-2.50732000	-4.19663300	-3.11231300
H	-2.86290700	-2.09326300	-3.36160000
C	-2.30107100	-5.23949200	-2.21249000
H	-2.14473300	-5.79692900	-0.13590300
H	-2.50739600	-4.39003600	-4.18377400
H	-2.14031800	-6.25201700	-2.57874400
C	-3.73123300	-1.03659400	0.92301400
C	-4.91202600	-1.77888600	1.05464800
C	-3.24196500	-0.32974000	2.02135800
C	-5.57967700	-1.81323500	2.27331300
H	-5.30678400	-2.33561600	0.20358200
C	-3.91183900	-0.36212100	3.24194300
H	-2.32511000	0.24906900	1.91683600
C	-5.08020500	-1.10641500	3.36771200
H	-6.49518700	-2.39422900	2.37178100
H	-3.51397200	0.19483900	4.09035400
H	-5.60758200	-1.13829200	4.31970500
H	-4.94726600	0.23438900	-1.16408500
H	-2.60032000	0.64503100	-3.05839300
H	-4.85779600	2.54182600	-2.23431200
H	-3.74798500	1.94687400	-0.12519200

TS$_{1aRh\text{-}A}$

E (M06-SMD/BS1) = -3328.47411767 au

H (M06-SMD/BS1) = -3327.561784 au

G (M06-SMD/BS1) = -3327.700765 au

E (M06-SMD/BS2//M06-SMD/BS1) = -3330.49403641 au

C	-2.64670000	5.71690100	-2.26088600
C	-3.27936000	4.47650500	-2.33524400
C	-2.53269200	3.30851700	-2.26243900
C	-1.13794000	3.37048700	-2.11050200
C	-0.50757400	4.62238400	-2.03925100
C	-1.26178600	5.78604800	-2.11459800
H	-3.23397700	6.63177000	-2.31867100
H	-4.36042800	4.42071100	-2.45232700
H	-3.01506400	2.33146400	-2.31036800
H	0.57488900	4.66631700	-1.92282400
H	-0.76715400	6.75420800	-2.05816300
C	-0.38343500	2.17118300	-2.00688900
C	0.24092700	1.12977000	-1.90436200
C	0.99664300	-0.07326800	-1.85031800
H	2.02403400	0.01472300	-2.20665400
C	0.36347200	-1.35388800	-2.10433500
H	1.06637300	-2.10011400	-2.48946400
C	-0.95897600	-1.31496100	-2.85594900
H	-1.55895800	-0.47965000	-2.45536500
N	-1.68948700	-2.58805500	-2.65768900
C	-1.02305300	-3.44810000	-1.68480800
H	-0.24374700	-4.02924800	-2.19981800
H	-1.72916300	-4.16483500	-1.25194000
C	-0.41680700	-2.60239900	-0.62949300
C	-0.34042600	-2.43316300	0.63577100
H	-0.73737300	-2.85800600	1.55024400
S	-3.36165100	-2.48269300	-2.56560600
O	-3.76850700	-1.69287100	-3.72080600
O	-3.83561100	-3.85024600	-2.38979200
C	-3.80172100	-1.56277700	-1.10885800
C	-4.26321700	-0.25498600	-1.24027800
C	-3.73078100	-2.17725400	0.14236900
C	-4.66793900	0.43569400	-0.10380900
H	-4.34194600	0.19737500	-2.22720700
C	-4.10478500	-1.45966300	1.27010300
H	-3.41273400	-3.21472100	0.23356400
C	-4.58695200	-0.14848500	1.16421800
H	-5.06764700	1.44562600	-0.20470600
H	-4.05482000	-1.93523800	2.25051800
C	-4.97869800	0.61800700	2.38746900
H	-4.09769700	1.10931000	2.82984800
H	-5.70710500	1.40491000	2.15609500
H	-5.40438700	-0.03830000	3.15683000
C	1.90606100	0.52347000	3.33790500
H	1.49987600	1.12586900	4.16454600
C	2.69447200	-0.65649100	3.89688000

H	2.07162600	-1.21254400	4.61217400
C	3.25721300	-1.61574200	2.85507800
H	3.92305800	-2.32569000	3.36451100
C	4.04770200	-0.91185500	1.75763300
H	4.83953800	-1.56665100	1.37179200
P	3.10583700	-0.34702600	0.24395000
P	0.47789200	0.05931200	2.26769200
Rh	0.83537400	-0.83668000	0.15075900
C	-0.68066900	-1.08946600	-4.33358700
H	-0.05859400	-1.90401000	-4.72890400
H	-1.62198800	-1.06810200	-4.89322300
H	-0.15747600	-0.13769200	-4.49085200
C	-0.35934000	1.64030000	1.90760800
C	-1.61196900	1.60803100	1.27928000
C	0.21214900	2.87535000	2.22810500
C	-2.29340500	2.79016800	1.01852600
H	-2.06831800	0.65026400	1.00866100
C	-0.46044300	4.05907100	1.93072300
H	1.18524300	2.93030800	2.71282400
C	-1.71821100	4.01866800	1.33995600
H	-3.27622700	2.75439100	0.55020300
H	-0.00135600	5.01441800	2.18017300
H	-2.25056600	4.94285200	1.11964400
C	-0.62629100	-0.76381200	3.48828100
C	-1.66532900	-0.06528700	4.11343100
C	-0.38265700	-2.09044600	3.86867500
C	-2.45472300	-0.68622000	5.07828900
H	-1.86305600	0.97458600	3.85611200
C	-1.17126300	-2.70808100	4.83436000
H	0.43520700	-2.64998100	3.41389800
C	-2.21458000	-2.00921100	5.43598200
H	-3.26027300	-0.12744100	5.55262000
H	-0.96811700	-3.73982200	5.11601800
H	-2.83501500	-2.49423900	6.18754100
C	3.83192600	-1.40986900	-1.06026700
C	4.32737300	-0.90746300	-2.26656100
C	3.78578900	-2.79703300	-0.86323500
C	4.76966000	-1.77916100	-3.25972600
H	4.37471800	0.16835900	-2.43713800
C	4.23015500	-3.66295100	-1.85540800
H	3.39551300	-3.20547400	0.07120800
C	4.71981600	-3.15459600	-3.05776600
H	5.15673700	-1.37676400	-4.19425100
H	4.19413500	-4.73840500	-1.68986900
H	5.06543800	-3.83306400	-3.83573400
C	3.73930200	1.34074900	-0.03955500
C	5.11540600	1.58753500	-0.13907400

C	2.84474300	2.41249500	-0.06142700
C	5.58047100	2.89088600	-0.26789600
H	5.82356500	0.75778600	-0.11745600
C	3.31490700	3.71863900	-0.17518600
H	1.77370200	2.22381300	0.02105700
C	4.68114600	3.95695800	-0.28240700
H	6.64974600	3.07779400	-0.35077600
H	2.60778400	4.54759800	-0.17319500
H	5.05139700	4.97665900	-0.37444500
H	4.55112200	-0.02201800	2.16234700
H	2.45276500	-2.22306600	2.40998200
H	3.52901500	-0.23884600	4.48023300
H	2.56006000	1.18640700	2.75107800

TS$_{A-B}$

E (M06-SMD/BS1) = -3328.50192683 au
H (M06-SMD/BS1) = -3327.588308 au
G (M06-SMD/BS1) = -3327.726850 au
E (M06-SMD/BS2//M06-SMD/BS1) = -3330.51505009 au

C	1.34669500	-4.10629000	-3.07443900
C	1.57624800	-3.91915700	-1.71197100
C	1.44731000	-2.65657300	-1.14843600
C	1.08873300	-1.56484000	-1.95089900
C	0.87650300	-1.75207700	-3.32510600
C	1.00634500	-3.02056600	-3.87922200
H	1.43623400	-5.09983400	-3.50998700
H	1.85186400	-4.76452400	-1.08243000
H	1.60477400	-2.50712200	-0.07977500
H	0.58993400	-0.90151800	-3.94242600
H	0.83138000	-3.16303500	-4.94419600
C	0.84577500	-0.27498900	-1.36339400
C	0.97547800	0.99426000	-1.34013700
C	0.78968400	2.15201800	-0.62736700
H	0.17927600	2.96024700	-1.03584600
C	1.67899500	2.44556300	0.55678100
H	1.17393600	3.22014700	1.16333900
C	3.10399000	2.94401700	0.23127500
H	3.40459900	2.50267800	-0.73396000
N	3.94422600	2.36303000	1.31995200
C	3.15217300	1.46171500	2.19173800
H	2.93440600	1.98854800	3.13272000
H	3.70127300	0.54857300	2.44740000
C	1.91029200	1.21215100	1.39121200
C	1.15603100	0.11790800	1.31370000
H	1.36054100	-0.81643000	1.84297500
S	5.45956700	1.82558000	0.87098800
O	6.06880100	2.91583800	0.11736000

O	6.08891800	1.31788700	2.08479800
C	5.23147100	0.46209700	-0.24460900
C	5.18156600	0.70364300	-1.61874200
C	5.09395100	-0.83041300	0.25945700
C	5.00379100	-0.36351100	-2.48739200
H	5.31788500	1.71310400	-2.00185300
C	4.92059100	-1.88673900	-0.62713200
H	5.16095800	-1.01556700	1.32989200
C	4.88373300	-1.67271700	-2.00818500
H	4.98308400	-0.18530500	-3.56284900
H	4.83340300	-2.90316900	-0.24232900
C	4.73716000	-2.81105200	-2.96818800
H	5.54960300	-2.80680500	-3.70754500
H	4.74860400	-3.78000400	-2.45511900
H	3.79417100	-2.73572800	-3.52873100
C	-2.96750500	-0.92128900	2.32804400
C	-2.72334200	0.15291700	3.39187000
C	-1.98219100	1.43101800	3.01382000
C	-2.65317900	2.38314000	2.02240700
P	-2.63708700	1.80879700	0.26414500
P	-1.59507100	-1.44264200	1.18803700
Rh	-0.47098800	0.38054700	0.18182200
C	3.21367800	4.44999800	0.17231300
H	2.94611000	4.88925600	1.14299000
H	4.23266300	4.76199500	-0.08169500
H	2.52788500	4.84762200	-0.58915300
H	-3.66431000	2.65501500	2.35712700
H	-2.06986900	3.31640100	2.00364200
H	-1.82461600	1.99682900	3.94380300
H	-0.97075500	1.18670400	2.64174900
H	-3.71482800	0.42632500	3.78878700
H	-2.18646100	-0.30212600	4.23660400
H	-3.32690900	-1.83021100	2.83179300
H	-3.77185000	-0.61444400	1.64407000
C	-2.48867900	-2.50035500	-0.00843700
C	-3.44826300	-3.42498100	0.42311000
C	-2.21185300	-2.39467300	-1.37319900
C	-4.12018400	-4.21866700	-0.49972900
H	-3.66908300	-3.53909300	1.48423300
C	-2.88053200	-3.19408100	-2.29645100
H	-1.46866600	-1.67483000	-1.71530000
C	-3.83748700	-4.10493100	-1.85972600
H	-4.86570300	-4.93272700	-0.15421500
H	-2.65348000	-3.10121600	-3.35752000
H	-4.36387400	-4.73037200	-2.57886100
C	-0.58852500	-2.61759800	2.16174900
C	-0.09711100	-2.23608500	3.41708000

C	-0.24341300	-3.87248700	1.64865300
C	0.70862700	-3.10102600	4.15052100
H	-0.33497500	-1.25335600	3.82555100
C	0.56954500	-4.73224400	2.38211700
H	-0.61428500	-4.18569500	0.67286400
C	1.04416100	-4.35002200	3.63347900
H	1.07752600	-2.79577000	5.12822700
H	0.82741200	-5.70804300	1.97323200
H	1.67677900	-5.02517900	4.20714400
C	-4.28063700	1.07835100	-0.10904500
C	-4.36959800	0.21674300	-1.20730900
C	-5.43391600	1.36561300	0.62768900
C	-5.58474400	-0.36497300	-1.55361800
H	-3.47598600	-0.01116300	-1.79114600
C	-6.64838600	0.77990000	0.28200200
H	-5.39694100	2.04595300	1.47807900
C	-6.72486800	-0.08892800	-0.80370700
H	-5.63616300	-1.04198100	-2.40516200
H	-7.53993900	1.00527000	0.86499900
H	-7.67550500	-0.55005600	-1.06621000
C	-2.70951700	3.34418900	-0.73570800
C	-3.27435500	4.52848500	-0.25279000
C	-2.23587100	3.29792200	-2.05175400
C	-3.34607100	5.65289700	-1.07024900
H	-3.66647100	4.57958400	0.76302300
C	-2.31690200	4.42057900	-2.86982800
H	-1.79787400	2.37472000	-2.43818100
C	-2.86733700	5.60098400	-2.37682700
H	-3.78285300	6.57274100	-0.68484100
H	-1.94441500	4.37477800	-3.89179500
H	-2.92444000	6.48251200	-3.01311700

$TS_{B\text{-}F}$
E (M06-SMD/BS1) = -3328.46851413 au
H (M06-SMD/BS1) = -3327.560284 au
G (M06-SMD/BS1) = -3327.701303 au
E (M06-SMD/BS2//M06-SMD/BS1) = -3330.48725687 au

C	-0.46089900	4.94796400	-3.77451300
C	0.04945300	3.74465200	-4.26152000
C	0.49311900	2.76939600	-3.37940800
C	0.43684600	3.00016400	-1.99253600
C	-0.07399400	4.21726600	-1.50882600
C	-0.52360200	5.18046600	-2.40077800
H	-0.80994400	5.70944700	-4.46960200
H	0.10002200	3.56667500	-5.33408600
H	0.89000300	1.82168300	-3.74337500
H	-0.11501800	4.38834700	-0.43353000

H	-0.92144800	6.12110000	-2.02476200
C	0.87218800	1.99035200	-1.10122400
C	1.25757900	1.06760100	-0.39446600
C	1.58497300	0.00594100	0.46298100
H	1.90630700	0.25277900	1.47720700
C	1.66779800	-1.37350500	0.06169600
H	0.51614000	-1.90088700	0.71338500
C	2.74016600	-2.35583500	0.54216400
H	3.40924300	-1.89560900	1.27946300
N	3.48127700	-2.58559700	-0.72800700
C	2.57313300	-2.49053300	-1.89927400
H	2.31548500	-3.49992300	-2.24711100
H	3.07346300	-1.98029800	-2.73178100
C	1.38479000	-1.74634100	-1.35408700
C	0.11510100	-1.45797800	-1.64869100
H	-0.42397500	-1.70284800	-2.56718000
S	5.00746100	-1.89839600	-0.85577800
O	5.70858000	-2.27039800	0.36710300
O	5.51114100	-2.30287800	-2.16191900
C	4.80360700	-0.13660700	-0.85695400
C	4.80514800	0.55396100	0.35702500
C	4.58208600	0.52898400	-2.06132400
C	4.56378400	1.91999100	0.35631900
H	5.00502000	0.02607100	1.28807000
C	4.34432700	1.89813400	-2.04081400
H	4.61239000	-0.01341500	-3.00448200
C	4.32267400	2.60970200	-0.83779400
H	4.55653700	2.46616000	1.30048900
H	4.16781400	2.42746600	-2.97719900
C	4.04896800	4.08080900	-0.81521700
H	4.95356700	4.64648600	-0.55064100
H	3.70167700	4.44361800	-1.79016400
H	3.28813800	4.32938000	-0.06252100
C	-3.89842500	-1.54023300	1.06453700
C	-3.56776100	-2.67492600	2.03052000
C	-2.27540800	-2.54529100	2.83407100
C	-2.07892800	-1.17402800	3.48318500
P	-1.12635900	0.04011800	2.43491900
P	-2.71334500	-1.29020500	-0.32460600
Rh	-0.51873700	-0.68475500	0.18237500
C	2.20608500	-3.66891000	1.07438900
H	1.52440700	-4.14009300	0.35177400
H	3.03624500	-4.35832100	1.26675300
H	1.65929300	-3.50840100	2.01384500
H	-3.97872100	-0.57788400	1.59425400
H	-4.88310200	-1.73767600	0.61431800
H	-4.40948500	-2.73453700	2.73740400

H	-3.56279100	-3.63114300	1.48564400
H	-2.29573400	-3.31115300	3.62197900
H	-1.40487800	-2.78920300	2.20308900
H	-1.53738800	-1.26836800	4.43346600
H	-3.04885700	-0.71661500	3.72735500
C	-2.07545900	1.60102900	2.51287400
C	-2.54289100	2.11047500	3.73103700
C	-2.31770300	2.31247500	1.33464600
C	-3.24697000	3.30843600	3.76349900
H	-2.34441500	1.57198500	4.65910700
C	-3.02199100	3.51464800	1.36883900
H	-1.96139000	1.91792900	0.37989200
C	-3.48734200	4.01038300	2.58243800
H	-3.60785600	3.69934300	4.71334600
H	-3.20585600	4.06072400	0.44442100
H	-4.03874000	4.94874600	2.61154000
C	-2.97085400	-2.78815900	-1.34099000
C	-4.04646900	-2.87592100	-2.22975000
C	-2.12373100	-3.88827600	-1.17449400
C	-4.26681600	-4.04846400	-2.94609100
H	-4.71528400	-2.02527100	-2.36589800
C	-2.35173000	-5.06240500	-1.88534500
H	-1.27835400	-3.82273000	-0.48643500
C	-3.42071800	-5.14100000	-2.77452300
H	-5.10224300	-4.10740600	-3.64173900
H	-1.68906300	-5.91537300	-1.74932900
H	-3.59449500	-6.05703600	-3.33668100
C	-3.43269200	0.10707100	-1.26956800
C	-4.72571100	0.59084900	-1.04253200
C	-2.61283200	0.76979100	-2.19386200
C	-5.18415200	1.71751100	-1.72075700
H	-5.38581900	0.10085800	-0.32891300
C	-3.07314300	1.89497600	-2.86905600
H	-1.59500300	0.41473700	-2.36852100
C	-4.35986900	2.37261200	-2.63073700
H	-6.19106000	2.08581400	-1.53148800
H	-2.41861200	2.40379600	-3.57691200
H	-4.71859100	3.25771800	-3.15345500
C	0.36885100	0.38420200	3.43531600
C	1.09043700	-0.69330800	3.96309000
C	0.91739700	1.66894200	3.49981800
C	2.33786400	-0.48916500	4.54501100
H	0.68476700	-1.70593100	3.90864100
C	2.16539700	1.87074600	4.08431400
H	0.37554600	2.51701600	3.07909100
C	2.87966700	0.79353400	4.60298800
H	2.88896700	-1.33487900	4.95350400

H	2.58094200	2.87612500	4.13365400
H	3.85675100	0.95290700	5.05583300

TS$_{B-C}$
E (M06-SMD/BS1) = -3328.50739832 au
H (M06-SMD/BS1) = -3327.594188 au
G (M06-SMD/BS1) = -3327.732405 au
E (M06-SMD/BS2//M06-SMD/BS1) = -3330.51953972 au

C	3.03073000	-4.31811600	-1.24141700
C	2.57122700	-3.94889600	0.01949000
C	1.86316600	-2.76452100	0.19143800
C	1.61127400	-1.92213000	-0.89957900
C	2.09259800	-2.29845300	-2.16686900
C	2.78952000	-3.48626900	-2.33421400
H	3.57289800	-5.25288300	-1.37532500
H	2.75194000	-4.59176400	0.87968400
H	1.49329800	-2.50951900	1.18259400
H	1.88686600	-1.65616900	-3.02239200
H	3.14218300	-3.76788400	-3.32541100
C	0.80999300	-0.71150100	-0.81470500
C	0.45402100	0.24144700	-1.67791800
C	0.18083600	1.56502600	-1.38431300
H	-0.35934200	2.22450100	-2.06464000
C	1.01478700	2.21136400	-0.28764200
H	0.41613700	3.04641900	0.11727200
C	2.39062400	2.75433900	-0.70061700
H	2.81839500	2.05113100	-1.43906400
N	3.16501900	2.68082100	0.57328600
C	2.54718000	1.73528400	1.53574700
H	2.31642200	2.26124300	2.47303300
H	3.20698300	0.89117700	1.78560500
C	1.31947400	1.27231700	0.82229800
C	0.64846400	0.10833100	0.96310900
H	0.87126800	-0.61461000	1.74841400
S	4.82892400	2.57974800	0.46787400
O	5.24934200	3.56416400	-0.52184200
O	5.32145200	2.64868700	1.83893400
C	-3.66287300	-1.51489600	1.85797100
H	-4.10474400	-2.51047800	2.01582500
C	-3.52546900	-0.79615900	3.19540700
H	-2.92538500	-1.40534700	3.88673800
C	-2.95764600	0.61788800	3.13327000
H	-3.06808300	1.07381400	4.12670500
C	-3.65143500	1.50463400	2.09560700
H	-3.75734300	2.53340800	2.46316300
P	-2.77009300	1.61006400	0.45929600
P	-2.11286600	-1.77906400	0.89824500

Rh	-1.05290100	0.02138200	-0.13213600
C	2.31872600	4.15354900	-1.26727400
H	1.95097100	4.84879300	-0.49964900
H	3.29418600	4.50366600	-1.61840200
H	1.61636500	4.17050900	-2.11297600
H	-4.67168000	1.14742600	1.89331000
H	-1.87259200	0.58527400	2.93832500
H	-4.53518700	-0.74954300	3.63109400
H	-4.34780700	-0.96375100	1.19615000
C	-1.15192800	-2.92747700	1.94354500
C	-1.04484900	-4.29066200	1.65257000
C	-0.50849800	-2.41663400	3.07965000
C	-0.29472600	-5.12515500	2.47658700
H	-1.54400300	-4.70703900	0.77854800
C	0.23714100	-3.25474200	3.90214500
H	-0.58691400	-1.35638800	3.32389900
C	0.35050400	-4.60935800	3.59701900
H	-0.21548800	-6.18475300	2.23944000
H	0.73226500	-2.84790300	4.78210000
H	0.93903800	-5.26429500	4.23719000
C	-2.65814100	-2.71934200	-0.57328600
C	-1.68052700	-3.18900800	-1.46356800
C	-4.00822700	-2.89048300	-0.89921400
C	-2.04393000	-3.81438400	-2.64903800
H	-0.62386400	-3.07287500	-1.22136600
C	-4.36870400	-3.50936900	-2.09400800
H	-4.79473200	-2.54323700	-0.23220500
C	-3.39125500	-3.96797100	-2.97107600
H	-1.27055300	-4.17781400	-3.32405300
H	-5.42284700	-3.63328100	-2.33619600
H	-3.67793000	-4.44946200	-3.90441900
C	-4.08208100	1.60111900	-0.80561600
C	-5.20410600	2.43359100	-0.71985900
C	-3.95827600	0.71670900	-1.88116100
C	-6.19061500	2.37248300	-1.69676500
H	-5.30001700	3.13591900	0.10970400
C	-4.94993300	0.65393300	-2.85754000
H	-3.07724300	0.06848000	-1.94809000
C	-6.06450500	1.48185800	-2.76301200
H	-7.06184800	3.02180600	-1.62952200
H	-4.85030000	-0.04227000	-3.68877100
H	-6.84101500	1.43588300	-3.52487500
C	-2.12684900	3.32357500	0.47082800
C	-1.51866500	3.81698600	1.63105900
C	-2.12514100	4.10576100	-0.68843500
C	-0.92288500	5.07518100	1.63200300
H	-1.49972600	3.21515400	2.54145000

C	-1.52835500	5.36276600	-0.68453400
H	-2.59118900	3.73130400	-1.60069600
C	-0.92612500	5.84937900	0.47422500
H	-0.45416200	5.45048900	2.54016400
H	-1.53716500	5.96677000	-1.59047100
H	-0.45995100	6.83321400	0.47459000
C	5.20947300	0.96498800	-0.16834500
C	5.48533500	-0.07497200	0.71860000
C	5.22361600	0.75218600	-1.54830300
C	5.78959500	-1.33305100	0.21386600
H	5.49483600	0.10918200	1.79130000
C	5.52818100	-0.51253000	-2.03292400
H	5.03235800	1.57510900	-2.23489400
C	5.82877700	-1.56756100	-1.16349000
H	6.01605800	-2.14878500	0.90052600
H	5.55268500	-0.68562800	-3.10907200
C	6.23655400	-2.90349000	-1.70041800
H	6.05289900	-3.70445200	-0.97390000
H	5.70548700	-3.14560400	-2.62966200
H	7.31188300	-2.91203000	-1.93129400

$TS_{C\text{-}B}$
E (M06-SMD/BS1) = -3328.5097495 au
H (M06-SMD/BS1) = -3327.600243 au
G (M06-SMD/BS1) = -3327.738661 au
E (M06-SMD/BS2//M06-SMD/BS1) = -3330.52300108 au

C	-0.21322700	5.30728700	-2.30898500
C	0.74642000	4.41565300	-2.78317000
C	1.01704400	3.24359900	-2.08596200
C	0.33910900	2.94134100	-0.89446600
C	-0.62113900	3.85209700	-0.42732500
C	-0.89691700	5.01911400	-1.12913000
H	-0.42631600	6.22439800	-2.85570600
H	1.29439300	4.63714900	-3.69764800
H	1.78988500	2.57429300	-2.46178700
H	-1.15424800	3.62238400	0.49366600
H	-1.65135400	5.70816000	-0.75216700
C	0.62043500	1.70484700	-0.13492800
C	0.34365800	1.64817400	1.26868600
C	0.78415100	0.47641200	1.93605200
H	0.52888700	0.32741100	2.98931100
C	1.56452800	-0.58527800	1.31825800
C	2.66961700	-1.47796500	1.88232400
H	3.23656200	-0.91633900	2.63568300
N	3.53465500	-1.73766100	0.69260900
C	2.72112400	-1.53272700	-0.52690700
H	2.20595500	-2.47306000	-0.77224600

H	3.34189000	-1.26460200	-1.38898700
C	1.76086800	-0.46184900	-0.10402000
C	1.21125800	0.59650200	-0.82863300
H	1.33498300	0.59878400	-1.91339600
S	5.04162700	-0.97077800	0.68311700
O	5.59749800	-1.19508800	2.01149300
O	5.72133700	-1.46192800	-0.50851200
C	-2.95245000	-2.68398300	-1.27604600
H	-3.17082000	-3.09987200	-2.26956600
C	-2.98097500	-3.81489200	-0.25298400
H	-2.33548300	-4.63813800	-0.58827400
C	-2.63291000	-3.43601100	1.18102100
H	-2.86513200	-4.29158600	1.82945400
C	-3.40911600	-2.21496800	1.67269500
H	-3.67712700	-2.31724000	2.73211300
P	-2.52467700	-0.58779600	1.53012400
P	-1.42541100	-1.66315500	-1.46705700
Rh	-0.55981600	-0.46824600	0.31734500
C	2.19861800	-2.78169200	2.49152900
H	1.68460400	-3.39953100	1.74005300
H	3.05202800	-3.34731900	2.88270800
H	1.50171800	-2.58149500	3.31743700
H	-4.35758400	-2.11670600	1.12611700
H	-1.54808100	-3.26696800	1.28431700
H	-4.00624900	-4.21572800	-0.26719600
H	-3.74731300	-1.94699100	-1.07687400
C	4.73935200	0.76553800	0.46978800
C	4.43180300	1.55235700	1.58164300
C	4.72366700	1.30510800	-0.81606300
C	4.06923700	2.87800200	1.39035200
H	4.48570200	1.13617900	2.58622000
C	4.36839800	2.63819500	-0.98521200
H	4.99841400	0.69077100	-1.67172600
C	4.01613300	3.43657800	0.10750100
H	3.82239000	3.49765900	2.25244500
H	4.36348600	3.07099700	-1.98581600
C	-3.80721200	0.61902200	1.06185200
C	-5.08775400	0.55504600	1.62564500
C	-3.49920400	1.64414400	0.16715100
C	-6.04693900	1.49810100	1.27586700
H	-5.33520500	-0.22464900	2.34680400
C	-4.46080700	2.58831400	-0.18146400
H	-2.49366600	1.70555900	-0.24886900
C	-5.73612700	2.51135400	0.36958900
H	-7.04213100	1.44392700	1.71367500
H	-4.20976900	3.38169900	-0.88469800
H	-6.49247900	3.24558100	0.09755800

C	-2.12317800	-0.19237900	3.26828600
C	-1.58798500	-1.19511000	4.08598700
C	-2.24561600	1.10828300	3.76129700
C	-1.17877000	-0.89769400	5.38089100
H	-1.48321400	-2.21459300	3.70977500
C	-1.83456100	1.40184700	5.05914400
H	-2.65900200	1.89689900	3.13216500
C	-1.29894300	0.40331700	5.86730800
H	-0.76670300	-1.68303400	6.01259600
H	-1.93469600	2.41711400	5.43886600
H	-0.97719800	0.63695500	6.88073900
C	-1.90388400	-0.59062100	-2.87480200
C	-2.47420100	-1.13145100	-4.03567500
C	-1.71802400	0.78987700	-2.79373700
C	-2.85798500	-0.29730900	-5.07936900
H	-2.60618700	-2.20873500	-4.13893300
C	-2.10479600	1.62659300	-3.83674700
H	-1.26337100	1.21408700	-1.90077500
C	-2.67950000	1.08204500	-4.98013700
H	-3.29916900	-0.72713400	-5.97696000
H	-1.95193800	2.70224900	-3.74865600
H	-2.98670500	1.72966500	-5.79955500
C	-0.15170200	-2.79181200	-2.14240600
C	0.21477900	-3.92844100	-1.40808800
C	0.52489500	-2.50708500	-3.33320900
C	1.20995800	-4.78073500	-1.87534100
H	-0.26572400	-4.14846100	-0.45454100
C	1.53094100	-3.35513000	-3.79043600
H	0.26467100	-1.62282600	-3.91460500
C	1.86998300	-4.49537200	-3.06847700
H	1.47634300	-5.66549100	-1.29976600
H	2.04824400	-3.12250700	-4.71974300
H	2.65331300	-5.15852300	-3.43091800
C	3.56076000	4.84907800	-0.08213400
H	3.80797700	5.22547200	-1.08206900
H	2.46828600	4.91900400	0.03826500
H	4.00824100	5.51927500	0.66303700
H	0.42576900	-1.51854400	1.24787800

$TS_{E\text{-}2aRh}$

E (M06-SMD/BS1) = -3328.58536891 au
H (M06-SMD/BS1) = -3327.675933 au
G (M06-SMD/BS1) = -3327.813800 au
E (M06-SMD/BS2//M06-SMD/BS1) = -3330.59698905 au

C	-2.01788200	4.31245900	-1.16535000
C	-1.69824500	3.62235300	-2.33877300
C	-0.72890500	2.63164300	-2.32637000

C	-0.06015700	2.30450800	-1.13310200
C	-0.41155700	2.98339200	0.04948500
C	-1.38319500	3.98824300	0.02383100
H	-2.77106600	5.09852100	-1.18404900
H	-2.20738000	3.87014100	-3.26904600
H	-0.46470100	2.10434900	-3.24337300
H	0.15423900	2.79541300	0.96237400
H	-1.61986400	4.53078500	0.93759500
C	1.04420900	1.31706400	-1.11873300
C	0.94557300	0.25956200	-0.19636300
C	2.00101400	-0.64862200	-0.08053600
H	1.97848100	-1.46682800	0.63835100
C	3.09237600	-0.51508600	-0.92643400
C	4.31614300	-1.38623400	-1.01346800
H	4.75078600	-1.61181200	-0.03105000
N	5.26946200	-0.54545900	-1.79993500
C	4.45898700	0.38619300	-2.62650900
H	4.32199900	-0.06045400	-3.62214500
H	4.98458300	1.33586600	-2.77904200
C	3.16144100	0.49928400	-1.87749100
C	2.13526900	1.42597400	-1.98792400
H	2.17986700	2.24653700	-2.70613300
S	6.47917400	0.19200300	-0.89072600
O	7.09416700	-0.87032200	-0.10282600
O	7.27453200	0.97109900	-1.83284700
C	-2.55611300	-2.43702700	2.06129000
H	-2.38742200	-3.51125900	2.23313700
C	-2.72869800	-1.73319000	3.40198300
H	-1.82532000	-1.86116100	4.01649500
C	-3.10521800	-0.25656600	3.33041500
H	-3.46403100	0.05213300	4.32191100
C	-4.18781600	0.04802600	2.29425000
H	-4.78655700	0.91116400	2.60779700
P	-3.51028300	0.47127400	0.60767800
P	-1.14604800	-1.89267400	1.00666900
Rh	-1.02361100	0.28015300	0.38381200
C	4.04242400	-2.69256900	-1.74045100
H	3.63619300	-2.50604900	-2.74453800
H	4.96173900	-3.28259400	-1.83744000
H	3.30426000	-3.28358200	-1.17965200
H	-4.89212000	-0.79155200	2.20329900
H	-2.21459100	0.35959400	3.12350600
H	-3.53058100	-2.27263500	3.92846200
H	-3.47386500	-2.35420000	1.46212100
C	5.66942400	1.32109800	0.21541600
C	5.14938900	0.85163200	1.42048300
C	5.45312800	2.63764600	-0.19258100

C	4.38521600	1.70549400	2.20641700
H	5.34188200	-0.16914400	1.74627100
C	4.69138900	3.47710600	0.60743600
H	5.88343000	3.00027600	-1.12405700
C	4.13777500	3.02330900	1.81009500
H	3.97444000	1.34359300	3.14945600
H	4.52042300	4.50808600	0.29717000
C	-4.29759500	-0.61656600	-0.62681400
C	-5.56258400	-1.18930600	-0.46584700
C	-3.58934100	-0.83542900	-1.81296000
C	-6.09588300	-1.99191900	-1.46992800
H	-6.14124100	-1.00363700	0.43920400
C	-4.13347000	-1.61855200	-2.82626700
H	-2.59954000	-0.38731600	-1.93776200
C	-5.38358900	-2.20598500	-2.64825700
H	-7.07713500	-2.44421500	-1.33610400
H	-3.57612300	-1.78096000	-3.74741200
H	-5.80736600	-2.83097900	-3.43260300
C	-4.30632500	2.09630600	0.29377700
C	-4.22860200	3.08592400	1.28149200
C	-4.98048700	2.37479000	-0.89750800
C	-4.84683900	4.31696900	1.09653300
H	-3.68184800	2.89395600	2.20753700
C	-5.59252600	3.61276300	-1.08421800
H	-5.04773100	1.62022000	-1.68117200
C	-5.53486900	4.58109000	-0.08680000
H	-4.79106600	5.07291400	1.87845700
H	-6.12589700	3.81411600	-2.01192400
H	-6.02408000	5.54313800	-0.23028200
C	-1.29136600	-2.94210200	-0.48968400
C	-2.30605400	-3.89669700	-0.62497200
C	-0.37073500	-2.77980000	-1.53505700
C	-2.39673100	-4.66907100	-1.77989600
H	-3.04358800	-4.04974800	0.15969100
C	-0.45918000	-3.55998500	-2.68176200
H	0.42615100	-2.04028900	-1.45142800
C	-1.47556300	-4.50473600	-2.80859500
H	-3.19629000	-5.40233900	-1.87165200
H	0.26847100	-3.42628100	-3.48062100
H	-1.54820600	-5.11083400	-3.70995000
C	0.28562600	-2.55325800	1.93818900
C	0.82596800	-1.82288400	3.00307100
C	0.84400800	-3.79006100	1.60107500
C	1.92173400	-2.31317300	3.70487400
H	0.40748200	-0.85030900	3.26886600
C	1.94609900	-4.27356500	2.30101900
H	0.43311000	-4.37477300	0.77852400

C	2.48995200	-3.53397600	3.34698500
H	2.33987600	-1.73475600	4.52686800
H	2.38357800	-5.23055800	2.02195100
H	3.35796800	-3.90933600	3.88615400
C	3.33115400	3.94931000	2.66633700
H	3.98204800	4.66525000	3.18815600
H	2.62780700	4.53919900	2.06452600
H	2.76236400	3.40348900	3.42875000
H	0.12863700	0.41822500	1.43378200
